# Multiscale transcriptomic network models of Parkinson’s disease at the single-cell level

**DOI:** 10.1093/gigascience/giag096

**Published:** 2026-10-06

**Authors:** Linh Chu, Gefei Yu, Xianxiao Zhou, Minghui Wang, Zhenyu Yue, Bin Zhang

**Affiliations:** Department of Genetics and Genomic Sciences, Icahn School of Medicine at Mount Sinai, New York, NY 10029, USA; Mount Sinai Center for Transformative Disease Modeling, Icahn School of Medicine at Mount Sinai, New York, NY 10029, USA; Department of Neurology, Icahn School of Medicine at Mount Sinai, New York, NY 10029, USA; Department of Pharmacological Sciences, Icahn School of Medicine at Mount Sinai, New York, NY 10029, USA; Department of Genetics and Genomic Sciences, Icahn School of Medicine at Mount Sinai, New York, NY 10029, USA; Mount Sinai Center for Transformative Disease Modeling, Icahn School of Medicine at Mount Sinai, New York, NY 10029, USA; Icahn Institute of Genomics, Icahn School of Medicine at Mount Sinai, New York, NY 10029, USA; Department of Genetics and Genomic Sciences, Icahn School of Medicine at Mount Sinai, New York, NY 10029, USA; Mount Sinai Center for Transformative Disease Modeling, Icahn School of Medicine at Mount Sinai, New York, NY 10029, USA; Icahn Institute of Genomics, Icahn School of Medicine at Mount Sinai, New York, NY 10029, USA; Department of Genetics and Genomic Sciences, Icahn School of Medicine at Mount Sinai, New York, NY 10029, USA; Mount Sinai Center for Transformative Disease Modeling, Icahn School of Medicine at Mount Sinai, New York, NY 10029, USA; Icahn Institute of Genomics, Icahn School of Medicine at Mount Sinai, New York, NY 10029, USA; Department of Neurology, Icahn School of Medicine at Mount Sinai, New York, NY 10029, USA; Nash Family Department of Neuroscience, Icahn School of Medicine at Mount Sinai, New York, NY 10029, USA; Friedman Brain Institute, Icahn School of Medicine at Mount Sinai, New York, NY 10029, USA; Center of Parkinson’s Disease Neurobiology, Icahn School of Medicine at Mount Sinai, New York, New York, NY 10029, USA; Department of Genetics and Genomic Sciences, Icahn School of Medicine at Mount Sinai, New York, NY 10029, USA; Mount Sinai Center for Transformative Disease Modeling, Icahn School of Medicine at Mount Sinai, New York, NY 10029, USA; Department of Pharmacological Sciences, Icahn School of Medicine at Mount Sinai, New York, NY 10029, USA; Icahn Institute of Genomics, Icahn School of Medicine at Mount Sinai, New York, NY 10029, USA; Department of Artificial Intelligence & Human Health, Icahn School of Medicine at Mount Sinai, New York, NY 10029, USA

**Keywords:** single-nucleus RNA sequencing, Parkinson’s disease, cell sparsity, GWAS, metacells, multiscale embedded gene coexpression network analysis, Bayesian network, key drivers

## Abstract

**Background:**

Multiple single-nucleus RNA sequencing (snRNA-seq) studies of the vulnerable brain regions of Parkinson’s disease (PD) have revealed alterations in brain cell populations and cell-type–specific transcriptomes. However, a systematic analysis of cell-type–resolved gene regulatory architecture in PD is lacking.

**Results:**

Here, we develop an integrative, metacell-based multiscale network analysis (MCMNA) of snRNA-seq data from the substantia nigra to systematically uncover molecular mechanisms and identify potential therapeutic targets for PD. MCMNA overcomes the inherent sparsity of single-cell data by leveraging metacell-based aggregation, enabling construction of robust gene regulatory networks. Gene coexpression network analysis identifies cell-type–specific gene modules associated with PD. Integration of metacell-based differential gene expression and a Bayesian causal network systematically reveals putative driver genes and their hierarchies.

**Conclusions:**

Our multiscale network models provide a framework for prioritizing candidate molecular regulators and pathways for further investigation of disease mechanisms and therapeutic targets.

## Introduction

Parkinson’s disease (PD) is the second most prevalent neurodegenerative disorder, with both motor and nonmotor symptoms [1, 2]. The pathology of PD is characterized by the progressive loss of dopaminergic (DA) neurons in the substantia nigra (SN) and accumulation of Lewy bodies (LBs), which contain abnormal α-synuclein protein aggregation [3, 4]. Rare variants in over 20 genes account for 3–5% of PD cases in most populations, most of which are highly penetrant and often known to be causal genes [5]. To date, genome-wide association studies (GWAS) have identified 90 genetic risk variants, which collectively account for 16–36% of the heritable risk of nonmonogenic PD [6]. Given that most patients with PD have no familial history or known PD mutations, there remains a critical gap in understanding how molecular signatures relate to disease mechanisms and therapeutic strategies in idiopathic PD. While the loss of DA neurons is a hallmark of the disease, microglial activation was first reported in PD brains in 1988 [7] and has been supported by *in vivo* and *in vitro* studies [8–11]. In addition, astrocyte reactivity has been prominently observed in the SN of patients with PD, with research highlighting both neuroprotective and neurotoxic roles such as production of oxidative and nitrosative stress, inflammation, and contribution to the blood–brain barrier [12–15]. Recently, studies have indicated oligodendrocyte dysfunction in PD, with changes pointing to altered inflammatory responses, protein folding stress, and myelination abnormalities [16–18].

The gene regulatory network (GRN) uncovers new pathways and gene targets in biological processes in human disease. Previously, our labs integrated a multiscale transcriptomic network analysis of PD SN samples by assembling the gene expression profiles from 8 published bulk RNA-seq studies to systematically identify intrinsic network structures and key regulators such as *STMN2* in PD [19]. Bulk tissue datasets do not have cell-type–level granularity, thus limiting the understanding of complex signaling across cell–cell populations and within a population. Recently, single-cell/single-nucleus RNA sequencing (sc/snRNA-seq) has emerged as a promising tool to unveil cell-level signaling pathways associated with neurodegenerative diseases including PD. We and others have profiled single nuclei of human SN from PD and healthy controls, identifying changes in gene expression and new biological pathways at single-cell levels [20–25]. While single-cell datasets continue to expand in public repositories, little progress has been made toward cell-type–specific GRN analysis for PD.

The challenges in constructing single-cell GRN inference mainly stem from the high sparsity in gene expression profiles (large percentage of missing values or zeros) in snRNA-seq data. Many model-based imputation methods have been developed, yet the performance of these methods varies across different datasets [26]. To address this challenge, recent studies have adopted the concept of “metacells” [27] and developed several metacell algorithms [27–30] to not only compress data but also enhance the signal-to-noise ratio in expression matrices. This new approach results in higher clustering accuracy, improved annotation, and increased reliability in the identification of differentially expressed genes (DEGs). Despite the potential, these metacell algorithms have yet to be applied to gene network analysis in PD [31].

In this study, we develop an integrative metacell-based gene coexpression and causal network analysis framework and apply it to a recently published PD snRNA-seq dataset in the human SN from 23 patients with PD and 9 healthy controls [20]. By adopting a systematic and data-driven approach in both coexpression and causal networks, we identify neuron- and glia-specific subnetworks that are dysregulated in PD. These cell-type– specific networks not only reveal putative key drivers for PD but also shed light on the molecular mechanisms of PD. Notably, the top putative drivers include known targets of PD such as *UCHL1, NSF*, and *PREPL* in neurons, as well as novel candidate genes, such as *CADPS2, APBB1IP*, and *HSP90AA1* in neurons or glial cells. In addition, PD driver genes (*STMN2* and *RALYL*) identified in our previously bulk RNA-seq network analysis are also key drivers in the respective neuronal single-cell networks. Altogether, our study provides a framework for integrating diverse, cell-type–specific networks to pinpoint putative drivers of disease networks.

## Results

### Integrative network biology framework for decoding molecular mechanisms of Parkinson’s disease at the single-cell level

To systematically model single-cell transcriptomic data in PD, we develop an integrative, metacell-based multiscale network analysis (MCMNA) framework that includes metacell construction and characterization, coexpression network analysis, causal network inference, and key driver analysis (Fig. 1). In brief, we first group single cells into metacells—a group of cells with highly similar gene expression profiles (Table 1). To preserve the cell-type annotation and phenotype information from snRNA-seq data, the metacell analysis is performed for each condition in each cell cluster. Single-cell profiles within each metacell are aggregated to create a metacell gene expression profile. Then, DEGs between the disease and control are identified, and functional enrichment analysis is performed on the DEGs to identify dysregulated pathways in PD (Fig. 1A). Next, we construct metacell-based coexpression analysis using multiscale embedded gene coexpression network analysis (MEGENA) [32] for each cell type to identify cell-type–specific coexpression modules (groups of highly coexpressed genes) (Fig. 1B). In parallel, we construct the global Bayesian probabilistic causal network (BN) [33, 34] and perform key driver analysis (KDA) [35] to predict potential key regulators, termed key drivers (KDs) of PD (Fig. 1C). A unique feature of MCMNA is that it addresses the sparsity of single-cell gene expression data and increases the robustness of the single-cell network by the generation of metacells, construction of a biologically meaningful correlation (BMC) matrix for the coexpression network, and additional pruning of the data matrix for causal network. Additionally, network models based on both PD and control allow us to capture early changes driving PD, as such signals may not change across various disease stages.

**Figure 1.**
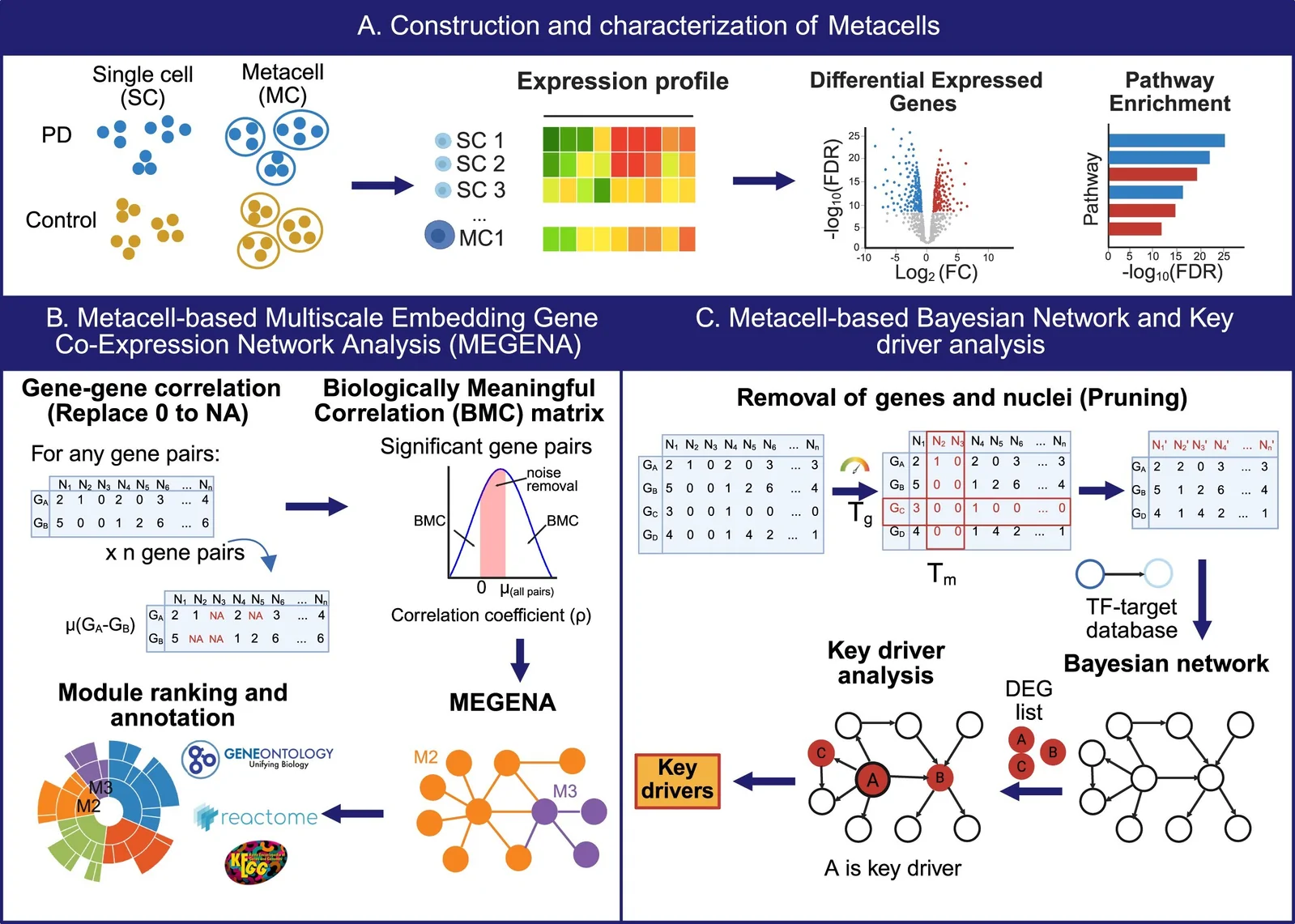
Integrative network biology framework for decoding molecular mechanisms of Parkinson’s disease at the single-cell level. (A) Construction and characterization of metacells. Metacells were generated using SuperCell by constructing a k-NN network. Cells were aggregated within each cell cluster and condition, followed by differential expression and pathway enrichment analyses. (B) Metacell-based multiscale embedding gene coexpression network analysis. Gene correlations were used to generate a biologically meaningful correlation (BMC) matrix, followed by MEGENA to identify multiscale coexpression modules and functional enrichment analysis. (C) Metacell-based Bayesian network and key driver analysis. Bayesian networks were constructed using RIMBANet with TF–target relationships from TFLink as regulatory priors. Key driver analysis was then performed to prioritize candidate regulators in the reconstructed causal networks. μ, mean gene expression within a metacell; BMC, biologically meaningful correlation; BN, Bayesian network; G, gene; KDA, key driver analysis; k-NN, k-nearest neighbors; MEGENA, multiscale embedded gene coexpression network analysis; N, network node; TF, transcription factor.

**Table 1.** Distribution of metacell sizes across cell clusters in the MSSM dataset.

Cluster	Metacell number		
	Control	PD	Total
C0 Oli	1,743	7,191	8,934
C1 Mic	438	1,545	1,983
C2 Ast	306	1,457	1,763
C3 Oli	441	1,433	1,874
C4 End	290	1,182	1,472
C5 OPC	204	1,157	1,361
C6 Neu	288	923	1,211
C7 Neu	419	848	1,267
C8 Per	130	520	650
C9 Neu	183	99	282

In the remainder of the article, we apply MCMNA to a snRNA-seq dataset derived from SN samples of 23 idiopathic PD cases and 9 controls, with an average age of 81 years, as described in a previous publication. [20]. The study profiled a total of 315,867 high-quality nuclei and identified 12 distinct cell clusters.

### Metacells uncover cell-type–specific gene expression enrichment between PD and control

At each cell cluster and condition (PD or control), single-cell profiles within each metacell are aggregated to generate metacell gene expression profiles, either by averaging the log-normalized single-cell expression data (average—“avg”) or by summing the raw single-cell values, followed by log normalization (sum). Additionally, the method allows for the inclusion of metacell size (the number of single cells in each metacell) as a weighting factor (weighted) or omitting it (unweighted). To explore global transcriptomic changes using the metacell method, we first examine the number of DEGs in each cell cluster (at least 1.1-fold change [FC]) and false discovery rate (adjusted *P* value less than 0.05) and calculate the percentage of preservation compared to the single-cell method. The average method demonstrates higher preservation compared to the sum method across most clusters (Fig. 2A, Supplementary Fig. S1A, B). While the number of DEGs is reduced in metacells compared to the single-cell method, the number of conserved markers increases, potentially reflecting enhanced cell cluster specificity and reduced noise in metacells (Supplementary Fig. S1C). However, this observation has not been formally tested, and further studies are needed to confirm it.

**Figure 2.**
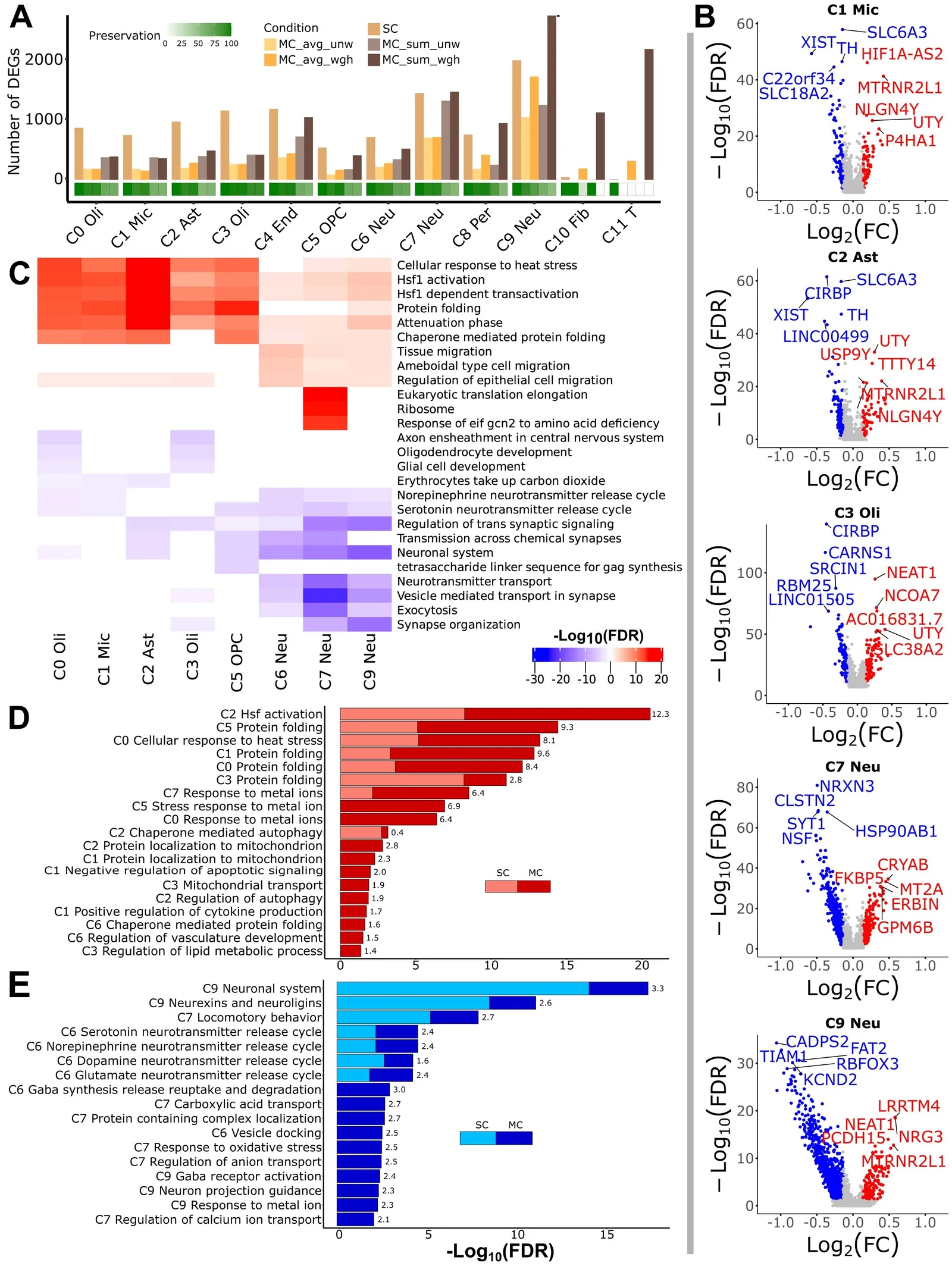
Generation and characterization of metacells. (A) Number of differentially expressed genes (DEGs) in single and metacells across different conditions in 12 cell clusters. (B) Volcano plot showing DEGs in selected major cell clusters: c1, Microglia; c2, Astrocyte; c3, Oligodendrocyte; c7, Neuron; and c9, Neuron. Top 5 upregulated and 5 downregulated DEGs (by −log10 [false discovery rate (FDR)]) of each cell cluster are labeled in red or blue, respectively. (C) Heatmap of top pathways enriched for up- and downregulated genes in major cell-type clusters. (D, E) Bar plot of gain of enriched pathways from upregulated genes (D) or downregulated DEGs (E) in major cell clusters. Color indicates the −log10 (FDR) value of the corresponding pathway from the single-cell (SC) and metacell (MC) methods. The number on the bar indicates the difference in the −log10 (FDR) value between MC and SC. Ast, astrocytes; Mic, microglia; Neu, neurons; Oli, oligodendrocytes; OPC, oligodendrocyte progenitor cells.

While weighted aggregation can incorporate cell-level weight, it substantially increases the data size and computation time, without consistently improving downstream analyses such as differential expression and pathway enrichment (Supplementary Fig. S1A–C, Supplementary Table S1). Therefore, the unweighted method provides a good balance between efficiency and accuracy. Distinct gene signatures of 5 major brain cell types (e.g., neurons, microglia, astrocytes, oligodendrocytes, and oligodendrocyte progenitor cells [OPCs]) are highlighted using the selected metacell method (average and unweighted) (Fig. 2B, Supplementary Fig. S1E, Table 2, Supplementary Table S3). Next, we compare our DEG signatures with other published snRNA-seq data of human SN from 3 independent cohorts in PD [21, 22, 25]. We reprocess the original datasets using our own analytical pipeline to identify cell clusters (Supplementary Tables S4–S10, Supplementary Fig. S1F–G, I–J, L–M). We then assess the overlap of DEG signatures in each cell type using Fisher’s exact test (FET). The results show that, for 3 datasets, there is a decrease in corrected *P* value but an increase in fold enrichment, indicating reduced statistical preservation strength but greater specificity in the preservation of DEG signatures across major cell cluster between the 2 datasets in metacells (Supplementary Tables S11–S13, Supplementary Fig. S1H, K, N).

**Table 2.** Number of differentially expressed genes (DEGs) identified by the metacell (MC) method per cell cluster.

Cluster	Number of DEGs identified by metacell (average, unweighted)		
	Upregulated DEGs	Downregulated DEGs	Total
C0 Oli	83	98	181
C1 Mic	77	105	182
C2 Ast	58	142	200
C3 Oli	124	137	261
C4 End	136	238	374
C5 OPC	49	39	88
C6 Neu	149	68	217
C7 Neu	194	514	708
C8 Per	67	114	181
C9 Neu	149	899	1,048
C10 Fib	0	1	1
C11 T	0	0	0

### Metacells reveal common and unique cell-type–specific biological pathways between PD and control

Functional enrichment analysis is performed on the DEG signatures from both metacell and single-cell methods. Using selected conditions (average and unweighted), we identify common and unique biological pathways. We observe that 10–30% and 0–60% shared pathways in major cell types from upregulated and downregulated DEGs, respectively (Supplementary Tables S14–S16, Supplementary Fig. S2A, B). Similar to previously reported results, upregulated genes across cell types are involved in heat stress, Hsf1 activation, protein folding, and the cell migration pathway. In contrast, downregulated genes are associated with nervous system development, synaptic transmission, neurotransmitter transport across cell types, and glial cell development in glial cells (Fig. 2C). Many pathways unique to the single-cell and metacell methods are also detected (Supplementary Tables S17–S18, Supplementary Fig. S2A, B). Positive correlation of both upregulated and downregulated pathways across cell types indicates highly conserved pathways between the 2 methods. Additionally, several pathways showing a significant difference in enrichment in the single-cell and metacell-based DEG signatures are highly relevant to PD biological pathways. These include protein folding in glial cells, autophagy in astrocytes, mitochondrial processes in microglia and astrocytes, cytokine production in microglia, vasculature development, neurotransmitter release, and calcium transport in neurons (Fig. 2D, E).

Additional metacell-specific DEGs showed biologically coherent and cell-type–associated functional enrichment. In glial populations, these genes were enriched for pathways related to metal ion response and ion homeostasis, driven in part by metallothionein genes (*MT1M, MT1G, MT1X*, and *MT1E*) (Supplementary Tables S19–S20). In neuronal populations, metacell-specific DEGs were predominantly enriched for pathways related to synaptic transmission. Comparison with DEG results from two independent datasets (Columbia dataset [25] and KAIST dataset [21]) further supported the biological relevance. Recurrently identified genes such as heat-shock and stress-response genes (*BAG3, DNAJA4, STIP1*, and *FKBP4*) are consistent with the enhanced detection of stress–response pathways in the metacell analysis in the glial population (Supplementary Table S21). Synaptic genes, including *GRIN2B* and *GAD1*, also overlapped with DEGs identified in the independent Columbia dataset [25], supporting the stronger enrichment of synaptic pathways detected using the metacell approach (Supplementary Table S21).

### A novel framework to address single-cell sparsity in the coexpression network

To understand the interactions among genes, we construct gene coexpression networks to identify gene modules using MEGENA under the MCMNA framework (Supplementary Fig. S3A). While metacells improve the gene–gene correlation, as noted in previous studies [27–30], gene–gene correlation still suffers from data sparsity. Therefore, zero counts are removed for each gene pair before calculating Spearman correlation coefficients (SCCs), leading to an increase in overall correlation distribution in metacells compared to single cells (Supplementary Fig. S3B). To address inherent noise in sc/snRNA-seq data observed by the drift in the mean signed SCCs across cell types, we propose a framework to create a biologically meaningful correlation (BMC) matrix at the optimal threshold of number of cells and genes (Supplementary Fig. S3A, C). We perform FET analysis between *STMN2*-related genes and *STMN2* perturbation DEG signatures in PD mouse SN [19] across all clusters and 3 neuronal clusters. Compared to the metacell false discovery rate (FDR) only and single-cell matrix only, the BMC approach yields higher −log10 (FDR) and fold enrichment in the MC-average comparison, as well as higher fold enrichment in the MC-sum comparison (Fig. 3A, Supplementary Table S22). These suggest that the BMC matrix indeed reduces noise in the weak positive correlation, resulting in a more consistent gene subset aligned with the STMN2 perturbation. Averaging log-normalized expression values indicates more statistically significant enrichment and is therefore used for downstream analysis (Fig. 3A, Supplementary Table S22). The optimized BMC matrix is then subjected to MEGENA. Additionally, this method enables construction of a gene coexpression network for the small neuron cluster 9 (c9), which was not possible due to high sparsity and small sample size. No MEGENA modules are found in the fibroblast cluster 10 and the T-cell cluster 11.

**Figure 3.**
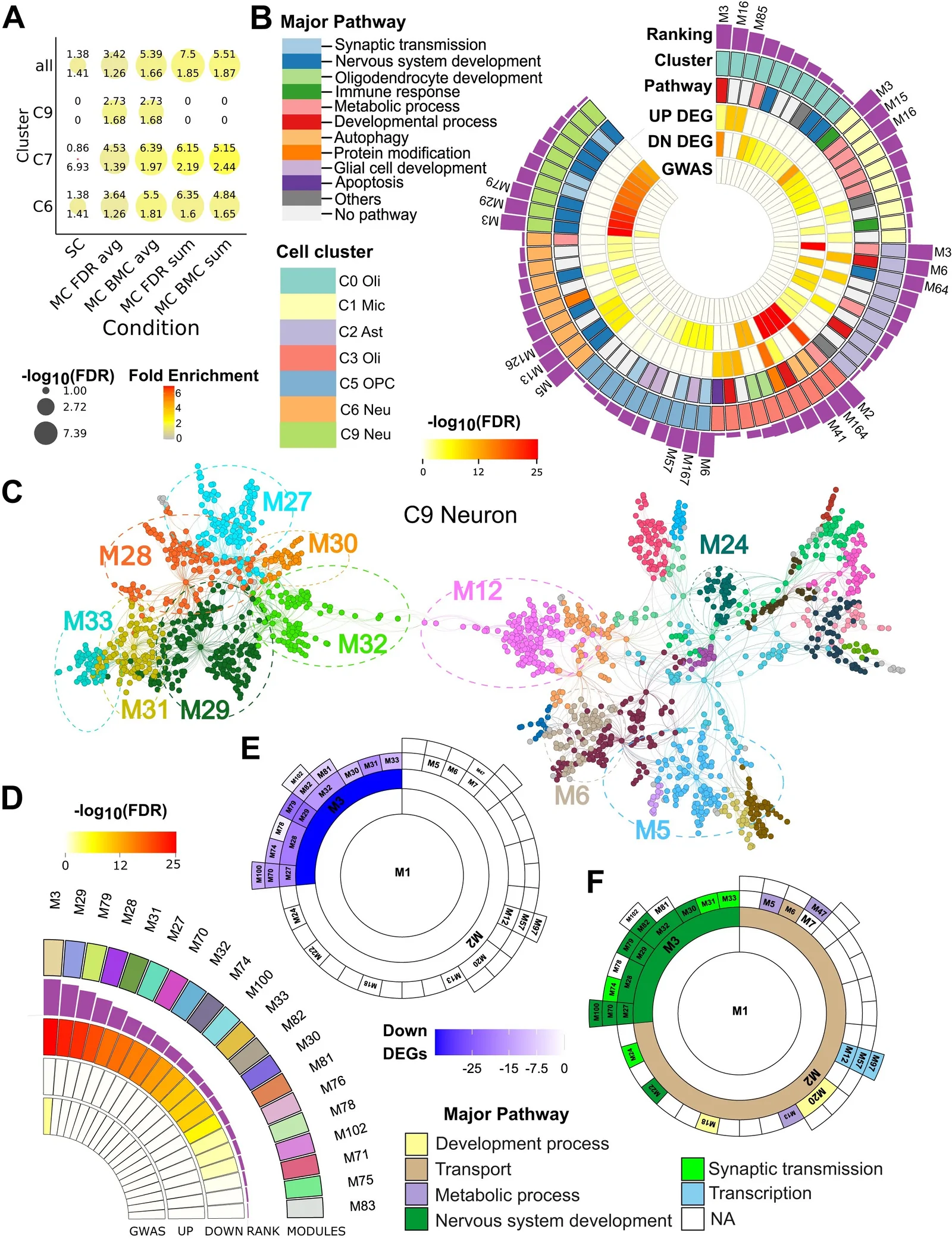
Metacell-based gene coexpression network of Parkinson’s disease. (A) Comparison of FET enrichment of STMN2-related genes and STMN2 perturbation DEGs. Top value: −log10 (FDR); bottom value: FE (C6, cluster 6; C7, cluster 7; C9, cluster 9; FDR, false discovery rate; MC, metacell; SC, single cell; “all” represents all cell clusters). (B) Top 10 modules enriched for the PD-related signatures within each major cell cluster (DN, downregulated; UP, upregulated). (C). A global MEGENA network in cluster 9 neurons. The module membership is represented by different colors. Seven top-ranked modules (M27, M28, M29, M30, M31, M32, M33) from parental module M3 and other modules enriched with other pathways (M5, M6, M12, M24) are highlighted. (D) The top 30 MEGENA modules most enriched for the PD-related gene signatures include the PD DEGs in each cell cluster and PD GWAS genes. (E) Enrichment of the DEGs of PD in MEGENA modules in cluster 9 neurons. Color intensity is proportional to −log10 (corrected FET *P* value). (F) Enrichment of the major pathways in MEGENA modules in cluster 9 neurons.

To assess potential bias from excluding zero-count observations, we correlate each gene’s average expression with its MEGENA connectivity (node degree) using Spearman’s correlation. Correlations are weak across all clusters (|ρ| < 0.2; Supplementary Fig. S3D), indicating that network connectivity is largely independent of baseline expression. Only cluster 2 (astrocyte) and cluster 7 (neuron) show moderate negative correlations (ρ = −0.2 to −0.3), suggesting that lower-expressed genes tend to have slightly higher connectivity. These results demonstrate that excluding zero-count observations does not preferentially favor highly expressed genes.

### Metacell-based coexpressed networks reveal cell-type–specific functional gene modules

In total, we identify 1,057 significant gene modules (FDR-adjusted *P* values less than 0.05) in all cell types and 833 modules in 5 major cell types (Table 3, Supplementary Table S23). To annotate biological functions associated with each module, functional enrichment analysis is performed. To prioritize gene modules associated with PD, a summary score is calculated based on the following FET enrichment tests: (i) enrichment for the DEG signatures between PD and control (upregulated [UP] and downregulated [DN] DEGs) and (ii) enrichment for the PD genetic risk variants (PD GWAS catalog) (Supplementary Table S24) [36]. Given that the central nervous system consists of various cell types, each with distinct roles in PD pathogenesis and progression, we focus on the major brain cell types: neurons (cluster 6 [c6], cluster 7 [c7], and cluster 9 [c9]), astrocytes (cluster 2 [c2]), microglia (cluster 1 [c1]), OPCs (cluster 5 [c5]), and oligodendrocytes (cluster 0 [c0] and cluster 3 [c3]). The findings of MEGENA modules reveal cell-type–specific functional modules characterized by highly coordinated molecular dysregulations in PD (Supplementary Tables S25–S27). The top 10 PD-associated gene modules within each major cell type are shown in Fig. 3B. Among the top 50 ranked modules, there are 17 neuronal modules, of which 14 originate from the neuron cluster c9. The number of top-ranked modules from other glial cells is as follows: oligodendrocytes (c0: 5, c3: 13), microglia (c1: 4), astrocytes (c2: 7), and OPC (c5: 4).

**Table 3.** Number of MEGENA modules, modules associated with PD DEGs/pathway annotation.

Cluster	Cell type	Number of modules	Number of signficant modules	Note	Number of modules with pathway annotation	Percentage of modules with pathway enrichment	Number of modules associated with upregulated DEGs	Number of modules associated with downregulated DEGs	Number of modules associated with GWAS genes
C0	Oligodendrocyte	104	70	Major cell type	33	47.1%	5	11	0
C1	Microglia	129	93	Major cell type	63	67.7%	8	12	0
C2	Astrocyte	113	91	Major cell type	42	46.2%	5	16	1
C3	Oligodendrocyte	265	204	Major cell type	97	47.5%	18	14	0
C4	Endothelia	109	75		55	73.3%	11	23	2
C5	Oligodendrocyte progenitor cell	145	102	Major cell type	43	42.2%	0	8	0
C6	Neuron	275	165	Major cell type	71	43.0%	11	8	1
C7	Neuron	63	53	Major cell type	27	50.9%	0	1	0
C8	Pericyte	238	149		94	63.1%	11	15	1
C9	Neuron	72	55	Major cell type	26	47.3%	0	17	1
	Total in all cell types		1,057						
	Total modules in major cell types		833						

We validate the biological coherence of our network modules in another snRNA-seq dataset from the SN of patients with PD and healthy participants (termed the Ma dataset) at the cell-type–specific level [25]. As shown in Supplementary Tables S28–S29, based on a global approach across all identified modules, module preservation is highest in oligodendrocytes (~30–50%), followed by microglia/astrocytes (~20–30%) and neurons (~5–20%) (Supplementary Fig. S4A). Additionally, we examine the conservation of the PD-related modules (i.e., modules enriched for upregulated, downregulated, and/or PD GWAS genes) in the 2 datasets. PD-related modules show higher and more significant preservation across all cell clusters (Supplementary Tables S30–S31). Similar to the global approach, module preservation is strongest in oligodendrocytes (35–90%), followed by astrocytes/microglia (~60–90%) and neurons (~0–30%). Notably, in our dataset, the PD-related modules in neuron cluster C9 show greater preservation in the corresponding neuron cluster C4 from the Ma dataset than the PD-related modules in neuron cluster C6 of our dataset show in C4 of the Ma dataset (Supplementary Fig. S4B).

We additionally performed a network-based module preservation analysis using weighted gene coexpression network analysis (WGCNA) [37]. This approach evaluates preservation of the modules from our dataset in the Ma dataset. Overall, the module preservation results are consistent with those from the FET analysis. Moderate-to-high module preservation is observed across major cell types, with the highest preservation in oligodendrocytes, followed by microglia and astrocytes, while module preservation is more variable across neuronal clusters. PD-associated modules generally show greater preservation than total global modules (Supplementary Tables S32–S33). These results are broadly consistent with those from the FET analysis and provide complementary evidence for the cross-cohort reproducibility of PD-associated gene modules.

### Neuronal gene modules are most dysregulated in PD

Among all the modules, the module M3 in the coexpression network from c9 is ranked as the top for its most significant enrichment of the downregulated DEGs using FET (multiple testing-corrected *P* or FDR = 1.7E-200, 2.3-fold enrichment [FE]) and GWAS genes (FDR = 8.1E-04, 2-FE) (Fig. 3B, Supplementary Table S25). The global network of c9 shows a highly hierarchical structure, suggesting strong modular organization and multiscale biological processes (Fig. 3C). Seven child modules of the module M3 in c9’s coexpression network are highly enriched for downregulated DEGs such as M27─*▸*M70─*▸*M100 (M27: FDR = 5.8E-18, 2.3 FE), M28─*▸*M74 (M28: FDR = 1.1E-20, 2.4 FE), M29─*▸*M79 (M29: FDR = 9.3E-24, 2.2 FE), M32─*▸*M82─*▸*M81 (M32: FDR = 2.2 E-16, 2.4 FE), M30 (FDR = 9.5 E-11, 2.8 FE), M31 (FDR = 5.8E-18, 2.5 FE), and M33 (FDR = 1.2 E-11, 2.7 FE) (Fig. 3E, Supplementary Table S26). M3 is associated with synapse organization (FDR *P* = 7.9E-24). All other child modules of M3 are also enriched for downregulated DEGs with synapse-related functions such as synapse assembly, regulation of neuron projection development, regulation of transsynaptic signaling, neuronal system, receptor localization to synapses, and dendrite development (Fig. 3F, Supplementary Table S25, Supplementary Table S27). These child modules of M3 are also among the top 50 modules from all cell clusters and among the top 20 modules from c9 (Fig. 3D, Supplementary Table S25). Interestingly, other branches of c9 separated from the M3 module are not enriched for the downregulated DEGs. These modules are associated with pathways such as ion transport, metabolic processes, and transcription, suggesting that only a small subset of c9 neurons appears to adopt the disease phenotype.

Apart from the neuron cluster c9, the modules in cluster c6 are less enriched for DEGs. In c6, a few top-ranked modules are associated with upregulated DEGs, such as M5 (FDR = 1.7E-08, 4.3 FE), M13 (FDR = 1.7E-08, 10 FE), M126 (FDR = 1.7E-08, 45 FE), and M8 (FDR = 3.73E-05, 4.6 FE), followed by modules associated with downregulated DEGs such as M92 (FDR = 1.7E-05, 16.9 FE) andM124 (FDR = 1.7, 36.1 FE) (Supplementary Fig. S5A, Supplementary Table S26). These are also linked to pathways involved in synapse organization and axon development (Figure S5B, Supplementary Table S25, Supplementary Table S27). Interestingly, no module in the network from the neuron cluster c7 is enriched for DEGs (Supplementary Table S25), though many modules have meaningful biological functions (Supplementary Fig. S5C). The global network of c7 also shows a more compact structure (Supplementary Fig. S5D). This implies that the dysregulated genes in c7 are not well coordinated.

### Glial gene modules associated with PD are involved in the development process, metabolic processes, and immune responses

In addition to neuronal modules, we also identify numerous glial modules in oligodendrocytes, microglia, astrocytes, and OPCs. Oligodendrocytes represent the largest population in the dataset, and consequently, the single-cell coexpression networks of cluster 0 (c0) and cluster 3 (c3) are among the largest, containing many genes and gene interactions. Among the 50 top-ranked modules overall of c3, M10─*▸*M41─*▸*M164 emerged as 1 branch of particular interest. M10 is a parent module of M41 and M42, with its members enriched with both upregulated (FDR = 2.9E-05, 4.3 FE) and downregulated DEGs (FDR = 5.9E-34, 10.3 FE) and involved in cell polarity (FDR = 0.005), mitochondria organization (FDR *P* = 0.05), and vesicle organization (FDR = 0.05) (Fig. 4A, B, Supplementary Fig. S6A). M10 has 2 branches, M41─*▸*M164 and M42─*▸*M166. One is the M41─*▸*M164 branch, in which both modules are enriched with downregulated DEGs (M41: FDR = 1.4E-43, 16 FE; M164: FDR = 1E-44, 17 FE), and their members are associated with autophagy of mitochondria (M41: FDR = 0.04; M164: FDR = 0.04) (Supplementary Fig. S6B). Another is M42─*▸*M166, with both modules enriched with upregulated DEGs (M42: FDR = 3.7E-11, 12 FE; M166: FDR = 1.7E-06, 11 FE) and associated with the apoptosis pathway (M42: FDR = 0.006; M166: FDR = 0.001) (Supplementary Fig. S6C). Some branches in the network of c3 are also associated with protein folding (M8─*▸*M38─*▸*M159) and oligodendrocyte differentiation (M5─*▸*M20─*▸*M111), highlighting the diverse pathways underlying oligodendrocyte functions. The network from the cell cluster c0 has a slightly smaller number of modules ranked among the top 50 modules from all cell clusters; the most significant module M3 is enriched for both upregulated (FDR = 2.2E-04, 2.1 FE) and downregulated DEGs (FDR = 1E-15, 2.3 FE), and it is associated with actin filament organization (FDR = 1.5E-04). Several child modules of M3 from c0 are also enriched for DEGs, including M6─*▸*M67 (involved in nervous system development), M8 (involved in metabolic processes), and M17 (involved in nervous system development) (Supplementary Fig. S6E, F).

**Figure 4.**
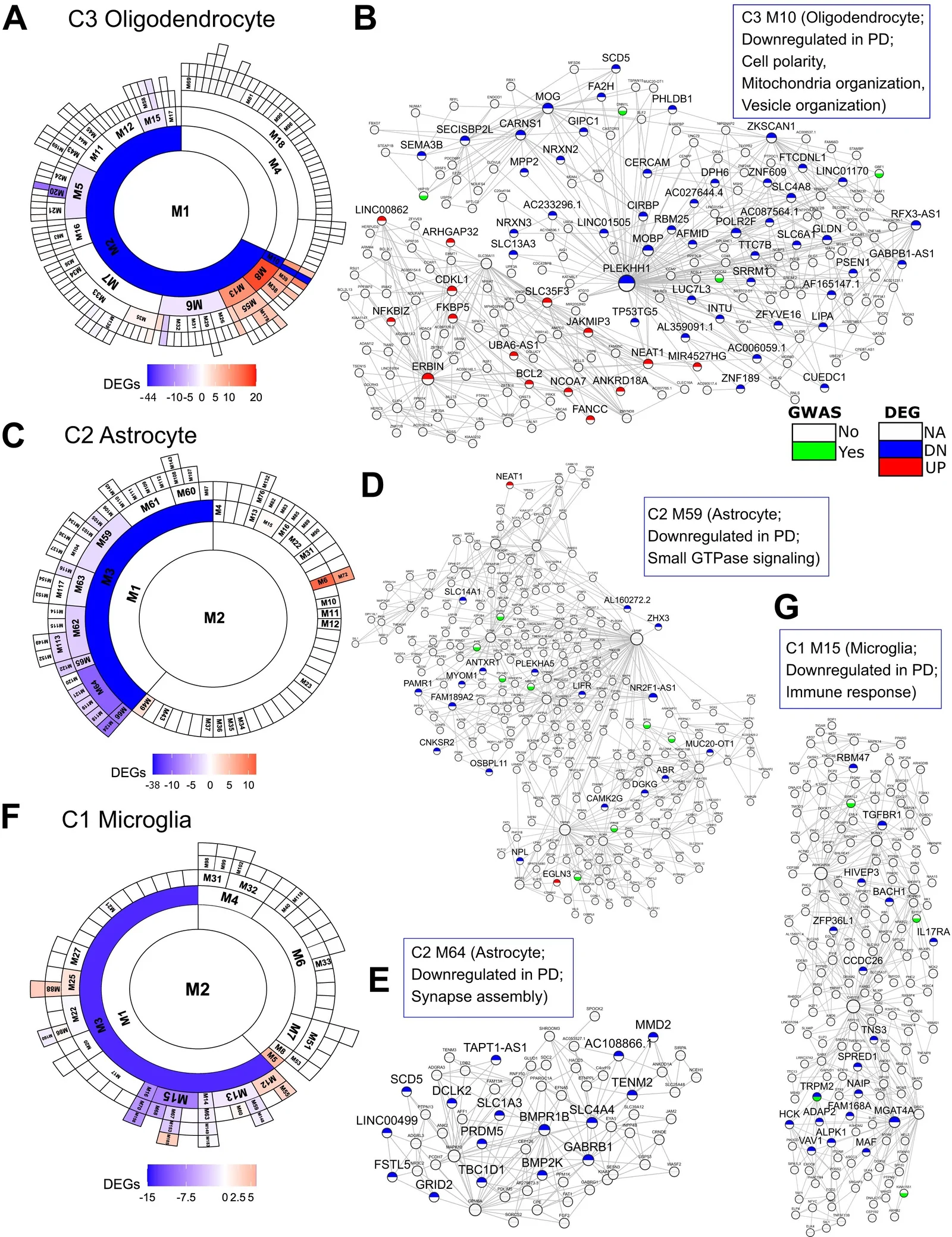
Metacell-based gene coexpression of several glial cell clusters. (A, C, F) Enrichment of the DEGs of PD in MEGENA modules in cluster 3 oligodendrocytes (A), cluster 2 astrocytes (C), or cluster 1 microglia (F). Color intensity is proportional to −log10 (corrected FET *P* value). (B, D, E, G). The top-ranked module M10 of cluster 3 oligodendrocytes (B), M59 of cluster 2 astrocytes (D), M64 of cluster 2 astrocytes (E), or M15 of cluster 1 microglia (G). The pie chart of each node indicates whether it is a DEG or GWAS gene. The size of a node is proportional to the node connectivity in the MEGENA network. DN, downregulated; UP, upregulated.

We also identify several significant modules in the astrocyte cluster c2. Among those, the most significant module M3 is enriched for the downregulated DEGs (FDR = 2.1E-38, 3.6 FE) and the GWAS genes (FDR = 4E-04, 1.9 FE) (Fig. 4C, Supplementary Fig. S7A). Some of M3’s child modules are functionally important and enriched for downregulated genes. The M3─*▸*M59 branch is highly associated with regulation of small GTPase mediate signaling transduction (M3: FDR = 2.1E-10; M59: FDR = 0.004) (Fig. 4D), while the M3─*▸*M64 branch is associated with synapse assembly (M64: FDR = 5.1E-03) (Fig. 4E). On the other hand, the modules M6 and M72 are highly enriched for upregulated DEGs (M6: FDR = 5.8E-14, 21.3 FE; M72: FDR = 2.35E-08, 18 FE) (Supplementary Fig. S7B) and linked to inclusion body assembly (M6: FDR = 1.7E-04) and regulation of endothelial cell chemotaxis (FDR = 2.5E-03) (Supplementary Fig. S7C). These findings demonstrate the role of astrocytes in modulating and supporting neuronal and synaptic functions.

Furthermore, we identify several microglial modules highly associated with PD. The top microglial module M15, which is associated with mononuclear cell differentiation, is enriched for downregulated genes (FDR = 4.8E-09, 6.6 FE) (Fig. 4F, G, Supplementary Fig. S7C). Additionally, we also observe a few branches associated with GTPase activity. While the branch of M16─*▸*M70─*▸*M156 is associated with downregulated DEGs (M16: FDR = 3.9E-08, 6.1 FE; M70: FDR = 1.7E-07, 6.3 FE; M156: FDR = 9.7E-08, 6.7 FE) (Supplementary Fig. S7D), the branch of M12─*▸*M55 is associated with upregulated DEGs (M12: FDR = 3.6E-04, 8.7 FE; M55: FDR = 5.4–04, 13.8 FE) (Supplementary Fig. S7E). The presence of coexpressed genes with roles in innate immune signaling and GTPase activity might suggest the role of certain gene modules in translating or facilitating myonuclear cells to microglia into more reactive states.

Last but not least, although the modules in the network of OPCs are less enriched for DEGs compared to other cell types, several modules are enriched for downregulated DEGs (Supplementary Fig. S7F) and associated with glial cell development, nervous system development, and synaptic transmission (Supplementary Fig. S7G). Interestingly, these downregulated modules are the daughter modules of M2 (FDR = 0.01, 1.3 FE) with diverse functions, including the branch M6─*▸*M57 (M6: FDR = 2.9E-07, 6.4 FE; M57–cFET = 5.3E-07, 7.3 FE) involved in regulation of transsynaptic signaling (M6: FDR = 6.1E-03, M57: FDR = 1.4E-03) and the branch M8─*▸*M67 (M8: FDR = 5.3E-03, 5.3 FE; M67: FDR = 3.8E-04, 8.1 FE) involved in glial cell differentiation (M8: FDR = 1.8E-04; M67: FDR = 1.4E-06). These findings suggest that certain components of OPCs support both neuronal and glial functions, which have been understudied in the field.

### Metacell-based Bayesian networks predict well-known and novel key drivers

While the gene coexpression network analysis identifies coherent gene modules associated with PD in each major brain cell type, the findings remain inherently associative and do not establish causal relationships among genes. To move beyond correlation and uncover potential regulatory mechanisms, we construct Bayesian probabilistic causal networks (BNs) to identify gene–gene causal relationships. Given the sparsity existing in the datasets, we further remove additional genes and metacells in a process of pruning to find the optimal cutoff (Fig. 1C). To evaluate whether expression-based pruning preferentially excluded transcriptional regulators, we examined transcription factors (TFs) before and after applying the Tg = 0.4 threshold. Across all major cell types, around 38–77% of TFs were removed, compared with 54–74% of genes overall. Thus, TFs were not preferentially depleted by the filtering procedure (Supplementary Table S34).

After that, we construct a global BN for each cell cluster, followed by KDA, to predict key regulators for each cell cluster [19, 38] (Fig. 5A, B, Supplementary Fig. S8A, Fig. 6A, B, Supplementary Fig. S9A, C, Supplementary Fig. S9E). We identify a total of 151 key drivers (138 unique genes), and interestingly, several drivers are detected in multiple cell clusters (Table 4, Supplementary Table S35). We also estimate the relevance of each gene to PD as the co-occurrence of a given gene and PD in PubMed. Among 138 unique key drivers, 43 (~30%) have occurrence above or equal to 5 (Fig. 5C). Then, we classify the key drivers into 2 categories: (i) well-studied key drivers (co-occurrence ≥5) and (ii) novel key drivers (co-occurrence <5) (Table 4, Supplementary Table S36).

**Figure 5.**
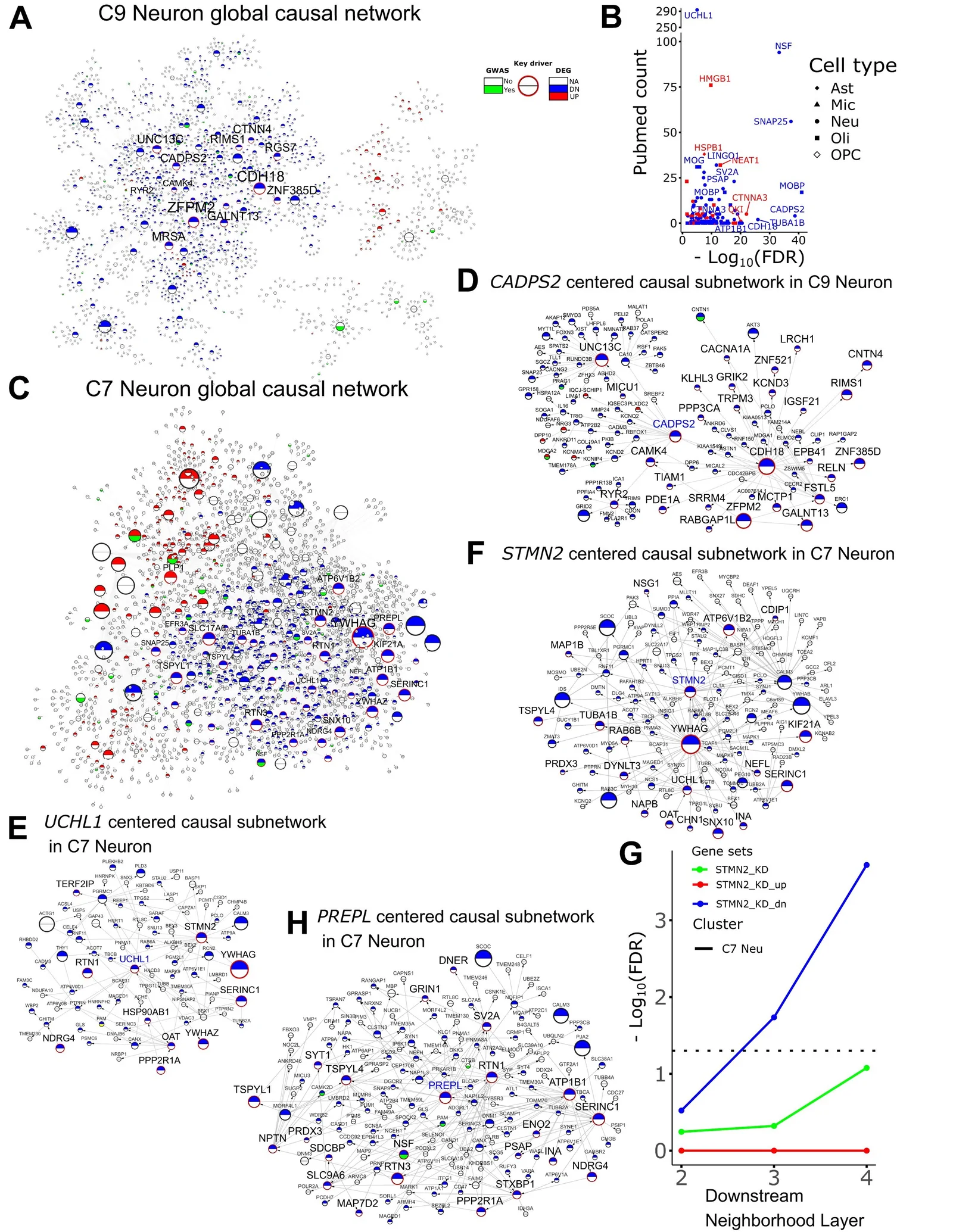
Bayesian probabilistic causal network (BN) predicts novel key drivers in neuronal cells. (A) BN in cluster 9 neurons. (B) BN in cluster 7 neurons. (C) Key driver identification in major cell types. (D–F, H) The 2-layer network neighborhoods of *CADPS2* in cluster 9 neurons, *UCHL1* in cluster 7 neurons, *STMN2* in cluster 7 neurons, and *PREPL* in cluster 7 neurons, respectively. The size of a node is proportional to the node connectivity in the BN. The directionality of the arrow indicates the upstream–downstream effect. Nodes with burgundy labels are key drivers in that network. (G). FET enrichment for the overlap in vivo perturbation signature of STMN2 knockdown in mice and each n-layer neighborhood regulated by the STMN2 driver gene in cluster 7 neuron BN.

**Figure 6.**
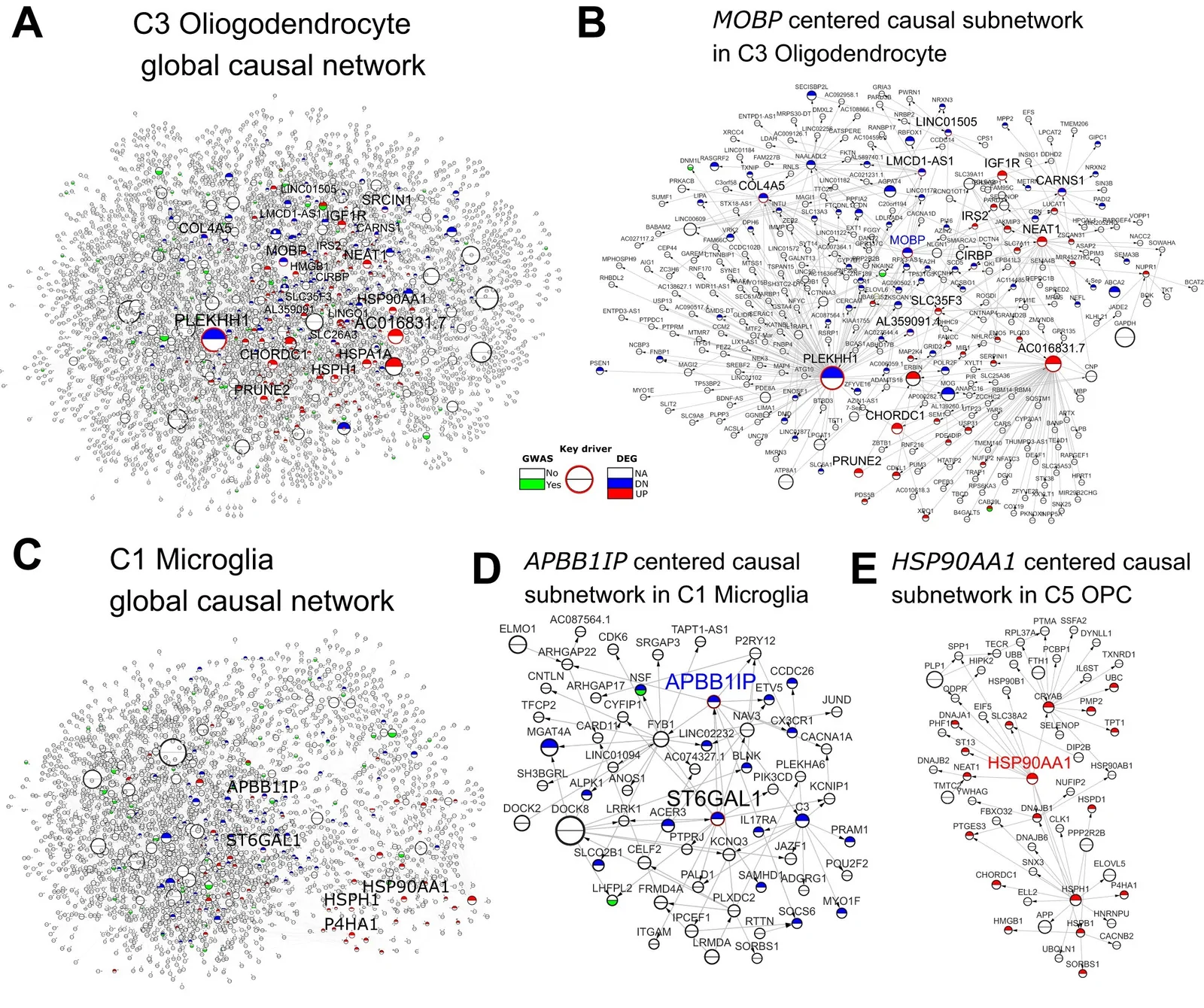
Bayesian probabilistic causal network (BN) predicts novel key drivers in glial cells. (A, C) BN in cluster 3 neurons (A), cluster 1 microglia (C). (B, D, E) The 2-layer network neighborhoods of *MOBP* in cluster 3 oligodendrocytes, *APBB1IP* in cluster 1 microglia, and *HSP90AA1* in cluster 5 oligodendrocyte progenitor cells. The size of a node is proportional to the node connectivity in the BN. The directionality of the arrow indicates the upstream–downstream effect. Nodes with burgundy labels are key drivers in that network.

**Table 4.** Summary of key driver genes.

Gene	FDR network	Clusterid	Cell_type	DEGs
MOBP	9.21226E-42	Cluster 3	Oli	DOWN
CADPS2	1.89876E-39	Cluster 9	Neu	DOWN
SNAP25	4.58012E-38	Cluster 7	Neu	DOWN
NSF	5.18355E-34	Cluster 7	Neu	DOWN
TUBA1B	1.09898E-26	Cluster 7	Neu	DOWN
CTNNA3	9.14221E-23	Cluster 6	Neu	UP
CDH18	2.90425E-21	Cluster 9	Neu	DOWN
QKI	8.26895E-21	Cluster 6	Neu	UP
ATP1B1	1.89952E-20	Cluster 7	Neu	DOWN
LMCD1-AS1	2.2634E-20	Cluster 0	Oli	DOWN
YWHAG	1.05962E-19	Cluster 7	Neu	DOWN
AC016831.7	5.93343E-19	Cluster 3	Oli	UP
STXBP1	8.40587E-19	Cluster 7	Neu	DOWN
ATP6V1B2	1.45309E-18	Cluster 7	Neu	DOWN
PREPL	1.68954E-18	Cluster 7	Neu	DOWN
ST18	3.43037E-18	Cluster 6	Neu	UP
PPP2R2B	3.08793E-17	Cluster 0	Oli	DOWN
RIMS1	1.05017E-16	Cluster 9	Neu	DOWN
CIRBP	2.83015E-16	Cluster 3	Oli	DOWN
AGPAT4	3.16844E-16	Cluster 0	Oli	DOWN
ZNF385D	5.02798E-16	Cluster 9	Neu	DOWN
PPP2R1A	2.02198E-15	Cluster 7	Neu	DOWN
TAGLN3	2.43878E-15	Cluster 7	Neu	DOWN
MOBP	2.66953E-15	Cluster 0	Oli	DOWN
NCKAP5	9.53044E-15	Cluster 6	Neu	UP
INA	1.04713E-14	Cluster 7	Neu	DOWN
GRIK2	1.3459E-14	Cluster 9	Neu	DOWN
LINC01505	2.38875E-14	Cluster 3	Oli	DOWN
NAALADL2	4.05882E-14	Cluster 0	Oli	DOWN
RNF220	4.18166E-14	Cluster 6	Neu	UP
NDRG4	5.66485E-14	Cluster 7	Neu	DOWN
NEAT1	9.28666E-14	Cluster 3	Oli	UP
HSP90AB1	1.6997E-13	Cluster 7	Neu	DOWN
RTN3	2.31924E-13	Cluster 7	Neu	DOWN
SV2B	2.37708E-13	Cluster 7	Neu	DOWN
SYT1	2.65429E-13	Cluster 7	Neu	DOWN
SLC9A6	6.72434E-13	Cluster 7	Neu	DOWN
PRDX3	7.84196E-13	Cluster 7	Neu	DOWN
NEFL	1.45787E-12	Cluster 7	Neu	DOWN
TRPM3	1.61898E-12	Cluster 9	Neu	DOWN
SV2A	2.0525E-12	Cluster 7	Neu	DOWN
DYNLT3	2.3045E-12	Cluster 7	Neu	DOWN
CHORDC1	2.41788E-12	Cluster 3	Oli	UP
ZFPM2	4.59149E-12	Cluster 9	Neu	DOWN
WDR49	7.65653E-12	Cluster 2	Ast	DOWN
SLC35F3	1.1931E-11	Cluster 3	Oli	UP
IRS2	1.61137E-11	Cluster 3	Oli	UP
NAPB	1.69433E-11	Cluster 7	Neu	DOWN
FSTL5	2.02846E-11	Cluster 9	Neu	DOWN
NRXN3	2.9778E-11	Cluster 0	Oli	DOWN
HSP90AA1	3.60675E-11	Cluster 5	OPC	UP
TSPYL4	4.48731E-11	Cluster 7	Neu	DOWN
HSPH1	5.04294E-11	Cluster 3	Oli	UP
RELN	9.0848E-11	Cluster 9	Neu	DOWN
CARNS1	1.06311E-10	Cluster 0	Oli	DOWN
PSD3	1.50424E-10	Cluster 0	Oli	DOWN
HMGB1	1.65668E-10	Cluster 3	Oli	UP
CHN1	2.51511E-10	Cluster 7	Neu	DOWN
PLEKHH1	2.58888E-10	Cluster 3	Oli	DOWN
CDIP1	2.93404E-10	Cluster 7	Neu	DOWN
OAT	2.93404E-10	Cluster 7	Neu	DOWN
GLDN	3.42564E-10	Cluster 0	Oli	DOWN
FGF14	4.50302E-10	Cluster 9	Neu	DOWN
NSG1	4.64979E-10	Cluster 7	Neu	DOWN
APBB1IP	8.54351E-10	Cluster 1	Mic	DOWN
KIF3A	1.88889E-09	Cluster 7	Neu	DOWN
ZFYVE16	1.98436E-09	Cluster 0	Oli	DOWN
HSPA1B	2.12676E-09	Cluster 2	Ast	UP
RALYL	2.13818E-09	Cluster 9	Neu	DOWN
CARNS1	2.87771E-09	Cluster 3	Oli	DOWN
TIAM1	5.14323E-09	Cluster 9	Neu	DOWN
SNX10	5.88232E-09	Cluster 7	Neu	DOWN
PPP3CA	6.22547E-09	Cluster 9	Neu	DOWN
STMN2	8.12876E-09	Cluster 7	Neu	DOWN
NPAS3	1.02625E-08	Cluster 6	Neu	UP
PLEKHH1	1.068E-08	Cluster 0	Oli	DOWN
TSPYL1	1.26511E-08	Cluster 7	Neu	DOWN
KLHL3	1.49504E-08	Cluster 9	Neu	DOWN
IGSF21	1.49504E-08	Cluster 9	Neu	DOWN
SRRM4	1.49504E-08	Cluster 9	Neu	DOWN
EPB41	1.49504E-08	Cluster 9	Neu	DOWN
CTNNA3	1.51411E-08	Cluster 0	Oli	DOWN
CACNA1A	1.77074E-08	Cluster 9	Neu	DOWN
AL359091.1	1.89004E-08	Cluster 3	Oli	DOWN
RAB6B	1.99932E-08	Cluster 7	Neu	DOWN
MDGA2	2.06594E-08	Cluster 6	Neu	UP
KIF21A	2.49608E-08	Cluster 7	Neu	DOWN
KCND3	2.83236E-08	Cluster 9	Neu	DOWN
HSPB1	3.04917E-08	Cluster 2	Ast	UP
SLC26A3	3.76762E-08	Cluster 3	Oli	DOWN
CAMK4	3.77013E-08	Cluster 9	Neu	DOWN
PSAP	3.79245E-08	Cluster 7	Neu	DOWN
SLC17A6	5.42435E-08	Cluster 7	Neu	DOWN
ENO2	9.78856E-08	Cluster 7	Neu	DOWN
NPTN	9.93365E-08	Cluster 7	Neu	DOWN
HSP90AA1	1.18516E-07	Cluster 3	Oli	UP
EFR3A	1.76577E-07	Cluster 7	Neu	DOWN
RABGAP1L	2.56737E-07	Cluster 9	Neu	DOWN
MSRA	3.27897E-07	Cluster 9	Neu	DOWN
DNER	4.71134E-07	Cluster 7	Neu	DOWN
HSPA1A	8.96263E-07	Cluster 3	Oli	UP
HSPH1	1.12105E-06	Cluster 1	Mic	UP
LRCH1	1.23183E-06	Cluster 9	Neu	DOWN
LINGO1	1.37409E-06	Cluster 3	Oli	DOWN
MAP7D2	1.74094E-06	Cluster 7	Neu	DOWN
UNC13C	2.1177E-06	Cluster 9	Neu	DOWN
PDE1A	2.12292E-06	Cluster 9	Neu	DOWN
ZNF521	2.48268E-06	Cluster 9	Neu	DOWN
COL4A5	2.87927E-06	Cluster 3	Oli	DOWN
HSP90AA1	3.22132E-06	Cluster 1	Mic	UP
GALNT13	4.18235E-06	Cluster 9	Neu	DOWN
PIP4K2A	4.20398E-06	Cluster 6	Neu	UP
SLC14A1	4.29232E-06	Cluster 2	Ast	DOWN
CLTC	4.33542E-06	Cluster 7	Neu	DOWN
TERF2IP	4.46601E-06	Cluster 7	Neu	DOWN
NTRK3	5.24225E-06	Cluster 9	Neu	DOWN
RYR2	5.93555E-06	Cluster 9	Neu	DOWN
RGS7	6.96662E-06	Cluster 9	Neu	DOWN
MAP1B	7.90668E-06	Cluster 7	Neu	DOWN
UCHL1	8.01583E-06	Cluster 7	Neu	DOWN
SERINC1	9.53234E-06	Cluster 7	Neu	DOWN
MOG	1.03566E-05	Cluster 0	Oli	DOWN
NCDN	1.51592E-05	Cluster 7	Neu	DOWN
HSP90AA1	1.73943E-05	Cluster 2	Ast	UP
GABRB1	1.99361E-05	Cluster 2	Ast	DOWN
MCTP1	2.68032E-05	Cluster 9	Neu	DOWN
YWHAZ	3.29825E-05	Cluster 7	Neu	DOWN
P4HA1	4.2445E-05	Cluster 1	Mic	UP
PATJ	8.40131E-05	Cluster 9	Neu	DOWN
LMCD1-AS1	0.00011215	Cluster 3	Oli	DOWN
LMCD1-AS1	0.00011608	Cluster 2	Ast	DOWN
GRIN1	0.000120242	Cluster 7	Neu	DOWN
ST6GAL1	0.000138384	Cluster 1	Mic	DOWN
MICU1	0.00021693	Cluster 9	Neu	DOWN
PRUNE2	0.000236518	Cluster 3	Oli	UP
QDPR	0.000269033	Cluster 2	Ast	UP
PLP1	0.000274736	Cluster 7	Neu	UP
CPLX1	0.000316944	Cluster 7	Neu	DOWN
NEFM	0.000363602	Cluster 7	Neu	DOWN
RTN1	0.000882048	Cluster 7	Neu	DOWN
ETV1	0.000926521	Cluster 9	Neu	DOWN
CALN1	0.001081454	Cluster 9	Neu	DOWN
PPP3CA	0.001346304	Cluster 7	Neu	DOWN
HSPH1	0.001455366	Cluster 2	Ast	UP
SDCBP	0.003187749	Cluster 7	Neu	DOWN
LINC00609	0.003807398	Cluster 0	Oli	DOWN
KIAA1217	0.009682925	Cluster 6	Neu	UP
CNTN4	0.013895365	Cluster 9	Neu	DOWN
SRCIN1	0.022011465	Cluster 3	Oli	DOWN
HSP90AA1	0.025656444	Cluster 0	Oli	UP
IGF1R	0.033855889	Cluster 3	Oli	UP

### Novel and known key drivers of neuronal clusters converge on synaptic regulation

The key drivers of neuronal clusters c7 and c9 are among the most significant drivers across all cell clusters. The most significant driver is *CADPS2* in c9 (KDA FDR = 1.8E-39; 1.8 FE), which is the most downregulated in c9 (FC = 0.48, FDR = 5.2E-35). In neurons, *CADPS2* encodes a calcium-binding protein that regulates synaptic exocytosis and dense core vesicles [39, 40] and has been previously studied in dopamine release [41]. Also, *CADPS2* is a novel small neuronal cell marker in idiopathic PD [42]. *CADPS2* is also a member of the gene modules, including M3, M32, and M81, which are associated with synapse organization and neural system pathways (Supplementary Table S23, Supplementary Table S23). The BN in c9 also indicates *CADPS2* as an upstream driver of 4 downregulated key drivers such as *CDH18, RYR2, UNC13C*, and *FSTL5*, 3 of which (*CDH18, RYR2*, and *UNC13C*) play roles in calcium-dependent synaptic cell–cell adhesion [43, 44], release of calcium ions [45], and exocytosis [46], respectively (Fig. 5D, Supplementary Table S36). Interestingly, the 2-layer subnetwork centered on *CAPDS2* (111 genes) is also involved in the modulation of chemical synaptic transmission (FDR = 2E-04), regulation of transsynaptic signaling (FDR = 2.1E-03), and synaptic vesicle docking (FDR = 3.2E-03) (Supplementary Table S37). These genes form a coordinated subnetwork to modulate a calcium-mediated process, which is essential for neuronal communication and synaptic function.

*RALYL*, previously identified as a downregulated key regulator from the network analysis of bulk transcriptomic data in PD [19], also emerges as a key driver in c9 (KDA: FDR = 2.1E-09; 2.2 FE; expression: 0.6 FC, FDR = 9.8E-23) (Supplementary Fig. S8B, Supplementary Table S36). *RALYL* is a member of M3, M29, and M74 (Supplementary Table S23). The subnetwork around *RALYL* is associated with metabolic pathways such as cell surface receptor protein tyrosine kinase activity (FDR *P* = 3.7E-03), cellular response to histamine (FDR = 1E-02), and hypoxia (FDR = 1E-02) (Supplementary Table S37). Thirty of the 36 drivers in c9, which are downregulated in PD, are novel as only 6 genes have been associated with PD or studied in PD (Table 4, Supplementary Table S35).

In contrast to c9, 24 of the 54 drivers in c7 have well-known functions associated with PD (24/54 genes). For example, a major driver, *UCHL1* (ubiquitin C-terminal hydroxylase), is implicated in the early onset of family and sporadic PD [5], influencing α-synuclein degradation and contributing to PD susceptibility [47]. In cluster c7, *UCHL1* is significantly downregulated and enriched in cluster 7 (KDA: FDR = 8E-06, FE = 2; expression: FC = 0.8, FDR = 5.4E-22). BN results indicate *UCHL1* is the downstream effector of 2 key drivers, *STMN2* (previously identified as a key driver in bulk transcriptomic data) and *OAT* (Fig. 5E, Supplementary Table S36). The subnetwork around *UCHL1* is involved in regulation of high-voltage-gated calcium channel activity (FDR = 6.3E-02), insulin secretion (FDR = 6.3E-02), and membrane invagination (FDR *P* = 6.3E-02) (Supplementary Table S37).

Besides *UCHL1*, we also identify another GWAS-associated gene, *NSF*, as a downregulated key driver in c7 (KDA: FDR = 5.2E-34, FE = 1.9; expression: FC = 0.7, FDR = 1.8E-56). *NSF* encodes N-ethylmaleimide-sensitive fusion protein, which plays an essential role in intracellular vesicle transport, synaptic transmission, and membrane fusion [48], interacting with α-synuclein via the SNARE complex protein [49], with a diminishing expression level in an MPTP mouse model of PD [50] and lower blood and brain levels in PD [51]. Additionally, *NSF* also directly regulates other key drivers such as *ATP1B1, SV2B*, and *STXBP1*, which play a role in regulating the ATPase Na^+^/K^+^ pump [52], vesicle trafficking and exocytosis [53], and neurotransmitter release [54], respectively (Supplementary Fig. S8F, Supplementary Table S36). The subnetwork centered on *NSF* is also involved in protein localization to the plasma membrane (FDR = 9.4E-06), neuron projection development (FDR = 1.6E-04), and synaptic vesicle membrane organization (FDR = 5.02E-03) (Supplementary Table S37), highlighting the functional context of the PD GWAS gene.

*STMN2*, previously identified as a regulator [19], is also a key driver in c7 (FDR = 8E-09, 1.9 FE) and downregulated (FC = 0.8, FDR = 7.5E-15) (Fig. 5F, Supplementary Table S36). *STMN2 i*s known to regulate microtubule dynamics, axon formation, and neurite outgrowth during development [55–57]. The subnetwork around *STMN2* also indicates the enrichment of pathways such as regulation of microtubule depolymerization (FDR = 3.4E-02), regulation of macroautophagy (FDR = 3.9E-02), and polymeric cytoskeletal fiber (FDR *P* = 9.5E-02) (Supplementary Table S37). The subnetworks around *STMN2* are enriched for the knockdown signature of *STMN2* in mouse SN [19], validating the accuracy of our predicted metacell-based causal network (Fig. 5G).

Additionally, we also identify *PREPL* as a key driver (KDA: FDR = 1.7E-18, FE = 2.2; expression: FC = 0.8, FDR = 3.0E-23). *PREPL* encodes a propyl endopeptidase-like enzyme and has recently been implicated in neuronal functions, including protein trafficking, endocytosis, synaptic activity [58, 59], and the facilitation of α-synuclein aggregation seeding [60], highlighting its relevance as a candidate for further investigation. The subnetwork around *PREPL* (161 genes) suggests *PREPL* as downstream of *ATP1B1* and upstream of many other key drivers such as *STXBP1, SLC9A6, RTN1, NPTN, TSPYL4, SV2A*, and *SERINC1* (Fig. 5H, Supplementary Table S36). This network is involved in positive regulation of synaptic transmission (FDR = 2.6E-03), negative regulation of amyloid beta formation (FDR = 2.6E-03), and intracellular monoatomic cation homeostasis (FDR = 2.6E-03) (Supplementary Table S37).

In cluster c6, we identify the PD GWAS gene, *MDGA2*, as an upregulated key driver (KDA: FDR = 2.1E-08, FE = 3.3; expression: FC = 1.2, FDR = 2.1E-05). *MDGA2*, which encodes a regulator of axonal growth [61], contains a single-nucleotide polymorphism that has been potentially linked to earlier onset of familial PD [62]. The subnetwork around *MDGA2* (68 genes), which is involved in chemical synaptic transmission (FDR = 2.7E-02) and synapse organization (FDR *P* = 3.5E-02), shows that *MDGA2* directly regulates another key driver, *NPAS3* (Supplementary Fig. S6E, Supplementary Tables S36–S37).

### Emerging key drivers in glial cells suggest novel causal roles in homeostasis, metabolic responses, immunity, and heat shock response

In oligodendrocytes, *MOBP* (myelin-associated oligodendrocyte basic protein) is identified as a key driver. *MOBP* plays a role in myelination and is a structural constituent of the myelin sheath [63, 64]. This gene is the top driver with the highest network significance and is downregulated in c3 (KDA: FDR *P* = 9.2E-42, 5.5 FE; 0.8 FC, FDR = 3E-59) and c0 (KDA: FDR = 2.7E-15, 6.05 FE; expression: 0.9 FC, FDR *P* = 6.3E-100). The subnetworks around *MOBP* in c3 and c0 indicate *MOBP* as an upstream regulator of key drivers such as *NEAT1, COL4A5, CARNS1*, and *LINC01505* in c3 (Fig. 6B) and *CARNS1* in c0 (Supplementary Fig. S9B). In c3, the subnetwork around *MOBP* (307 genes) is associated with regulation of dendritic spine development (*P* = 2.7E-04), myelination (*P* = 9.9E-04), neuronal cell–cell adhesion (*P* = 1.1E-03), and protein localization to synapse (*P* = 2.1E-03), while in c0, the subnetwork (61 genes) is associated with transition metal ion transport (*P* = 1.1E-04), substantia nigra development (*P* = 3.3E-04), and regulation of neuron apoptotic process (*P* = 6.5E-04) in c0 (Supplementary Table S36). Although *MOBP*’s role is not novel, the specific pathway coenrichment identified in our study highlights its dual function in both structural maintenance and cellular response. In c3, the subnetwork (307 genes in c3, 61 genes in c0) is associated with regulation of dendritic spine development (*P* = 2.7E-04), myelination (*P* = 9.9E-04), neuronal cell–cell adhesion (*P* = 1.1E-03), and protein localization to synapse (*P* = 2.1E-03) in c3 and associated with transition metal ion transport (*P* = 1.1E-04), substantia nigra development (*P* = 3.3E-04), and regulation of neuron apoptotic processes (*P* = 6.5E-04) in c0 (Supplementary Table S37).

We also identify a few key drivers in astrocytes, including *WDR49* and heat shock protein family genes such as *HSPA1B, HSPB1, HSP90AA1*, and *HSPH1. WDR49* is downregulated in PD and highly ranked as a key driver in cluster 2 astrocytes (KDA: FDR = 7.6E-12, 5.53 FE; expression: 0.9 FC, FDR *P* = 3.4E-05). Interestingly, *WDR49* encodes a protein with unknown function, but a recent study suggests *WDR49* as the disease-associated subpopulation in frontotemporal dementia carrying the GRN mutation and Alzheimer’s disease [65]. The subnetwork around *WDR49* (100 genes) is involved in the phosphatidic acid process (FDR *P* = 9.0E-03) (Supplementary Fig. S8D, Supplementary Tables S36). Interestingly, a recent study shows that α-Synuclein (α-Syn), a protein causally linked to PD, binds to vesicles containing phosphatidic acid (PA), a plasma membrane phospholipid [66]. The identification of *WDR49* as a key driver opens a new avenue for research into its potential role in PD pathogenesis.

We also identify key drivers in microglial clusters. One driver is *APBB1IP*, which is prominently associated with activation and modulation of the innate immune response. *APBB1IP* is a regulator of leukocyte recruitment and pathogen clearance through complement-mediated phagocytosis [67, 68]. In our analysis, *APBB1IP* is moderately downregulated (KDA: FDR = 8.5E-10, FE = 5.45; expression: FC = 0.85, FDR = 1.1E-09), and the subnetwork around *APBB1IP* is involved in synapse pruning (FDR *P* = 6.7E-04), microglial cell migration (FDR *P* = 3.2E-02), and macrophage migration (FDR *P* = 4.7E-02) (Fig. 6E, Supplementary Tables S36–S37). The observed association between *APBB1IP* and pathways related to microglial activation underscores its importance in disease-related glial responses. Interestingly, the direct effectors of *APBB1IP*, such as *C3* and *CX3CR1*, are also downregulated and involved in immune responses and inflammation (Fig. 6E). Moreover, *ST6GAL1*, an upstream gene of *APBB1IP*, encodes a member of the glycosyltransferase family, ST6Gal-I sialyltransferase, which is known to regulate several immunological processes such as immunoglobulin production in B cells [69] or inflammatory pathways [70]. Therefore, our findings further underscore the importance of *APBB1IP* and its surrounding effectors in driving immune responses in microglia.

Last but not least, *HSP90AA1* is identified as a key driver and is consistently upregulated across multiple glial cells, including OPCs (KDA: FDR = 3.6E-11, 10.4 FE; expression: 1.3 FC, FDR = 9.9E-13), oligodendrocytes in c3 (KDA: FDR = 1.2E-07, 3.69 FE; expression: 1.1 FC, FDR = 2.7E-08), oligodendrocytes in c0 (KDA: FDR = 1.7E-05, 3.52 FE; expression: 1.2 FC, FDR = 0.005), microglia (KDA: FDR = 3.2E-06, 4.02 FE; expression: 1.2 FC, FDR *P* = 9.7E-11), and astrocytes (KDA: FDR = 0.02, 4.3 FE; expression: 1.1 FC, FDR = 1.2E-068) (Fig. 6E, Supplementary Fig. S9F–I). In the OPC cluster c5, the subnetwork around *HSP90AA1* is involved in responses to unfolded protein (FDR = 1.9E-10), protein ubiquitination (FDR = 7.8E-06), and apoptosis (FDR = 6.4E-05) (Fig. 6E, Supplementary Table S36). The subnetworks around *HSP90AA1* in other glial cells are enriched for similar pathways (Supplementary Tables S37–S38). The consistent regulatory pattern of *HSP90AA1* in glial cells suggests its potential involvement in maintaining glial homeostasis and contributing to the underlying neurodegenerative mechanisms.

## Discussion

In this study, we develop an MCMNA framework for analyzing snRNA-seq data and apply it to PD snRNA-seq data to identify cell-type–specific gene modules and key drivers for PD. Use of metacells rather than single cells addresses the challenge of dealing with the intrinsic sparsity in snRNA-seq data. The application of MCMNA to PD systematically identifies gene modules associated with PD and putative key network drivers for PD in the major brain cell types. Notably, 30% of the predicted drivers in neurons have been studied in PD, including *UCHL1, NSF, SNAP25, STMN2*, and *PREPL*, while the rest (70%) are novel or understudied, suggesting that our method can capture previously unrecognized regulators with potential significance in the disease.

Disease-associated networks often exhibit sparse connectivity, reflecting the complexity and heterogeneity of underlying biological processes. Especially in snRNA-seq, some signals can be hidden by sparsity in gene expression profiles. One key advantage of the metacell approach in snRNA-seq is the ability to reduce data sparsity, thereby significantly enhancing downstream biological signals [27–29]. Aggregation of similar cells into metacells allows us to improve the detection of conserved markers specific to each cell type and reduce false-positive differentially expressed genes. Metacell-specific DEGs captured biologically relevant signals, consistent with some previous PD studies. In glial populations, the enrichment of metal ion homeostasis pathways was driven by metallothionein genes, whose dysregulation has also been reported in PD [71]. Additionally, we observed that several canonical dopaminergic neurons (*SLC6A3, TH, SLC18A2*) were downregulated in microglia and astrocytes in PD. Although unexpected, dopaminergic-associated transcriptional signals in glial cells have been reported in a previous dataset [22], suggesting that such signals are not unique to our dataset. Although dopaminergic-associated transcripts detected in glial populations may reflect biologically relevant expression, we cannot fully exclude a contribution from ambient RNA. Future studies incorporating an ambient RNA correction algorithm, such as SoupX [72] or CellBender [73], will help further distinguish cell-intrinsic transcriptional signals from potential background contamination. Similarly, heat shock and proteostasis-related genes were recurrently identified in independent datasets [21, 25], consistent with the involvement of cellular stress responses in PD. Together, these findings support the ability of metacell analysis to detect subtle but biologically coherent disease-associated changes.

Importantly, it strengthens gene–gene correlation analysis, which serves as a foundation for constructing more robust networks. We examine different metacell construction methods and parameter choices (e.g., graining level—the ratio between the number of cells and the number of metacells, and expression representation—average vs. summing up, unweighted vs. weighted) on our dataset to accelerate and enhance substantial downstream analyses. Additionally, the denser and more accurate correlation structure stemmed from biologically meaningful matrix (BMC) results in more meaningful gene coexpression networks. Additionally, the process of pruning enables more accurate construction of causal Bayesian networks.

Our metacell-based MEGENA results show that different modules capture distinct biological pathways and reveal cell-type–specific regulatory patterns. For example, the top-ranked modules in neurons are involved in synaptic transmission and nervous system development, while the top-ranked modules in glial cells are enriched with diverse functions such as developmental processes, metabolic signaling, and immune responses. We identify 3 distinct neuronal clusters, each displaying unique network architecture that reflects the cellular heterogeneity in PD. The typical neuronal cluster c7 with the tyrosine hydroxylase (TH) marker exhibits a more compact and highly connected network structure, while the neuronal cluster c9 with the novel *RIT2* marker has a sparser structure. Cluster c7 has no hierarchical modules that are enriched with PD signatures, indicating that many dysregulated genes in this neuron are rather highly coordinated. Interestingly, in c9 neurons prone to be most degenerated in PD, only a small subset of cells demonstrates the most pronounced network changes (c9_M3), highlighting the selective vulnerability within this cluster. Previous studies using midbrain datasets and mouse models have implicated SOX6^+^/AGTR1^+^ dopaminergic neuronal populations as particularly vulnerable in PD [74–76]. In our human substantia nigra dataset, neither SOX6 nor AGTR1 was identified as a defining feature of c9 or within its dysregulated M3 module (Supplementary Table S23). Given that the substantia nigra is the primary site of dopaminergic neuronal degeneration in PD, our analysis provides a complementary, anatomic, and disease-relevant context for investigating selective neuronal vulnerability. The RIT2-enriched c9 signature therefore represents an additional axis of vulnerability in the human substantia nigra that is not fully captured by the previously described *SOX6*^+^/*AGTR1*^+^ signature. However, differences in species, anatomic sampling, and analytical resolution may also contribute to this apparent distinction, and further investigation is required to determine their relationship.

In addition to neurons, our metacell-based MEGENA allows us to uncover key features within glial cells, highlighting their distinct and potentially underappreciated roles in disease pathologies. Beyond neurons, oligodendrocyte clusters c0 and c3 have gene modules highly associated with PD, revealing broader functional roles than previously described. Several modules in c0 and c3 are enriched for mitochondrial autophagy, apoptosis, oligodendrocyte differentiation, and protein folding, revealing a more dynamic and multifaceted contribution to PD. Additionally, we observe an increased abundance of astrocytes (Supplementary Fig. S1D), yet their functional role in PD remains poorly understood. Our MEGENA pinpoints additional astrocyte involvement, highlighting modules enriched for signaling transduction and synapse assembly, suggesting a possible contribution to maintaining or altering the synaptic environment during disease progression. Microglia, in contrast, have fewer high-ranked modules, which are strongly associated with the immune response pathway. This agrees with well-established knowledge of their roles in neuroinflammation. Additionally, our study also indicates that several OPC modules associated with neural functions and glial differentiation are dysregulated, further highlighting the critical roles of OPCs in PD.

Our module preservation analyses show that module preservation varies substantially across cell types, with the highest score in oligodendrocytes, followed by astrocytes, microglia, and neurons (Supplementary Fig. S4A, B). This indicates that gene coexpression networks are more stable and conserved in glial populations, while neuronal networks are comparatively more dynamic and reflect greater heterogeneity. PD-related modules also have higher preservation overall, suggesting that disease-associated transcriptional programs are more robust and reproducible.

Our analysis identifies both known and novel gene targets that are strongly related to PD-related pathways, allowing us to propose a dynamic mechanistic model of neurodegeneration and susceptibility. Several key drivers in neuronal clusters, such as *CADPS2, NSF*, and *PREPL*, are associated with calcium signaling, synaptic transmission, and protein trafficking and have been studied in PD to some extent [42, 50, 51, 60, 77, 78], highlighting their central role in neuronal functions. We also observe distinct neuronal subpopulations with unique key driver profiles and transcriptomic alterations, suggesting heterogeneity in the degree of the regulatory pattern in degeneration and vulnerability. This observation aligns with previous studies reporting selective neuronal vulnerability in neurodegenerative diseases, including PD [31, 79]. Our findings suggest that multiple pathogenic processes may operate in parallel or sequentially. For example, in novel cluster 9, which shows degeneration in PD, *CADPS2*, enriched for calcium signaling, can point to a novel mechanism of calcium dysregulation, contributing to stress or glial–neuronal signaling imbalance. In contrast, cluster c7 is dominated by key drivers in synaptic transmission, exocytosis, and protein trafficking such as *NSF* or *PREPL*.

Oligodendrocytes in the central nervous system (CNS) are essential for forming myelin sheaths—multilayered lipid membranes that insulate axons [80, 81]. Among the top key drivers we identify in oligodendrocytes, *MOBP* stands out as the highest-ranked gene across both oligodendrocyte clusters c0 and c3. It is the third most abundant protein in CNS myelin and is known to contribute to myelin integrity. Moreover, a recent study suggests a possible association between *MOBP* and PD [82]. Our findings highlight *MOBP* as a potential therapeutic target, given its role in maintaining structural stability and mediating cellular responses. Additionally, key drivers identified in microglia cells point toward compelling hypotheses involving innate immune activation. Consistent with the important role of *APBB1IP* suggested by our network, *APBB1IP* has been identified as a modulator of immune gene coexpression in a specific microglial subtype in a recent aged mouse hippocampus study [83]. It is also recognized as a core component of microglial expression signatures [84, 85]. Furthermore, human GWAS has linked *APBB1IP* to both Alzheimer’s disease and schizophrenia [86, 87]. Our findings contribute to this growing body of evidence by highlighting the relevance of *APBB1IP* in PD.

Heat shock proteins (HSPs) are molecular chaperones that manage protein folding and prevent misfolding by regulating protein aggregation and disaggregation in both intracellular and extracellular environments, playing a critical role in maintaining proteostasis [88]. In our study, we observe consistent upregulation of HSPs across multiple glial cell types (Fig. 2B). Pathway analysis further highlights the activation of processes such as protein folding and Hsf1 activation (Fig. 2C). In particular, in PD, HSP60, HSP70, and HSP90 interact with α-synuclein, becoming incorporated into α-synuclein aggregates as they attempt to prevent or repair protein misfolding and accumulation [89–92]. Among these, we nominate *HSP90AA1* as a key candidate, as it is significantly upregulated in 5 glial cell types and ranks highly in our network analysis. Notably, *HSP90AA1* has been identified as a marker of microglial activation in recent PD omics studies [42, 93]. Altogether, these findings support emerging evidence that nonneuronal cells significantly contribute to disease progression through immune signaling, stress response, or other metabolic and cellular responses.

Our results also reveal strong concordance between bulk and snRNA-seq analyses, providing mutual validation of key molecular drivers implicated in PD. Several key drivers previously identified in bulk transcriptomic studies, such as *STMN2* and *RALYL*, are also drivers in our snRNA-seq network. This overlap underscores the robustness of our network-based approach and demonstrates that strong, biologically relevant signals identified in bulk are not only preserved but also refined at the single-cell level. The replication of these findings confirms the reliability of bulk-based insights while offering higher-resolution interpretation of cell-type–specific dynamics. By disentangling the contributions of distinct cell populations, we uncover cell-specific regulatory responses, enabling increased specificity in identifying therapeutic targets.

We apply our pipeline to a previously published snRNA-seq dataset to identify key drivers [25]. In this dataset, we identify 121 candidate key driver genes (Supplementary Table S38); however, there is minimal overlap with our results. Only a small number of genes are shared between the 2 analyses, including *HSP90AA1* in glial cells and *CADPS2, CPLX1*, and*SERINC1* in neurons (Supplementary Table S39). The limited concordance likely reflects the smaller sample size of the external dataset, which reduces the robustness of network construction and, consequently, the reliability of key driver identification.

We also highlight the functional contexts of PD GWAS genes using cell-type–specific Bayesian causal networks. Our causal networks in 5 major cell types across 8 cell clusters harbor a total of 509 PD GWAS genes, but only 2 (*NSF* in c7 and *MDGA2* in c6) are identified as key drivers (Supplementary Table S39). Additionally, we predict *UCHL1*, a known PD causal gene, as a putative driver. Moreover, our network reveals a substantial number of novel key driver genes. These understudied genes may represent critical yet unrecognized components involved in disease progression, particularly within specific cell types, enabling a broader and more comprehensive understanding of PD mechanisms.

We also compare our DEG signatures with previously published bulk proteomics datasets of human SN [94]. We retrieve the identified differentially expressed proteins (DEPs) from 2 published cohorts (Supplementary Tables S41–S43) and assess the overlap with cell-type–specific DEGs using FET. The results show that the shared DEPs are predominantly enriched in neuronal clusters, with consistent directionality (Supplementary Fig. S10A, B, Supplementary Tables S44–S45). While bulk proteomics capture an overall signal of the molecular dysregulation in PD, these findings indicate that the greatest and most robust changes are driven by the neuronal population. This underscores the importance of cell-type–resolved analyses for accurately interpreting disease-associated signatures.

In summary, our study identifies a set of molecular subnetworks and putative key driver genes that illuminate the molecular architecture of PD at single-cell resolution. By applying MCMNA to address sparsity in snRNA-seq, we uncover well-known genes and novel regulatory candidates that may have important roles in PD. These findings offer a better understanding of the cellular heterogeneity and regulatory dynamics underlying neuronal and glial cell populations.

### Limitations of the study

Although this cohort is the largest dataset of SN to date, the sample size is still relatively small. Furthermore, the current study lacks experimental validation to confirm computational predictions. Even though we observe that previous perturbation signatures overlapped with our predicted *STMN2* network in c7 neurons, more validation is needed for the novel targets. Future work will focus on expanding the analysis to larger and more diverse datasets and implementing different confounding factors, such as sex and Braak stages in the study, to get more insights into the dynamic regulatory machinery. Functional perturbation of prioritized regulators in relevant *in vitro* and *in vivo* models to determine whether these predicted drivers directly contribute to the disease pathogenesis and represent tractable therapeutic targets. Our approach can also be improved with comprehensive longitudinal studies, extended studies beyond sporadic cases and specific brain regions, and incorporation of other omics-based networks such as proteomics and epigenomics. More importantly, this integrative approach establishes a foundation to guide future efforts toward generating increasingly high-quality and informative coexpression and causal inference, paving the way for deeper mechanistic insights and the discovery of novel therapeutic targets.

## Methods

### Postmortem substantia nigra pars compacta samples used

The framework we develop in this article is created using a discovery cohort from our previous study of human 23 PD cases and 9 controls that contain 30,038 genes across 315,867 nuclei [20]. To validate some of our findings, we used 3 publicly available snRNA-seq datasets of human substantia nigra: the Lee et al. dataset [21] of 6 PD and 6 control cases, the Martirosyan et al. dataset [22] of 15 PD and 14 control cases, and the Ma et al. [25] dataset of 11 PD and 13 control cases.

### SnRNA-seq data preprocessing and clustering analysis

Three publicly available snRNA-seq datasets were downloaded and subjected to the same pipeline we already established in the previous study [20]. Briefly, from Cell Ranger–derived unique molecular identifier (UMI) count matrices from all sequencing libraries, we performed quality control (QC) by removing low-quality nuclei with too few genes (<200) or an excessive number (>2,500) of genes detected. Then we removed insufficiently detected genes and discarded mitochondrial genes. We then performed a preclustering analysis using an scRNA-seq data integration workflow based on R packages Harmony [95] (v.1.2.3) and Seurat (v.4.3.0.1) [96, 97]. A total of 2,000 most variable gene features were identified, scaled, and centered after regressing out covariates sex, age, and post-mortem interval (PMI). Next, dimensional reduction was performed using principal component analysis (PCA) based on the 2,000 most variable genes. Preclustering analysis, cluster stability analysis, and double prediction analysis were performed as previously described [20]. As a result, we obtained 28,339 genes across 44,450 high-quality nuclei in the KAIST dataset, 33,537 genes across 69,147 high-quality nuclei in the VIB dataset, and 31,280 genes across 194,905 high-quality nuclei in the Columbia dataset.

### Cluster cell-type annotation

For each major cluster of the 3 external datasets, we interrogated the expression patterns of known gene markers to annotate clusters into major cell types: neurons (*SLC17A6, RBFOX3, GAD1*), astrocytes (*AQP4*), oligodendrocytes (*MOG*), microglia (*C3, CSF1R*), oligodendrocyte progenitor cells (*VCAN*), endothelial cells (*FLT1*), pericytes (*PDGFRB*), and T cells (*SKAP1*). Additionally, we used sets of marker genes we identified in our study, such as typical dopaminergic markers in c7 (*SLC6A3, TH, SLC18A2*), enriched markers in c6 (*DCC* and *GALNTL6*), and novel enriched markers in c9 (*RIT2*).

### Construction of metacells

Metacells were built based on a log-normalized gene expression matrix using the R package SuperCell (v.1.0.1) [29]. The set of features (genes) used for the construction of metacells was defined by the set of the 2,000 most variable genes. The top principal components (top 10 by default) are used to build a single-cell network with a k-nearest neighbors algorithm built in the R package (k-NN, k = 5 by default). To preserve the cell-type annotation and phenotype information from snRNA-seq data, metacells were constructed by merging single cells (nodes) at a graining level γ = 15 in a cell cluster– and condition-specific manner. A gene expression profile of metacells was computed by averaging gene expression within metacells, or summing up the single-cell raw values, followed by log normalization.

### Identification of differentially expressed genes

DEGs between PD and control and DEGs between PD and control in each cluster were identified using the R package MAST (v.1.16.0) implemented in Seurat [96, 97], with correction for PMI, age, and sex. DEGs were identified at the cutoff of a Bonferroni-corrected *P* value ≤0.05 and fold change ≥10%.

### Identification of conserved markers

A conserved marker is defined as a cell-type–specific gene that distinguishes a given cell type from others and shows consistent expression across 2 groups (control and PD). Conserved markers in each cluster were identified using the *FindConservedMarkers* function in the R Seurat package [96, 97]. Genes that reached the average expression levels across the cluster with logFC >0 were identified as conserved markers.

### Overlap and functional enrichment analysis

To understand biological processes underlying the DEGs and coexpressed modules, we tested these signatures for enrichment of Gene Ontology (GO) and canonical functional pathway gene sets. We used the functional gene sets collection from the Molecular Signature Database MSigDb that was downloaded from the R package called *msigdb (v.25.1.1)*. Functional enrichment of DEGs with MSigDB gene annotation collections was examined using the R package *clusterProfiler (v.4.14.6)* [98]. To test the pathways of the 2-layer network neighborhood of candidate genes, we loaded the gene members onto the *EnrichR* server and identified the top pathways associated with these genes [99–101]. Functional enrichment analysis between 2 gene sets was determined by FET. FE was calculated as the ratio between observed overlap and expected overlap size. We employed the Benjamini–Hochberg (BH) approach to correct for FDR.

### Generation of the BMC matrix

We implemented a novel framework to generate a BMC matrix. First, we filtered out unexpressed genes using a range of minimum cell cutoffs (i.e., requiring that each gene be expressed in at least x% of cells), selected genes that were conserved markers, and removed noisy cells (those expressing fewer than 500 genes). For each gene pair, we excluded zero counts before calculating all possible signed SCCs (FDR = 1) using the *calculate.rho.signed* function in MEGENA (v.1.6.0) [32]. To capture all signed correlations, we set direction as “positive” or “negative,” then combined results from both directions.

Next, we evaluated whether the distribution of the correlation coefficient (ρ) in each cell cluster followed a truncated normal distribution. For each cell cluster at each minimum cell cutoff, we first calculated the mean and standard deviation of the observed ρ values and used them to define a truncated normal distribution bounded by the minimum and maximum observed ρ. The truncated normal density was generated at 100 equally spaced points across this range using the *truncnorm* function in the R package (v 1.0–9). We applied linear interpolation using the *approx* function in R to estimate the histogram densities at 100 equally spaced points across the observed range of correlations. We then calculated the mean squared error (MSE) using the following equation: $MSE = \ \frac{1}{n}\mathop \sum \limits_{i = 1}^n ( {{{Y}_i} - \ {{{\hat{Y}}}_i}} )$, where *n* is the number of evaluation points (100 in this case) in the correlation distribution, *Y* is the observed counts of the SCC, and $\hat{Y}$ is the predicted counts of SCCs from the stimulated truncated normalization distribution.

We then plotted MSE values against the minimum cell cutoff to identify the optimal cutoff. The mean (µ) of all gene pair correlations was calculated. Finally, at the optimal minimum cell cutoff, all significant gene pair correlations (FDR < 0.05) were identified. However, even after applying FDR < 0.05, a substantial fraction of gene pairs fell within the (0, µ) interval across clusters ranging from 0% in c9 to 47% in C5 (Supplementary Table S24). Therefore, at the optimal mincell cutoff, we further subtracted gene pairs in the (0, µ) interval. This new set of significant correlations, named the BMC matrix, potentially removed weak and suspicious associations prior to network construction.

### MEGENA coexpression network analysis

For each cell cluster, the optimized BMC matrix was then subjected to the MEGENA pipeline [32] (v.1.6.0). Briefly, significant SCCs were ranked and iteratively tested for planarity to grow a planar filtered network (PFN) by using the PMFG algorithm. Multiscale clustering analysis (MCA) was conducted with the resulting PFN to identify coexpression modules at different network-scale topologies. We identified a total of 1,057 modules in all cell clusters (Table 3). To annotate the biological functions associated with the modules, we performed MSigDB gene set enrichment analysis using FET as described above. Most of these modules (42.2–73.3%) were enriched for MSigDB GO Pathway gene sets (adjusted *P* < 0.05; Table 3), indicating that MEGENA can capture biologically functional signals. For simplicity, modules were annotated by the top enriched pathway and then grouped into a functional category. The MEGENA modules are formed in a hierarchy with parent–child relationships and illustrated by a sunburst plot using the R package *ggsunburst*.

To prioritize the gene modules with respect to the PD phenotypes, we applied a ranking metric across multiple features such as enrichment with (i) the DEG signatures identified and (ii) PD GWAS signatures. PD GWAS hits were downloaded from the GWAS catalog and filtered to remove redundance. The ranking of the top modules was based on the average of −log10 (*P*adj value) of all features using this equation.


\begin{eqnarray*}
\textit{Module}\ \textit{Scor}{{e}_m} = \ \frac{{ - lo{{g}_{10}}\left( {P_{adj,\ m}^{\textit{Upregulated}\ DEG}} \right) + - lo{{g}_{10}}\left( {P_{adj,\ m}^{\textit{Downregulated}\ DEG}} \right) + - lo{{g}_{10}}\ \left( {P_{adj,\ m}^{\textit{GWAS}}} \right)}}{3},
\end{eqnarray*}

where $P_{adj,\ m}^{\textit{Upregulated}\ DEG}$, $P_{adj,\ m}^{\textit{Downregulated}\ DEG}$, and $P_{adj,\ m}^{\textit{GWAS}}$ are the adjusted *P* values from the FET enrichment test for the DEGs and GWAS signatures, respectively, for module m. Higher scores indicate modules with stronger combined enrichment for disease-associated transcriptional and genetic signals.

The circular track plot to visualize the gene coexpression network modules and their enrichment was generated using the R package *NetWeaver (v.0.0.6)* [102]. The global network of the c9 neuron was visualized using Gephi (version 0.10.1). The global network of other cell clusters and the subnetwork of selected gene modules were visualized using Cytoscape (version 3.10.3).

### MEGENA coexpression module preservation analysis

We assessed module preservation in the MEGENA coexpression network between our MSSM snRNA-seq data and Columbia’s snRNA-seq data using the FET, with genes from the corresponding global network in our dataset as the background. We performed pairwise comparisons of modules within the same cell type (e.g., microglia modules vs. microglia modules).

Additionally, we used module preservation analysis in WGCNA (v.1.73) [37]. For each cluster, MEGENA modules identified in the MSSM snRNA-seq dataset were used as reference modules, and module preservation was evaluated in the Columbia dataset. Expression matrices were restricted to genes available in both datasets. Because MEGENA allows a single gene to belong to multiple modules, each gene–module membership was treated as a separate feature. Prior to the preservation analysis, the expression datasets were evaluated using the goodSamplesGenes function, and the genes or the samples with zero variance or excessive missing values were removed. Module preservation was then evaluated using the modulePreservation function, which took the MSSM gene coexpression networks as the reference networks and the Columbia dataset as the testing dataset. The composite module preservation statistics, Zsummary.pres, was calculated for each module. Modules with Zsummary.pres ≥ 10 were classified as highly preserved, modules with 2 < Zsummary.pres < 10 were considered moderately preserved, and modules with Zsummary.pres ≤ 2 were considered not preserved.

### Bayesian probabilistic causal network inference

We constructed the global BN based on Monte Carlo Markov chain simulation-based procedures using RIMBANet software [33, 34]. We used established TF–target relationships for *Homo sapiens* acquired from the TFLink database [103] to infer regulatory relationships among genes. In the causal network construction, the TFs are allowed to be parent nodes of their target genes, but targets are inhibited from being parent nodes of their TFs.

First, we began by removing genes expressed in fewer than 20% of metacells. To resolve the issue of dropouts in snRNA-seq, we applied an iterative pruning procedure to remove genes and nuclei in a data matrix. For this, we defined 2 thresholds: Tg, representing the maximum allowable gene missing rate, and Tm, representing the maximum allowable metacell missing rate. For each iteration, genes exceeding the Tg threshold and metacells exceeding the Tm threshold were removed. This pruning process was repeated iteratively until the number of remaining genes and metacells stabilized, with no changes observed between iterations. After 2 iterations, the numbers converged. We then selected the cutoff Tg = 0.4 and Tm = 0.4 for all cell clusters.

We employed a network averaging approach where 1,000 networks were generated starting with a different random structure. Links that appear in more than 30% of the networks were used as a final consensus network. An iterative de-loop procedure was executed to ensure that the consensus network was a directed acyclic graph, removing the most weakly supported link of all links involved in any loop. The global network of cell clusters was visualized using Cytoscape (version 3.10.3).

### KDA

For each major cell type cluster, we performed KDA [35] on the consensus Bayesian network to identify key hub genes that are DEGs and regulate a large number of downstream nodes. Here we loaded all the BN nodes and DEGs from the corresponding cluster as input for KDA, making the resulting key drivers representative of the global network. The algorithm defined a background subnetwork by identifying nodes within an L-layer neighborhood of each DEG in the network. For each node in this subnetwork, it assessed enrichment within its L-layer downstream neighborhood for DEGs. In our analysis, we tested a combination of 1-layer (1 L), 2-layer (2 L), and 3-layer (3 L) neighborhoods, considering both directed and undirected edges to account for potential missing directionality in the causal BN. Then key drivers were compiled based on those identified from the 3 L directed analysis and those shared by both 2 L and 1 L undirected analyses. To ensure that key drivers had sufficient downstream targets, we also excluded drivers with a degree lower than the average degree of all nodes in the network. KDA was performed by the R package *KDA* (version 0.02).

### PubMed literature mining

The function EUtilsSummary (type = “esearch,” db=“pubmed,” datetype=“pdat”) from the R package RISmed (version 2.3.0) was used to query and download content from PubMed. The query words were built as “KDG and Parkinson’s disease,” where the word KDG was replaced by the gene symbol of a specific key driver gene. For example, the query of “CADPS2 and Parkinson’s disease” would pull out all the papers that have the occurrence of CADPS2 and Parkinson’s disease.

### Statistical analysis

FET was used to examine the significance of the overlap between 2 gene sets. An overlap with BH-corrected *P* < 0.05 was considered statistically significant unless stated otherwise.

## Supplementary Material

giag096_Supplemental_Files

giag096_Authors_Response_To_Reviewer_Comments_original_submission

giag096_GIGA-D-26-00192_original_submission

giag096_GIGA-D-26-00192_revision_1

giag096_Reviewer_1_Report_original_submission Reviewer 1 -- 7/12/2026

giag096_Reviewer_1_Report_revision_1 Reviewer 1 -- 9/19/2026

giag096_Reviewer_2_Report_original_submission Reviewer 2 -- 7/16/2026

giag096_Reviewer_2_Report_revision_1 Reviewer 2 -- 9/30/2026

## Data Availability

This study did not generate new unique reagents. The raw data of our snRNA-seq [20] are available from the Gene Expression Omnibus (GEO) under the accession code GSE184950. The raw data from Lee et al. [21] are available from GEO under the accession code GSE148434. The raw data from the Martirosyan et al. dataset [22] are available from GEO under the accession code GSE243639. The raw data from Ma et al. [25] are available from GEO under the accession code GSE253462. The scripts and data used in this study have been deposited on GitHub [104] and Zenodo [105]. Any additional information required to reanalyze the data reported in this study is available upon request from the lead contact.

## Additional files

**Supplementary Fig. S1**. Generation and characterization of metacells. (A, B) Number of upregulated (A) and downregulated (B) differentially expressed genes (DEGs) in single cells and metacells across different conditions in 12 cell clusters. (C) Number of conserved marker-expressed genes (DEGs) in single cells and metacells across different conditions in 12 cell clusters. (D) Nuclei fraction distribution between PD and control across 12 clusters. *P* values were calculated using the Wilcoxon rank-sum test. **P* < 0.05, ***P* < 0.01. (E) Volcano plot showing DEGs in selected major cell clusters c0 (oligodendrocytes), c5 (oligodendrocyte progenitor cells), and c6 (neurons). The top 5 upregulated and 5 downregulated DEGs (by −log10 [FDR]) of each cell cluster are labeled in red or blue, respectively. (F, I, L) UMAP plot showing cell clusters in the VIB dataset [22] (F), KAIST dataset [21] (I), or Columbia dataset [25] (L). (G, J, M) Expression pattern of known brain cell-type marker genes in the VIB dataset [22] (G), KAIST dataset [21] (J), or Columbia dataset [25] (M). (H, K, N) Comparison of Fisher’s exact test (FET) enrichment of DEGs of the MSSM dataset and VIB dataset [22] (H), the MSSM dataset and KAIST dataset [21] (K), or the MSSM dataset and Columbia dataset [25] (N) at the cell cluster level. The size of the dot is proportional to the fold enrichment, and color intensity indicates the value of −log10 (FET FDR). Ast, astrocytes; End, endothelia; Fib, fibroblast-like cells; Neu, neurons; Mic, microglia; Oli, oligodendrocytes; OPC, oligodendrocyte progenitor cells; Per, pericytes; T, T cells.

**Supplementary Fig. S2**. Comparison of the enrichments of all pathways in the single-cell (SC) and metacell (MC) DEG signatures. The −log10 (FDR) values of all pathways from (A) upregulated or (B) downregulated differentially expressed genes (DEGs) were plotted. Black dot indicates the unique pathways from each method. Spearman correlation was calculated from all pathways in each cluster.

**Supplementary Fig. S3**. Generation of biologically meaningful correlation (BMC) matrix. (A) Workflow of the method. (B) Correlation distribution of SC and MC across 9 cell clusters. SC and MC at the min cell cutoff = 20%. (C) Plot of mean square error values against different min cell cutoffs across 12 cell clusters. (D) Plot of gene expression versus gene network connectivity (the number of nodes that directly connect with a given node in a gene network).

**Supplementary Fig. S4**. Summary of the module preservation analysis of the MSSM and Columbia datasets. (A) Module preservation summary for all the coexpressed gene modules. (B) Module preservation summary for the PD-associated coexpressed gene modules within the same cell type.

**Supplementary Fig. S5**. Metacell-based gene coexpression of neuronal cell clusters. (A) Enrichment of the DEGs of PD in MEGENA modules in cluster 6 neurons. Color intensity is proportional to −log10 (corrected FET *P* value). (B, C) Enrichment of the major pathways in MEGENA modules in cluster 6 neurons (B) and cluster 7 neurons (C). (D) The global MEGENA-based gene coexpression network in cluster 7 (neurons). The pie chart of each node indicates whether it is a DEG or GWAS gene. The size of a node is proportional to the node connectivity in the MEGENA network. DN, downregulated; UP, upregulated.

**Supplementary Fig. S6**. Metacell-based gene coexpression of the oligodendrocyte clusters. (A, D) Enrichment of the major pathways in MEGENA modules in cluster 3 (A) and cluster 0 (D). (B, C, F) The top-ranked module M41 of cluster 3 (B), M42 of cluster 3 (C), and M3 of cluster 0 (F). The pie chart of each node indicates whether it is a DEG or GWAS gene. The size of a node is proportional to the node connectivity in the MEGENA network. (E) Enrichment of the DEGs of PD in MEGENA modules in cluster 0. Color intensity is proportional to −log10 (corrected FET *P* value). DN, downregulated; UP, upregulated.

**Supplementary Fig. S7**. Metacell-based gene coexpression of other glial cell clusters. (A, C, G) Enrichment of the major pathways in MEGENA modules in cluster 2 (astrocytes) (A), cluster 1 (microglia) (C), and oligodendrocyte progenitor cells (OPCs) (G). (B, D, E, H, I) The top-ranked module M6 of cluster 2 (astrocytes) (B), M16 of cluster 1 (microglia) (D), M12 of cluster 1 (microglia) (E), M6 of cluster 5 (OPC) (H), and M8 of cluster 5 (OPC) (I). The pie chart of each node indicates whether it is a DEG or GWAS gene. The size of a node is proportional to the node connectivity in the MEGENA network. (F) Enrichment of the DEGs of PD in MEGENA modules in cluster 5 (OPC). Color intensity is proportional to −log10 (corrected FET *P* value). DN, downregulated; UP, upregulated.

**Supplementary Fig. S8**. Bayesian probabilistic causal network (BN) of neuronal cells. (A) BN in cluster 6 (neurons). (B–D) The 2-layer network neighborhoods of *RALYL* in cluster 9 neurons (B), *NSF* in cluster 7 neurons (C), and *MDGA2* in cluster 6 neurons (D), respectively. The size of a node is proportional to the node connectivity in the BN. The directionality of the arrow indicates the upstream–downstream effect. Nodes with burgundy labels are key drivers in that network.

**Supplementary Fig. S9**. Bayesian probabilistic causal network (BN) of glial cells. (A, C, E) BN in cluster 0 (oligodendrocytes), C2 (astrocytes), and C5 (OPCs). (B, D, F–I) The 2-layer network neighborhoods of *MOBP* in cluster 0 (oligodendrocytes) (B), *WDR49* in cluster 2 (astrocytes) (D), *HSP90AA1* in cluster 0 (oligodendrocytes) (F), cluster 1 (microglia) (G), cluster 2 (astrocytes) (H), and cluster 3 (oligodendrocytes) (I), respectively. The size of a node is proportional to the node connectivity in the BN. The directionality of the arrow indicates the upstream–downstream effect. Nodes with burgundy labels are key drivers in that network.

**Supplementary Fig. S10**. Comparison of FET enrichment of DEGs of the MSSM dataset and DEPs of the bulk proteomics study (A) using a cohort of 15 PD and 15 control cases or (B) using the cohort of 10 PD and 9 control cases. The size of the dot is proportional to the fold enrichment, and color intensity indicates the value of −log10 (FET FDR). Ast, astrocytes; End, endothelial cells; Fib, fibroblast-like cells; Neu, neurons; Mic, microglia; Oli, oligodendrocytes; OPC, oligodendrocyte progenitor cells; Per, pericytes; T, T cells.

**Supplementary Table S1**. Seurat data sizes for the SC and MC methods across MSSM, KAIST, VIB, and Columbia datasets.

**Supplementary Table S2**. Comparison of major pathways between the SC and MC methods in the MSSM dataset.

**Supplementary Table S3**. Differentially expressed genes (DEGs) identified by the metacell (MC) method per cell cluster.

**Supplementary Table S4**. Conserved marker genes in the MSSM dataset identified by metacell.

**Supplementary Table S5**. Distribution of metacell sizes across cell clusters in the VIB dataset.

**Supplementary Table S6**. Differentially expressed genes (DEGs) identified by the metacell (MC) method per cell cluster in the VIB dataset.

**Supplementary Table S7**. Distribution of metacell sizes across cell clusters in the KAIST dataset.

**Supplementary Table S8**. Differentially expressed genes (DEGs) identified by the metacell (MC) method per cell cluster in the KAIST dataset.

**Supplementary Table S9**. Distribution of metacell sizes across cell clusters in the Columbia dataset.

**Supplementary Table S10**. Differentially expressed genes (DEGs) identified by the metacell (MC) method per cell cluster in the Columbia dataset.

**Supplementary Table S11**. Fisher’s exact test (FET) pathway overlap analysis between the VIB and MSSM datasets.

**Supplementary Table S12**. Fisher’s exact test (FET) pathway overlap analysis between the KAIST and MSSM datasets.

**Supplementary Table S13**. Fisher’s exact test (FET) DEG overlap analysis between the Columbia and MSSM datasets.

**Supplementary Table S14**. Shared enriched pathways across all cell types between the metacell (MC) and single-cell (SC) methods.

**Supplementary Table S15**. Summary statistics of shared and unique upregulated pathways identified by the SC and MC methods.

**Supplementary Table S16**. Summary statistics of shared and unique downregulated pathways identified by the SC and MC methods.

**Supplementary Table S17**. Unique enriched pathways per cell cluster identified by the metacell (MC) method.

**Supplementary Table S18**. Unique enriched pathways per cell cluster identified by the single-cell (SC) method.

**Supplementary Table S19**. Unique DEGs per cell cluster identified by the metacell (MC) method.

**Supplementary Table S20**. Enriched pathways per cell cluster driven by unique DEGs per cell cluster identified by metacell (MC) method using the EnrichR database.

**Supplementary Table S21**. Intersection analysis of unique DEGs identified by the metacell (MC) method and DEGs in the Columbia and KAIST datasets.

**Supplementary Table S22**. Fisher’s exact test (FET) results for STMN2 pathway preservation.

**Supplementary Table S23**. Gene membership in significant coexpression modules per cell cluster.

**Supplementary Table S24**. GWAS genes used in the study.

**Supplementary Table S25**. Module ranking and functional pathway annotation per cell cluster.

**Supplementary Table S26**. Comprehensive Fisher’s exact test (FET) results across all MEGENA modules.

**Supplementary Table S27**. Complete pathway enrichment information for all MEGENA modules.

**Supplementary Table S28**. Summary of module preservation of all modules at cell-type–specific levels.

**Supplementary Table S29**. FET results of module preservation of all modules at cell-type–specific levels.

**Supplementary Table S30**. Module preservation of PD-related modules at cell-type–specific levels.

**Supplementary Table S31**. FET results of module preservation of PD-related modules at cell-type–specific levels.

**Supplementary Table S32**. Module preservation analysis of all the modules by WGCNA at cell-type–specific levels.

**Supplementary Table S33**. Summary of module preservation analysis by WGCNA at cell-type–specific levels.

**Supplementary Table S34**. Number of TF and genes removed during pruning process.

**Supplementary Table S35**. Detailed key driver genes identified in major cell types.

**Supplementary Table S36**. Two-layer Bayesian network of selected key drivers.

**Supplementary Table S37**. Pathway enrichment results for the two-layer network of a key driver gene using the EnrichR database.

**Supplementary Table S38**. Detailed key drivers gene identified in major cell types in the Columbia dataset.

**Supplementary Table S39**. Shared key drivers in 2 datasets.

**Supplementary Table S40**. GWAS genes mapped to cell-type–specific gene coexpression networks.

**Supplementary Table S41**. Number of gene pairs before and after BMC filtering.

**Supplementary Table S42**. Differentially expressed proteins (DEPs) identified in the published cohort of 15 PD and 15 control samples.

**Supplementary Table S43**. Differentially expressed proteins (DEPs) identified in the published cohort of 9 PD and 10 control samples.

**Supplementary Table S44**. Fisher’s exact test (FET) results of shared DEGs and DEPs from the cohort of 15 PD and 15 control samples.

**Supplementary Table S45**. Fisher’s exact test (FET) results of shared DEGs and DEPs from the cohort of 9 PD and 10 control samples.

## Abbreviations

BH: Benjamini–Hochberg; BMC: biologically meaningful correlation; CNS: central nervous system; DA: dopaminergic; DEG: differentially expressed gene; DEP: differentially expressed protein; FC: fold change; FDR: false discovery rate; FE: fold enrichment; FET: Fisher’s exact test; GO: Gene Ontology; GRN: gene regulatory network; GWAS: genome-wide association studies; HSP: heat shock protein; KD: key driver; KDA: key driver analysis; LB: Lewy body; MCA: multiscale clustering analysis; MCMNA: metacell-based multiscale network analysis; MEGENA: Multiscale Embedded Gene co-Expression Network Analysis; MSE: mean square error; OPC: oligodendrocyte progenitor cell; PA: phosphatidic acid; PCA: principal component analysis; PD: Parkinson’s disease; PFN: planar filtered network; QC: quality control; sc/snRNA-seq: single-cell/single-nucleus RNA sequencing; SCC: Spearman correlation coefficient; SN: substantia nigra; TH: tyrosine hydroxylase; UMI: unique molecular identifier; WGCNA: weighted gene coexpression network analysis.
