# Supplementary material for: Multiscale transcriptomic network models of Parkinson’s disease at the single-cell level
Source: Gigascience. 2026 Oct 6;15:giag096. doi: 10.1093/gigascience/giag096 (PMC13723071; doi:10.1093/gigascience/giag096)
Supplement: Supplementary file 1 [file giag096_authors_response_to_reviewer_comments_original_submission.pdf]

Author's Response To Reviewer Comments

We sincerely appreciate the very thoughtful and constructive comments from the two reviewers, which provided us with valuable insights and opportunities to greatly improve the quality of the manuscript. We have revised the manuscript according to the reviewers' comments and provided a point-by-point response to each suggestion below.
Responses to the reviewers' comments are provided below with our responses given in blue. Please also see the cover letter and responses to comments PDF file which contains the same information here but is much easier to read.

Editor
GIGA-D-26-00192
Multiscale Transcriptomic Network Models of Parkinson's Disease at the Single Cell Level
Chu Linh; Gefei Yu; Xianxiao Zhou; Minghui Wang; Zhenyu Yue; Bin Zhang
GigaScience

Dear Prof. Zhang,

Your manuscript "Multiscale Transcriptomic Network Models of Parkinson's Disease at the Single Cell Level" (GIGA-D-26-00192) has been assessed by our reviewers. Although it is of interest, we are unable to consider it for publication in its current form. The reviewers have raised a number of points which we believe would improve the manuscript and may allow a revised version to be published in GigaScience.

Their reports, together with any other comments, are below. Please also take a moment to check our website at https://www.editorialmanager.com/giga/ for any additional comments that were saved as attachments.

In addition, please register any new software application in the bio.tools and SciCrunch.org databases to receive RRID (Research Resource Identification Initiative ID) and biotoolsID identifiers, and include these in your manuscript. Computational workflows should be registered in workflowhub.eu and the DOIs cited in the relevant places in the manuscript. These will facilitate tracking, reproducibility and re-use of your tool.

If you are able to fully address these points, we would encourage you to submit a revised manuscript to GigaScience. Once you have made the necessary corrections, please submit online at:

https://www.editorialmanager.com/giga/

If you have forgotten your username or password please use the "Send Login Details" link to get your login information. For security reasons, your password will be reset.

Please include a point-by-point within the 'Response to Reviewers' box in the submission system. Please ensure you describe additional experiments that were carried out and include a detailed rebuttal of any criticisms or requested revisions that you disagreed with. Please also ensure that your revised manuscript conforms to the journal style, which can be found in the Instructions for Authors on the journal homepage. If the data and code has been modified in the revision process please be sure to update the public versions of this too.

The due date for submitting the revised version of your article is 18 Oct 2026.

I look forward to receiving your revised manuscript soon.

Best wishes,

Weixue Mu
GigaScience

www.gigasciencejournal.com

Response #1: Thank you for this recommendation. The software/code developed for this study has already been archived in Zenodo and assigned a persistent DOI (10.5281/zenodo.20834299]), which is cited in the manuscript to ensure long-term accessibility and reproducibility.

Reviewer #1:
Chu et al.'s manuscript offers a detailed Multiscale Transcriptomic Network Analysis (MCMNA) pipeline, which seeks to decipher the molecular mechanisms of Parkinson's Disease (PD) through the use of single-nucleus RNA-sequencing (snRNA-seq) data.
By applying a metacell aggregation approach to cluster log-normalised gene expression matrices, the authors effectively address the inherent sparsity and noise of snRNA-seq datasets. The integration of Multiscale Embedded Gene Co-expression Network Analysis (MEGENA) with Bayesian probabilistic causal networks successfully identifies both known targets (such as UCHL1 and NSF) and novel putative driver genes (such as CADPS2 and WDR49) across major neuronal and glial cell types.
In summary, the study employs a robust methodology and offers a significant computational framework. However, certain analytical parameters, integration steps, and correlation assumptions require further clarification to ensure full reproducibility and robustness.
Response #2: We sincerely thank the reviewer for the highly positive evaluation of our study and for the insightful and constructive comments and suggestions.

Major Comments
1. The authors outline a method to tackle data sparsity by eliminating zero-values for every gene pair before computing Spearman correlation coefficients for the BMC matrix. While this strategy effectively enhances the overall correlation distribution in comparison to single-cell data, excluding all zero counts may artificially inflate correlation structures, potentially overlooking biologically important transcripts with low expression levels. The authors need to include an additional sensitivity analysis as supplementary material, showing that the complete exclusion of a particular set does not unintentionally favor genes that are universally highly expressed.
Response #3: We appreciated the important suggestion. To evaluate whether excluding zero-count unintentionally favor genes that highly expressed, we performed a sensitivity analysis examining the relationship between each gene’s average expression level and its network activity (defined as the node degree in the MEGENA network). For each cluster, we calculated the average gene expression and correlated it with its node degree using Spearman’s rank correlation. Across the cell clusters, the observed correlations were generally weak ($|\rho| < 0.2$), indicating that network connectivity was not strongly associated with baseline expression level. Only cluster 2 Astrocyte ($\rho = -0.239$) and cluster 7 Neuron ($\rho = -0.323$) showed modest negative correlation, suggesting that genes with lower average expression tended to have slightly higher network connectivity rather than the opposite. These findings indicate that excluding zero-count observations did not artificially bias the inferred co-expression network structure or preferentially enrich universally highly expressed genes. We have added these results in Figure Supplement 2D, and described them in the manuscript (Lines 198-204, Page 9).

2. The research employs the Harmony R package for merging data and the MAST package for identifying differentially expressed genes, while accounting for covariates including sex, age, and post-mortem interval (PMI). Considering the study independently reprocesses several external snRNA-seq datasets (the Lee, Martirosyan, and Ma datasets), the authors must clearly address how Harmony successfully reduced batch effects within these technologically and biologically diverse groups. Including a UMAP visualisation of the data before and after integration would greatly strengthen confidence in the alignment.
Response #4: This is a misunderstanding as the external snRNA-seq datasets were reprocessed independently using the same analysis pipeline and no cross-dataset batch correction or dataset merging was performed. Here, the consistency across studies was evaluated by comparing module and pathway-level results.

3. The reliability of network module validation through external datasets hinges on Fisher's Exact Test. Given that the FET necessitates stringent threshold standards for identifying gene set overlaps, the authors might overlook more subtle, rank-based preservation patterns across the datasets. Integrating a threshold-free metric like Gene Set Enrichment Analysis (GSEA) with FET might offer a more robust and

comprehensive evaluation of module consistency across various neuronal and glial clusters.
Response #5: We thank the reviewer for this helpful suggestion. We agree that Fisher's Exact Test (FET), which relies on discrete gene-set overlap, may not capture more subtle patterns of network preservation across independent datasets. To provide a more comprehensive assessment and complement to our original analysis, we have now performed the WGCNA module preservation analysis at the cell type specific level in addition to FET. This approach evaluates whether the connectivity (if genes maintain similar relative connectivity patterns) and density structure of each module is preserved (if the genes in the module remain strongly interconnected in the test dataset) in an independent dataset, without requiring a predefined threshold for gene-set overlap.
We define a module as highly preserved if Zsummary.pres ≥ 10, moderately preserved if 2 < Zsummary.pres < 10, and not preserved otherwise. Similar to the previous FET results, moderate-to-high module preservation was highest in oligodendrocytes (43–53%), followed by microglia (50%), astrocytes (40%), and neurons (~8–44%). Overall, PD-associated modules showed greater moderate-to-high preservation. Consistent with the global findings, PD-associated modules showed the highest preservation in oligodendrocytes (87–97%), followed by microglia (95%), astrocytes (81%), OPCs (71%), and neurons (0–29%). Importantly, among neuronal clusters, PD-associated modules showed greater preservation in Cluster 9 and Cluster 6, but not in Cluster 7 (Supplemental Table S30-31).
These analyses provide an orthogonal assessment of the robustness and reproducibility of the identified network modules across datasets and align with the findings using our FET analysis. These analyses were added into the manuscript (Lines 991-1002, Pages 42-43; Lines 539-544, Page 23).

4. Bayesian causal networks were developed by incorporating transcription factor (TF)-target associations from the TFLink repository as priors. The authors should indicate if these priors were specifically curated for brain- or neural-derived functional genomic information, as transcription factor-target regulatory interactions can show substantial tissue variability.

Response #6: We agree that TF-target regulatory interactions exhibit tissue-specific activity. The TF-target interactions used as priors in our Bayesian causal network analysis were obtained from the TFLink database, which is not restricted to brain- or neural-specific regulatory interactions. Importantly, to our knowledge, there is currently no comprehensive, experimentally validated TF-target regulatory interaction database that is specific to the human substantia nigra or its constituent neuronal and glial cell types. Rather, TFLink is a comprehensive and continuously maintained repository that integrates experimentally validated and manually curated TF-target interactions from multiple public resources and the literature across diverse biological contexts. We selected TFLink because of its broad coverage, high-quality curation, and regular updates, providing a comprehensive set of prior regulatory relationships for Bayesian network inference. These priors were used to constrain the network structure rather than to define brain-specific regulatory programs. We clarified this in the revised manuscript (Lines 1005-1008, Page 43).

Minor Comments
1. Software Provenance: In the GigaScience submission checklist, the authors checked "No" in the Resources section, stating there are no experimental data. However, computational reproducibility requires exact software provenance. The authors should make sure to clearly state the exact version numbers of all R packages used (such as Seurat, SuperCell, MAST, Harmony) in the Methods section.
Response #7: We have updated our method section in the manuscript.

2. The authors observed that weighted aggregation did not consistently enhance downstream analyses while increasing data size, prompting them to choose the unweighted averaging method. Adding the statistical metric used would improve the transparency of the method.
Response #8: We have added supplementary tables (now Supplementary Table S1, S2) to improve the transparency of the method.

Reviewer #2:
Review of "Multiscale Transcriptomic Network Models of Parkinson's Disease at the Single Cell Level"
In this manuscript, the authors present an integrative, data-driven computational framework (MCMNA) to delineate cell-type-specific gene regulatory networks in the substantia nigra of Parkinson's disease (PD). By combining metacell aggregation with co-expression network analysis (MEGENA) and Bayesian causal network inference, the authors seek to overcome the technical sparsity inherent to single-nucleus RNA

sequencing (snRNA-seq) datasets and identify biologically meaningful regulatory modules and putative key drivers across neuronal and glial populations.

This manuscript presents a technically sophisticated computational framework that integrates metacell aggregation, co-expression network analysis and Bayesian causal inference to investigate cell-type-specific molecular networks in Parkinson's disease. The study represents a valuable resource for the field and identifies numerous hypotheses worthy of future investigation. However, several methodological issues—including potential ambient RNA contamination, incomplete validation of the metacell framework, statistical implications of averaging log-normalized expression, dependence on clustering resolution, and overinterpretation of causal and therapeutic implications—should be addressed before the biological conclusions can be fully supported. Addressing these concerns would substantially strengthen the manuscript and improve confidence in the proposed disease mechanisms and candidate regulators.
Response #9: We appreciate the positive evaluation of the study. Please see our responses to the major concerns below.
Major strengths
1. Rigorous analytical framework
The proposed pipeline directly addresses one of the major limitations of single-cell network inference—technical sparsity and dropout. The generation of a Biologically Meaningful Correlation (BMC) matrix to remove weak background correlations prior to co-expression analysis represents a thoughtful strategy to improve network robustness and reduce false-positive interactions.
2. Multiscale characterization of disease-associated networks
The study provides a detailed map of transcriptomic network organization across multiple neuronal and glial populations. In particular, distinguishing the network architecture of the vulnerable RIT2+ (c9) neuronal population from the more compact TH+ (c7) cluster provides potentially important insights into selective neuronal vulnerability in PD.
3. Integration of independent sources of evidence
The prioritization of co-expression modules using both differential expression and PD GWAS enrichment substantially strengthens the biological relevance of the identified networks. This integrative strategy increases confidence that the highlighted modules reflect disease-associated biology rather than statistical artifacts.
4. Identification of putative causal regulators
The Bayesian causal network analysis identifies 138 putative key drivers of PD. Recovery of previously implicated regulators, including STMN2, UCHL1 and NSF, provides confidence in the computational framework, while the identification of numerous novel, cell-type-specific candidates (for example CADPS2 in neurons and MOBP or APBB1IP in glial populations) offers an important resource for future experimental studies.

Major concerns
1. Potential ambient RNA contamination in glial populations
One of the most concerning observations is the presence of canonical dopaminergic neuronal markers (SLC6A3, TH and SLC18A2) among the most significantly downregulated genes in both microglia (c1) and astrocytes (c2) (Figure 2B). These genes are not expected to be expressed in glial populations under normal conditions. This raises the possibility of ambient RNA contamination, doublets, or cell-type misclassification.
The authors should clarify the origin of these signals and describe whether ambient RNA correction methods were applied. Because the downstream differential expression, pathway enrichment and network analyses rely on these gene lists, the robustness of the glial analyses depends on adequately addressing this issue.
Response #10: We thank the reviewer for raising this important concern regarding the detection of dopaminergic markers as differentially expressed genes in glial populations. Several observations suggested that these findings are unlikely to be primarily driven by cell-type misclassification. Cell type annotation was performed at the single-cell level, and the microglia and astrocyte populations retained robust expression of established lineage-specific glial markers without broad neuronal transcriptional signatures. In addition, doublets were carefully identified and removed during quality control, further reducing the likelihood that neuronal-glial doublets account for these signals in our previous publication1. We acknowledge that that ambient RNA correction was not explicitly performed. However, TH, SLC6A3, and SLC18A2 were detected at relatively low levels in glial cells, and their identification as differentially

expressed genes should not be interpreted as indicating high or canonical expression of these genes in glia. Notably, these genes were not significantly differentially expressed at the single-cell level but emerged in the metacell analysis. This is consistent with the increased statistical power of metacell approach, and therefore can reveal subtle disease-associated expression differences.
Importantly, the presence of dopaminergic-associated transcripts in glial populations is not without precedent. Martirosyan et al. 2 reported TH-enriched microglial and astrocytic populations and observed their depletion in PD, providing independent evidence that TH-associated transcriptional signals can be detected in glial populations in the human substantial nigra. We have clarified these points in the revised manuscript (Lines 490-493 , Page 21).

2. Validation of the metacell strategy remains incomplete
The primary motivation for metacell aggregation is to improve signal-to-noise ratio and recover biological signals that are difficult to detect in conventional single-cell analyses. However, the current validation primarily demonstrates preservation of DEGs identified using standard single-cell approaches. While this establishes consistency, it does not demonstrate that the additional DEGs uniquely identified by the metacell approach represent genuine biological discoveries.
The authors should provide additional validation demonstrating that these unique findings reflect improved biological sensitivity rather than aggregation-induced artifacts. Replication in independent datasets, enrichment for established biological pathways, or orthogonal experimental validation would substantially strengthen this conclusion.
Response #11: We thank the reviewer for this important suggestion. To determine whether the additional DEGs detected by the metacell analysis represent biologically meaningful signals rather than aggregation-induced artifacts, we performed pathway-enrichment analysis specifically on the metacell-specific DEGs, which are genes identified as significant by the metacell approach but not by the conventional single-cell analysis (Supplementary Table S19)
The metacell-specific DEGs showed biologically coherent, cell-type-associated enrichment. Across the glial populations, these genes were enriched in pathways related to responses to cadmium and zinc ions and the regulation of ion homeostasis (driven by MT1M, MT1G, MT1X, MT1E) (Supplementary Table S20) Various studies have shown that changes in metal ion homeostasis may lead to damage to the CNS, in neurodegeneration, and in PD3. In neuronal populations, the metacell-specific genes were enriched in pathways associated with synaptic transmission, consistent with neuronal function.
We further compared these metacell-specific DEGs with DEG results from two independent datasets. This comparison identified recurrent genes (e.g. BAG3, DNAJA4, STIP1, FKBP4) involved the heat-shock response, providing independent support for the increased enrichment of stress-response pathways observed in meta cells. In addition, synaptic genes (e.g. GRIN2B, GAD1) identified in the Columbia's independent dataset overlapped with the metacell-specific gene set, consistent with the stronger enrichment of synaptic pathways detected by the metacell approach (Supplementary Table S21).
Together, these results indicate that the additional DEGs recovered by metacell analysis are not a random collection of genes. Instead, they exhibit cell-type-relevant functional coherence and partial reproducibility across independent datasets. Although these analyses cannot exclude all possible effects of aggregation, they support the conclusion that the metacell approach improves sensitivity for detecting biologically relevant signals that are less readily identified by conventional single-cell analysis.
We have added these analyses and the corresponding results to the revised manuscript (Lines 165-176, Pages 7-8; Lines 487-490, Lines 493 - 496, Page 21).

3. Averaging log-normalized expression may introduce statistical bias
The authors construct metacells by averaging log-normalized expression values ("avg") rather than summing raw counts followed by normalization ("sum"). Because logarithmic transformation is nonlinear, averaging expression values in log space may artificially reduce within-metacell variance compared with aggregating raw counts before normalization.
Reduced variance could potentially inflate downstream statistical significance and contribute to the larger number of DEGs identified using the "avg" method. The authors should justify this methodological choice and demonstrate that their principal findings are robust to alternative metacell aggregation strategies.
Response #12: Contrary to the concern that averaging normalized expression values might artificially reduce variance and inflate differential expression, we observed the opposite trend in our data: the "sum" strategy consistently identified a larger number of DEGs than the "avg" strategy (Fig. 2A, Figure S1A-B; Lines 120-132, Page 6). Therefore, our empirical results do not support the hypothesis that the "avg"

approach inflates statistical significance through variance reduction.
We selected the "avg" approach because it produced results that were more consistent with the original single-cell analyses. Specifically, the differential expression patterns and downstream biological interpretations derived from metacells generated using the averaging strategy showed greater concordance with those obtained from the original single-cell data, suggesting that this approach better preserves the underlying biological signals while reducing technical sparsity (Supplementary Fig. 2A-C, Supplementary Table S2).
Our choice is also consistent with the recommendations of the developers of the SuperCell framework, who suggest that averaging normalized expression values is appropriate when metacells are used for downstream analyses such as visualization, cell annotation, co-expression analysis, gene regulatory network inference, and trajectory analysis, as this strategy avoids repeating upstream normalization and scaling procedures while preserving biologically meaningful relationships.

4. Limited cell-type resolution
The analyses rely on relatively broad cell-type clusters, many of which have been shown by recent single-cell studies to contain multiple transcriptionally distinct subpopulations. Since metacell construction depends on the initial clustering, the resulting co-expression and Bayesian networks may be sensitive to clustering resolution.
For example, would the conclusions differ if multiple microglial subpopulations were analyzed independently? Similarly, because PD is associated with selective degeneration of dopaminergic neurons and reactive expansion of certain glial populations, the authors should discuss how changes in cell abundance across disease states might influence metacell construction and downstream network inference.
Response #13: Our study intentionally performed metacell construction within the major annotated cell types rather than finer subpopulations for two reasons.
First, reliable co-expression and Bayesian network inference require a relatively large number of cells to generate robust metacells and stable correlation estimates. Further subdividing already relatively small cell populations would substantially reduce the number of cells available for metacell construction, thereby decreasing statistical power and increasing the risk of unstable network inference. However, the proposed metacell-based network analysis can be performed on any cell subpopulation with a sufficient number of cells in larger single-cell or single-nucleus RNA-seq datasets that will be generated for PD in the future.
Second, our primary objective was to identify cell-type-specific rather than cell-state-specific regulatory networks that are reproducible across multiple independent PD cohorts. Using major cell types provided a consistent annotation framework across datasets despite differences in sequencing depth, sample composition, and clustering resolution.
In our framework, metacells were constructed within each annotated cell type, thereby minimizing the direct influence of changes in global cell-type composition on metacell construction. Consequently, differences in the abundance of one cell type (e.g., dopaminergic neurons) do not directly affect the construction of metacells for another cell type (e.g., microglia or astrocytes).

5. Interpretation of causal mechanisms and therapeutic targets
A conceptual limitation of the study is that differential gene expression largely reflects disease-associated transcriptional changes, many of which are likely secondary consequences rather than primary drivers of neurodegeneration. Although the Bayesian framework attempts to infer causal relationships, the current study remains computational.
Accordingly, the concluding statement in the abstract that "The multiscale network models developed here lay down a foundation for the discovery of novel mechanisms and therapeutic targets for PD" appears stronger than is currently supported by the presented data. While the framework successfully prioritizes candidate regulators, no experimental evidence is provided demonstrating that these pathways contribute causally to dopaminergic neurodegeneration or represent tractable therapeutic targets. The authors should moderate these conclusions accordingly.
Response #14: We thank the reviewer for this important comment. We agree that disease-associated transcriptional changes may reflect primary drivers, downstream consequences, or compensatory responses, and that our Bayesian network provides computational evidence rather than experimental proof of causality. We have therefore moderated the language in the Abstract and revised the Discussion to emphasize the need for future perturbation studies in relevant in vitro and in vivo models to experimentally validate the predicted key drivers in PD and assess their therapeutic potential (Lines 624-626, Pages 26-27).

Specific comments
1. Figure 1: Please define all variables and abbreviations used in the schematic (e.g., μ, G, N and TF). The figure legend should more explicitly describe each analytical step.
Response #15: We thank the reviewer for this comment. We have updated the figure legend.

2. Please write out the full term for FET in the Figure S1 legend and provide references for the VIB, KAIST and Columbia datasets.
Response #16: We thank the reviewer for this comment. We have updated the figure legend.

3. In Figure S3B, μ(SC) appears to be missing.
Response #17: Due to high sparsity and small sample size, a co-expression network of the neuron Cluster 9 was unable to be constructed. Therefore, there is no correlation distribution of network in Cluster 9 in Figure S3B.

4. The manuscript uses PD GWAS loci to prioritize network modules. Because most GWAS variants reside in non-coding regions, the authors should clarify how variants were assigned to genes. Were loci mapped using nearest-gene assignment, eQTL colocalization, chromatin interaction data (Hi-C), TWAS, or another functional annotation strategy?
Response #18: In this study, we did not perform de novo mapping of GWAS variants to genes. Instead, we used a published, manually curated PD GWAS gene resource, in which GWAS loci had already been assigned to genes based on the methodology described by the original study. Therefore, gene assignment was determined by the curation strategy of the published database rather than by nearest-gene mapping or an in-house functional annotation pipeline.

5. The mathematical definition of the summary score is insufficiently described. Please provide the explicit equation used to combine DEG and GWAS enrichment statistics, including the weighting of each component.
Response #19: We have included the equation in the method section to describe our methodology (Lines 976-980, Page 42).

$$\text{Module Score}_m = \frac{-\log_{10}\left(P_{(adj,m)}^{\text{Upregulated DEG}}\right) + -\log_{10}\left(P_{(adj,m)}^{\text{Downregulated DEG}}\right) + -\log_{10}\left(P_{(adj,m)}^{\text{GWAS}}\right)}{3},$$

where $P_{(adj,m)}^{\text{Upregulated DEG}}$, $P_{(adj,m)}^{\text{Downregulated DEG}}$, and $P_{(adj,m)}^{\text{GWAS}}$are the adjusted P-values from the FET enrichment test for the DEGs and GWAS signatures, respectively, for module m. Higher scores indicate modules with stronger combined enrichment for disease associated transcriptional and genetic signals.

6. The manuscript identifies cluster c9 as a highly vulnerable neuronal population characterized by RIT2 expression. Recent human substantia nigra snRNA-seq studies have consistently identified SOX6+/AGTR1+ dopaminergic neurons as the population exhibiting the greatest vulnerability in PD. Does cluster c9 (or its dysregulated M3 module) significantly overlap with these established vulnerable neuronal signatures, or does RIT2 define an independent axis of selective vulnerability? Could the authors comment on this in the discussion?
Response #20: We thank the reviewer for raising this important point. Previous studies using midbrain datasets and mouse models have identified SOX6+/AGTR1+ dopaminergic neuronal populations as particularly vulnerable in PD. We examined these markers in our results, however, neither SOX6 nor AGTR1 was identified as a defining feature of cluster c9 or as a gene member of the dysregulated M3 module (Supplementary Table S23). Therefore, based on the current dataset, we do not find evidence that the RIT2-associated c9 population directly corresponds to the previously described SOX6+/AGTR1+ vulnerable dopaminergic population.
Importantly, our study directly profiles the human substantia nigra, a primary site of dopaminergic neuronal degeneration and a region central to PD pathology, providing a complementary disease-relevant context to findings derived from broader midbrain datasets and mouse models. The absence of the SOX6+/AGTR1+ signature in c9 therefore raises the possibility that RIT2 and the M3 network capture an additional axis of selective neuronal vulnerability that is particularly relevant to the human substantia nigra. Nevertheless, differences in species, anatomical sampling, cellular composition, and analytical resolution may contribute to the observed differences, and the lack of overlap alone does not establish that

these vulnerability mechanisms are independent. Further investigation, including direct integration with larger substantia nigra single-nucleus datasets and experimental characterization of RIT2-expressing neuronal populations, will be needed to determine the relationship between these vulnerability signatures. We have added this point to the Discussion (Lines 516-525, Page 22-23).

7. Construction of the Bayesian causal networks involved removing genes expressed in fewer than 40% of metacells (Tg = 0.4). While this filtering step may improve computational stability, it likely biases the resulting networks against lowly expressed transcription factors and other regulatory genes. The authors should report how many known PD-associated transcription factors were removed during this pruning step and discuss the resulting bias in their key driver analysis.
Response #21: We thank the reviewer for raising this important point. To assess whether the Tg = 0.4 pruning threshold disproportionately excluded transcriptional regulators, we quantified the removal of known transcription factors (TFs) and compared it with the removal of genes overall. Across all of the cell types, the percentages of removed TF, were similar to, and in some cases, slightly lower than, the overall percentage of genes removed during the pruning process (Supplementary Table S34), suggesting that the Tg = 0.4 filtering step did not preferentially deplete known PD-associated TFs relative to the background gene set. Nevertheless, we agree that an expression-based threshold inherently reduces representation of genes with sparse or low expression, including individual lowly expressed regulatory genes. Consequently, such regulators cannot be evaluated as potential key drivers if they are excluded before Bayesian network construction. We have therefore added the number and proportion of TFs removed by the filtering step and clarified this limitation in the revised manuscript (Lines 337-341, Page 14).

8. Approximately 70% of the predicted key drivers are described as novel. Validation based primarily on recovery of a single experimentally supported regulator (STMN2) provides only limited confidence in the broader set of newly identified candidates. Additional benchmarking using independent perturbation datasets, CRISPR screens, published regulatory networks or external validation datasets would substantially strengthen confidence in these predictions.
Response #22: We thank the reviewer for this important comment. We agree that validation based primarily on STMN2 provides limited support for the broader set of novel key drivers. Currently, large-scale CRISPR screening and perturbation datasets in PD-relevant neuronal models, particularly dopaminergic neurons, remain limited, restricting systematic independent validation of these predictions. We have acknowledged this limitation in the Discussion and emphasized that future studies using CRISPR screens and other functional perturbation approaches in relevant cellular and in vivo models will be important for validating the prioritized key drivers and establishing their roles in PD pathogenesis (Lines 619-626, Pages 27).

Editor Comments:
GigaScience has also published a number of relevant papers that may help contextualize your work. We have listed several recent examples below for your reference. Citing some of these articles, where appropriate in the Background or Discussion, would help frame your contribution within ongoing community efforts and strengthen the presentation of related methods and findings.
1. Bhattacharya, et al. Celltype transcriptomic modules reveal shared molecular mechanisms in Alzheimer's and Parkinson's d. GigaScience. 2026. https://doi.org/10.1093/gigascience/giag059/8689923.
2. Liu, et al. Multi-omics and high-spatial-resolution omics: deciphering complexity in neurological disorders. GigaScience. 2025. https://doi.org/10.1093/gigascience/giaf137/8371776.
Response #23: We thank the editor for bringing these relevant studies to our attention. We have incorporated the findings from Bhattacharya et al. into our discussion.

References
1. Wang Q, Wang M, Choi I, et al. Molecular profiling of human substantia nigra identifies diverse neuron types associated with vulnerability in Parkinson’s disease. Science advances. 2024;10(2):eadi8287.
2. Martirosyan A, Ansari R, Pestana F, et al. Unravelling cell type-specific responses to Parkinson’s Disease at single cell resolution. Molecular neurodegeneration. 2024;19(1):1-24.
3. Bhattacharya A, Fon EA, Dagher A, et al. Cell type transcriptomic modules reveal shared molecular mechanisms in Alzheimer’s and Parkinson’s disease. GigaScience. 2026;15:giag059.

Editorial Manager®

Cookie Preference Center

We use cookies which are necessary to make our site work. We may also use additional cookies to analyze, improve and personalize our content and your digital experience. For more information, see our Cookie Policy.

You may choose not to allow some types of cookies. However, blocking some types may impact your experience of our site and the services we are able to offer. See the different category headings below to find out more or change your settings.

Allow all

Manage Consent Preferences

Strictly Necessary Cookies

Always active

These cookies are necessary for the website to function and cannot be switched off in our systems. They are usually only set in response to actions made by you which amount to a request for services, such as setting your privacy preferences, logging in or filling in forms. You can set your browser to block or alert you about these cookies, but some parts of the site will not then work.

Functional Cookies

These cookies enable the website to provide enhanced functionality and personalisation. They may be set by us or by third party providers whose services we have added to our pages. If you do not allow these cookies then some or all of these services may not function properly.

Performance Cookies

These cookies allow us to count visits and traffic sources so we can measure and improve the performance of our site. They help us to know which pages are the most and least popular and see how visitors move around the site.

Targeting Cookies

These cookies may be set through our site by our advertising partners. They may be used by those companies to build a profile of your interests and show you relevant adverts on other sites. If you do not allow these cookies, you will experience less targeted advertising.

Cookie List

Clear

Apply Cancel

Consent Leg.Interest

Confirm my choices

Powe
