# Supplementary material for: Multiscale transcriptomic network models of Parkinson’s disease at the single-cell level
Source: Gigascience. 2026 Oct 6;15:giag096. doi: 10.1093/gigascience/giag096 (PMC13723071; doi:10.1093/gigascience/giag096)
Supplement: Supplementary file 2 [file giag096_giga-d-26-00192_original_submission.pdf]

GigaScience

Multiscale Transcriptomic Network Models of Parkinson’s Disease at the Single Cell Level

--Manuscript Draft--

**Manuscript Number:**	GIGA-D-26-00192
**Full Title:**	Multiscale Transcriptomic Network Models of Parkinson’s Disease at the Single Cell Level
**Article Type:**	Research
**Funding Information:**	National Institutes of Health (R01AG074010) — Prof. Bin Zhang<br>National Institutes of Health (R01AG085182) — Prof. Bin Zhang<br>Parkinson's Foundation (PF-RC-936279) — Dr. Zhenyu Yue<br>National Institutes of Health (S10OD026880) — Dr. Zhenyu Yue<br>National Institutes of Health (S10OD030463) — Dr. Zhenyu Yue
**Abstract:**	In Parkinson’s Disease (PD), multiple single nuclei RNA-sequencing (snRNA-seq) studies of the vulnerable brain regions have revealed alterations in brain cell populations and cell type specific transcriptomes. However, a systematic analysis of cell-type–resolved gene regulatory architecture in PD is lacking. Here, we develop an integrative, meta-cell based multiscale network analysis (MCMNA) of snRNA-seq data from the substantia nigra to systematically uncover molecular mechanisms and identify therapeutic targets for PD. MCMNA overcomes the inherent sparsity of single cell data by leveraging metacell-based aggregation, enabling construction of robust gene regulatory networks. Gene co-expression network analysis identifies cell-type specific gene modules associated with PD. Integration of meta-cell based differential gene expression and a Bayesian causal network systematically identifies putative driver genes and the top drivers. The multiscale network models developed here lay down a foundation for the discovery of novel mechanisms and therapeutic targets for PD.
**Corresponding Author:**	Bin Zhang, Ph.D.<br>Icahn School of Medicine at Mount Sinai<br>New York, New York UNITED STATES
**Corresponding Author Secondary Information:**	
**Corresponding Author's Institution:**	Icahn School of Medicine at Mount Sinai
**Corresponding Author's Secondary Institution:**	
**First Author:**	Chu Linh
**First Author Secondary Information:**	
**Order of Authors:**	Chu Linh<br>Gefei Yu<br>Xianxiao Zhou<br>Minghui Wang<br>Zhenyu Yue<br>Bin Zhang, Ph.D.
**Order of Authors Secondary Information:**	
**Additional Information:**	
**Question**	**Response**

Powered by Editorial Manager® and ProduXion Manager® from Aries Systems Corporation

Are you submitting this manuscript to a special series or article collection?	No
**Experimental design and statistics**<br><br>Full details of the experimental design and statistical methods used should be given in the Methods section, as detailed in our Minimum Standards Reporting Checklist. Information essential to interpreting the data presented should be made available in the figure legends.<br><br>Have you included all the information requested in your manuscript?	Yes
**Resources**<br><br>A description of all resources used, including antibodies, cell lines, animals and software tools, with enough information to allow them to be uniquely identified, should be included in the Methods section. Authors are strongly encouraged to cite Research Resource Identifiers (RRIDs) for antibodies, model organisms and tools, where possible.<br><br>Have you included the information requested as detailed in our Minimum Standards Reporting Checklist?	No
If not, please give reasons for any omissions below.<br><br>as follow-up to "**Resources**<br><br>A description of all resources used, including antibodies, cell lines, animals and software tools, with enough information to allow them to be uniquely identified, should be included in the Methods section. Authors are strongly encouraged to cite Research Resource Identifiers (RRIDs) for antibodies, model organisms and tools, where possible.	There is no experimental data in this study

Powered by Editorial Manager® and ProduXion Manager® from Aries Systems Corporation

Have you included the information requested as detailed in our Minimum Standards Reporting Checklist?<br><br>"	
**Availability of data and materials**<br><br>All datasets and code on which the conclusions of the paper rely must be either included in your submission or deposited in publicly available repositories (where available and ethically appropriate), referencing such data using a unique identifier in the references and in the "Availability of Data and Materials" section of your manuscript.<br><br>Have you have met the above requirement as detailed in our Minimum Standards Reporting Checklist?	Yes
GigaScience has policies and guidelines in place for the use of generative AI-writing tools such as ChatGPT. If you have used such writing tools to assist with writing the manuscript this must be declared and cited in the text. Authors should not list AI-writing tools and other AI-assisted technologies as an author or co-author and should acknowledge that they are fully responsible for text generated or refined by AI-writing tools.<p><br><br>A summary of use (particularly in the introduction or among methods) needs to be included at the end of the paper, and the outputs should also be included as a supplementary file hosted in GigaDB or other open repositories. Please read our guidelines for more information. <p><br><br>By submitting to GigaScience, you are aware of the journal's AI-writing tools policy, and if you have declared use of such tools below, you have acknowledged this where appropriate in your manuscript and have made a summary of use and outputs available. <p><br><b>AI-assisted writing tools have been	No

used in the preparation of this manuscript?	

Powered by Editorial Manager® and ProduXion Manager® from Aries Systems Corporation

Manuscript

Click here to access/download;Manuscript;PD_metacell-networks_v5_BZ2.docx

Multiscale Transcriptomic Network Models of Parkinson's Disease at the Single Cell Level

Linh Chu[1,2,3,4], Gefei Yu[1,2,5], Xianxiao Zhou[1,2,5], Minghui Wang[1,2,5], Zhenyu Yue[3,6,7], and Bin Zhang[1,2,4,5,8]*

[1]Department of Genetics and Genomic Sciences, Icahn School of Medicine at Mount Sinai, 1425 Madison Avenue, NY 10029, USA

[2]Mount Sinai Center for Transformative Disease Modeling, Icahn School of Medicine at Mount Sinai, 1470 Madison Avenue, NY 10029, USA

[3]Department of Neurology, Icahn School of Medicine at Mount Sinai, 1425 Madison Avenue, NY 10029, USA

[4]Department of Pharmacological Sciences, Icahn School of Medicine at Mount Sinai, 1425 Madison Avenue, NY 10029, USA

[5]Icahn Institute of Genomics, Icahn School of Medicine at Mount Sinai, 1425 Madison Avenue, NY 10029, USA.

[6]Nash Family Department of Neuroscience, Icahn School of Medicine at Mount Sinai, 1470 Madison Avenue, NY 10029, USA

[7]Friedman Brain Institute, Icahn School of Medicine at Mount Sinai, 1470 Madison Avenue, NY 10029, USA

[8]Department of Artificial Intelligence & Human Health, Icahn School of Medicine at Mount Sinai, 1470 Madison Avenue, NY 10029, USA

Corresponding author:
Bin Zhang, PhD
Professor, Department of Genetics & Genomic Sciences, Icahn Institute of Genomics
Icahn School of Medicine at Mount Sinai

1425 Madison Avenue, Room 3-43

New York, NY 10029

(O) 212-659-1726

Email: bin.zhang@mssm.edu

SUMMARY

In Parkinson's Disease (PD), multiple single nuclei RNA-sequencing (snRNA-seq) studies of the vulnerable brain regions have revealed alterations in brain cell populations and cell type specific transcriptomes. However, a systematic analysis of cell-type–resolved gene regulatory architecture in PD is lacking. Here, we develop an integrative, meta-cell based multiscale network analysis (MCMNA) of snRNA-seq data from the substantia nigra to systematically uncover molecular mechanisms and identify therapeutic targets for PD. MCMNA overcomes the inherent sparsity of single cell data by leveraging metacell-based aggregation, enabling construction of robust gene regulatory networks. Gene co-expression network analysis identifies cell-type specific gene modules associated with PD. Integration of meta-cell based differential gene expression and a Bayesian causal network systematically identifies putative driver genes and the top drivers. The multiscale network models developed here lay down a foundation for the discovery of novel mechanisms and therapeutic targets for PD.

Keywords: single-nucleus RNA-sequencing, Parkinson's disease, cell sparsity, GWAS, Metacells, Multiscale Embedded Gene Co-expression Network Analysis, Bayesian Network, key drivers

INTRODUCTION

Parkinson's disease (PD) is the second most prevalent neurodegenerative disorder, with both motor and non-motor symptoms[1,2]. The pathology of PD is characterized by the progressive loss of dopaminergic (DA) neurons in the substantial nigra (SN) and accumulation of Lewy bodies (LB), which contain abnormally α-synuclein protein aggregation[3,4]. Rare variants in over 20 genes account for 3-5% of PD cases in most populations, most of which are highly penetrant, and often known to be causal genes[5]. To date, genome-wide association studies (GWAS) have identified 90 genetic risk variants, which collectively account for 16-36% of the heritable risk of non-monogenic PD[6]. Given that most PD patients have no familial history or known PD mutations, there remains a critical gap in understanding how molecular signatures relate to disease mechanisms and therapeutic strategies in idiopathic PD. While the loss of DA neurons is a hallmark of the disease, microglial activation was first reported in PD brains in 1988[7], and has been consistently supported by *in vivo* and *in vitro* studies[8-11]. In addition, astrocyte reactivity has been prominently observed in the SN of PD patients, with research highlighting both neuroprotective and neurotoxic roles such as production of oxidative and nitrosative stress, inflammation, and contribution to the blood-brain barrier[12-15]. Recently, studies have indicated oligodendrocyte dysfunction in PD, with changes pointing to altered inflammatory responses, protein folding stress, and myelination abnormalities[16-18].

Gene regulatory network (GRN) uncovers new pathways and gene targets in biological processes in human disease. Previously, our labs integrated a multiscale transcriptomic network analysis of PD SN samples by assembling the gene expression profiles from eight published bulk RNAseq studies to systematically identify intrinsic network structures and key regulators such as *STMN2* in PD[19]. Bulk tissue datasets do not have cell-type level granularity, thus limiting the understanding of complex signaling across and within cell populations. Nowadays, single-cell/Single-nucleus RNA sequencing (sc/sn-RNAseq) has emerged as a promising tool to unveil cell-level signaling pathways associated with neurodegenerative diseases including PD. We and others, have profiled single nuclei of human SN from PD and healthy

control, identifying changes in gene expression and new biological pathways at single cell levels[20-25]. Although these studies accumulate quickly and become publicly accessible, little progress has been made regarding cell type specific GRN analysis for PD.

The challenges to construct single cell GRN inference mainly stem from the high sparsity in gene expression profile (large percentage of missing values or zeros) in snRNA-seq data. Many model-based imputation methods have been developed, yet the performance of these methods varies across different datasets[26]. Alternatively, researchers have adopted the concept of "Metacells"[27] and developed several metacell algorithms[27-30] to not only compress data but also enhance the signal-to-noise ratio in expression matrices. This results in more accurate clustering, improved annotation, and more reliable identification of differentially expressed genes (DEGs). Despite the potential, these metacell algorithms have yet to be applied to gene network analysis in PD[31].

In this study, we develop an integrative metacell-based gene co-expression and causal network analysis framework and applied it to a recently published PD snRNAseq dataset in the human SN from 23 PD and 9 healthy controls[20]. By adopting a systematic and data-driven approach in both co-expression and causal networks, we identify neuron-specific and glia-specific subnetworks that are dysregulated in PD. These cell type specific networks not only reveal putative key drivers for PD but also shed a light on the molecular mechanisms of PD. Notably, the top putative drivers include known targets of PD such as *UCHL1*, *NSF*, and *PREPL* in neurons, as well as novel candidate genes, such as *CADPS2*, *APBB1IP, HSP90AA1* in neurons or glial cells. In addition, PD driver genes (*STMN2* and *RALYL*) identified in our previously bulk RNAseq network analysis are also found to be key drivers in the respective neuronal single cell networks. Altogether, our study provides a framework for integrating diverse, cell type specific networks to pinpoint putative drivers of PD.

RESULTS

Integrative network biology framework for decoding molecular mechanisms of Parkinson's Disease at the single cell level

To systematically model single cell transcriptomic data in PD, we develop an integrative, meta-cell based multiscale network analysis (MCMNA) framework that includes metacell construction and characterization, co-expression network analysis, causal network inference, and key driver analysis (**Fig. 1**). In brief, we first group single cells into metacells – a group of cells with highly similar gene expression profiles (**Table 1**). To preserve the cell type annotation and phenotype information from snRNA-seq data, the metacell analysis is performed for each condition in each cell cluster. Single cell profiles within each metacell are aggregated to create metacell gene expression profile. Then, differentially expressed genes (DEGs) between the disease and control are identified, and functional enrichment analysis is performed on the DEGs to identify dysregulated pathways in PD (**Fig. 1A**). Next, we construct metacell-based co-expression analysis using Multiscale Embedded Gene co-Expression Network Analysis (MEGENA)[32] for each cell type to identify cell type specific co-expression modules (groups of highly co-expressed genes) (**Fig. 1B**). In parallel, we construct the global Bayesian probabilistic causal network (BN)[33,34] and perform key driver analysis (KDA)[35] to predict potential key regulators, termed key drivers (KDs) of PD (**Fig. 1C**). A unique feature of MCMNA is that it addresses the sparsity of single cell gene expression data and increase the robustness of single cell network by the generation of metacells, construction of biologically meaningful correlation (BMC) matrix for co-expression network, and additional pruning of data matrix for causal network. Additionally, network models based on both PD and control allow us to capture early changes driving PD as such signals may not change across various disease stages.

In the remainder of the manuscript, we apply MCMNA to a single-nucleus RNA sequencing (snRNA-seq) dataset derived from substantia nigra (SN) samples of 23 idiopathic PD cases and 9 controls,

with an average age of 81, as described in a previous publication.[20]. The study profiled a total of 315,867 high quality nuclei and identified 12 distinct cell clusters.

Metacells uncover cell type- specific gene expression enrichment between PD and control

At each cell cluster and condition (PD or control), single-cell profiles within each metacell are aggregated to generate metacell gene expression profiles, either by averaging the log-normalized single-cell expression data (average - "avg") or by summing the raw single-cell values followed by log normalization (sum). Additionally, the method allows for the inclusion of metacell size (the number of single cells in each metacell) as a weighting factor (weighted) or omitting it (unweighted). To explore global transcriptomic changes using metacell method, we first examine the number of DEGs in each cell cluster (at least 1.1-fold change (FC) and false discovery rate (adjusted p value less than 0.05) and calculate the percentage of preservation compared to the single-cell method. The average method demonstrates higher preservation compared to the sum method across the majority of the clusters (**Fig. 2A**, Supplementary **Fig. S1A-S1B**). While the number of DEGs is reduced in metacells compared to the single cell method, the number of conserved markers increases, potentially reflecting enhanced cell cluster specificity and reduced noise in metacells (Supplementary **Fig. S1C**). However, this observation has not been formally tested, and further studies are needed to confirm it.

While weighted aggregation can incorporate cell-level weight, it substantially increases the data size and computation time, without consistently improving downstream analyses such as differential expression, pathway enrichment or correlation distribution (data not shown). Therefore, the unweighted method provides a good balance between efficiency and accuracy. Distinct gene signatures of five major brain cell types (e.g. neurons, microglia, astrocytes, oligodendrocytes and oligodendrocytes progenitor cells (OPCs)) are highlighted using the selected metacell method (average and unweighted) (**Fig. 2B**, **Supplementary Fig. S1E**, **Table 2, Supplementary Table S1**). Next, we compare our DEG signatures with other published snRNA-seq of human SN from three independent cohorts in PD[21,22,25]. We reprocess the original datasets using our own analytical pipeline to identify

cell clusters (**Table S2-8**, **Supplementary Fig. S1F-G, Fig. S1I-J, Fig. S1L-M**). We then assess the overlap of DEG signatures in each cell type using Fisher's exact test (FET). The results show that, for three datasets, there is a decrease in corrected p-value but an increase in fold enrichment, indicating more reduced statistical preservation strength but greater specificity in the preservation of DEG signatures across major cell cluster between the two datasets in metacells (**Table S9-11**, **Supplementary Fig. S1H, Supplementary Fig. S1K, Supplementary Fig. S1N**). **Metacells reveal common and unique cell type- specific biological pathways between PD and control**

Functional enrichment analysis is performed on the DEG signatures from both metacell and single cell methods. Using selected conditions (average and unweighted), we identify common and unique biological pathways. We observe that 10-30% and 0-60% shared pathways in major cell types from up-regulated and down-regulated DEGs, respectively (**Supplementary Table S12-14, Supplementary Figure S2A-B**). Similar to previously reported results, upregulated genes across cell types are involved in heat stress, Hsf1 activation, protein folding, and cell migration pathway. In contrast, downregulated genes are associated with nervous system development, synaptic transmission, neurotransmitter transports across cell types, and glial cell development in glial cells (**Figure 2C**). Many pathways unique to the single cell and metacell methods are also detected (Supplementary **Table S15-16**, Supplementary **Figure S2A-B**). Positive correlation of both up-regulated and down-regulated pathways across cell types indicates highly conserved pathways between the two methods. Additionally, several pathways showing significant difference in enrichments in the single cell and metacell based DEG signatures are highly relevant in PD biological pathways. These include protein folding in glial cells, autophagy in astrocytes, mitochondrial process in microglia and astrocytes, cytokine production in microglia, vasculature development, neurotransmitter release, calcium transport in neurons (**Figure 2D-E**).

A novel framework to address single cell sparsity in co-expression network

To understand the interactions among genes, we construct gene co-expression networks to identify gene modules using MEGENA under the MCMNA framework (Supplementary **Figure S3A**). While metacell improves the gene-gene correlation, as noted in previous studies[27-30], gene-gene correlation still suffers from data sparsity. Therefore, zero counts are removed for each gene pair before calculating Spearman correlation coefficients (SCCs), leading to an increase in overall correlation distribution in metacells compared to single cell (Supplementary **Figure S3B**). To address inherent noise in sc/snRNA-seq data observed by the drift in the mean signed SCCs across cell types, we propose a framework to create a Biologically Meaningful Correlation (BMC) matrix at the optimal threshold of number of cells and genes (Supplementary **Figure S3A, C**). We performe FET analysis between *STMN2*-related genes and *STMN2* perturbation DEG signatures in PD mouse SN[19] across all clusters and three neuronal clusters. Compared to the metacell FDR only and single cell matrix only, BMC approach yields higher -log10(FDR) and fold enrichment in the MC-average comparison, and higher fold enrichment in the MC-sum comparison (**Figure 3A, Supplementary Table S17**). These suggest that BMC matrix indeed reduces noise the weak positive correlation, resulting in a more consistent gene subset aligned with the STMN2 perturbation. Averaging log-normalized expression values indicate more statistically significant enrichment and is therefore used for downstream analysis (**Figure 3A**, Supplementary **Table S17**). Optimized BMC matrix is then subjected to MEGENA. Additionally, this method enables the construction of a gene co-expression network for the small neuron cluster 9 (c9), which was not possible due to high sparsity and small sample size. No MEGENA modules are found in the fibroblast cluster 10 and the T cells cluster 11.

Metacell-based co-expressed networks reveal cell-type-specific functional gene modules

In total, we identify 1,057 significant gene modules (FDR adjusted P values less than 0.05) in all the cell types, and 833 modules in 5 major cell types (**Table 3, Supplementary Table S18**). To annotate biological functions associated with each module, functional enrichment analysis is performed. To prioritize gene modules associated with PD, a summary score is calculated based on the following FET

enrichment tests: 1) enrichment for the DEG signatures between PD and control (Upregulated (UP) and Downregulated (DN) DEGs), and 2) enrichment for the PD genetic risk variants (PD GWAS catalogue) (Supplementary **Table S19**)[37]. Given that the central nervous system consists of various cell types, each with distinct roles in PD pathogenesis and progression, we focus on the major brain cell types: neurons (cluster 6 (c6), cluster 7 (c7), and cluster 9 (c9), astrocytes (cluster 2 (c2)), microglia (cluster 1 (c1)), oligodendrocyte precursor cells (OPCs) (cluster 5 (c5)), and oligodendrocytes (cluster 0 (c0) and cluster 3 (c3)). The findings of MEGENA modules reveal cell-type-specific functional modules characterized by highly coordinated molecular dysregulations in PD (Supplementary **Table S20-22**). The top 10 PD associated gene modules within each major cell type are shown in **Figure 3B**. Among the top 50 ranked modules, there are 17 neuronal modules, of which 14 originate from the neuron cluster c9. The number of top ranked modules from other glial cells are as follows: oligodendrocytes (c0: 5, c3: 13), microglia (c1: 4), astrocytes (c2: 7), and OPC (c5: 4).

We validated the biological coherence of our network modules in another snRNAseq dataset from the SN of PD and normal subjects (termed Ma dataset) at the cell type specific level[25]. As shown in **Supplementary Table S23-24** based on a global approach across all identified modules, module preservation is highest in oligodendrocytes (~30-50%), followed by microglia/astrocytes (~20-30%), and neurons (~5-20%) (**Supplementary Figure S4A**). Additionally, we examined the conservation of the PD-related modules (i.e. modules enriched for up-regulated, down-regulated, and/or PD GWAS genes) in the two datasets. PD-related modules showed higher and more significant preservation across all cell clusters (**Supplementary Table S25-26**). Similar to the global approach, module preservation is strongest in oligodendrocytes (35- 90%), followed by astrocytes/microglia (~60-90%), and neurons (~0-30%). Notably, in our dataset, the PD-related modules of neuron cluster C9 showed greater preservation in the corresponding neuron cluster C4 from the Ma dataset than the PD-related modules identified in the other neuron cluster C6 of our dataset showed in C4 of the Ma dataset (**Supplementary Figure S4B**).

Neuronal gene modules are most dysregulated in PD

Among all the modules, the module M3 in the co-expression network from c9 is ranked as the top for its most significant enrichment of the down-regulated DEGs using Fisher's Exact Test (FET) (multiple testing corrected p or false discovery rate (FDR) = 1.7E-200, 2.3-fold enrichment (FE)) and GWAS genes (FDR = 8.1E-04, 2-FE) (**Figure 3B**, **Supplementary Table S20**). The global network of c9 shows a highly hierarchical structure, suggesting strong modular organization and multiscale biological processes (**Figure 3C**). Seven child modules of the module M3 in c9's co-expression network are highly enriched for down-regulated DEGs such as M27─►M70─►M100 (M27: FDR = 5.8E-18, 2.3 FE), M28─►M74 (M28: FDR = 1.1E-20, 2.4 FE), M29─►M79 (M29: FDR = 9.3E-24, 2.2 FE), M32─►M82─►M81 (M32: FDR = 2.2 E-16, 2.4 FE), M30 (FDR = 9.5 E-11, 2.8 FE), M31 (FDR = 5.8E-18, 2.5 FE), M33 (FDR = 1.2 E-11, 2.7 FE) (**Figure 3E, Supplementary Table S21**). M3 is associated with synapse organization (FDR p= 7.9E-24). All the other child modules of M3 are also enriched for down-regulated DEGs with synapse-related functions such as synapse assembly, regulation of neuron projection development, regulation of trans synaptic signaling, neuronal system, receptor localization to synapse and dendrite development (**Figure 3F, Supplementary Table S20, Supplementary Table S22**). These child modules of M3 are also among the top 50 modules from all the cell clusters and among the top 20 modules from c9 (**Figure 3D**, **Supplementary Table S20**). Interestingly, other branches of c9 separated from M3 module are not enriched for the downregulated DEGs. These modules are associated with pathways such as ion transport, metabolic process, and transcription, suggesting that only a small subset of c9 neurons appears to adopt the disease phenotype.

Apart from the neuron cluster c9, the modules in cluster c6 are less enriched for DEGs. In c6, a few top-ranked modules are associated with upregulated DEGs, such as M5 (FDR = 1.7E-08, 4.3 FE), M13 (FDR = 1.7E-08 , 10 FE), M126 (FDR = 1.7E-08, 45 FE), M8 (FDR = 3.73E-05, 4.6 FE), followed by modules are associated with downregulated DEGs such as M92 (FDR = 1.7E-05, 16.9 FE), M124 (FDR = 1.7, 36.1 FE) (**Supplementary Figure S5A, Supplementary Table S21**). These are also linked

to pathways involved in synapse organization and axon development (**Figure S5B, Table S20, Supplementary Table S22**). Interestingly, no module in the network from the neuron cluster c7 is enriched for DEGs (**Supplementary Table S20**), though many modules have meaningful biological functions (**Supplementary Figure S5C**). The global network of c7 also shows a more compact structure (**Supplementary Figure S5D**). This implies that the dysregulated genes in c7 are not well coordinated.

Glial gene modules associated with PD are involved in development process, metabolic processes and immune responses

In addition to neuronal modules, we also identify numerous glial modules in oligodendrocytes, microglia, astrocytes, and OPCs. Oligodendrocytes represent the largest population in the dataset, and consequently, the single-cell co-expression networks of cluster 0 (c0) and cluster 3 (c3) are among the largest, containing many genes and gene interactions. Among the top 50 ranked modules overall of c3, M10─►M41─►M164 emerged as one branch of particular interest. M10 is a parent module of M41 and M42, with its members enriched with both up (FDR = 2.9E-05, 4.3 FE), and down-regulated DEGs (FDR = 5.9E-34, 10.3 FE), and involved in cell polarity (FDR = 0.005), mitochondria organization (FDR p = 0.05), vesicle organization (FDR = 0.05) (**Figure 4A-B**, **Supplementary Figure S6A**). M10 has two branches, M41─►M164 and M42─►M166. One is the M41─►M164 branch in which both modules enriched with downregulated DEG (M41: FDR = 1.4E-43, 16 FE; M164: FDR=1E-44, 17 FE), and their members are associated with autophagy of mitochondria (M41: FDR = 0.04; M164: FDR = 0.04) (**Supplementary Figure S6B**). Another is M42─►M166 with both modules enriched with up-regulated DEGs (M42: FDR = 3.7E-11, 12 FE; M166: FDR = 1.7E-06, 11 FE) and associated with apoptosis pathway (M42: FDR = 0.006; M166: FDR = 0.001) (**Supplementary Fig. S6C**). Some branches in the network of c3 are also associated with protein folding (M8─►M38─►M159), and oligodendrocyte differentiation (M5─►M20─►M111), highlighting the diverse pathways underlying oligodendrocyte functions. The network from the cell cluster c0 has a slightly smaller number of modules

ranked at the top 50 modules from all the cell clusters and the most significant module M3 is enriched for both up-regulated (FDR = 2.2E-04, 2.1 FE), down-regulated DEGs (FDR = 1E-15, 2.3 FE), and is associated with actin filament organization (FDR = 1.5E-04). Several child modules of M3 from c0 are also enriched for DEGs, including M6─►M67 (involved in nervous system development), M8 (involved in metabolic process), and M17 (involved in nervous system development) (**Supplementary Figure S6E-F**).

We also identify several significant modules in the astrocyte cluster c2. Among those, the most significant module M3 is enriched for the down-regulated DEGs (FDR = 2.1E-38, 3.6 FE) and the GWAS genes (FDR = 4E-04, 1.9 FE) (**Figure 4C, Supplementary Figure S7A**). Some of M3's child modules are functionally important and enriched for down-regulated genes. The M3─►M59 branch is highly associated with regulation of small GTPase mediate signaling transduction (M3: FDR = 2.1E-10; M59: FDR = 0.004) (**Figure 4D**) while the M3─►M64 branch is associated with synapse assembly (M64: FDR = 5.1E-03) (**Figure 4E**). On the other hand, the modules M6 and M72 are highly enriched for upregulated DEGs (M6: FDR = 5.8E-14, 21.3 FE; M72: FDR =2.35E-08, 18 FE) (**Supplementary Figure S7B**), and linked to inclusion body assembly (M6: FDR = 1.7E-04), and regulation of endothelial cell chemotaxis (FDR = 2.5E-03) (**Supplementary Figure S7C**). These findings demonstrate the role of astrocytes in modulating and supporting neuronal and synaptic functions.

Furthermore, we identify several microglial modules highly associated with PD. The top microglia module M15, which is associated with mononuclear cell differentiation, is enriched for down-regulated genes (FDR = 4.8E-09, 6.6 FE) (**Figure 4F**, **Supplementary Figure S7C**, **Figure 4G**). Additionally, we also observe a few branches associated with GTPase activity. While the branch of M16─►M70─►M156 is associated with down-regulated DEGs (M16: FDR = 3.9E-08, 6.1 FE; M70: FDR = 1.7E-07, 6.3 FE; M156: FDR = 9.7E-08, 6.7 FE) (**Supplementary Figure S7D**), the branch of M12─►M55 is associated with up-regulated DEGs (M12: FDR = 3.6E-04, 8.7 FE; M55: FDR = 5.4-04, 13.8 FE) (**Supplementary Figure S7E**). The presence of co-expressed genes with the roles in

innate immune signaling and GTPase activity might suggest the role of certain gene modules in translating or facilitating myonuclear cells to microglia into more reactive states.

Last but not least, although the modules in the network of OPC are less enriched for DEGs compared to other cell types, several modules are enriched for down-regulated DEGs (**Supplementary Figure S7F**) and are associated with glial cell development, nervous system development, and synaptic transmission (**Supplementary Figure S7G**). Interestingly, these down-regulated modules are the daughter modules of M2 (FDR = 0.01, 1.3 FE) with diverse functions, including the branch M6─►M57 (M6: FDR =2.9E-07, 6.4 FE; M57 – cFET = 5.3E-07, 7.3 FE) involved in regulation of trans-synaptic signaling (M6: FDR = 6.1E-03, M57: FDR= 1.4E-03), the branch M8─►M67 (M8: FDR = 5.3E-03, 5.3 FE; M67: FDR = 3.8E-04, 8.1 FE) involved in glial cell differentiation (M8: FDR = 1.8E-04; M67: FDR = 1.4E-06). These findings suggest that certain components of OPCs support both neuronal and glial functions, which have been understudied in the field.

Metacell-based Bayesian Networks predict well-known and novel key drivers

While the gene co-expression network analysis identifies coherent gene modules associated with PD in each major brain cell type, the findings remain inherently associative and don't establish causal relationships among genes. To move beyond correlation and uncover potential regulatory mechanisms, we construct Bayesian probabilistic causal networks (BNs) to identify gene-gene causal relationships. Given the sparsity existing in the datasets, we further remove additional genes and metacells, in a process of pruning to find the optimal cutoff (**Figure 1C**). After that, we construct a global BN for each cell cluster, followed by key driver analysis (KDA) to predict key regulators for each cell cluster[38,39] (**Figure 5A-B**, **Figure S8A**, **Figure 6A-B**, **Supplementary Figure S9A**, **Supplementary Figure SC, Supplementary Figure S9E**). We identify a total of 151 key drivers (138 unique genes) and interestingly several drivers are detected in multiple cell clusters (**Table 4, Supplementary Table S27**). We also estimate the relevance of each gene to PD as the cooccurrence of a given gene and PD in PubMed. Among 138 unique key drivers, 43 (~30%) have occurrence above or equal to 5 (**Figure 5C**). Then, we classify the key drivers into two

categories, 1) well-studied key drivers (cooccurrence ≥ 5), and 2) novel key drivers (cooccurrence <5) (**Table 4, Supplementary Table S27**).

Novel and known key drivers of neuronal clusters converge on synaptic regulation

The key drivers of neuronal clusters c7 and c9 are among the most significant drivers across all the cell cluster. The most significant driver is *CADPS2* in c9 (KDA FDR = 1.8E-39; 1.8 FE), which is the most downregulated in c9 (Fold Change (FC) = 0.48, FDR = 5.2E-35). In neurons, *CADPS2* encodes for a calcium binding protein that regulates synaptic exocytosis and dense core vesicles[40,41], and has been previously studied in dopamine release[42]. Also *CADPS2* is a novel small neuronal cell marker in idiopathic PD[43]. *CADPS2* is also a member of the gene modules including M3, M32, and M81, which are associated with synapse organization, and neural system pathways (**Supplementary Table S18, Supplementary Table S20**). The BN in c9 also indicates *CADPS2* as an upstream driver of 4 down-regulated key drivers such as *CDH18*, *RYR2*, *UNC13C*, and *FSTL5*, three of which (*CDH18*, *RYR2*, *UNC13C*) play roles in calcium-depending synaptic cell-cell adhesion[44,45], release of calcium ions[46], exocytosis[47], respectively (**Figure 5D, Supplementary Table S28**). Interestingly, the 2-layer subnetwork centered around *CAPDS2* (111 genes) is also involved in and modulation of chemical synaptic transmission (FDR = 2E-04), regulation of trans-synaptic signaling (FDR=2.1E-03), synaptic vesicle docking (FDR=3.2E-03) (**Supplementary Table S29**). These genes form a coordinated subnetwork to modulate calcium-mediated process which is essential for neuronal communication and synaptic function.

RALYL, previously identified as a down-regulated key regulator from the network analysis of bulk transcriptomic data in PD[19], also emerges as a key driver in c9 (KDA: FDR = 2.1E-09; 2.2 FE; Expression: 0.6 FC, FDR = 9.8E-23) (**Supplementary Figure S8B, Supplementary Table S28)**. *RALYL* is a member of M3, M29, and M74 (**Supplementary Table S18**). The subnetwork around *RALYL* is associated with metabolic pathways such as cell surface receptor protein tyrosine kinase activity (FDR p = 3.7E-03), cellular response to histamine (FDR = 1E-02), and hypoxia (FDR = 1E-02) (**Supplementary Table S29**). Thirty of

the 36 drivers in c9, which are downregulated in PD, are novel as only six genes have been associated with PD or studied in PD (**Table 4, Supplementary Table S27**).

In contrast to c9, twenty four of the 54 drivers in c7 have well-known functions associated with PD (24/54 genes). For example, a major driver *UCHL1* (Ubiquitin C-Terminal Hydroxylase) is implicated in the early onset of family and sporadic PD[5], influencing α-synuclein degradation and contributing to PD susceptibility[48]. In cluster c7, *UCHL1* is significantly downregulated and enriched in cluster 7 (KDA: FDR = 8E-06, FE=2; Expression: FC=0.8, FDR = 5.4E-22). BN results indicate *UCHL1* is the downstream effector of two key drivers, *STMN2* (previously identified as a key driver in bulk transcriptomic data) and *OAT* (**Figure 5E, Supplementary Table S28**). The subnetwork around *UCHL1* is involved in regulation of high voltage-gated calcium channel activity (FDR = 6.3E-02), insulin secretion (FDR = 6.3E-02), and membrane invagination (FDR p = 6.3E-02) (**Supplementary Table S29**).

Besides *UCHL1*, we also identify other GWAS-associated gene *NSF* as a down-regulated key driver in c7 (KDA: FDR = 5.2E-34, FE =1.9; Expression: FC=07, FDR = 1.8E-56). *NSF* encodes N-ethylmaleimide-sensitive fusion protein, which plays an essential role in intracellular vesicle transport, synaptic transmission and membrane fusion[49], interacting with α-synuclein via SNARE complex protein[50], with diminishing expression level in a MPTP mouse model of PD[51] and lower blood and brain level in PD[52]. Additionally, *NSF* also directly regulates other key drivers such as *ATP1B1*, *SV2B*, and *STXBP1*, which play a role in regulating ATPase Na+/K+ pump[53], vesicle trafficking and exocytosis[54], and neurotransmitter release[55], respectively (**Supplementary Figure S8F, Supplementary Table S28**). The subnetwork centered around *NSF* is also involved in protein localization to plasma membrane (FDR = 9.4E-06), neuron projection development (FDR = 1.6E-04) and synaptic vesicle membrane organization (FDR=5.02E-03) (**Table S29**), highlighting the functional context of the PD GWAS gene.

STMN2, previously identified as regulator [19], is also a key driver in c7 (FDR = 8E-09, 1.9 FE) and downregulated (FC=0.8, FDR = 7.5E-15) (**Figure 5F, Supplementary Table S28**). *STMN2 i*s known to regulate microtube dynamic, axon formation and neurite outgrowth during development [56-58]. The

subnetwork around *STMN2* also indicates the enrichment of pathways such as regulation of microtube depolymerization (FDR = 3.4E-02), regulation of macroautophagy (FDR=3.9E-02), and polymeric cytoskeletal fiber (FDR p = 9.5E-02) (**Table S29**). The subnetworks around *STMN2* are enriched for the knock-down signature of *STMN2* in mouse SN[19], validating the accuracy of our predicted metacell-based causal network (**Figure 5G**).

Additionally, we also identify *PREPL* as a key driver (KDA: FDR = 1.7E-18, FE=2.2; Expression: FC=0.8, FDR = 3.0E-23). *PREPL* encodes for propyl endopeptidase-like enzyme and has recently been implicated in neuronal functions, including protein trafficking, endocytosis, synaptic activity[59,60], and the facilitation of α-synuclein aggregation seeding[61], highlighting its relevance as a candidate for further investigation. The subnetwork around *PREPL* (161 genes) suggests *PREPL* as downstream of *ATP1B1*, and upstream of many other key drivers such as *STXBP1*, *SLC9A6*, *RTN1*, *NPTN*, *TSPYL4*, *SV2A*, *SERINC1* (**Figure 5H, Supplementary Table S28**). This network is involved in positive regulation of synaptic transmission (FDR = 2.6E-03), negative formation of amyloid beta formation (FDR = 2.6E-03), and intracellular monoatomic cation homeostasis (FDR = 2.6E-03) (**Table S29**).

In cluster c6, we identify the PD GWAS gene, *MDGA2,* as an up-regulated key driver (KDA: FDR = 2.1E-08, FE=3.3; Expression: FC=1.2, FDR = 2.1E-05). *MDGA2*, which encodes a regulator of axonal growth[62], contains a SNP that has been potentially linked to earlier onset of familial Parkinson's disease[63]. The subnetwork around *MDGA2* (68 genes), which is involved in chemical synaptic transmission (FDR = 2.7E-02) and synapse organization (FDR p = 3.5E-02), shows that *MDGA2* directly regulates another key driver *NPAS3* (**Supplementary Figure S6E, Table S28-29**).

Emerging key drivers in glial cells suggest novel causal roles in homeostasis, metabolic responses, immunity, and heat shock response

In oligodendrocytes, *MOBP* or myelin-associated oligodendrocyte basic protein, is identified as a key driver. *MOBP* plays a role in myelination and structural constituent of myelin sheath[64,65]. This gene is the top driver

with the highest network significance and downregulated in c3 (KDA: FDR p = 9.2E-42, 5.5 FE; 0.8 FC, FDR = 3E-59) and c0 (KDA: FDR = 2.7E-15, 6.05 FE; Expression: 0.9 FC, FDR p = 6.3E-100). The subnetworks around *MOBP* in c3 and c0 indicate *MOBP* as an upstream of key drivers such as *NEAT1*, *COL4A5*, *CARNS1*, *LINC01505* in c3 (**Figure 6B**), and *CARNS1* in c0 (**Figure S9B**). In c3, the subnetwork around *MOBP* (307 genes) are associated with regulation of dendritic spine development (p = 2.7E-04), myelination (p = 9.9E-04), neuronal cell-cell adhesion (p = 1.1E-03), and protein localization to synapse (p = 2.1E-03) while in c0, the subnetwork (61 genes) is associated with transition metal ion transport (p = 1.1E-04), substantia nigra development (p = 3.3E-04), and regulation of neuron apoptotic process (p=6.5E-04) in c0 (**Table S28**). Although *MOBP*'s role is not novel, the specific pathway co-enrichment identified in our study highlights its dual function in both structural maintenance and cellular response. In c3, the subnetwork (307 genes in c3, 61 genes in c0) are associated with regulation of dendritic spine development (p = 2.7E-04), myelination (p = 9.9E-04), neuronal cell-cell adhesion (p = 1.1E-03), and protein localization to synapse (p = 2.1E-03) in c3 and associated with transition metal ion transport (p = 1.1E-04), substantia nigra development (p = 3.3E-04), and regulation of neuron apoptotic process (p=6.5E-04) in c0 (**Supplementary Table S29**).

We also identify a few key drivers in astrocytes including *WDR49*, and heat shock protein family genes such as *HSPA1B*, *HSPB1*, *HSP90AA1, HSPH1*. *WDR49* is downregulated in PD and highly ranked as a key driver in cluster 2 astrocytes (KDA: FDR = 7.6E-12, 5.53 FE; Expression: 0.9 FC, FDR p = 3.4E-05). Interestingly, *WDR49* encodes a protein with unknown function, however, recent study suggests *WDR49* as the disease-associated subpopulation in Frontotemporal dementia carrying GRN mutation and Alzheimer's Disease[66]. The subnetwork around *WDR49* (100 genes) is involved in phosphatidic acid process (FDR p = 9.0E-03) (**Figure S8D, Supplementary Table S28-29**). Interestingly, a recent study shows that α-Synuclein (α-Syn), a protein causally linked to PD, binds to vesicles containing phosphatidic acid (PA), a plasma membrane phospholipid[67]. The identification of *WDR49* as a key driver opens a new avenue for research into its potential role in PD pathogenesis.

We also identify key drivers in microglial clusters. One driver is *APBB1IP* which is prominently associated with activation and modulation of innate immune response. *APBB1IP* is a regulator of leukocyte recruitment and pathogen clearance through complement-mediated phagocytosis[68,69]. In our analysis, *APBB1IP* is moderately downregulated (KDA: FDR = 8.5E-10, FE=5.45; Expression: FC=0.85, FDR = 1.1E-09), and the subnetwork around *APBB1IP* is involved in synapse pruning (FDR p = 6.7E-04), microglial cell migration (FDR p = 3.2E-02), and macrophage migration (FDR p = 4.7E-02) (**Figure 6E**, **Table S28-29**). The observed association between *APBB1IP* and pathways related to microglial activation underscores its importance in disease-related glial responses. Interestingly, the direct effectors of *APBB1IP* such as *C3* and *CX3CR1* are also downregulated and involved in immune responses and inflammation (**Figure 6E**). Moreover, *ST6GAL1*, an upstream gene of *APBB1IP*, encodes a member of glycosyltransferase family, ST6Gal-I sialyltransferase, is known to regulate several immunological process such as immunoglobulin production in B cells[70] or inflammatory pathways[71]. Therefore, our findings further underscore the importance of *APBB1IP* and its surrounding effectors in driving immune responses in microglia.

Last but not least, *HSP90AA1* is identified as key driver and is consistently upregulated across multiple glial cells including OPC (KDA: FDR = 3.6E-11, 10.4 FE; Expression: 1.3 FC, FDR =9.9E-13), oligodendrocytes in c3 (KDA: FDR = 1.2E-07, 3.69 FE; Expression: 1.1 FC, FDR =2.7E-08), oligodendrocytes in c0 (KDA: FDR = 1.7E-05, 3.52 FE; Expression: 1.2 FC, FDR = 0.005), microglia (KDA: FDR = 3.2E-06, 4.02 FE; Expression: 1.2 FC, FDR p = 9.7E-11), and astrocytes (KDA: FDR = 0.02, 4.3 FE; Expression: 1.1 FC, FDR = 1.2E-068) (**Figure 6E, Supplementary Figure S9F-I**). In the OPC cluster c5, the subnetwork around *HSP90AA1* is involved in responses to unfolded protein (FDR = 1.9E-10), protein ubiquitination (FDR = 7.8E-06), and apoptosis (FDR = 6.4E-05) (**Figure 6E, Supplementary Table S28**). The subnetworks around *HSP90AA1* in other glial cells are enriched for similar pathways (**Supplementary Table S28-29**). The consistent regulatory pattern of *HSP90AA1* in glial

cells suggests its potential involvement in maintaining glial homeostasis and contribution to the underlying neurodegenerative mechanisms.

DISCUSSION

In this study, we develop a metacell-based multiscale network analysis (MCMNA) framework for analyzing snRNA-seq data and apply it to a PD snRNA-seq data to identify cell type specific gene modules and key drivers for PD. Use of metacells rather than single cells addresses the challenge in dealing with the intrinsic sparsity in snRNA-seq data. The application of MCMNA to PD systematically identifies gene modules associated with PD and putative key network drivers for PD in the major brain cell types. Notably, 30% of the predicted drivers in neurons have been studied in PD, including *UCHL1*, *NSF*, *SNAP25*, *STMN2*, and *PREPL* while the rest 70% are novel or understudied, suggesting that our method can capture previously unrecognized regulators with potential significance in the disease.

Disease associated networks often exhibit sparse connectivity, reflecting the complexity and heterogeneity of underlying biological processes. Especially in snRNA-seq, some signals can be hidden by sparsity in gene expression profiles. One key advantage of the metacell approach in snRNA-seq is the ability to reduce data sparsity, thereby significantly enhancing downstream biological signals[27-29]. Aggregation of similar cells into metacells allows us to improve the detection of conserved markers specific to each cell type and reduce the false positive differentially expressed genes. Importantly, it strengthens gene-gene correlation analysis, which serves as a foundation for constructing more robust networks. We examine different metacell construction method and parameters choices (e.g. graining level – the ratio between the number of cells and the number of metacells, and expression representation – average vs. summing up, unweighted vs weighted) on our dataset to accelerate and enhance substantial downstream analyses. Additionally, the denser and more accurate correlation structure stemmed from biologically meaningful matrix (BMC) results in more meaningful gene co-expression networks. Additionally, the process of pruning enables more accurate construction of causal Bayesian Networks.

Our metacell-based MEGENA results show that different modules capture distinct biological pathways and reveal cell-type-specific regulatory patterns. For example, the top-ranked modules in neurons are involved in synaptic transmission and nervous system development while the top-ranked modules in

glial cells are enriched with diverse functions such as development process, metabolic signaling, and immune responses. We identify three distinct neuronal clusters, each displaying unique network architecture that reflects the cellular heterogeneity in PD. The typical neuronal cluster c7 with Tyrosine hydroxylase (TH) marker exhibits a more compact and highly connected network structure, while the neuronal cluster c9 with the novel *RIT2* marker has a sparser structure. Interestingly, in c9 neurons prone to be most degenerated in PD, only a small subset of cells demonstrates the most pronounced network changes (c9_M3), highlighting the selective vulnerability within this cluster. Cluster c7 has no hierarchical modules that are enriched with PD signatures, indicating that many dysregulated genes in this neuron are rather highly coordinated.

In addition to neurons, our metacell-based MEGENA allows us to uncover key features within glial cells, highlighting their distinct and potentially underappreciated roles in disease pathologies. Beyond neurons, oligodendrocytes cluster c0 and c3 have gene modules highly associated with PD, revealing broader functional roles than previously described. Several modules in c0 and c3 are enriched for mitochondrial autophagy, apoptosis, oligodendrocyte differentiation, and protein folding, revealing a more dynamic and multifaceted contribution to PD. Additionally, we observe an increased abundance of astrocytes (**Figure S1D**), yet their functional role in PD remains poorly understood. Our MEGENA pinpoints additional astrocytes involvement, highlighting modules enriched for signaling transduction, synapse assembly, suggesting possible contribution to maintaining or altering synaptic environment during disease progression. Microglia, in contrast, has fewer high-ranked modules, which are strongly associated with the immune response pathway. This agrees with well-established knowledge of their roles in neuroinflammation. Additionally, our study also indicates that several OPC modules associated with neural functions and glial differentiation are dysregulated, further highlighting the critical roles of OPC in PD.

Our module preservation analyses show that module preservation varies substantially across cell types, with highest score in oligodendrocytes, followed by astrocyte, microglia and neurons (**Supplementary Figure S4A-B**). This indicates that gene co-expression networks are more stable and

conserved in glial populations, while neuronal networks are comparatively more dynamic, and reflect greater heterogeneity. PD-related modules also have higher preservation overall, suggesting that disease-associated transcriptional programs are more robust and reproducible.

Our analysis identifies both known and novel gene targets that are strongly related to PD-related pathways, allowing us to propose a dynamic mechanistic model of neurodegeneration and susceptibility. Several key drivers in neuronal clusters such as *CADPS2*, *NSF*, *PREPL* are associated with calcium signaling, synaptic transmission, protein trafficking and have been studied in PD in some extent[43,51,52,61,72,73], highlighting the central role in neuronal functions. We also observe distinct neuronal subpopulations with unique key driver profiles and transcriptomic alterations, suggesting heterogeneity in the degree of regulatory pattern in degeneration and vulnerability. This observation aligns with previous studies reporting selective neuronal vulnerability in neurodegenerative diseases including PD[31,74]. Our findings suggest that multiple pathogenic processes may operate in parallel or sequentially. For example, in novel cluster 9 that shows degeneration in PD, *CADPS2* enriched for calcium signaling can point to novel mechanism of calcium dysregulation, contributing to stress or glial-neuronal signaling imbalance. In contrast, cluster c7 is dominated by key drivers in synaptic transmission, exocytosis and protein trafficking such as *NSF* or *PREPL*.

Oligodendrocytes in the central nervous system (CNS) are essential for forming myelin sheaths—multi-layered lipid membranes that insulate axons[75,76]. Among the top key drivers we identify in oligodendrocytes, *MOBP* stands out as the highest-ranked gene across both oligodendrocyte clusters c0 and c3. It is the third most abundant protein in CNS myelin and is known to contribute to myelin integrity. Moreover, recent study suggests a possible association between *MOBP* and Parkinson's disease[77]. Our findings highlight *MOBP* as a potential therapeutic target, given its role in maintaining structural stability and mediating cellular responses. Additionally, key drivers identified in microglia cells point toward compelling hypotheses involving innate immune activation. Consistent with the important role of *APBB1IP* suggested by our network, *APBB1IP* has been identified as modulator of immune gene co-expression in

specific microglial subtype in a recent aged mouse hippocampus study[78]. It is also recognized as a core component of microglial expression signatures[79,80]. Furthermore, human GWAS has linked *APBB1IP* to both AD and schizophrenia[81,82]. Our findings contribute to this growing body of evidence by highlighting the relevance of *APBB1IP* in PD.

Heat shock proteins (HSPs) are molecular chaperons that manage protein folding and prevent misfolding by regulating protein aggregation and disaggregation in both intracellular and extracellular environments, playing a critical role in maintaining proteostasis[83]. In our study, we observe consistent upregulation of HSPs across multiple glial cell types (**Figure 2B**). Pathways analysis further highlights the activation of processes such as protein folding and Hsf1 activation (**Figure 2C**). In particular, in PD, HSP60, HSP70, and HSP90 interact with α-synuclein, becoming incorporated into α-synuclein aggregates as they attempt to prevent or repair protein misfolding and accumulation[84-87]. Among these, we nominate *HSP90AA1* as a key candidate as it is significantly upregulated in 5 glial cell types and ranks highly in our network analysis. Notably, *HSP90AA1* has been identified as a marker of microglial activation in recent PD omics-studies[43,88]. Altogether, these findings support emerging evidence that non-neuronal cells significantly contribute to disease progression through immune signaling, stress response, or other metabolic and cellular response.

Our result also reveals strong concordance between bulk and snRNA-seq analyses, providing mutual validation of key molecular drivers implicated in PD. Several key drivers previously identified in bulk transcriptomic studies such as *STMN2* and *RALYL* are also drivers in our snRNA-seq network. This overlap underscores the robustness of our network-based approach and demonstrates that strong, biologically relevant signals identified in bulk are not only preserved but also refined at the single cell level. The replication of these findings confirms the reliability of bulk-based insights while offering higher resolution interpretation of cell-type-specific dynamics. By disentangling the contributions of distinct cell populations, we uncover cell-specific regulatory responses, enabling increased specificity in identifying therapeutic targets.

We apply our pipeline to a previously published snRNA-seq dataset to identify key drivers[25]. In this dataset, we identify 121 candidate key driver genes (**Supplementary Table S31**); however, there is minimal overlap with our results. Only a small number of genes are shared between the two analyses, including *HSP90AA1* in glial cells, and *CADPS2*, *CPLX1*, *SERINC1* in neurons (**Supplementary Table S32**). The limited concordance likely reflects the smaller sample size of the external dataset, which reduces the robustness of network construction, and consequently, the reliability of key driver identification.

We also highlight the functional contexts of PD GWAS genes using cell type specific Bayesian causal networks. Our causal networks in 5 major cell types across 8 cell clusters harbor a total of 509 PD GWAS genes, but only two of them (*NSF* in c7 and *MDGA2* in c6) are identified as key drivers (**Table S34**). Additionally, we predict *UCHL1*, a known PD causal gene, as a putative driver. Moreover, our network reveals a substantial number of novel key driver genes. These understudied genes may represent critical, yet unrecognized components involved in disease progression, particularly within specific cell types, enabling a broader and more comprehensive understanding of PD mechanisms.

We also compare our DEGs signatures with previously published bulk proteomics datasets of human SN[36]. We retrieve the identified differentially expressed proteins (DEPs) from 2 published cohorts (**Supplementary Table 34-35**). and assess the overlap with cell-type-specific DEGs using FET. The results show that the shared DEPs are predominantly enriched in neuronal clusters, with consistent directionality (**Supplementary Figure S10A-B, Supplementary Table S36-37**). While bulk proteomics capture an overall signal of the molecular dysregulation in PD, these findings indicate that the strongest and most robust changes are driven by neuronal population. This underscores the importance of cell-type resolved analyses for accurately interpreting disease-associated signatures.

In summary, our study identifies a set of molecular subnetworks and putative key driver genes that illuminate the molecular architecture of PD at the single cell resolution. By applying MCMNA to address sparsity in snRNA-seq, we uncover well known gene and novel regulatory candidates that may have

important roles in PD. These findings offer a better understanding of the cellular heterogeneity and regulatory dynamics underlying neuronal and glial cell populations.

Limitations of the study

Even though the cohort is the largest dataset of SN to date, the sample size is still relatively small. Furthermore, the current study lacks experimental validation to confirm computational validation. Even though we observe that previously perturbation signatures overlapped with our predicted *STMN2* network in c7 neurons, more validation is needed for the novel targets. Future work will focus on expanding the analysis to larger and more diverse datasets, and implement different confounding factors such as sex, Braak stages in the study to get more insights into the dynamic regulatory machinery. Our approach can also be improved with comprehensive longitudinal studies, extended studies beyond sporadic cases and specific brain regions, and incorporation of other omics-based networks such as proteomics and epigenomics. More importantly, this integrative approach establishes a foundation to guide future effort toward generating increasingly high-quality and informative co-expression and causal inference, paving the way for deeper mechanistic insights and the discovery of novel therapeutic targets.

Material Availability

This study did not generate new unique reagents. The raw data of the our snRNA-seq[20] are available from the Gene Expression Omnibus (GEO) under the accession code GSE184950. The raw data from Lee et al.[21] are available from GEO under the accession code GSE148434. The raw data from Martirosyan et al. dataset[22] are available from GEO under the accession code GSE243639. The raw data from Ma et al.[25] are available from GEO under the accession code GSE253462.

Data and Code Availability

The scripts and data used in this study have been deposited on GitHub and Zenodo at https://github.com/linhchu-3/PD_metacell_network and https://zenodo.org/records/20834300.

ACKNOWLEDGEMENTS

We thank the members from the B.Z. laboratory and Z.Y. laboratory for the comments and feedback during the development of the manuscript. The schematics were created using BioRender. This work was supported in part by grants from Parkinson's Foundation Center Grant PF-RC-936279 (to B.Z & Z.Y.), National Institutes of Health R01AG074010 (to B.Z.) and R01AG085182 (to B.Z.). This work was also supported in part through the computational and data resources and staff expertise provided by Scientific Computing and Data at the Icahn School of Medicine at Mount Sinai and supported by the Clinical and Translational Science Awards (CTSA) grant UL1TR004419 from the National Center for Advancing Translational Sciences. Research reported in this publication was also supported by the Office of Research Infrastructure of the NIH under award number S10OD026880 and S10OD030463. The content is solely the responsibility of the authors and does not necessarily represent the official views of the NIH. These funding sources had no role in the design and conduct of the study or collection, management, and analysis of the data.

AUTHORS' CONTRIBUTION

Conceptualization, B.Z.; Investigation, L.C., G.Y., X.Z., M.W., Z.Y., and B.Z.; Resources, B.Z.; Writing – Original Draft, L.C. and B.Z.; Writing – Review & Editing, All authors; Supervision, B.Z., Funding Acquisition, B.Z., and Z.Y.

DECLARATION OF INTERESTS

The authors declare that they have no competing interests

Table information

Table 1. Distribution of Metacell Sizes Across Cell Clusters in the MSSM dataset

Table 2. Number of Differentially Expressed Genes (DEGs) Identified by the Metacell (MC) Method per Cell Cluster

Table 3. Number of MEGENA modules, modules associated with PD DEGs/ pathway annotation

Table 4. Summary of Key Driver Genes

Supplementary Information

Table S1. Differentially Expressed Genes (DEGs) Identified by the Metacell (MC) Method per Cell Cluster

Table S2. Conserved Marker Genes in the MSSM Dataset Identified by Metacell

Table S3. Distribution of Metacell Sizes Across Cell Clusters in the VIB dataset

Table S4. Differentially Expressed Genes (DEGs) Identified by the Metacell (MC) Method per Cell Cluster in the VIB dataset

Table S5. Distribution of Metacell Sizes Across Cell Clusters in the KAIST dataset

Table S6. Differentially Expressed Genes (DEGs) Identified by the Metacell (MC) Method per Cell Cluster in the KAIST Dataset

Table S7. Distribution of Metacell Sizes Across Cell Clusters in the Columbia dataset

Table S8. Differentially Expressed Genes (DEGs) Identified by the Metacell (MC) Method per Cell Cluster in the Columbia Dataset

Table 9. Fisher's Exact Test (FET) Pathway Overlap Analysis Between VIB and MSSM Datasets

Table 10. Fisher's Exact Test (FET) Pathway Overlap Analysis Between KAIST and MSSM Datasets

Table S11. Fisher's Exact Test (FET) DEGs Overlap Analysis Between Columbia and MSSM Datasets

Table S12. Shared Enriched Pathways Across All Cell Types Between Metacell (MC) and Single-Cell (SC) Methods

Table S13 . Summary Statistics of Shared and Unique Upregulated Pathways Identified by SC and MC Methods

Table S14. Summary Statistics of Shared and Unique Downregulated Pathways Identified by SC and MC Methods

Table S15 . Unique Enriched Pathways per Cell Cluster Identified by the Metacell (MC) Method

Table S16 . Unique Enriched Pathways per Cell Cluster Identified by the Single-Cell (SC) Method

Table S17 . Fisher's Exact Test (FET) Results for STMN2 Pathway Preservation

Table S18 . Gene Membership in Significant Co-Expression Modules per Cell Cluster

Table S19 . GWAS Gene List Used in the Analysis

Table S20 . Module Ranking and Functional Pathway Annotation per Cell Cluster

Table S21. Comprehensive Fisher's Exact Test (FET) Results Across All MEGENA Modules

Table S22 . Complete Pathway Enrichment Information for All MEGENA Modules

Table S23. Summary of Module Preservation of All Modules at Cell Type Specific Levels

Table S24. FET Results of Module Preservation of All Modules at Cell Type Specific Levels

Table S25. Module Preservation of PD-related Modules at Cell Type Specific Levels

Table S26. FET Results of Module Preservation of All Modules at Cell Type Specific Levels

Table S27. Detailed Key Driver Genes Identified in Major Cell Types

Table S28 . Two-Layer Bayesian Network of selected key driver

Table S29 S. Pathway Enrichment Results for the Two-Layer Network of a Key Driver Gene using EnrichR database

Table S30. Detailed Key Drivers Gene identified in Major Cell Types in Columbia dataset

Table S31. Shared Key drivers in both datasets

Table S32 . GWAS Genes Mapped to Cell Type-Specific Gene Co-Expression Networks

Table S33 . Number of gene pairs before and after BMC generation.

Table 34. Differentially Expressed Proteins (DEPs) identified in published cohort of 15 PD and 15 Control

Table 35. Differentially Expressed Proteins (DEPs) identified in published cohort of 9 PD and 10 Control

Table S36. Fisher's Exact Test (FET) Results of shared DEGs and DEPs from cohort of 15 PD and 15 Control

Table S37. Fisher's Exact Test (FET) Results of shared DEGs and DEPs from cohort of 9 PD and 10 Control

Supplementary figure legends

Figure S1. Generation and characterization of metacells.

(A-B) Number of upregulated (A) and downregulated (B) differentially expressed genes (DEGs) in single cells and metacells across different conditions in 12 cell clusters.

(C) Number of conserved markers expressed genes (DEGs) in single and metacells across different conditions in 12 cell clusters.

(D) Nuclei fraction distribution between PD and control across 12 clusters. P-value was calculated using Wilcoxon rank sum test. * p-value < 0.05, ** p-value < 0.01.

(E) Volcano plot showing DEGs in selected major cell clusters c0 Oligodendrocyte, c5 Oligodendrocyte progenitor cell, and c6 Neuron. Top 5 upregulated and 5 downregulated DEGs (by -log10(FDR)) of each cell cluster are labeled in red or blue, respectively.

(F, I, L) UMAP plot showing cell clusters in VIB dataset (F) or KAIST dataset (I) or Columbia dataset (L)

(G, J, M) Expression pattern of known brain cell- type marker genes in VIB dataset (G) or KAIST dataset (J) or Columbia dataset (M)

(H, K, N) Comparation of FET enrichment of DEGs of MSSM dataset and VIB dataset (H) or MSSM dataset and KAIST dataset (K) or MSSM dataset and Columbia dataset (N) at cell cluster information. The size of dot is proportional to the Fold Enrichment and color intensity indicates the value of -log10(FET FDR). Ast, astrocytes; Neu, neurons; Mic, microglia; Oli, oligodendrocytes; OPC, oligodendrocyte progenitor cells; End, endothelia; Fib, fibroblast-like cells; Per, pericytes, T, T cells.

Figure S2. Comparison of the enrichments of all the pathways in the single cell (SC) and Metacell (MC) DEG signatures.

The -log10(FDR) values of all pathways from (A) upregulated or (B) downregulated differentially expressed genes (DEGs) were plotted. Black dot indicates the unique pathways from each method. Spearman correlation was calculated from all pathways in each cluster.

Figure S3. Generation of Biologically Meaningful Correlation (BMC) Matrix.

(A) Workflow of the method.

(B) Correlation distribution of SC and MC across 9 cell cluster. SC and MC at the mincell cutoff = 20%.

(C) Plot of Mean Square Error values against different min cell cut off across 12 cell cluster.

Figure S4. Module Preservation Summary between MSSM and Columbia Dataset of (A) all co-expressed modules and (B) of PD-associated co-expressed modules within the same cell type.

Figure S5. Metacell-based gene coepxression of neuronal cell clusters.

(A) Enrichment of the DEGs of PD in MEGENA modules in cluster 6 neurons. Color intensity is proportional to -log10(corrected FET p-value).

(B-C) Enrichment of the major pathways in MEGENA modules in cluster 6 neurons (B) and cluster 7 neurons (C).

(D) A global MEGENA network in cluster 7 neurons. The pie chart of each node indicates whether it is a DEG or GWAS gene. The size of a node is proportional to the node connectivity in the MEGENA network. (UP: Upregulated; DN: Downregulated).

Figure S6. Metacell-based gene coepxression of oligodendrocyte clusters.

(A, D) Enrichment of the major pathways in MEGENA modules in cluster 3 oligodendrocyte (A) and cluster 0 oligodendrocyte (D).

(B, C, F) The top-ranked module M41 of cluster 3 oligodendrocyte (B), M42 of cluster 3 oligodendrocyte (C), M3 of cluster 0 oligodendrocyte (F). The pie chart of each node indicates whether

it is a DEG or GWAS gene. The size of a node is proportional to the node connectivity in the MEGENA network. (UP: Upregulated; DN: Downregulated).

(E) Enrichment of the DEGs of PD in MEGENA modules in cluster 0 oligodendrocyte. Color intensity is proportional to -log10(corrected FET p-value).

Figure S7. Metacell-based gene coepxression of other glial cell clusters.

(A, C, G) Enrichment of the major pathways in MEGENA modules in cluster 2 astrocytes (A) cluster 1 microglia (C), and oligodendrocyte progenitor cells (OPC) (G).

(B, D, E, H, I) The top-ranked module M6 of cluster 2 astrocytes (B), M16 of cluster 1 microglia (D), M12 of cluster 1 microglia (E), M6 of cluster 5 OPC (H), and M8 of cluster 5 OPC (I). The pie chart of each node indicates whether it is a DEG or GWAS gene. The size of a node is proportional to the node connectivity in the MEGENA network. (UP: Upregulated; DN: Downregulated).

(F) Enrichment of the DEGs of PD in MEGENA modules in cluster 5 OPC. Color intensity is proportional to -log10(corrected FET p-value).

Figure S8. Bayesian Probabilistic Causal Network (BN) of neuronal cells.

(A) BN in cluster 6 neurons.

(B-D) The 2-layer network neighborhoods of *RALYL* in cluster 9 neurons (B), *NSF* in cluster 7 neurons (C), *MDGA2* in cluster 6 neurons (D), respectively. The size of a node is proportional to the node connectivity in the BN. The directionality of the arrow indicates the upstream-downstream effect. Nodes with burgundy labels are key drivers in that network.

Figure S9. Bayesian Probabilistic Causal Network (BN) of glial cells.

(A, C, E) BN in cluster 0 oligodendrocyte, C2 astrocytes, and C5 Oligodendrocyte progenitor cells (OPC).

(B, D, F-I) The 2-layer network neighborhoods of *MOBP* in cluster 0 oligodendrocyte (B), *WDR49* in cluster 2 astrocytes (D), *HSP90AA1* in cluster 0 oligodendrocyte (F), cluster 1 microglia (G), cluster 2 astrocytes (H), and cluster 3 oligodendrocyte (I), respectively. The size of a node is proportional to the node connectivity in the BN. The directionality of the arrow indicates the upstream-downstream effect. Nodes with burgundy labels are key drivers in that network.

Figure S10. Comparation of FET enrichment of DEGs of MSSM dataset and DEPs of the bulk proteomics study (A) using cohort of 15 PD and 15 Control or (B) using the cohort of 10 PD and 9 Control. The size of dot is proportional to the Fold Enrichment and color intensity indicates the value of -log10(FET FDR). Ast, astrocytes; Neu, neurons; Mic, microglia; Oli, oligodendrocytes; OPC, oligodendrocyte progenitor cells; End, endothelia; Fib, fibroblast-like cells; Per, pericytes, T, T cells.

Figure legends

Figure 1. Integrative network biology framework for decoding molecular mechanisms of Parkinson's Disease at single cell level.

(A) Construction and characterization of metacells.

(B) Metacell-based Multiscale Embedding Gene Co-expression Network Analysis.

(C) Metacell-based Bayesian Network and Key Driver analysis.

Figure 2. Generation and characterization of metacells.

(A) Number of differentially expressed genes (DEGs) in single and metacells across different conditions in 12 cell clusters.

(B) Volcano plot showing DEGs in selected major cell clusters c1 Microglia, c2 Astrocyte, c3 Oligodendrocyte, c7 Neuron and c9 Neuron. Top 5 upregulated and 5 downregulated DEGs (by -log10(FDR)) of each cell cluster are labeled in red or blue, respectively.

(C) Heatmap of top pathways enriched for up- and down-regulated genes in major cell type cluster.

(D-E) Barplot of gain of enriched pathways from up-regulated genes. (D) or downregulated DEGs (E) in major cell clusters. Color indicates the -log10(FDR) value of the corresponding pathway from single cell (SC) and metacell (MC) method. The number on the bar indicates the difference of -log10(FDR) value between MC and SC. Ast, astrocytes; Neu: neurons; Mic, microglia; Oli, oligodendrocytes; OPC, oligodendrocyte progenitor cells.

Figure 3. Metacells-based gene co-expression network of Parkinson's Disease.

(A) Comparison of FET enrichment of STMN2-related genes and STMN2 perturbation DEGs. Top value -log10(FDR), bottom value FE (SC: Single cell, MC: Metacell, FDR: False Discovery Rate, C6: Cluster 6, C7: Cluster 7, C9: Cluster 9, all: all cell clusters).

(B). Top 10 modules enriched for the PD-related signatures within each major cell cluster (UP: Upregulated; DN: Downregulated).

(C). A global MEGENA network in cluster 9 neurons. The module membership is represented by different colors. Seven Top ranked modules (M27, M28, M29, M30, M31, M32, M33) from parental module M3 and other modules enriched with other pathways (M5, M6, M12, M24) are highlighted.

(D) The top 30 MEGENA modules most enriched for the PD-related gene signatures include the PD DEGs with each cell cluster, and PD GWAS genes.

(E) Enrichment of the DEGs of PD in MEGENA modules in cluster 9 neurons. Color intensity is proportional to -log10(corrected FET p-value). (F) Enrichment of the major pathways in MEGENA modules in cluster 9 neurons.

Figure 4. Metacell-based gene coepxression of several glial cell clusters.

(A,C,F) Enrichment of the DEGs of PD in MEGENA modules in cluster 3 oligodendrocytes (A), cluster 2 astrocytes (C), or cluster 1 microglia (F). Color intensity is proportional to -log10(corrected FET p-value).

(B, D, E, G). The top-ranked module M10 of cluster 3 oligodendrocyte (B), M59 of cluster 2 astrocytes (D), M64 of cluster 2 astrocytes (E), or M15 of cluster 1 microglia (G). The pie chart of each node indicates whether it is a DEG or GWAS gene. The size of a node is proportional to the node connectivity in the MEGENA network. (UP: Upregulated; DN: Downregulated).

Figure 5. Bayesian Probabilistic Causal Netwwrk (BN) predicts novel key drivers in neuronal cells.

(A) BN in cluster 9 neurons.

(B) BN in cluster 7 neurons.

(C) Key driver identification in major cell types.

(D-F, H) The 2-layer network neighborhoods of *CADPS2* in cluster 9 neurons, *UCHL1* in cluster 7 neurons, *STMN2* in cluster 7 neurons, and *PREPL* in cluster 7 neurons, respectively. The size of a node is proportional to the node connectivity in the BN. The directionality of the arrow indicates the upstream-downstream effect. Nodes with burgundy labels are key drivers in that network.

(G). FET Enrichment for the overlap in vivo perturbation signature of STMN2 knockdown in mice and each n-layer neighborhood regulated by the STMN2 driver gene in cluster 7 neuron BN.

Figure 6. Bayesian Probabilistic Causal Network (BN) predicts novel key drivers in glial cells.

(A, C) BN in cluster 3 neurons (A), cluster 1 microglia (C).

(B, D, E) The 2-layer network neighborhoods of *MOBP* in cluster 3 oligodendrocytes, *APBB1IP* in cluster 1 microglia, *HSP90AA1* in cluster 5 oligodendrocyte progenitor cells. The size of a node is proportional to

the node connectivity in the BN. The directionality of the arrow indicates the upstream-downstream effect. Nodes with burgundy labels are key drivers in that network.

METHODS

Postmortem substantia nigra pars compacta samples used

The framework we develop in this paper, is created using a discovery cohort from our previous study of human 23 PD cases and 9 controls that contain 30,038 genes across 315,867 nuclei [20]. To validate some of our findings, we used three publicly available snRNA-seq datasets of human Substantia Nigra, Lee et al. dataset[21] of 6 PD and 6 Control cases,Martirosyan et al. dataset[22] of 15 PD and 14 Control, and Ma et al. dataset of 11 PD and 13 Control (cited).

snRNA-seq data preprocessing and clustering analysis

Three publicly available SnRNA-seq datasets were downloaded and subjected to the same pipeline we already established in the previous study[20]. Briefly, from Cell Ranger–derived unique molecular identifier (UMI) count matrices from all sequencing libraries, we performed QC by removing low- quality nuclei with too few genes (<200) or an excessive number (>2500) of genes detected. Then we removed insufficiently detected genes and discarded mitochondrial genes. We then performed a pre-clustering analysis using scRNA-seq data integration workflow based on R packages Harmony[89] and Seurat[90,91]. A total of 2000 most variable gene features were identified, scaled, and centered after regression out covariates sex, age, and PMI. Next, dimensional reduction was performed using principal components analysis (PCA) based on the 2000 most variable genes. Preclustering analysis, cluster stability analysis and double prediction analysis were performed as previously described[20]. As a result, we obtained 28,339 genes across 44,450 high quality nuclei in KAIST dataset, 33,537 genes across 69,147 high quality nuclei in VIB dataset, and 31,280 genes across 194,905 high quality nuclei in Columbia dataset.

Cluster cell type annotation

For each major cluster of 3 external datasets, we interrogated the expression patterns of known gene markers to annotate clusters into major cell types: neurons (*SLC17A6, RBFOX3*, *GAD1*), astrocytes (*AQP4*), oligodendrocytes (*MOG*), microglia (*C3*, *CSF1R*), oligodendrocyte progenitor cells (*VCAN*), endothelial cells (*FLT1*), pericytes (*PDGFRB*), and T cell (*SKAP1*). Additionally, we used sets of marker genes we identified in our study such as typical dopaminergic markers in c7 (*SLC6A3*, *TH*, *SLC18A2*), enriched marker in c6 (*DCC* and *GALNTL6*), and novel enriched marker in c9 (*RIT2*).

Construction of Metacells

Metacells were built based on a log-normalized gene expression matrix using an R package SuperCell[29]. The set of features (genes) used for the construction of metacells was defined by the set of the most 2000 variable genes. The top principal components (top 10 by default) are used to build a single-cell network with a k-nearest neighbors algorithm built in the R package (k-NN, k =5 by default). To preserve the cell type annotation and phenotype information from snRNA-seq data, metacells were constructed by merging single cell (nodes) at a graining level $\gamma = 15$ at the cell cluster and condition specific manner. A gene expression profile of metacells was computed by averaging gene expression within metacells, or summing up the single cell raw values, followed by log normalization.

Identification of differentially expressed genes

DEGs between PD and control and DEGs between PD and control in each cluster were identified using the R package MAST implemented in Seurat [90,91], with correction for PMI, age, and sex. DEGs were identified at the cutoff of Bonferroni corrected P value ≤0.05 and fold change ≥10%

Identification of conserved markers

A conserved marker is defined as a cell type specific gene that distinguishes a given cell type from others and shows consistent expression across 2 groups (Control and PD). Conserved markers in each cluster were performed using the *FindConservedMarkers* function in the R Seurat package[90,91]. Genes that reached the average expression levels across the cluster with logFC >0 were identified as conserved markers.

Overlap and functional enrichment analysis

To understand biological processes underlying the DEGs and co-expressed modules, we tested these signatures for enrichment of gene ontology (GO) and canonical functional pathway gene sets. We used the functional gene sets collection from the Molecular Signature Database MSigDb that was downloaded from the R package called *msigdb* from https://github.com/mw201608/msigdb. Functional enrichment of DEGs with MSigDB gene annotation collections was examined using the R package *clusterProfiler*[92]. To test the pathways of 2 layer-network neighborhood of candidate genes, we loaded the gene members onto *EnrichR* server and identified the top pathways associated with these genes[93-95]. Functional enrichment analysis between two gene sets were determined by Fisher's exact test (FET). Fold Enrichment (FE) was calculated as the ratio between observed overlap and expected overlap size. We employed the Benjamini-Hochberg (BH) approach to correct for FDR.

Generation of Biologically meaningful correlation (BMC) matrix

We implemented a novel framework to generate biologically meaningful correlation (BMC) matrix. First, we filtered out unexpressed genes using a range of minimum cell cutoff (i.e. requiring that each gene be expressed in at least x% of cells expressed), selected genes that were conserved markers, and removed noisy cells (those expressing fewer than 500 genes). For each gene pair, we excluded zero counts before calculating all possible signed Spearman correlation coefficients (SCCs) (FDR = 1) using the *calculate.rho.signed* function in MEGENA (v.1.6.0) available from https://github.com/songw01/MEGENA/blob/master/MEGENA_1.6.tar.gz. To capture all signed correlation, we set direction as "positive" or "negative", then combined results from both directions.

Next, we evaluated whether the distribution of correlation coefficient (ρ) in each cell cluster followed a truncated normal distribution. For each cell cluster at each minimum cell cutoff, we first calculated the mean and standard deviation of the observed ρ values, and used to define a truncated normal distribution bounded by minimum and maximum observed ρ. The truncated normal density was

generated at 100 equally spaced points across this range using the *truncnorm* function R package. We applied linear interpolation using *approx* function in R to estimate the histogram densities at 100 equally spaced points across the observed range of correlations. We then calculated Mean Square Error (MSE) using this equation: $MSE = \frac{1}{n}\sum_{i=1}^{n}(Y_i - \hat{Y}_i)$, where n is the number of evaluation points (100 in this case) in the correlation distribution, Y is the observed counts of the SCC, and $\hat{Y}$ is the predicted counts of SCCs from the stimulated truncated normalization distribution.

We then plotted MSE values against the minimum cell cutoff to identify the optimal cutoff. The mean (μ) of all gene pair correlation was calculated. Finally, at the optimal minimum cell cutoff, all significant gene pairs correlation (FDR < 0.05) were identified. However, even after applying FDR < 0.05, substantial fraction of gene pairs fell within the (0, μ) interval across clusters ranging from 0% in C9 to 47% in C5 **(Table S24**). Therefore, at the optimal mincell cutoff, we further subtracted gene pairs in the (0, μ) interval. This new set of significant correlations, named BMC matrix, potentially removed weak and suspicious associations prior to network construction.

MEGENA co-expression network analysis

For each cell cluster, optimized BMC matrix was then subjected to MEGENA pipeline[32] (v.1.6.0) available from https://github.com/songw01/MEGENA/blob/master/MEGENA_1.6.tar.gz. Briefly, significant SCCs were ranked and iteratively tested for planarity to grow a Planar Filtered Network (PFN) by using the PMFG algorithm. Multiscale Clustering Analysis (MCA) was conducted with the resulting PFN to identify co-expression modules at different network scale topology. We identified a total of 1057 modules in all cell clusters **(Table 3)**. To annotate the biological functions associated within the modules, we performed MSigDB gene set enrichment analysis using FET as described above. Most of these modules (42.2% to 73.3%) were enriched for MSigDB GO/Pathway gene sets (adjusted P value < 0.05 (**Table 3**), indicating that MEGENA can capture biologically functional signals. For simplicity, modules were annotated by top enriched pathway and then grouped into functional category.

The MEGENA modules are formed in a hierarchy with parent-child relationship and illustrated by a sunburst plot using R package *ggsunburst* available from https://github.com/didacs/ggsunburst.

To prioritize the gene modules with respect to the PD phenotypes, we applied a ranking metric across multiple features such as enrichment with 1) the DEGs signatures identified and 2) PD GWAS signatures. PD GWAS hits were downloaded from GWAS catalogue (https://www.ebi.ac.uk/gwas/) and filtered to remove redundance. The ranking of the top modules was based on the average of -log10(Padj value) of all the features. The circular track plot to visualize the gene co-expression network modules and their enrichment was generated using R package *NetWeaver* available from https://github.com/mw201608/NetWeaver. The global network of c9 Neuron was visualized using Gephi (version 0.10.1). The global network of other cell clusters and subnetwork of selected gene modules were visualized using Cytoscape (version 3.10.3).

MEGENA co-expression module preservation analysis

We assessed module preservation in the MEGENA co-expression network between our MSSM SnRNAseq data and Columbia's snRNA-seq data using the Fisher's exact Test, with genes from the corresponding global network in our dataset as the background. We performed pairwise comparisons of modules within the same cell type (e.g. microglia modules versus microglia modules.

Bayesian probabilistic causal network inference

We constructed the global Bayesian probabilistic causal network (BN) based on a Monte Carlo Markov Chain simulation-based procedures using RIMBANet software[33,34]. We used established transcription factor (TF)-target relationships for *Homo sapiens* acquired from TFLink database[96] as prior to infer regulatory relationships among genes. In the causal network construction, the TFs are allowed to be parent nodes of their target genes, but targets are inhibited to be parent nodes of their TFs.

First, we began by removing genes expressed in fewer than 20% of metacells. To resolve the issue of dropouts in snRNA-seq, we applied an iterative pruning procedure to remove genes and nuclei

in a data matrix. For this, we defined two thresholds: Tg, representing the maximum allowable gene missing rate, and Tm, representing the maximum allowable metacell missing rate. For each iteration, genes exceeding the Tg threshold and metacells exceeding the Tm threshold were removed. This pruning process was repeated iteratively until the number of remaining genes and metacells stabilized, with no changes observed between iterations. After two iterations, the numbers converged. We then selected the cutoff Tg = 0.4 and Tm = 0.4 for all cell clusters.

We employed a network averaging approach where 1000 networks will be generated starting with a different random structure. Links that appear in more than 30% of the networks were used as a final consensus network. An iterative de-loop procedure was executed to ensure that the consensus network was a directed acyclic graph, removing the most-weakly supported link of all links involved in any loop. The global network of cell clusters was visualized using Cytoscape (version 3.10.3).

Key driver analysis (KDA)

For each major cell type cluster, we performed key driver analysis (KDA)[35] on the consensus Bayesian network to identify key hub genes that are DEGs and regulate a large number of downstream nodes. Here we loaded all the BN nodes and DEGs from the corresponding cluster as input for KDA, making the resulting key drivers' representative of the global network. The algorithm defined a background subnetwork by identifying nodes within an L-layer neighborhood of each DEG in the network. For each node in this subnetwork, it assessed enrichment within its L-layer downstream neighborhood for DEGs. In our analysis, we tested combination of 1-layer (1L), 2-layer (2L), and 3-layer (3L) neighborhoods, considering both directed and undirected edges to account for potential missing directionality in the causal BN. Then key drivers were compiled based on those identified from the 3L directed analysis and those shared by both 2L and 1L undirected analyses. To ensure that key drivers had sufficient downstream targets, we also excluded drivers with a degree lower than the average degree of all nodes in the network. KDA was performed by the R package *KDA* (version 0.02) downloaded from https://github.com/mw201608/mnml-public/blob/master/pkgs/KDA_0.2.2.zip.

PubMed literature mining

The function EUtilsSummary (type = "esearch", db="pubmed", datetype='pdat') from the R package RISmed (version 2.3.0) was used to query and download content from Pubmed. The query words were built as “KDG and Parkinson's disease” where the word KDG was replaced by the gene symbol of a specific key driver gene. For example, the query of “CADPS2 and Parkinson's disease” would pull out all the papers that have the occurrence of CADPS2 and Parkinson's disease.

Statistical Analysis

Fisher’s exact test (FET) was used to examine the significance of the overlap between two gene sets. An overlap with BH corrected $p < 0.05$ was considered to be statistically significant unless stated otherwise.

REFERENCES

1. De Lau LM, Breteler MM. Epidemiology of Parkinson's disease. *The Lancet Neurology*. 2006;5(6):525-535.
2. Parkinson J. An essay on the shaking palsy. *The Journal of neuropsychiatry and clinical neurosciences*. 2002;14(2):223-236.
3. Chu Y, Kordower JH. Age-associated increases of α-synuclein in monkeys and humans are associated with nigrostriatal dopamine depletion: is this the target for Parkinson's disease? *Neurobiology of disease*. 2007;25(1):134-149.
4. Pakkenberg B, Møller A, Gundersen HJ, Dam AM, Pakkenberg H. The absolute number of nerve cells in substantia nigra in normal subjects and in patients with Parkinson's disease estimated with an unbiased stereological method. *Journal of Neurology, Neurosurgery & Psychiatry*. 1991;54(1):30-33.
5. Blauwendraat C, Nalls MA, Singleton AB. The genetic architecture of Parkinson's disease. *The Lancet Neurology*. 2020;19(2):170-178.
6. Nalls MA, Blauwendraat C, Vallerga CL, et al. Identification of novel risk loci, causal insights, and heritable risk for Parkinson's disease: a meta-analysis of genome-wide association studies. *The Lancet Neurology*. 2019;18(12):1091-1102.
7. McGeer PL, Itagaki S, Boyes BE, McGeer E. Reactive microglia are positive for HLA‐DR in the substantia nigra of Parkinson's and Alzheimer's disease brains. *Neurology*. 1988;38(8):1285-1285.
8. Le W-d, Rowe D, Xie W, Ortiz I, He Y, Appel SH. Microglial activation and dopaminergic cell injury: an in vitro model relevant to Parkinson's disease. *Journal of Neuroscience*. 2001;21(21):8447-8455.
9. Gao HM, Jiang J, Wilson B, Zhang W, Hong JS, Liu B. Microglial activation‐mediated delayed and progressive degeneration of rat nigral dopaminergic neurons: relevance to Parkinson's disease. *Journal of neurochemistry*. 2002;81(6):1285-1297.

10. Zhang W, Wang T, Pei Z, et al. Aggregated α-synuclein activates microglia: a process leading to disease progression in Parkinson's disease. *The FASEB Journal*. 2005;19(6):533-542.
11. Zhang W, Phillips K, Wielgus AR, et al. Neuromelanin activates microglia and induces degeneration of dopaminergic neurons: implications for progression of Parkinson's disease. *Neurotoxicity research*. 2011;19:63-72.
12. Waak J, Weber SS, Waldenmaier A, et al. Regulation of astrocyte inflammatory responses by the Parkinson's disease-associated gene DJ–1. *The FASEB Journal*. 2009;23(8):2478-2489.
13. Gray MT, Woulfe JM. Striatal blood–brain barrier permeability in Parkinson's disease. *Journal of Cerebral Blood Flow & Metabolism*. 2015;35(5):747-750.
14. Kam T-I, Hinkle JT, Dawson TM, Dawson VL. Microglia and astrocyte dysfunction in parkinson's disease. *Neurobiology of disease*. 2020;144:105028.
15. Hirsch EC, Hunot S. Neuroinflammation in Parkinson's disease: a target for neuroprotection? *The Lancet Neurology*. 2009;8(4):382-397.
16. Bae E-J, Pérez-Acuña D, Rhee KH, Lee S-J. Changes in oligodendroglial subpopulations in Parkinson's disease. *Molecular brain*. 2023;16(1):65.
17. Azevedo C, Teku G, Pomeshchik Y, et al. Parkinson's disease and multiple system atrophy patient iPSC-derived oligodendrocytes exhibit alpha-synuclein–induced changes in maturation and immune reactive properties. *Proceedings of the National Academy of Sciences*. 2022;119(12):e2111405119.
18. Djelloul M, Holmqvist S, Boza-Serrano A, et al. Alpha-synuclein expression in the oligodendrocyte lineage: an in vitro and in vivo study using rodent and human models. *Stem cell reports*. 2015;5(2):174-184.
19. Wang Q, Zhang Y, Wang M, et al. The landscape of multiscale transcriptomic networks and key regulators in Parkinson's disease. *Nature communications*. 2019;10(1):5234.
20. Wang Q, Wang M, Choi I, et al. Molecular profiling of human substantia nigra identifies diverse neuron types associated with vulnerability in Parkinson's disease. *Science advances*. 2024;10(2):eadi8287.
21. Lee AJ, Kim C, Park S, et al. Characterization of altered molecular mechanisms in Parkinson's disease through cell type–resolved multiomics analyses. *Science advances*. 2023;9(15):eabo2467.
22. Martirosyan A, Ansari R, Pestana F, et al. Unravelling cell type-specific responses to Parkinson's Disease at single cell resolution. *Molecular neurodegeneration*. 2024;19(1):1-24.
23. Adams L, Song MK, Yuen S, Tanaka Y, Kim Y-S. A single-nuclei paired multiomic analysis of the human midbrain reveals age-and Parkinson's disease–associated glial changes. *Nature aging*. 2024;4(3):364-378.
24. Chatila Z, Yadav A, Mares J, et al. RNA-and ATAC-sequencing reveals a unique CD83+ microglial population focally depleted in Parkinson's disease. *BioRxiv*. 2023:2023.05. 17.540842.
25. Ma M, Paryani F, Jakubiak K, et al. The spatial landscape of glial pathology and T cell response in Parkinson's disease substantia nigra. *Nature Communications*. 2025;16(1):7146.
26. Cheng Y, Ma X, Yuan L, Sun Z, Wang P. Evaluating imputation methods for single-cell RNA-seq data. *BMC bioinformatics*. 2023;24(1):302.
27. Baran Y, Bercovich A, Sebe-Pedros A, et al. MetaCell: analysis of single-cell RNA-seq data using K-nn graph partitions. *Genome biology*. 2019;20:1-19.
28. Ben-Kiki O, Bercovich A, Lifshitz A, Tanay A. Metacell-2: a divide-and-conquer metacell algorithm for scalable scRNA-seq analysis. *Genome biology*. 2022;23(1):100.

29. Bilous M, Tran L, Cianciaruso C, et al. Metacells untangle large and complex single-cell transcriptome networks. *BMC bioinformatics*. 2022;23(1):336.
30. Persad S, Choo Z-N, Dien C, et al. SEACells infers transcriptional and epigenomic cellular states from single-cell genomics data. *Nature Biotechnology*. 2023;41(12):1746-1757.
31. Liang M, Chu L, Yue Z. New Multiomic Studies Shed Light on Cellular Diversity and Neuronal Susceptibility in Parkinson's Disease. *Movement Disorders*. 2025;40(3):431-437.
32. Song W-M, Zhang B. Multiscale embedded gene co-expression network analysis. *PLoS computational biology*. 2015;11(11):e1004574.
33. Zhu J, Zhang B, Smith EN, et al. Integrating large-scale functional genomic data to dissect the complexity of yeast regulatory networks. *Nature genetics*. 2008;40(7):854-861.
34. Zhu J, Wiener MC, Zhang C, et al. Increasing the power to detect causal associations by combining genotypic and expression data in segregating populations. *PLoS computational biology*. 2007;3(4):e69.
35. Zhang B, Zhu J. Identification of key causal regulators in gene networks. 2013:5-8.
36. Jang Y, Pletnikova O, Troncoso JC, et al. Mass spectrometry–based proteomics analysis of human substantia nigra from Parkinson's disease patients identifies multiple pathways potentially involved in the disease. *Molecular & Cellular Proteomics*. 2023;22(1)
37. Sollis E, Mosaku A, Abid A, et al. The NHGRI-EBI GWAS Catalog: knowledgebase and deposition resource. *Nucleic acids research*. 2023;51(D1):D977-D985.
38. Wang M, Li A, Sekiya M, et al. Transformative Network Modeling of Multi-Omics Data Reveals Detailed Circuits, Key Regulators, and Potential Therapeutics for Alzheimer's Disease. *Neuron*. 2020; S0896-6273(20):8. doi:10.1016/j.neuron.2020.11.002
39. Wang Q, Zhang Y, Wang M, et al. The landscape of multiscale transcriptomic networks and key regulators in Parkinson's disease. *Nat Commun*. Nov 20 2019;10(1):5234. doi:10.1038/s41467-019-13144-y
40. Grishanin RN, Kowalchyk JA, Klenchin VA, et al. CAPS acts at a prefusion step in dense-core vesicle exocytosis as a PIP2 binding protein. *Neuron*. 2004;43(4):551-562.
41. Zhang L, Wei Z, Dai Y, He F, Sun T. The role of CAPS in Ca2+-regulated exocytosis: promotion of vesicle tethering, priming, and fusion. *Neuropharmacology*. 2024:110247.
42. Iguchi H, Katsuzawa T, Saruta C, et al. Calcium-dependent activator protein for secretion 2 is involved in dopamine release in mouse midbrain neurons. *Frontiers in Molecular Neuroscience*. 2024;17:1444629.
43. Smajić S, Prada-Medina CA, Landoulsi Z, et al. Single-cell sequencing of human midbrain reveals glial activation and a Parkinson-specific neuronal state. *Brain*. 2022;145(3):964-978.
44. Mukherjee S, Goswami S, Dash S, Samanta D. Structural basis of molecular recognition among classical cadherins mediating cell adhesion. *Biochemical Society Transactions*. 2023;51(6):2103-2115.
45. Moreland T, Poulain FE. To stick or not to stick: The multiple roles of cell adhesion molecules in neural circuit assembly. *Frontiers in Neuroscience*. 2022;16:889155.
46. Steinberg C, Roston TM, van der Werf C, et al. RYR2-ryanodinopathies: from calcium overload to calcium deficiency. *Europace*. 2023;25(6):euad156.
47. Ansari U, Chen V, Sedighi R, et al. Role of the UNC13 family in human diseases: A literature review. *AIMS neuroscience*. 2023;10(4):388.

48. Liu Y, Fallon L, Lashuel HA, Liu Z, Lansbury PT. The UCH-L1 gene encodes two opposing enzymatic activities that affect α-synuclein degradation and Parkinson's disease susceptibility. *Cell*. 2002;111(2):209-218.
49. Yang J, Kong L, Zou L, Liu Y. The role of synaptic protein NSF in the development and progression of neurological diseases. *Frontiers in Neuroscience*. 2024;18:1395294.
50. Burré J, Sharma M, Tsetsenis T, Buchman V, Etherton MR, Südhof TC. α-Synuclein promotes SNARE-complex assembly in vivo and in vitro. *Science*. 2010;329(5999):1663-1667.
51. Miller RM, Callahan LM, Casaceli C, et al. Dysregulation of gene expression in the 1-methyl-4-phenyl-1, 2, 3, 6-tetrahydropyridine-lesioned mouse substantia nigra. *Journal of Neuroscience*. 2004;24(34):7445-7454.
52. Gao X, Huang Z, Feng C, et al. Multimodal analysis of gene expression from postmortem brains and blood identifies synaptic vesicle trafficking genes to be associated with Parkinson's disease. *Briefings in bioinformatics*. 2021;22(4):bbaa244.
53. Li Z, Langhans SA. Transcriptional regulators of Na, K-ATPase subunits. *Frontiers in cell and developmental biology*. 2015;3:66.
54. Paulussen I, Beckert H, Musial TF, et al. SV2B defines a subpopulation of synaptic vesicles. *Journal of Molecular Cell Biology*. 2023;15(9):mjad054.
55. Janz R, Goda Y, Geppert M, Missler M, Südhof TC. SV2A and SV2B function as redundant Ca2+ regulators in neurotransmitter release. *Neuron*. 1999;24(4):1003-1016.
56. Thornburg‐Suresh EJ, Summers DW. Microtubules, Membranes, and Movement: New Roles for Stathmin‐2 in Axon Integrity. *Journal of neuroscience research*. 2024;102(9):e25382.
57. Gagliardi D, Pagliari E, Meneri M, et al. Stathmins and motor neuron diseases: pathophysiology and therapeutic targets. *Biomedicines*. 2022;10(3):711.
58. San Juan IG, Nash LA, Smith KS, et al. Loss of mouse Stmn2 function causes motor neuropathy. *Neuron*. 2022;110(10):1671-1688. e6.
59. Duarte ML, Wang M, Gomes I, et al. Multiomics Analyses Identify Proline Endopeptidase–Like Protein As a Key Regulator of Protein Trafficking, a Pathway Underlying Alzheimer's Disease Pathogenesis. *Molecular Pharmacology*. 2023;104(1):1-16.
60. Morawski M, Nuytens K, Juhasz T, et al. Cellular and ultra structural evidence for cytoskeletal localization of prolyl endopeptidase-like protein in neurons. *Neuroscience*. 2013;242:128-139.
61. Santos GS, Oyadomari WY, Carvalho EA, Torquato RS, Oliveira V. Prolyl endopeptidase-like facilitates the α-synuclein aggregation seeding, and this effect is reverted by serine peptidase inhibitor PMSF. *Biomolecules*. 2020;10(6):962.
62. Joset P, Wacker A, Babey R, et al. Rostral growth of commissural axons requires the cell adhesion molecule MDGA2. *Neural development*. 2011;6:1-16.
63. Latourelle JC, Pankratz N, Dumitriu A, et al. Genomewide association study for onset age in Parkinson disease. *BMC medical genetics*. 2009;10:1-14.
64. Montague P, McCallion AS, Davies RW, Griffiths IR. Myelin-associated oligodendrocytic basic protein: a family of abundant CNS myelin proteins in search of a function. *Developmental neuroscience*. 2006;28(6):479-487.
65. Yamamoto Y, Yoshikawa H, Nagano S, et al. Myelin‐associated oligodendrocytic basic protein is essential for normal arrangement of the radial component in central nervous system myelin. *European Journal of Neuroscience*. 1999;11(3):847-855.

66. Rajicic A, Giannini LA, Gerrits E, et al. WDR49‐Positive Astrocytes Mark Severity of Neurodegeneration in Frontotemporal Lobar Degeneration and Alzheimer's Disease. *Glia*. 2025;73(5):948-968.
67. Mizuno S, Sasai H, Kume A, et al. Dioleoyl‐phosphatidic acid selectively binds to α‐synuclein and strongly induces its aggregation. *FEBS letters*. 2017;591(5):784-791.
68. Medraño-Fernandez I, Reyes R, Olazabal I, et al. RIAM (Rap1-interacting adaptor molecule) regulates complement-dependent phagocytosis. *Cellular and Molecular Life Sciences*. 2013;70:2395-2410.
69. Klapproth S, Sperandio M, Pinheiro EM, et al. Loss of the Rap1 effector RIAM results in leukocyte adhesion deficiency due to impaired β2 integrin function in mice. *Blood, The Journal of the American Society of Hematology*. 2015;126(25):2704-2712.
70. Irons EE, Punch PR, Lau JT. Blood-borne ST6GAL1 regulates immunoglobulin production in B cells. *Frontiers in Immunology*. 2020;11:617.
71. Holdbrooks AT, Ankenbauer KE, Hwang J, Bellis SL. Regulation of inflammatory signaling by the ST6Gal-I sialyltransferase. *PLoS One*. 2020;15(11):e0241850.
72. Obergasteiger J, Überbacher C, Pramstaller PP, Hicks AA, Corti C, Volta M. CADPS2 gene expression is oppositely regulated by LRRK2 and alpha-synuclein. *Biochemical and biophysical research communications*. 2017;490(3):876-881.
73. Tang L, Xu N, Huang M, et al. A primate nigrostriatal atlas of neuronal vulnerability and resilience in a model of Parkinson's disease. *Nature Communications*. 2023;14(1):7497.
74. Surmeier DJ, Obeso JA, Halliday GM. Selective neuronal vulnerability in Parkinson disease. *Nature Reviews Neuroscience*. 2017;18(2):101-113.
75. Stadelmann C, Timmler S, Barrantes-Freer A, Simons M. Myelin in the central nervous system: structure, function, and pathology. *Physiological reviews*. 2019;99(3):1381-1431.
76. Baumann N, Pham-Dinh D. Biology of oligodendrocyte and myelin in the mammalian central nervous system. *Physiological reviews*. 2001;81(2):871-927.
77. Siokas V, Aloizou AM, Liampas I, et al. Myelin‐associated oligodendrocyte basic protein rs616147 polymorphism as a risk factor for Parkinson's disease. *Acta Neurologica Scandinavica*. 2022;145(2):223-228.
78. Tomkins JE, Methi A, Bouland G, et al. Genetic modulation of immune gene co-expression in the aged mouse hippocampus by the Apbb1ip locus. *bioRxiv*. 2025:2025.06. 04.657838.
79. Geirsdottir L, David E, Keren-Shaul H, et al. Cross-species single-cell analysis reveals divergence of the primate microglia program. *Cell*. 2019;179(7):1609-1622. e16.
80. Boche D, Gordon MN. Diversity of transcriptomic microglial phenotypes in aging and Alzheimer's disease. *Alzheimer's & Dementia*. 2022;18(2):360-376.
81. Morgan A, Turic D, Jehu L, et al. Association studies of 23 positional/functional candidate genes on chromosome 10 in late‐onset Alzheimer's disease. *American Journal of Medical Genetics Part B: Neuropsychiatric Genetics*. 2007;144(6):762-770.
82. Ashbrook DG, Cahill S, Hager R. A cross-species systems genetics analysis links APBB1IP as a candidate for schizophrenia and prepulse inhibition. *Frontiers in behavioral neuroscience*. 2019;13:266.
83. Hunt AP, Minett GM, Gibson OR, Kerr GK, Stewart IB. Could heat therapy be an effective treatment for Alzheimer's and Parkinson's diseases? A narrative review. *Frontiers in physiology*. 2020;10:1556.

84. Saxena SK, Sharma D, Kumar S, et al. Decoding the role of large heat shock proteins in the progression of neuroinflammation-mediated neurodegenerative disorders. *Neuroprotection*. 2025;
85. Bohush A, Bieganowski P, Filipek A. Hsp90 and its co-chaperones in neurodegenerative diseases. *International journal of molecular sciences*. 2019;20(20):4976.
86. Li H, Yang J, Wang Y, Liu Q, Cheng J, Wang F. Neuroprotective effects of increasing levels of HSP70 against neuroinflammation in Parkinson's disease model by inhibition of NF-κB and STAT3. *Life Sciences*. 2019;234:116747.
87. Klucken J, Shin Y, Masliah E, Hyman BT, McLean PJ. Hsp70 reduces α-synuclein aggregation and toxicity. *Journal of Biological Chemistry*. 2004;279(24):25497-25502.
88. Zhu B, Park J-M, Coffey SR, et al. Single-cell transcriptomic and proteomic analysis of Parkinson's disease Brains. *Science Translational Medicine*. 2024;16(771):eabo1997.
89. Korsunsky I, Millard N, Fan J, et al. Fast, sensitive and accurate integration of single-cell data with Harmony. *Nature methods*. 2019;16(12):1289-1296.
90. Butler A, Hoffman P, Smibert P, Papalexi E, Satija R. Integrating single-cell transcriptomic data across different conditions, technologies, and species. *Nature biotechnology*. 2018;36(5):411-420.
91. Satija R, Farrell JA, Gennert D, Schier AF, Regev A. Spatial reconstruction of single-cell gene expression data. *Nature biotechnology*. 2015;33(5):495-502.
92. Yu G, Wang L-G, Han Y, He Q-Y. clusterProfiler: an R package for comparing biological themes among gene clusters. *Omics: a journal of integrative biology*. 2012;16(5):284-287.
93. Chen EY, Tan CM, Kou Y, et al. Enrichr: interactive and collaborative HTML5 gene list enrichment analysis tool. *BMC bioinformatics*. 2013;14:1-14.
94. Kuleshov MV, Jones MR, Rouillard AD, et al. Enrichr: a comprehensive gene set enrichment analysis web server 2016 update. *Nucleic acids research*. 2016;44(W1):W90-W97.
95. Xie Z, Bailey A, Kuleshov MV, et al. Gene set knowledge discovery with Enrichr. *Current protocols*. 2021;1(3):e90.
96. Liska O, Bohár B, Hidas A, et al. TFLink: an integrated gateway to access transcription factor–target gene interactions for multiple species. *Database*. 2022;2022:baac083.

Click here to access/download;Figure;Main Figures_final_HighRes.pdf

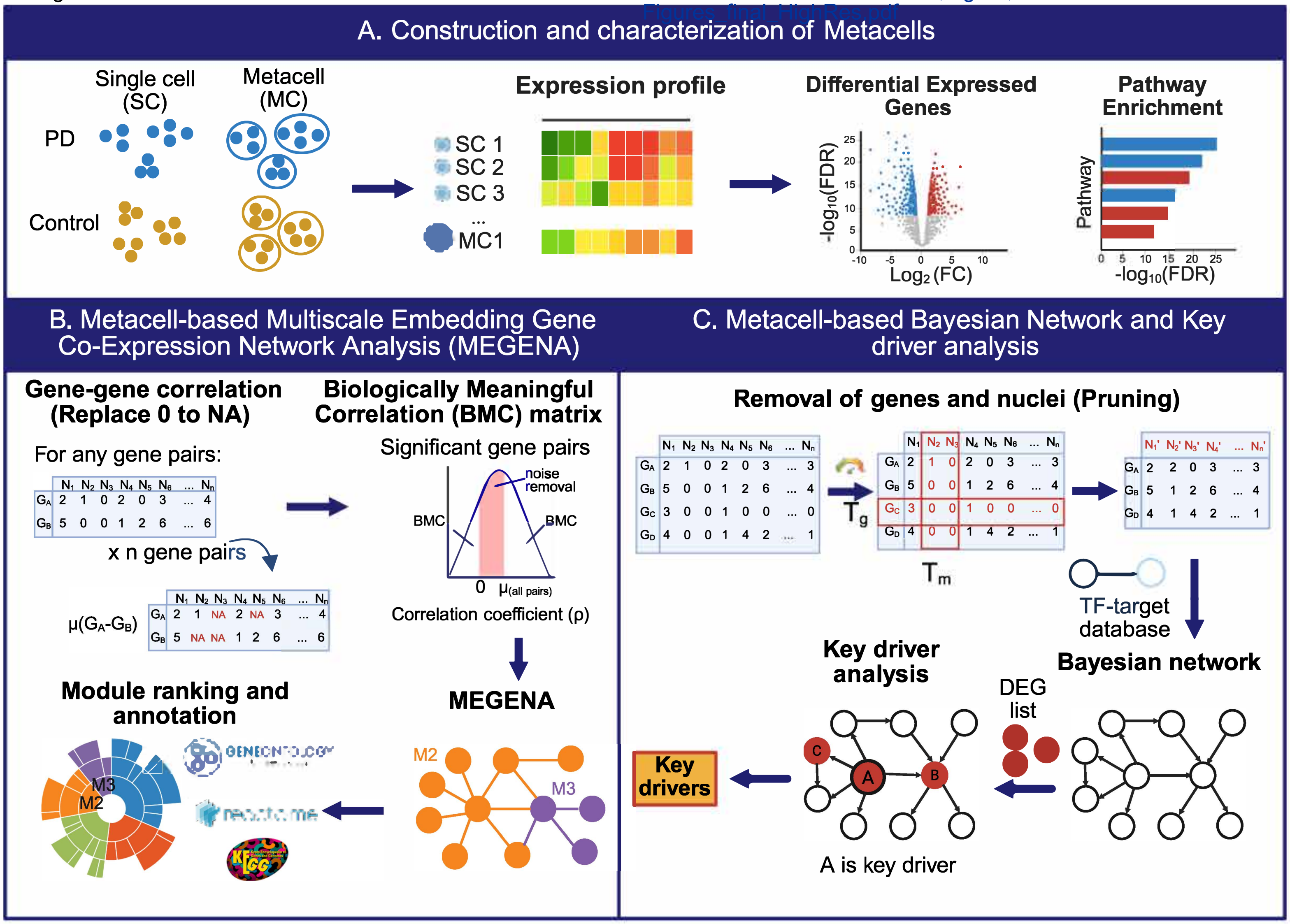

Figure 1

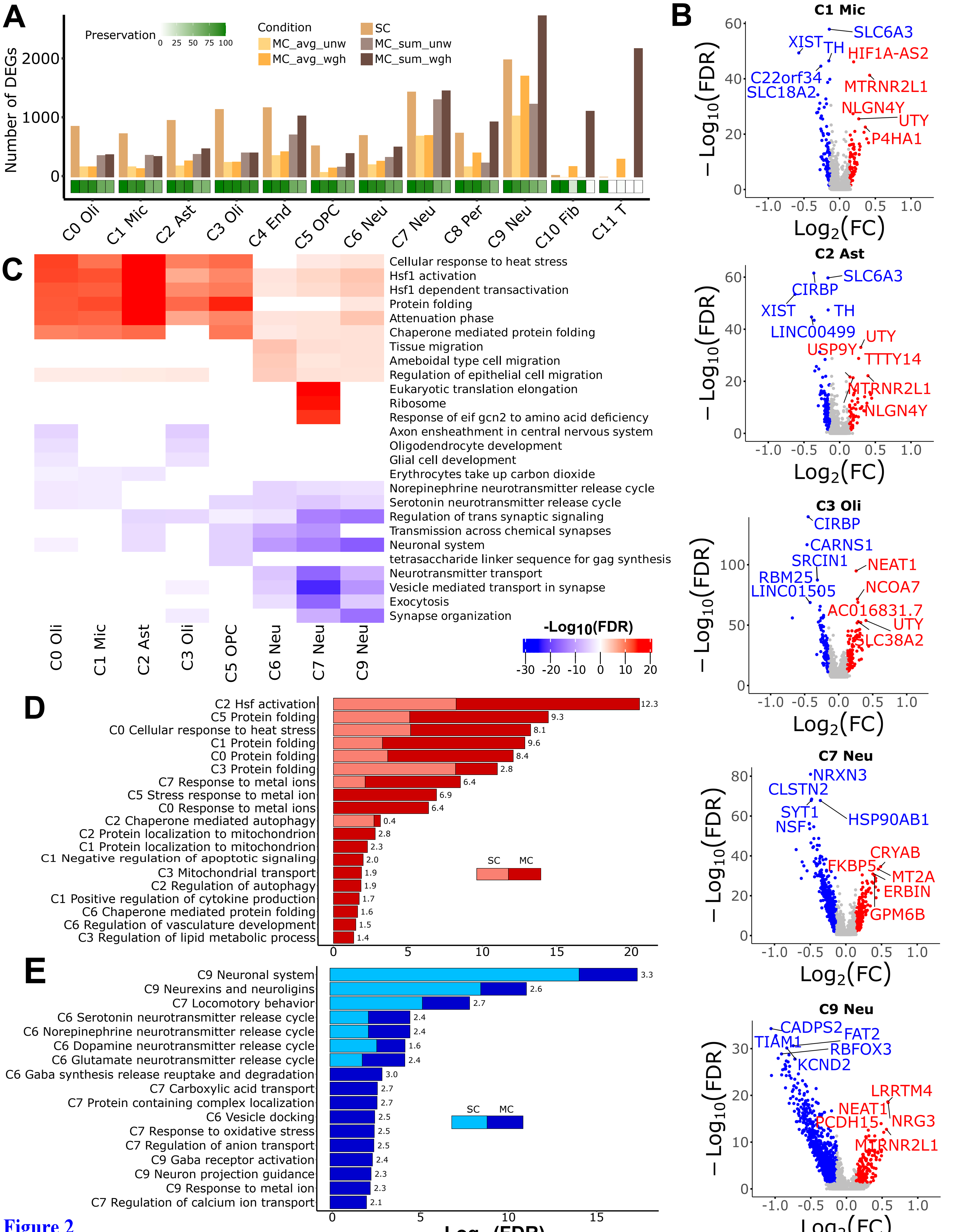
A
Preservation
0 25 50 75 100
Condition
MC_avg_unw
MC_avg_wgh
SC
MC_sum_unw
MC_sum_wgh
Number of DEGs
2000
1000
0
C0 Oli
C1 Mic
C2 Ast
C3 Oli
C4 End
C5 OPC
C6 Neu
C7 Neu
C8 Per
C9 Neu
C10 Fib
C11 T
B
C1 Mic
SLC6A3
XIST
TH
HIF1A-AS2
C22orf34
SLC18A2
MTRNR2L1
NLGN4Y
UTY
P4HA1
C2 Ast
SLC6A3
CIRBP
XIST
TH
LINC00499
UTY
USP9Y
TTTY14
MTRNR2L1
NLGN4Y
C3 Oli
CIRBP
CARNS1
SRCIN1
NEAT1
RBM25
LINC01505
NCOA7
AC016831.7
UTY
SLC38A2
C7 Neu
NRXN3
CLSTN2
SYT1
NSF
HSP90AB1
CRYAB
FKBP5
MT2A
ERBIN
GPM6B
C9 Neu
CADPS2
FAT2
TIAM1
RBFOX3
KCND2
LRRTM4
NEAT1
PCDH15
NRG3
MTRNR2L1
− Log10(FDR)
Log2(FC)
C
Cellular response to heat stress
Hsf1 activation
Hsf1 dependent transactivation
Protein folding
Attenuation phase
Chaperone mediated protein folding
Tissue migration
Ameboidal type cell migration
Regulation of epithelial cell migration
Eukaryotic translation elongation
Ribosome
Response of eif gcn2 to amino acid deficiency
Axon ensheathment in central nervous system
Oligodendrocyte development
Glial cell development
Erythrocytes take up carbon dioxide
Norepinephrine neurotransmitter release cycle
Serotonin neurotransmitter release cycle
Regulation of trans synaptic signaling
Transmission across chemical synapses
Neuronal system
tetrasaccharide linker sequence for gag synthesis
Neurotransmitter transport
Vesicle mediated transport in synapse
Exocytosis
Synapse organization
C0 Oli
C1 Mic
C2 Ast
C3 Oli
C5 OPC
C6 Neu
C7 Neu
C9 Neu
-Log10(FDR)
-30 -20 -10 0 10 20
D
C2 Hsf activation 12.3
C5 Protein folding 9.3
C0 Cellular response to heat stress 8.1
C1 Protein folding 9.6
C0 Protein folding 8.4
C3 Protein folding 2.8
C7 Response to metal ions 6.4
C5 Stress response to metal ion 6.9
C0 Response to metal ions 6.4
C2 Chaperone mediated autophagy 0.4
C2 Protein localization to mitochondrion 2.8
C1 Protein localization to mitochondrion 2.3
C1 Negative regulation of apoptotic signaling 2.0
C3 Mitochondrial transport 1.9
C2 Regulation of autophagy 1.9
C1 Positive regulation of cytokine production 1.7
C6 Chaperone mediated protein folding 1.6
C6 Regulation of vasculature development 1.5
C3 Regulation of lipid metabolic process 1.4
SC
MC
0 5 10 15 20
E
C9 Neuronal system 3.3
C9 Neurexins and neuroligins 2.6
C7 Locomotory behavior 2.7
C6 Serotonin neurotransmitter release cycle 2.4
C6 Norepinephrine neurotransmitter release cycle 2.4
C6 Dopamine neurotransmitter release cycle 1.6
C6 Glutamate neurotransmitter release cycle 2.4
C6 Gaba synthesis release reuptake and degradation 3.0
C7 Carboxylic acid transport 2.7
C7 Protein containing complex localization 2.7
C6 Vesicle docking 2.5
C7 Response to oxidative stress 2.5
C7 Regulation of anion transport 2.5
C9 Gaba receptor activation 2.4
C9 Neuron projection guidance 2.3
C9 Response to metal ion 2.3
C7 Regulation of calcium ion transport 2.1
SC
MC
0 5 10 15
-Log10(FDR)

Figure 2

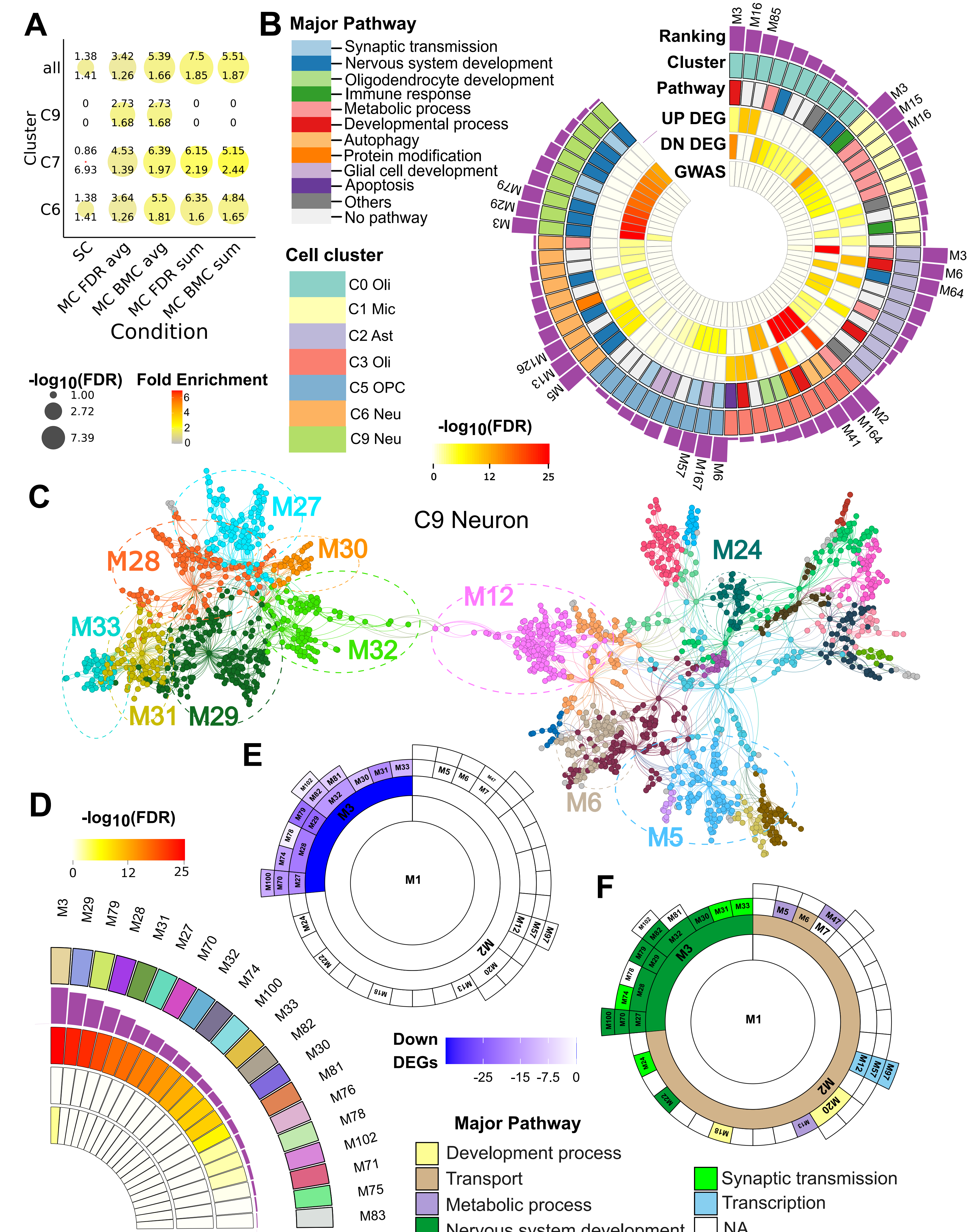
A
B
Major Pathway
Synaptic transmission
Nervous system development
Oligodendrocyte development
Immune response
Metabolic process
Developmental process
Autophagy
Protein modification
Glial cell development
Apoptosis
Others
No pathway
Cell cluster
C0 Oli
C1 Mic
C2 Ast
C3 Oli
C5 OPC
C6 Neu
C9 Neu
Ranking
Cluster
Pathway
UP DEG
DN DEG
GWAS
-log10(FDR)
Cluster
Condition
SC
MC FDR avg
MC BMC avg
MC FDR sum
MC BMC sum
-log10(FDR)
Fold Enrichment
C
C9 Neuron
M27
M28
M30
M33
M32
M12
M24
M31
M29
M6
M5
D
E
F
M1
M2
M3
Down DEGs
Major Pathway
Development process
Transport
Metabolic process
Nervous system development
Synaptic transmission
Transcription
NA
GWAS
UP
DOWN
RANK
MODULES

Figure 3

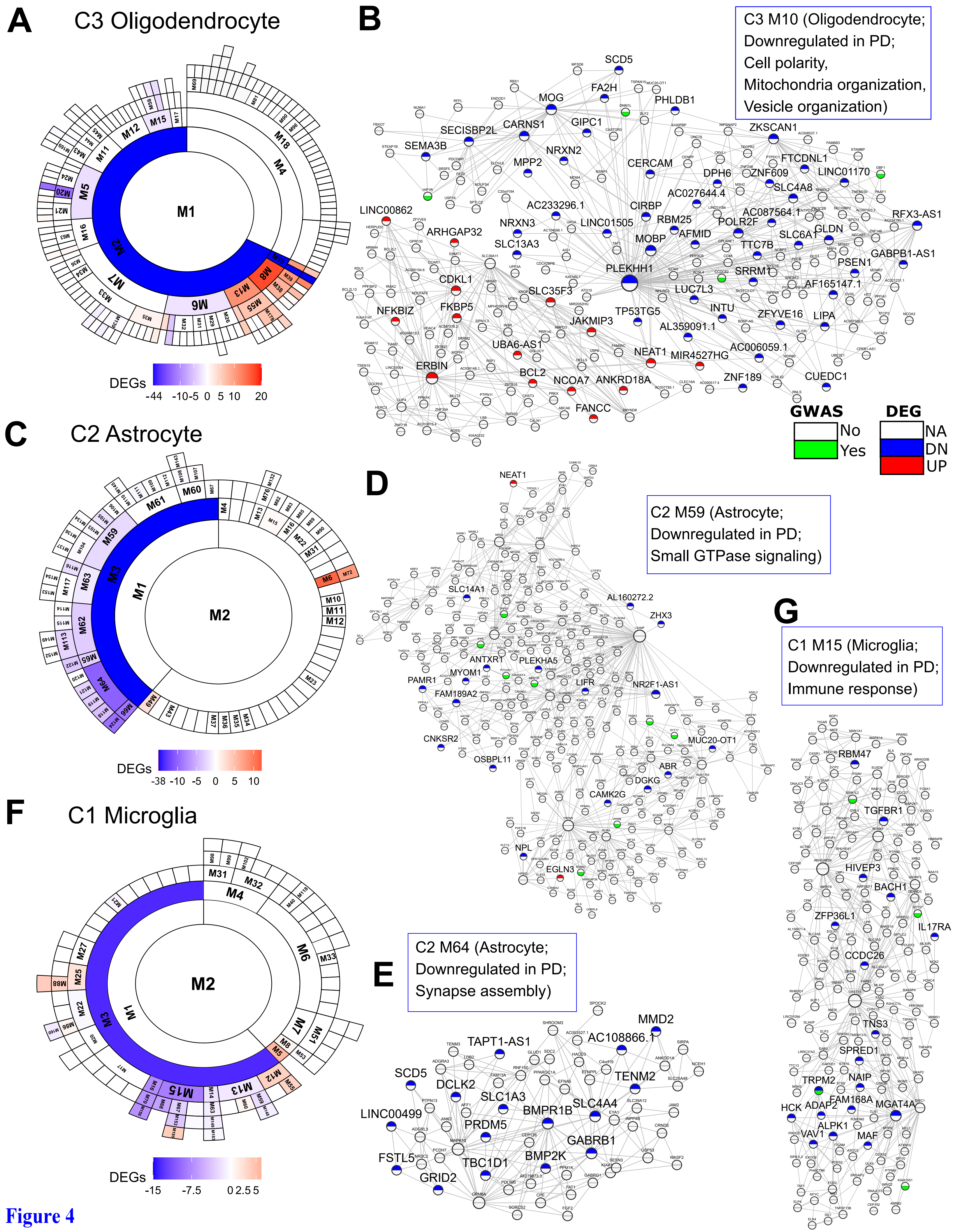
A
C3 Oligodendrocyte
M1
M2
M4
M5
M6
M7
M8
M11
M12
M13
M15
M16
M18
M20
DEGs
-44
-10
-5
0
5
10
20
B
C3 M10 (Oligodendrocyte;
Downregulated in PD;
Cell polarity,
Mitochondria organization,
Vesicle organization)
SCD5
FA2H
PHLDB1
MOG
SECISBP2L
CARNS1
GIPC1
SEMA3B
NRXN2
MPP2
CERCAM
ZKSCAN1
FTCDNL1
LINC01170
DPH6
ZNF609
SLC4A8
AC027644.4
AC087564.1
RFX3-AS1
LINC00862
ARHGAP32
AC233296.1
NRXN3
LINC01505
CIRBP
RBM25
POLR2F
AFMID
MOBP
SLC13A3
TTC7B
SLC6A1
GLDN
GABPB1-AS1
PSEN1
PLEKHH1
SRRM1
AF165147.1
CDKL1
SLC35F3
LUC7L3
NFKBIZ
FKBP5
TP53TG5
INTU
ZFYVE16
LIPA
JAKMIP3
AL359091.1
UBA6-AS1
AC006059.1
NEAT1
MIR4527HG
ERBIN
BCL2
NCOA7
ANKRD18A
ZNF189
CUEDC1
FANCC
GWAS
No
Yes
DEG
NA
DN
UP
C
C2 Astrocyte
M1
M2
M3
M6
M10
M11
M12
M59
M60
M61
M62
M63
M64
M65
M66
DEGs
-38
-10
-5
0
5
10
D
C2 M59 (Astrocyte;
Downregulated in PD;
Small GTPase signaling)
NEAT1
SLC14A1
AL160272.2
ZHX3
ANTXR1
PLEKHA5
MYOM1
PAMR1
LIFR
NR2F1-AS1
FAM189A2
CNKSR2
OSBPL11
MUC20-OT1
ABR
DGKG
CAMK2G
NPL
EGLN3
E
C2 M64 (Astrocyte;
Downregulated in PD;
Synapse assembly)
MMD2
TAPT1-AS1
AC108866.1
SCD5
DCLK2
TENM2
SLC1A3
SLC4A4
LINC00499
BMPR1B
PRDM5
GABRB1
FSTL5
TBC1D1
BMP2K
GRID2
F
C1 Microglia
M1
M2
M3
M4
M6
M7
M13
M15
M25
DEGs
-15
-7.5
0
2.5
5
G
C1 M15 (Microglia;
Downregulated in PD;
Immune response)
RBM47
TGFBR1
HIVEP3
BACH1
ZFP36L1
IL17RA
CCDC26
TNS3
SPRED1
TRPM2
NAIP
FAM168A
HCK
ADAP2
MGAT4A
VAV1
ALPK1
MAF
Figure 4

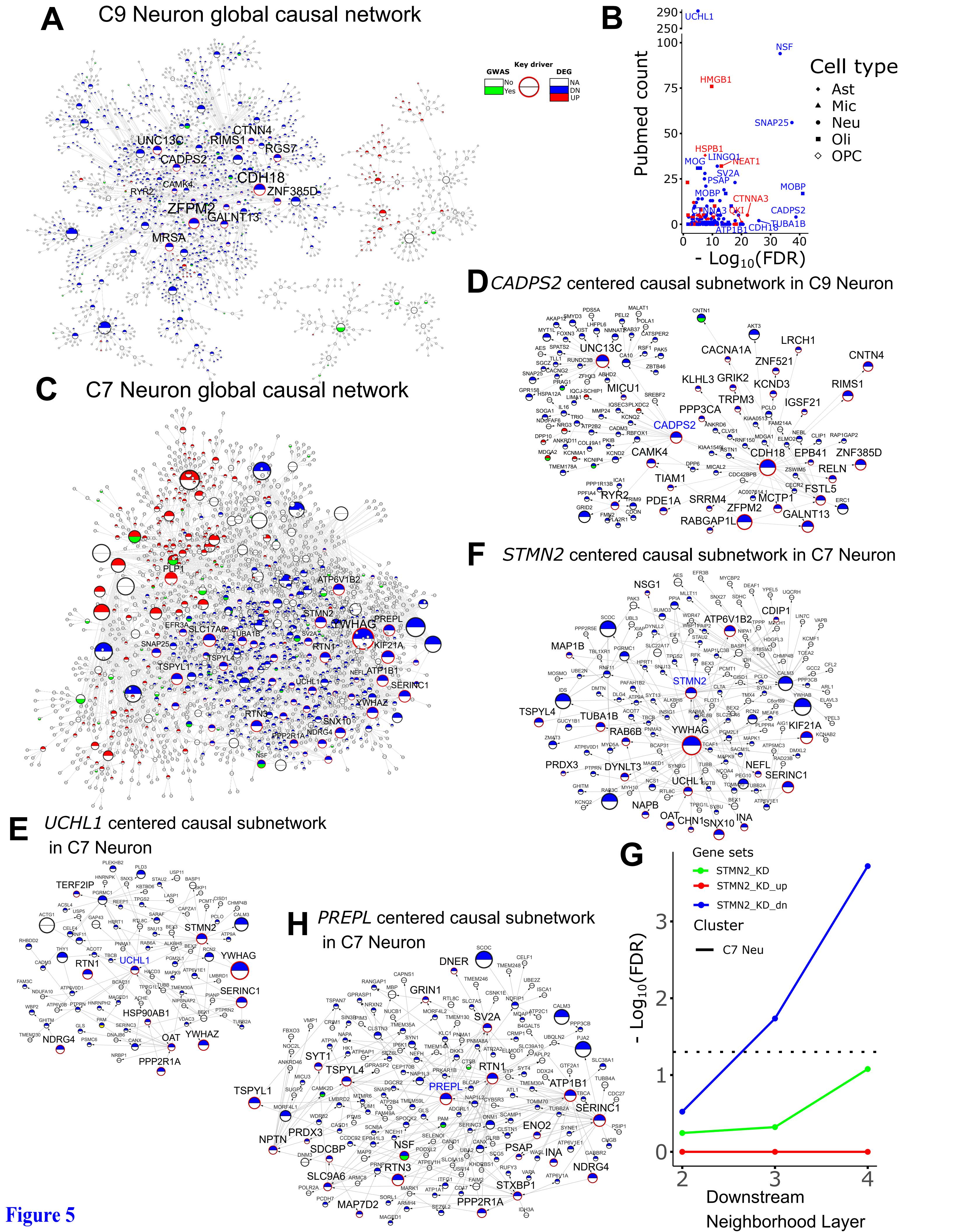
A C9 Neuron global causal network
GWAS
No
Yes
Key driver
DEG
NA
DN
UP
UNC13C
RIMS1
CTNN4
RGS7
CADPS2
CDH18
CAMK4
RYR2
ZNF385D
ZFPM2
GALNT13
MRSA
B
Pubmed count
- Log10(FDR)
Cell type
Ast
Mic
Neu
Oli
OPC
UCHL1
NSF
HMGB1
SNAP25
HSPB1
LINGO1
MOG
NEAT1
SV2A
PSAP
MOBP
CTNNA3
QKI
CADPS2
TUBA1B
ATP1B1
CDH18
C C7 Neuron global causal network
PLP1
ATP6V1B2
STMN2
YWHAG
PREPL
EFR3A
SLC17A6
TUBA1B
SNAP25
KIF21A
TSPYL4
TSPYL1
RTN1
NEFL
ATP1B1
UCHL1
SERINC1
YWHAZ
RTN3
SNX10
PPP2R1A
NDRG4
NSF
D CADPS2 centered causal subnetwork in C9 Neuron
E UCHL1 centered causal subnetwork in C7 Neuron
F STMN2 centered causal subnetwork in C7 Neuron
G
Gene sets
STMN2_KD
STMN2_KD_up
STMN2_KD_dn
Cluster
C7 Neu
- Log10(FDR)
Downstream Neighborhood Layer
H PREPL centered causal subnetwork in C7 Neuron

Figure 5

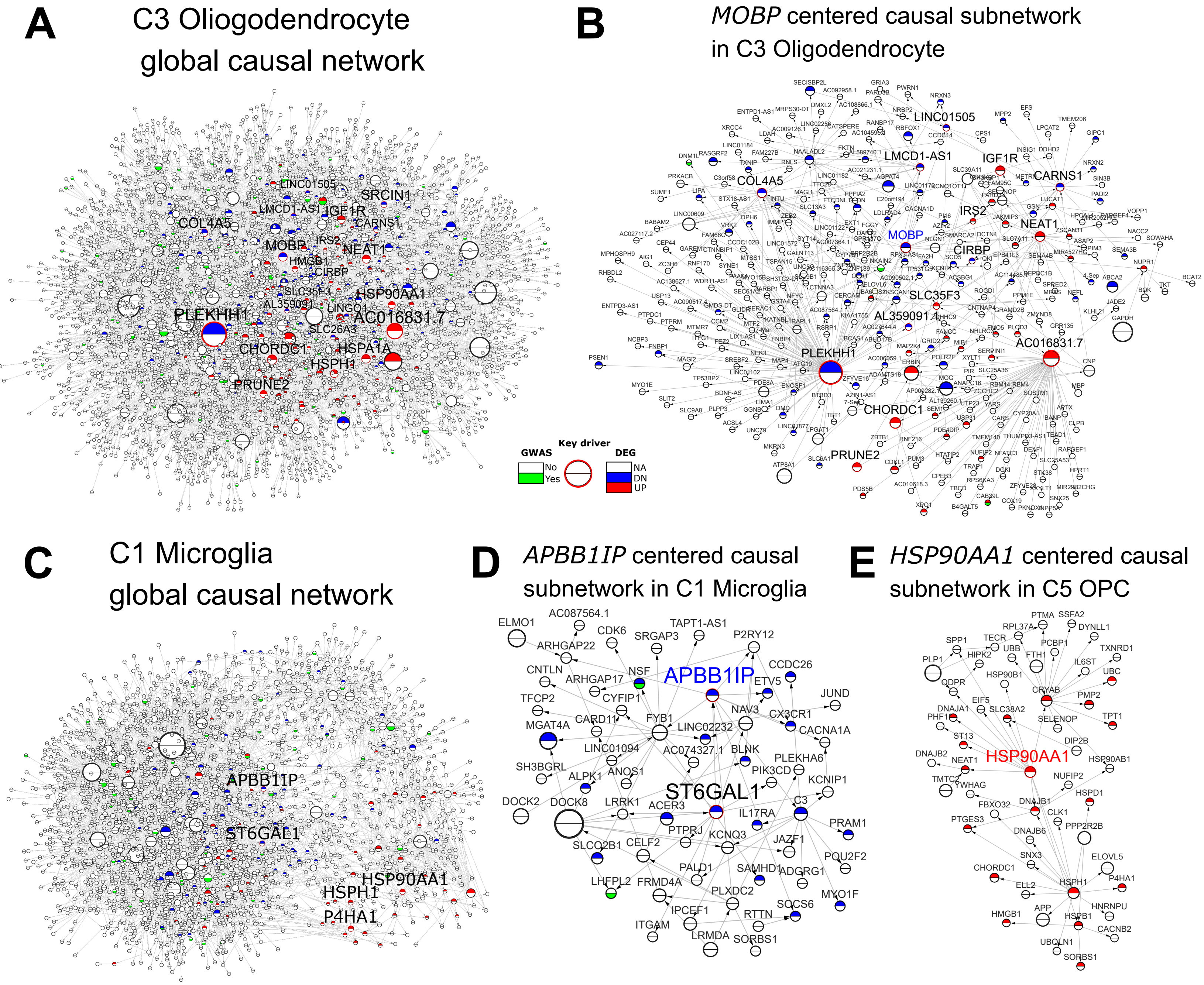
A
C3 Oliogodendrocyte
global causal network
LINC01505
SRCIN1
LMCD1-AS1
IGF1R
COL4A5
CARNS1
MOBP
IRS2
NEAT1
HMGB1
CIRBP
SLC35F3
HSP90AA1
AL359091
LINGO1
PLEKHH1
AC016831.7
SLC26A3
CHORDC1
HSPA1A
HSPH1
PRUNE2
B
MOBP centered causal subnetwork
in C3 Oligodendrocyte
LINC01505
LMCD1-AS1
IGF1R
CARNS1
COL4A5
IRS2
NEAT1
MOBP
CIRBP
SLC35F3
AL359091.1
PLEKHH1
AC016831.7
CHORDC1
PRUNE2
GWAS
No
Yes
Key driver
DEG
NA
DN
UP
C
C1 Microglia
global causal network
APBB1IP
ST6GAL1
HSP90AA1
HSPH1
P4HA1
D
APBB1IP centered causal
subnetwork in C1 Microglia
APBB1IP
ST6GAL1
E
HSP90AA1 centered causal
subnetwork in C5 OPC
HSP90AA1

Figure 6

Supplementary Figures

Click here to access/download
Supplementary Material
Figure Supplement_final_v3.pdf

Table S35

Click here to access/download

Supplementary Material

Tabel 35. DEP_ProteomicData2.csv

Table S34

Click here to access/download

Supplementary Material

Table 34. DEP_ProteomicData1.csv

Table S10

Click here to access/download

Supplementary Material

Table S10. FET_DEGs-Overlap_KAIST-and-MSSM.csv

Click here to access/download
Supplementary Material
Table S11. FET_DEGs-Overlap_Columbia-and-MSSM
.csv

Click here to access/download
Supplementary Material
Table S13. Summary_UpPathways_SC-and-MC.csv

Click here to access/download
Supplementary Material
Table S14. Summary_DnPathways_SC-and-MC.csv

Table S17

Click here to access/download

Supplementary Material

Table S17. FET)_STMN2-pathway-preservation.csv

Table S19

Click here to access/download
Supplementary Material
Table S19. GWAS_Gene-List.csv

Click here to access/download

Supplementary Material

Table S36. FET_DEGs-and-DEPs_ProteomicData1.csv

Table S37

Click here to access/download
Supplementary Material
Table S37. FET_DEGs-and-DEPs_ProteomicData2.csv

Table S9

Click here to access/download
Supplementary Material
Table S9. FET_DEGs-Overlap_VIB-and-MSSM.csv

Table S1

Click here to access/download
Supplementary Material
Table S1. DEGs-Metacell_MSSM-data.xlsx

Table S12

Click here to access/download
Supplementary Material
Table S12. Shared-Pathways_Metacell-and-SingleCell.xlsx

Click here to access/download

Supplementary Material

Table S15. UniquePathways_by-Metacell.xlsx

Table S16

Click here to access/download

Supplementary Material

Table S16. UniquePathways_by-SingleCell.xlsx

Table S18

Click here to access/download
Supplementary Material
Table S18. MemberGenes-in-Module.xlsx

Table S2

Click here to access/download
Supplementary Material
Table S2. Conserved Marker Genes_MSSM-data_by_Metacell.xlsx

Table S20

Click here to access/download
Supplementary Material
Table S20. Module-ranking_Pathway-annotation.xlsx

Click here to access/download
Supplementary Material
Table S21. FET_across All MEGENA Modules.xlsx

Table S22

Click here to access/download
Supplementary Material
Table S22. Pathwayed Enrichment for Modules.xlsx

Table S23

Click here to access/download

Supplementary Material

Table S23.Summary_Module-Preservation.xlsx

Click here to access/download
Supplementary Material
Table S24. FET_ModulePreservation.xlsx

Table S25

Click here to access/download

Supplementary Material

Table S25. ModulePreservation_PD-related-Modules.xlsx

Table S26

Click here to access/download
Supplementary Material
Table S26. FETModulePreservation_PD-related-modules.xlsx

Table S27

Click here to access/download
Supplementary Material
Table S27. KeyDriervGens.xlsx

Table S28

Click here to access/download
Supplementary Material
Table S28. Networks_Selected_Key Drivers.xlsx

Table S29

Click here to access/download

Supplementary Material

Table S29. Pathways_KeyDrivers-subnetwork.xlsx

Table S3

Click here to access/download
Supplementary Material
Table S3. Distribution_Metacell-Size_VIB-data.xlsx

Table S30

Click here to access/download
Supplementary Material
Table S30. KeyDrivers_Columbia-data.xlsx

Table S31

Click here to access/download
Supplementary Material
Table S31. KeyDrivers_shared_Columbia-MSSM.xlsx

Click here to access/download
Supplementary Material
Table S32. GWAS-Genes_subnetworks.xlsx

Click here to access/download
Supplementary Material
Table S33. Number-of-gene-pairs_before-and-after_BMC.xlsx

Click here to access/download
Supplementary Material
Table S4. DEGs-Metacell_VIB-data.xlsx

Table S5

Click here to access/download
Supplementary Material
Table S5. Distribution_Metacell-Sizes_KAIST-data.xlsx

Click here to access/download
Supplementary Material
Table S6. DEGs_Metacell_KAIST-data.xlsx

Table S7

Click here to access/download
Supplementary Material
Table S7. Distribution_MetacellSize_Columbia-data.xlsx

Click here to access/download
Supplementary Material
Table S8. DEG_Metacell_Columbia-data.xlsx

cover letter Click here to access/download;Personal Cover;coverletter.pdf

Icahn School of Medicine at Mount Sinai

Bin Zhang, PhD
Willard T.C. Johnson Research Professor of Neurogenetics
Professor, Departments of Genetics & Genomic Sciences, Pharmacological Sciences, and Artificial Intelligence and Human Health
Director, Mount Sinai Center for Transformative Disease Modeling
Icahn Institute of Genomics, Icahn School of Medicine at Mount Sinai

1425 Madison Avenue (Icahn 3-43)
Box 1498
New York, NY 10029
Phone: 212-659-1726
Fax: 212-659-5507
Email: bin.zhang@mssm.edu

June 14th, 2026

Dear Editor,

We are very pleased to submit our manuscript entitled "*Multiscale Transcriptomic Network Models of Parkinson's Disease at the Single Cell Level* " for consideration as a research article in *GigaScience.*

In Parkinson's Disease (PD), multiple single nuclei RNA-sequencing (snRNA-seq) studies of the vulnerable brain regions have revealed alterations in brain cell populations and cell type specific transcriptomes. However, a systematic analysis of cell-type–resolved gene regulatory architecture in PD is lacking. This study was built upon our multiscale network biology analysis of our single nucleus RNA-sequencing of the substantia nigra (SN) samples from postmortem brains of 32 donors, including 23 idiopathic Parkinson's disease (PD) subjects and 9 controls (*Science Advances* 10(2):eadi8287, 2024) and our collection of a large gene expression data in the substantia nigra from 161 control and Parkinson's disease (PD) brains (*Nature Communications* 10(1):5234, 2019) as well as our advanced transcriptomic and proteomic network modeling of Alzheimer's disease (AD) from over 3000 brain samples (Zhang et al., *Cell* 153(3):707-720, 2013; Wang et al., *Neuron* S0896-6273(20)30861-8, 2020; Wang et al., *Cell* (in press, Oct. 2025)).

To systematically uncover molecular mechanisms and identify therapeutic targets for PD, we develop an integrative, meta-cell based multiscale network analysis (MCMNA) of snRNA-seq data from the substantia nigra in PD. MCMNA overcomes the inherent sparsity of single cell data by leveraging metacell-based aggregation, enabling construction of robust gene co-expression and causal networks for identification of key drivers of PD progression. Gene co-expression network analysis identifies PD associated gene modules in major brain cell-types. Integration of meta-cell based differential gene expression and Bayesian causal network analysis systematically identifies putative driver genes and the top drivers including the calcium signaling gene *CADPS2*, synaptic transmission genes *PREPL* and *NSF*, the metabolic process gene *UCHL1*, the innate immune gene *APBB1IP*, and the heat shock stress response gene *HSP90AA1*. In particular, *STMN2* was previously identified and validated as a regulator of PD (*Nature Communications* 10(1):5234, 2019). *STMN2* is known to regulate microtube dynamic, axon formation and neurite outgrowth during development. The subnetwork around STMN2 also indicates the enrichment of pathways such as regulation of microtube depolymerization and polymeric cytoskeletal fiber. The subnetworks around *STMN2* are enriched for the knock-down signature of *STMN2* in the mouse substantia

Icahn School of Medicine at Mount Sinai

Bin Zhang, PhD
Willard T.C. Johnson Research Professor of Neurogenetics
Professor, Departments of Genetics & Genomic Sciences, Pharmacological Sciences, and Artificial Intelligence and Human Health
Director, Mount Sinai Center for Transformative Disease Modeling
Icahn Institute of Genomics, Icahn School of Medicine at Mount Sinai

1425 Madison Avenue (Icahn 3-43)
Box 1498
New York, NY 10029
Phone: 212-659-1726
Fax: 212-659-5507
Email: bin.zhang@mssm.edu

nigra that was generated from our previous study (*Nature Communications* 10(1):5234, 2019), validating the accuracy of our predicted metacell-based causal network.

In summary, the very first set of multiscale network models and putative key drivers identified from single cell transcriptomic data through this study lay down a foundation for the discovery of novel mechanisms and therapeutic targets for PD.

If there is any additional information we can provide to facilitate the review of this manuscript, please let us know.

Sincerely,

Bin Zhang, PhD
Willard T.C. Johnson Research Professor of Neurogenetics
Professor, Department of Genetics & Genomic Sciences
Professor, Department of Pharmacological Sciences
Professor, Department of Artificial Intelligence and Human Health
Director, Mount Sinai Center for Transformative Disease Modeling
Icahn Institute of Genomics
Icahn School of Medicine at Mount Sinai
