# Supplementary material for: Multiscale transcriptomic network models of Parkinson’s disease at the single-cell level
Source: Gigascience. 2026 Oct 6;15:giag096. doi: 10.1093/gigascience/giag096 (PMC13723071; doi:10.1093/gigascience/giag096)
Supplement: Supplementary file 3 [file giag096_giga-d-26-00192_revision_1.pdf]

GigaScience

Multiscale Transcriptomic Network Models of Parkinson’s Disease at the Single Cell Level

--Manuscript Draft--

**Manuscript Number:**	GIGA-D-26-00192R1
**Full Title:**	Multiscale Transcriptomic Network Models of Parkinson’s Disease at the Single Cell Level
**Article Type:**	Research
**Funding Information:**	National Institutes of Health (R01AG074010) — Prof. Bin Zhang<br>National Institutes of Health (R01AG085182) — Prof. Bin Zhang<br>Parkinson's Foundation (PF-RC-936279) — Dr. Zhenyu Yue<br>National Institutes of Health (S10OD026880) — Dr. Zhenyu Yue<br>National Institutes of Health (S10OD030463) — Dr. Zhenyu Yue
**Abstract:**	Multiple single nuclei RNA-sequencing (snRNA-seq) studies of the vulnerable brain regions of Parkinson’s Disease (PD) have revealed alterations in brain cell populations and cell type specific transcriptomes. However, a systematic analysis of cell-type–resolved gene regulatory architecture in PD is lacking. Here, we develop an integrative, meta-cell based multiscale network analysis (MCMNA) of snRNA-seq data from the substantia nigra to systematically uncover molecular mechanisms and identify potential therapeutic targets for PD. MCMNA overcomes the inherent sparsity of single cell data by leveraging metacell-based aggregation, enabling construction of robust gene regulatory networks. Gene co-expression network analysis identifies cell-type specific gene modules associated with PD. Integration of meta-cell based differential gene expression and a Bayesian causal network systematically reveals putative driver genes and their hierarchies. Our multiscale network models provide a framework for prioritizing candidate molecular regulators and pathways for further investigation of disease mechanisms and therapeutic targets.
**Corresponding Author:**	Bin Zhang, Ph.D.<br>Icahn School of Medicine at Mount Sinai<br>New York, New York UNITED STATES
**Corresponding Author Secondary Information:**	
**Corresponding Author's Institution:**	Icahn School of Medicine at Mount Sinai
**Corresponding Author's Secondary Institution:**	
**First Author:**	Chu Linh
**First Author Secondary Information:**	
**Order of Authors:**	Chu Linh<br>Gefei Yu<br>Xianxiao Zhou<br>Minghui Wang<br>Zhenyu Yue<br>Bin Zhang, Ph.D.
**Order of Authors Secondary Information:**	
**Response to Reviewers:**	We sincerely appreciate the very thoughtful and constructive comments from the two

reviewers, which provided us with valuable insights and opportunities to greatly improve the quality of the manuscript. We have revised the manuscript according to the reviewers' comments and provided a point-by-point response to each suggestion below.
Responses to the reviewers' comments are provided below with our responses given in blue. Please also see the cover letter and responses to comments PDF file which contains the same information here but is much easier to read.

Editor
GIGA-D-26-00192
Multiscale Transcriptomic Network Models of Parkinson's Disease at the Single Cell Level
Chu Linh; Gefei Yu; Xianxiao Zhou; Minghui Wang; Zhenyu Yue; Bin Zhang
GigaScience

Dear Prof. Zhang,

Your manuscript "Multiscale Transcriptomic Network Models of Parkinson's Disease at the Single Cell Level" (GIGA-D-26-00192) has been assessed by our reviewers. Although it is of interest, we are unable to consider it for publication in its current form. The reviewers have raised a number of points which we believe would improve the manuscript and may allow a revised version to be published in GigaScience.

Their reports, together with any other comments, are below. Please also take a moment to check our website at https://www.editorialmanager.com/giga/ for any additional comments that were saved as attachments.

In addition, please register any new software application in the bio.tools and SciCrunch.org databases to receive RRID (Research Resource Identification Initiative ID) and biotoolsID identifiers, and include these in your manuscript. Computational workflows should be registered in workflowhub.eu and the DOIs cited in the relevant places in the manuscript. These will facilitate tracking, reproducibility and re-use of your tool.

If you are able to fully address these points, we would encourage you to submit a revised manuscript to GigaScience. Once you have made the necessary corrections, please submit online at:

https://www.editorialmanager.com/giga/

If you have forgotten your username or password please use the "Send Login Details" link to get your login information. For security reasons, your password will be reset.

Please include a point-by-point within the 'Response to Reviewers' box in the submission system. Please ensure you describe additional experiments that were carried out and include a detailed rebuttal of any criticisms or requested revisions that you disagreed with. Please also ensure that your revised manuscript conforms to the journal style, which can be found in the Instructions for Authors on the journal homepage. If the data and code has been modified in the revision process please be sure to update the public versions of this too.

The due date for submitting the revised version of your article is 18 Oct 2026.

I look forward to receiving your revised manuscript soon.

Best wishes,

Weixue Mu
GigaScience
www.gigasciencejournal.com

Response #1: Thank you for this recommendation. The software/code developed for this study has already been archived in Zenodo and assigned a persistent DOI (10.5281/zenodo.20834299]), which is cited in the manuscript to ensure long-term

Powered by Editorial Manager® and ProduXion Manager® from Aries Systems Corporation

accessibility and reproducibility.

Reviewer #1:
Chu et al.'s manuscript offers a detailed Multiscale Transcriptomic Network Analysis (MCMNA) pipeline, which seeks to decipher the molecular mechanisms of Parkinson's Disease (PD) through the use of single-nucleus RNA-sequencing (snRNA-seq) data. By applying a metacell aggregation approach to cluster log-normalised gene expression matrices, the authors effectively address the inherent sparsity and noise of snRNA-seq datasets. The integration of Multiscale Embedded Gene Co-expression Network Analysis (MEGENA) with Bayesian probabilistic causal networks successfully identifies both known targets (such as UCHL1 and NSF) and novel putative driver genes (such as CADPS2 and WDR49) across major neuronal and glial cell types.
In summary, the study employs a robust methodology and offers a significant computational framework. However, certain analytical parameters, integration steps, and correlation assumptions require further clarification to ensure full reproducibility and robustness.
Response #2: We sincerely thank the reviewer for the highly positive evaluation of our study and for the insightful and constructive comments and suggestions.

Major Comments
1. The authors outline a method to tackle data sparsity by eliminating zero-values for every gene pair before computing Spearman correlation coefficients for the BMC matrix. While this strategy effectively enhances the overall correlation distribution in comparison to single-cell data, excluding all zero counts may artificially inflate correlation structures, potentially overlooking biologically important transcripts with low expression levels. The authors need to include an additional sensitivity analysis as supplementary material, showing that the complete exclusion of a particular set does not unintentionally favor genes that are universally highly expressed.
Response #3: We appreciated the important suggestion. To evaluate whether excluding zero-count unintentionally favor genes that highly expressed, we performed a sensitivity analysis examining the relationship between each gene's average expression level and its network activity (defined as the node degree in the MEGENA network). For each cluster, we calculated the average gene expression and correlated it with its node degree using Spearman's rank correlation. Across the cell clusters, the observed correlations were generally weak ($|\rho| < 0.2$), indicating that network connectivity was not strongly associated with baseline expression level. Only cluster 2 Astrocyte ($\rho = -0.239$) and cluster 7 Neuron ($\rho = -0.323$) showed modest negative correlation, suggesting that genes with lower average expression tended to have slightly higher network connectivity rather than the opposite. These findings indicate that excluding zero-count observations did not artificially bias the inferred co-expression network structure or preferentially enrich universally highly expressed genes. We have added these results in Figure Supplement 2D, and described them in the manuscript (Lines 198-204, Page 9).

2. The research employs the Harmony R package for merging data and the MAST package for identifying differentially expressed genes, while accounting for covariates including sex, age, and post-mortem interval (PMI). Considering the study independently reprocesses several external snRNA-seq datasets (the Lee, Martirosyan, and Ma datasets), the authors must clearly address how Harmony successfully reduced batch effects within these technologically and biologically diverse groups. Including a UMAP visualisation of the data before and after integration would greatly strengthen confidence in the alignment.
Response #4: This is a misunderstanding as the external snRNA-seq datasets were reprocessed independently using the same analysis pipeline and no cross-dataset batch correction or dataset merging was performed. Here, the consistency across studies was evaluated by comparing module and pathway-level results.

3. The reliability of network module validation through external datasets hinges on Fisher's Exact Test. Given that the FET necessitates stringent threshold standards for identifying gene set overlaps, the authors might overlook more subtle, rank-based preservation patterns across the datasets. Integrating a threshold-free metric like Gene Set Enrichment Analysis (GSEA) with FET might offer a more robust and comprehensive evaluation of module consistency across various neuronal and glial clusters.

Response #5: We thank the reviewer for this helpful suggestion. We agree that Fisher's Exact Test (FET), which relies on discrete gene-set overlap, may not capture more subtle patterns of network preservation across independent datasets. To provide a more comprehensive assessment and complement to our original analysis, we have now performed the WGCNA module preservation analysis at the cell type specific level in addition to FET. This approach evaluates whether the connectivity (if genes maintain similar relative connectivity patterns) and density structure of each module is preserved (if the genes in the module remain strongly interconnected in the test dataset) in an independent dataset, without requiring a predefined threshold for gene-set overlap.
We define a module as highly preserved if Zsummary.pres ≥ 10, moderately preserved if 2 < Zsummary.pres < 10, and not preserved otherwise. Similar to the previous FET results, moderate-to-high module preservation was highest in oligodendrocytes (43–53%), followed by microglia (50%), astrocytes (40%), and neurons (~8–44%). Overall, PD-associated modules showed greater moderate-to-high preservation. Consistent with the global findings, PD-associated modules showed the highest preservation in oligodendrocytes (87–97%), followed by microglia (95%), astrocytes (81%), OPCs (71%), and neurons (0–29%). Importantly, among neuronal clusters, PD-associated modules showed greater preservation in Cluster 9 and Cluster 6, but not in Cluster 7 (Supplemental Table S30-31).
These analyses provide an orthogonal assessment of the robustness and reproducibility of the identified network modules across datasets and align with the findings using our FET analysis. These analyses were added into the manuscript (Lines 991-1002, Pages 42-43; Lines 539-544, Page 23).

4. Bayesian causal networks were developed by incorporating transcription factor (TF)-target associations from the TFLink repository as priors. The authors should indicate if these priors were specifically curated for brain- or neural-derived functional genomic information, as transcription factor-target regulatory interactions can show substantial tissue variability.

Response #6: We agree that TF-target regulatory interactions exhibit tissue-specific activity. The TF-target interactions used as priors in our Bayesian causal network analysis were obtained from the TFLink database, which is not restricted to brain- or neural-specific regulatory interactions. Importantly, to our knowledge, there is currently no comprehensive, experimentally validated TF-target regulatory interaction database that is specific to the human substantia nigra or its constituent neuronal and glial cell types. Rather, TFLink is a comprehensive and continuously maintained repository that integrates experimentally validated and manually curated TF-target interactions from multiple public resources and the literature across diverse biological contexts. We selected TFLink because of its broad coverage, high-quality curation, and regular updates, providing a comprehensive set of prior regulatory relationships for Bayesian network inference. These priors were used to constrain the network structure rather than to define brain-specific regulatory programs. We clarified this in the revised manuscript (Lines 1005-1008, Page 43).

Minor Comments
1. Software Provenance: In the GigaScience submission checklist, the authors checked "No" in the Resources section, stating there are no experimental data. However, computational reproducibility requires exact software provenance. The authors should make sure to clearly state the exact version numbers of all R packages used (such as Seurat, SuperCell, MAST, Harmony) in the Methods section.
Response #7: We have updated our method section in the manuscript.

2. The authors observed that weighted aggregation did not consistently enhance downstream analyses while increasing data size, prompting them to choose the unweighted averaging method. Adding the statistical metric used would improve the transparency of the method.
Response #8: We have added supplementary tables (now Supplementary Table S1, S2) to improve the transparency of the method.

Reviewer #2:
Review of "Multiscale Transcriptomic Network Models of Parkinson's Disease at the Single Cell Level"
In this manuscript, the authors present an integrative, data-driven computational

framework (MCMNA) to delineate cell-type-specific gene regulatory networks in the substantia nigra of Parkinson's disease (PD). By combining metacell aggregation with co-expression network analysis (MEGENA) and Bayesian causal network inference, the authors seek to overcome the technical sparsity inherent to single-nucleus RNA sequencing (snRNA-seq) datasets and identify biologically meaningful regulatory modules and putative key drivers across neuronal and glial populations.

This manuscript presents a technically sophisticated computational framework that integrates metacell aggregation, co-expression network analysis and Bayesian causal inference to investigate cell-type-specific molecular networks in Parkinson's disease. The study represents a valuable resource for the field and identifies numerous hypotheses worthy of future investigation. However, several methodological issues—including potential ambient RNA contamination, incomplete validation of the metacell framework, statistical implications of averaging log-normalized expression, dependence on clustering resolution, and overinterpretation of causal and therapeutic implications—should be addressed before the biological conclusions can be fully supported. Addressing these concerns would substantially strengthen the manuscript and improve confidence in the proposed disease mechanisms and candidate regulators.
Response #9: We appreciate the positive evaluation of the study. Please see our responses to the major concerns below.
Major strengths
1. Rigorous analytical framework
The proposed pipeline directly addresses one of the major limitations of single-cell network inference—technical sparsity and dropout. The generation of a Biologically Meaningful Correlation (BMC) matrix to remove weak background correlations prior to co-expression analysis represents a thoughtful strategy to improve network robustness and reduce false-positive interactions.
2. Multiscale characterization of disease-associated networks
The study provides a detailed map of transcriptomic network organization across multiple neuronal and glial populations. In particular, distinguishing the network architecture of the vulnerable RIT2+ (c9) neuronal population from the more compact TH+ (c7) cluster provides potentially important insights into selective neuronal vulnerability in PD.
3. Integration of independent sources of evidence
The prioritization of co-expression modules using both differential expression and PD GWAS enrichment substantially strengthens the biological relevance of the identified networks. This integrative strategy increases confidence that the highlighted modules reflect disease-associated biology rather than statistical artifacts.
4. Identification of putative causal regulators
The Bayesian causal network analysis identifies 138 putative key drivers of PD. Recovery of previously implicated regulators, including STMN2, UCHL1 and NSF, provides confidence in the computational framework, while the identification of numerous novel, cell-type-specific candidates (for example CADPS2 in neurons and MOBP or APBB1IP in glial populations) offers an important resource for future experimental studies.

Major concerns
1. Potential ambient RNA contamination in glial populations
One of the most concerning observations is the presence of canonical dopaminergic neuronal markers (SLC6A3, TH and SLC18A2) among the most significantly downregulated genes in both microglia (c1) and astrocytes (c2) (Figure 2B). These genes are not expected to be expressed in glial populations under normal conditions. This raises the possibility of ambient RNA contamination, doublets, or cell-type misclassification.
The authors should clarify the origin of these signals and describe whether ambient RNA correction methods were applied. Because the downstream differential expression, pathway enrichment and network analyses rely on these gene lists, the robustness of the glial analyses depends on adequately addressing this issue.
Response #10: We thank the reviewer for raising this important concern regarding the detection of dopaminergic markers as differentially expressed genes in glial populations. Several observations suggested that these findings are unlikely to be primarily driven by cell-type misclassification. Cell type annotation was performed at the single-cell level, and the microglia and astrocyte populations retained robust

	expression of established lineage-specific glial markers without broad neuronal transcriptional signatures. In addition, doublets were carefully identified and removed during quality control, further reducing the likelihood that neuronal-glial doublets account for these signals in our previous publication1.<br>We acknowledge that that ambient RNA correction was not explicitly performed. However, TH, SLC6A3, and SLC18A2 were detected at relatively low levels in glial cells, and their identification as differentially expressed genes should not be interpreted as indicating high or canonical expression of these genes in glia. Notably, these genes were not significantly differentially expressed at the single-cell level but emerged in the metacell analysis. This is consistent with the increased statistical power of metacell approach, and therefore can reveal subtle disease-associated expression differences. Importantly, the presence of dopaminergic-associated transcripts in glial populations is not without precedent. Martirosyan et al. 2 reported TH-enriched microglial and astrocytic populations and observed their depletion in PD, providing independent evidence that TH-associated transcriptional signals can be detected in glial populations in the human substantial nigra. We have clarified these points in the revised manuscript (Lines 490-493 , Page 21).<br><br>2. Validation of the metacell strategy remains incomplete<br>The primary motivation for metacell aggregation is to improve signal-to-noise ratio and recover biological signals that are difficult to detect in conventional single-cell analyses. However, the current validation primarily demonstrates preservation of DEGs identified using standard single-cell approaches. While this establishes consistency, it does not demonstrate that the additional DEGs uniquely identified by the metacell approach represent genuine biological discoveries.<br>The authors should provide additional validation demonstrating that these unique findings reflect improved biological sensitivity rather than aggregation-induced artifacts. Replication in independent datasets, enrichment for established biological pathways, or orthogonal experimental validation would substantially strengthen this conclusion.<br>Response #11: We thank the reviewer for this important suggestion. To determine whether the additional DEGs detected by the metacell analysis represent biologically meaningful signals rather than aggregation-induced artifacts, we performed pathway-enrichment analysis specifically on the metacell-specific DEGs, which are genes identified as significant by the metacell approach but not by the conventional single-cell analysis (Supplementary Table S19)<br>The metacell-specific DEGs showed biologically coherent, cell-type-associated enrichment. Across the glial populations, these genes were enriched in pathways related to responses to cadmium and zinc ions and the regulation of ion homeostasis (driven by MT1M, MT1G, MT1X, MT1E) (Supplementary Table S20) Various studies have shown that changes in metal ion homeostasis may lead to damage to the CNS, in neurodegeneration, and in PD3. In neuronal populations, the metacell-specific genes were enriched in pathways associated with synaptic transmission, consistent with neuronal function.<br>We further compared these metacell-specific DEGs with DEG results from two independent datasets. This comparison identified recurrent genes (e.g. BAG3, DNAJA4, STIP1, FKBP4) involved the heat-shock response, providing independent support for the increased enrichment of stress-response pathways observed in meta cells. In addition, synaptic genes (e.g. GRIN2B, GAD1) identified in the Columbia's independent dataset overlapped with the metacell-specific gene set, consistent with the stronger enrichment of synaptic pathways detected by the metacell approach (Supplementary Table S21).<br>Together, these results indicate that the additional DEGs recovered by metacell analysis are not a random collection of genes. Instead, they exhibit cell-type-relevant functional coherence and partial reproducibility across independent datasets. Although these analyses cannot exclude all possible effects of aggregation, they support the conclusion that the metacell approach improves sensitivity for detecting biologically relevant signals that are less readily identified by conventional single-cell analysis.<br>We have added these analyses and the corresponding results to the revised manuscript (Lines 165-176, Pages 7-8; Lines 487-490, Lines 493 - 496, Page 21).<br><br>3. Averaging log-normalized expression may introduce statistical bias<br>The authors construct metacells by averaging log-normalized expression values ("avg") rather than summing raw counts followed by normalization ("sum"). Because logarithmic transformation is nonlinear, averaging expression values in log space may

artificially reduce within-metacell variance compared with aggregating raw counts before normalization.
Reduced variance could potentially inflate downstream statistical significance and contribute to the larger number of DEGs identified using the "avg" method. The authors should justify this methodological choice and demonstrate that their principal findings are robust to alternative metacell aggregation strategies.
Response #12: Contrary to the concern that averaging normalized expression values might artificially reduce variance and inflate differential expression, we observed the opposite trend in our data: the "sum" strategy consistently identified a larger number of DEGs than the "avg" strategy (Fig. 2A, Figure S1A-B; Lines 120-132, Page 6). Therefore, our empirical results do not support the hypothesis that the "avg" approach inflates statistical significance through variance reduction.
We selected the "avg" approach because it produced results that were more consistent with the original single-cell analyses. Specifically, the differential expression patterns and downstream biological interpretations derived from metacells generated using the averaging strategy showed greater concordance with those obtained from the original single-cell data, suggesting that this approach better preserves the underlying biological signals while reducing technical sparsity (Supplementary Fig. 2A-C, Supplementary Table S2).
Our choice is also consistent with the recommendations of the developers of the SuperCell framework, who suggest that averaging normalized expression values is appropriate when metacells are used for downstream analyses such as visualization, cell annotation, co-expression analysis, gene regulatory network inference, and trajectory analysis, as this strategy avoids repeating upstream normalization and scaling procedures while preserving biologically meaningful relationships.

4. Limited cell-type resolution
The analyses rely on relatively broad cell-type clusters, many of which have been shown by recent single-cell studies to contain multiple transcriptionally distinct subpopulations. Since metacell construction depends on the initial clustering, the resulting co-expression and Bayesian networks may be sensitive to clustering resolution.
For example, would the conclusions differ if multiple microglial subpopulations were analyzed independently? Similarly, because PD is associated with selective degeneration of dopaminergic neurons and reactive expansion of certain glial populations, the authors should discuss how changes in cell abundance across disease states might influence metacell construction and downstream network inference.
Response #13: Our study intentionally performed metacell construction within the major annotated cell types rather than finer subpopulations for two reasons.
First, reliable co-expression and Bayesian network inference require a relatively large number of cells to generate robust metacells and stable correlation estimates. Further subdividing already relatively small cell populations would substantially reduce the number of cells available for metacell construction, thereby decreasing statistical power and increasing the risk of unstable network inference. However, the proposed metacell-based network analysis can be performed on any cell subpopulation with a sufficient number of cells in larger single-cell or single-nucleus RNA-seq datasets that will be generated for PD in the future.
Second, our primary objective was to identify cell-type-specific rather than cell-state-specific regulatory networks that are reproducible across multiple independent PD cohorts. Using major cell types provided a consistent annotation framework across datasets despite differences in sequencing depth, sample composition, and clustering resolution.
In our framework, metacells were constructed within each annotated cell type, thereby minimizing the direct influence of changes in global cell-type composition on metacell construction. Consequently, differences in the abundance of one cell type (e.g., dopaminergic neurons) do not directly affect the construction of metacells for another cell type (e.g., microglia or astrocytes).

5. Interpretation of causal mechanisms and therapeutic targets
A conceptual limitation of the study is that differential gene expression largely reflects disease-associated transcriptional changes, many of which are likely secondary consequences rather than primary drivers of neurodegeneration. Although the Bayesian framework attempts to infer causal relationships, the current study remains

Powered by Editorial Manager® and ProduXion Manager® from Aries Systems Corporation

computational.
Accordingly, the concluding statement in the abstract that "The multiscale network models developed here lay down a foundation for the discovery of novel mechanisms and therapeutic targets for PD" appears stronger than is currently supported by the presented data. While the framework successfully prioritizes candidate regulators, no experimental evidence is provided demonstrating that these pathways contribute causally to dopaminergic neurodegeneration or represent tractable therapeutic targets. The authors should moderate these conclusions accordingly.
Response #14: We thank the reviewer for this important comment. We agree that disease-associated transcriptional changes may reflect primary drivers, downstream consequences, or compensatory responses, and that our Bayesian network provides computational evidence rather than experimental proof of causality. We have therefore moderated the language in the Abstract and revised the Discussion to emphasize the need for future perturbation studies in relevant in vitro and in vivo models to experimentally validate the predicted key drivers in PD and assess their therapeutic potential (Lines 624-626, Pages 26-27).

Specific comments
1. Figure 1: Please define all variables and abbreviations used in the schematic (e.g., μ, G, N and TF). The figure legend should more explicitly describe each analytical step.
Response #15: We thank the reviewer for this comment. We have updated the figure legend.

2. Please write out the full term for FET in the Figure S1 legend and provide references for the VIB, KAIST and Columbia datasets.
Response #16: We thank the reviewer for this comment. We have updated the figure legend.

3. In Figure S3B, μ(SC) appears to be missing.
Response #17: Due to high sparsity and small sample size, a co-expression network of the neuron Cluster 9 was unable to be constructed. Therefore, there is no correlation distribution of network in Cluster 9 in Figure S3B.

4. The manuscript uses PD GWAS loci to prioritize network modules. Because most GWAS variants reside in non-coding regions, the authors should clarify how variants were assigned to genes. Were loci mapped using nearest-gene assignment, eQTL colocalization, chromatin interaction data (Hi-C), TWAS, or another functional annotation strategy?
Response #18: In this study, we did not perform de novo mapping of GWAS variants to genes. Instead, we used a published, manually curated PD GWAS gene resource, in which GWAS loci had already been assigned to genes based on the methodology described by the original study. Therefore, gene assignment was determined by the curation strategy of the published database rather than by nearest-gene mapping or an in-house functional annotation pipeline.

5. The mathematical definition of the summary score is insufficiently described. Please provide the explicit equation used to combine DEG and GWAS enrichment statistics, including the weighting of each component.
Response #19: We have included the equation in the method section to describe our methodology (Lines 976-980, Page 42).

$$\text{Module Score}_m = \frac{-\log_{10}\left(P_{(adj,m)}^{(\text{Upregulated DEG})}\right) + -\log_{10}\left(P_{(adj,m)}^{(\text{Downregulated DEG})}\right) + -\log_{10}\left(P_{(adj,m)}^{GWAS}\right)}{3},$$

where $P_{(adj,m)}^{(\text{Upregulated DEG})}$, $P_{(adj,m)}^{(\text{Downregulated DEG})}$, and $P_{(adj,m)}^{GWAS}$are the adjusted P-values from the FET enrichment test for the DEGs and GWAS signatures, respectively, for module m. Higher scores indicate modules with stronger combined enrichment for disease associated transcriptional and genetic signals.

6. The manuscript identifies cluster c9 as a highly vulnerable neuronal population characterized by RIT2 expression. Recent human substantia nigra snRNA-seq studies have consistently identified SOX6+/AGTR1+ dopaminergic neurons as the population exhibiting the greatest vulnerability in PD. Does cluster c9 (or its dysregulated M3 module) significantly overlap with these established vulnerable neuronal signatures, or does RIT2 define an independent axis of selective vulnerability? Could the authors

Powered by Editorial Manager® and ProduXion Manager® from Aries Systems Corporation

comment on this in the discussion?
Response #20: We thank the reviewer for raising this important point. Previous studies using midbrain datasets and mouse models have identified SOX6+/AGTR1+ dopaminergic neuronal populations as particularly vulnerable in PD. We examined these markers in our results, however, neither SOX6 nor AGTR1 was identified as a defining feature of cluster c9 or as a gene member of the dysregulated M3 module (Supplementary Table S23). Therefore, based on the current dataset, we do not find evidence that the RIT2-associated c9 population directly corresponds to the previously described SOX6+/AGTR1+ vulnerable dopaminergic population.
Importantly, our study directly profiles the human substantia nigra, a primary site of dopaminergic neuronal degeneration and a region central to PD pathology, providing a complementary disease-relevant context to findings derived from broader midbrain datasets and mouse models. The absence of the SOX6+/AGTR1+ signature in c9 therefore raises the possibility that RIT2 and the M3 network capture an additional axis of selective neuronal vulnerability that is particularly relevant to the human substantia nigra. Nevertheless, differences in species, anatomical sampling, cellular composition, and analytical resolution may contribute to the observed differences, and the lack of overlap alone does not establish that these vulnerability mechanisms are independent. Further investigation, including direct integration with larger substantia nigra single-nucleus datasets and experimental characterization of RIT2-expressing neuronal populations, will be needed to determine the relationship between these vulnerability signatures. We have added this point to the Discussion (Lines 516-525, Page 22-23).

7. Construction of the Bayesian causal networks involved removing genes expressed in fewer than 40% of metacells (Tg = 0.4). While this filtering step may improve computational stability, it likely biases the resulting networks against lowly expressed transcription factors and other regulatory genes. The authors should report how many known PD-associated transcription factors were removed during this pruning step and discuss the resulting bias in their key driver analysis.
Response #21: We thank the reviewer for raising this important point. To assess whether the Tg = 0.4 pruning threshold disproportionately excluded transcriptional regulators, we quantified the removal of known transcription factors (TFs) and compared it with the removal of genes overall. Across all of the cell types, the percentages of removed TF, were similar to, and in some cases, slightly lower than, the overall percentage of genes removed during the pruning process (Supplementary Table S34), suggesting that the Tg = 0.4 filtering step did not preferentially deplete known PD-associated TFs relative to the background gene set. Nevertheless, we agree that an expression-based threshold inherently reduces representation of genes with sparse or low expression, including individual lowly expressed regulatory genes. Consequently, such regulators cannot be evaluated as potential key drivers if they are excluded before Bayesian network construction. We have therefore added the number and proportion of TFs removed by the filtering step and clarified this limitation in the revised manuscript (Lines 337-341, Page 14).

8. Approximately 70% of the predicted key drivers are described as novel. Validation based primarily on recovery of a single experimentally supported regulator (STMN2) provides only limited confidence in the broader set of newly identified candidates. Additional benchmarking using independent perturbation datasets, CRISPR screens, published regulatory networks or external validation datasets would substantially strengthen confidence in these predictions.
Response #22: We thank the reviewer for this important comment. We agree that validation based primarily on STMN2 provides limited support for the broader set of novel key drivers. Currently, large-scale CRISPR screening and perturbation datasets in PD-relevant neuronal models, particularly dopaminergic neurons, remain limited, restricting systematic independent validation of these predictions. We have acknowledged this limitation in the Discussion and emphasized that future studies using CRISPR screens and other functional perturbation approaches in relevant cellular and in vivo models will be important for validating the prioritized key drivers and establishing their roles in PD pathogenesis (Lines 619-626, Pages 27).

Editor Comments:
GigaScience has also published a number of relevant papers that may help contextualize your work. We have listed several recent examples below for your reference. Citing some of these articles, where appropriate in the Background or

	Discussion, would help frame your contribution within ongoing community efforts and strengthen the presentation of related methods and findings.<br>1. Bhattacharya, et al. Celltype transcriptomic modules reveal shared molecular mechanisms in Alzheimer's and Parkinson's d. GigaScience. 2026. https://doi.org/10.1093/gigascience/giag059/8689923.<br>2. Liu, et al. Multi-omics and high-spatial-resolution omics: deciphering complexity in neurological disorders. GigaScience. 2025. https://doi.org/10.1093/gigascience/giaf137/8371776.<br>Response #23: We thank the editor for bringing these relevant studies to our attention. We have incorporated the findings from Bhattacharya et al. into our discussion.<br><br>References<br>1.Wang Q, Wang M, Choi I, et al. Molecular profiling of human substantia nigra identifies diverse neuron types associated with vulnerability in Parkinson's disease. Science advances. 2024;10(2):eadi8287.<br>2.Martirosyan A, Ansari R, Pestana F, et al. Unravelling cell type-specific responses to Parkinson's Disease at single cell resolution. Molecular neurodegeneration. 2024;19(1):1-24.<br>3.Bhattacharya A, Fon EA, Dagher A, et al. Cell type transcriptomic modules reveal shared molecular mechanisms in Alzheimer's and Parkinson's disease. GigaScience. 2026;15:giag059.
**Additional Information:**	
**Question**	**Response**
Are you submitting this manuscript to a special series or article collection?	No
**Experimental design and statistics**<br><br>Full details of the experimental design and statistical methods used should be given in the Methods section, as detailed in our Minimum Standards Reporting Checklist. Information essential to interpreting the data presented should be made available in the figure legends.<br><br>Have you included all the information requested in your manuscript?	Yes
**Resources**<br><br>A description of all resources used, including antibodies, cell lines, animals and software tools, with enough information to allow them to be uniquely identified, should be included in the Methods section. Authors are strongly encouraged to cite Research Resource Identifiers (RRIDs) for antibodies, model organisms and tools, where possible.	No

Powered by Editorial Manager® and ProduXion Manager® from Aries Systems Corporation

Have you included the information requested as detailed in our Minimum Standards Reporting Checklist?	
If not, please give reasons for any omissions below.<br><br>as follow-up to "**Resources**<br><br>A description of all resources used, including antibodies, cell lines, animals and software tools, with enough information to allow them to be uniquely identified, should be included in the Methods section. Authors are strongly encouraged to cite Research Resource Identifiers (RRIDs) for antibodies, model organisms and tools, where possible.<br><br>Have you included the information requested as detailed in our Minimum Standards Reporting Checklist?<br><br>"	There is no experimental data in this study
**Availability of data and materials**<br><br>All datasets and code on which the conclusions of the paper rely must be either included in your submission or deposited in publicly available repositories (where available and ethically appropriate), referencing such data using a unique identifier in the references and in the "Availability of Data and Materials" section of your manuscript.<br><br>Have you have met the above requirement as detailed in our Minimum Standards Reporting Checklist?	Yes
GigaScience has policies and guidelines in place for the use of generative AI-writing tools such as ChatGPT. If you have used such writing tools to assist with writing the manuscript this must be declared and cited in the text. Authors should not list AI-writing tools and other	No

AI-assisted technologies as an author or co-author and should acknowledge that they are fully responsible for text generated or refined by AI-writing tools.<p><br><br>A summary of use (particularly in the introduction or among methods) needs to be included at the end of the paper, and the outputs should also be included as a supplementary file hosted in GigaDB or other open repositories. Please read our guidelines for more information. <p><br><br>By submitting to GigaScience, you are aware of the journal's AI-writing tools policy, and if you have declared use of such tools below, you have acknowledged this where appropriate in your manuscript and have made a summary of use and outputs available. <p><br><b>AI-assisted writing tools have been used in the preparation of this manuscript?	

Manuscript

Click here to access/download;Manuscript;PD_metacell-networks_v8.3.clean.docx

Multiscale Transcriptomic Network Models of Parkinson's Disease at the Single Cell Level

Linh Chu[1,2,3,4], Gefei Yu[1,2,5], Xianxiao Zhou[1,2,5], Minghui Wang[1,2,5], Zhenyu Yue[3,6,7,8], and Bin Zhang[1,2,4,5,9]*

[1]Department of Genetics and Genomic Sciences, Icahn School of Medicine at Mount Sinai, 1425 Madison Avenue, NY 10029, USA

[2]Mount Sinai Center for Transformative Disease Modeling, Icahn School of Medicine at Mount Sinai, 1470 Madison Avenue, NY 10029, USA

[3]Department of Neurology, Icahn School of Medicine at Mount Sinai, 1425 Madison Avenue, NY 10029, USA

[4]Department of Pharmacological Sciences, Icahn School of Medicine at Mount Sinai, 1425 Madison Avenue, NY 10029, USA

[5]Icahn Institute of Genomics, Icahn School of Medicine at Mount Sinai, 1425 Madison Avenue, NY 10029, USA.

[6]Nash Family Department of Neuroscience, Icahn School of Medicine at Mount Sinai, 1470 Madison Avenue, NY 10029, USA

[7]Friedman Brain Institute, Icahn School of Medicine at Mount Sinai, 1470 Madison Avenue, NY 10029, USA

[8]Center of Parkinson's Disease Neurobiology, Icahn School of Medicine at Mount Sinai, 1470 Madison Avenue, New York, NY, USA

[9]Department of Artificial Intelligence & Human Health, Icahn School of Medicine at Mount Sinai, 1470 Madison Avenue, NY 10029, USA

Corresponding author:
Bin Zhang, PhD
Professor, Department of Genetics & Genomic Sciences, Icahn Institute of Genomics
Icahn School of Medicine at Mount Sinai

1425 Madison Avenue, Room 3-43

New York, NY 10029

(O) 212-659-1726

Email: bin.zhang@mssm.edu

Linh Chu [0000-0001-7463-5947], Gefei Yu [0009-0001-4238-5100], Xianxiao Zhou [0000-0001-9350-4467], Minghui Wang 0000-0001-9171-4962], Zhenyu Yue [0000-0001-8730-8515] and Bin Zhang [0000-0002-9549-5653]

SUMMARY

Multiple single nuclei RNA-sequencing (snRNA-seq) studies of the vulnerable brain regions of Parkinson's Disease (PD) have revealed alterations in brain cell populations and cell type specific transcriptomes. However, a systematic analysis of cell-type–resolved gene regulatory architecture in PD is lacking. Here, we develop an integrative, meta-cell based multiscale network analysis (MCMNA) of snRNA-seq data from the substantia nigra to systematically uncover molecular mechanisms and identify potential therapeutic targets for PD. MCMNA overcomes the inherent sparsity of single cell data by leveraging metacell-based aggregation, enabling construction of robust gene regulatory networks. Gene co-expression network analysis identifies cell-type specific gene modules associated with PD. Integration of meta-cell based differential gene expression and a Bayesian causal network systematically reveals putative driver genes and their hierarchies. Our multiscale network models provide a framework for prioritizing candidate molecular regulators and pathways for further investigation of disease mechanisms and therapeutic targets .

Keywords: single-nucleus RNA-sequencing, Parkinson's disease, cell sparsity, GWAS, Metacells, Multiscale Embedded Gene Co-expression Network Analysis, Bayesian Network, key drivers

INTRODUCTION

Parkinson's disease (PD) is the second most prevalent neurodegenerative disorder, with both motor and non-motor symptoms(1, 2). The pathology of PD is characterized by the progressive loss of dopaminergic (DA) neurons in the substantial nigra (SN) and accumulation of Lewy bodies (LB), which contain abnormally α-synuclein protein aggregation(3, 4). Rare variants in over 20 genes account for 3-5% of PD cases in most populations, most of which are highly penetrant, and often known to be causal genes(5). To date, genome-wide association studies (GWAS) have identified 90 genetic risk variants, which collectively account for 16-36% of the heritable risk of non-monogenic PD(6). Given that most PD patients have no familial history or known PD mutations, there remains a critical gap in understanding how molecular signatures relate to disease mechanisms and therapeutic strategies in idiopathic PD. While the loss of DA neurons is a hallmark of the disease, microglial activation was first reported in PD brains in 1988(7), and has been supported by *in vivo* and *in vitro* studies(8-11). In addition, astrocyte reactivity has been prominently observed in the SN of PD patients, with research highlighting both neuroprotective and neurotoxic roles such as production of oxidative and nitrosative stress, inflammation, and contribution to the blood-brain barrier(12-15). Recently, studies have indicated oligodendrocyte dysfunction in PD, with changes pointing to altered inflammatory responses, protein folding stress, and myelination abnormalities(16-18).

Gene regulatory network (GRN) uncovers new pathways and gene targets in biological processes in human disease. Previously, our labs integrated a multiscale transcriptomic network analysis of PD SN samples by assembling the gene expression profiles from eight published bulk RNAseq studies to systematically identify intrinsic network structures and key regulators such as *STMN2* in PD(19). Bulk tissue datasets do not have cell-type level granularity, thus limiting the understanding of complex signaling across cell cell populations and within a population.Recently, single-cell/Single-nucleus RNA sequencing (sc/sn-RNAseq) have emerged as promising tools to unveil cell-level signaling pathways associated with neurodegenerative diseases including PD. We and others, have profiled single nuclei of human SN from

PD and healthy control, identifying changes in gene expression and new biological pathways at single cell levels(20-25). While single-cell datasets continue to expand in public repositories, little progress has been made toward the cell type specific GRN analysis for PD.

The challenges to construct single cell GRN inference mainly stem from the high sparsity in gene expression profile (large percentage of missing values or zeros) in snRNA-seq data. Many model-based imputation methods have been developed, yet the performance of these methods varies across different datasets(26). To address this challenge, recent studies have adopted the concept of "Metacells"(27) and developed several metacell algorithms(27-30) to not only compress data but also enhance the signal-to-noise ratio in expression matrices. This new approach results in higher clustering accuracy, improved annotation, and increased reliability in the identification of differentially expressed genes (DEGs). Despite the potential, these metacell algorithms have yet to be applied to gene network analysis in PD(31).

In this study, we develop an integrative metacell-based gene co-expression and causal network analysis framework and applied it to a recently published PD snRNAseq dataset in the human SN from 23 PD and 9 healthy controls(20). By adopting a systematic and data-driven approach in both co-expression and causal networks, we identify neuron- and glia - specific subnetworks that are dysregulated in PD. These cell type specific networks not only reveal putative key drivers for PD but also shed a light on the molecular mechanisms of PD. Notably, the top putative drivers include known targets of PD such as *UCHL1*, *NSF*, and *PREPL* in neurons, as well as novel candidate genes, such as *CADPS2*, *APBB1IP, HSP90AA1* in neurons or glial cells. In addition, PD driver genes (*STMN2* and *RALYL*) identified in our previously bulk RNAseq network analysis are also found to be key drivers in the respective neuronal single cell networks. Altogether, our study provides a framework for integrating diverse, cell type specific networks to pinpoint putative drivers of disease networks.

RESULTS

Integrative network biology framework for decoding molecular mechanisms of Parkinson's Disease at the single cell level

To systematically model single cell transcriptomic data in PD, we develop an integrative, meta-cell based multiscale network analysis (MCMNA) framework that includes metacell construction and characterization, co-expression network analysis, causal network inference, and key driver analysis (**Fig. 1**). In brief, we first group single cells into metacells – a group of cells with highly similar gene expression profiles (**Table 1**). To preserve the cell type annotation and phenotype information from snRNA-seq data, the metacell analysis is performed for each condition in each cell cluster. Single cell profiles within each metacell are aggregated to create metacell gene expression profile. Then, differentially expressed genes (DEGs) between the disease and control are identified, and functional enrichment analysis is performed on the DEGs to identify dysregulated pathways in PD (**Fig. 1A**). Next, we construct metacell-based co-expression analysis using Multiscale Embedded Gene co-Expression Network Analysis (MEGENA)(32) for each cell type to identify cell type specific co-expression modules (groups of highly co-expressed genes) (**Fig. 1B**). In parallel, we construct the global Bayesian probabilistic causal network (BN)(33, 34) and perform key driver analysis (KDA)(35) to predict potential key regulators, termed key drivers (KDs) of PD (**Fig. 1C**). A unique feature of MCMNA is that it addresses the sparsity of single cell gene expression data and increases the robustness of single cell network by the generation of metacells, construction of biologically meaningful correlation (BMC) matrix for co-expression network, and additional pruning of data matrix for causal network. Additionally, network models based on both PD and control allow us to capture early changes driving PD as such signals may not change across various disease stages.

In the remainder of the manuscript, we apply MCMNA to a single-nucleus RNA sequencing (snRNA-seq) dataset derived from substantia nigra (SN) samples of 23 idiopathic PD cases and 9 controls,

with an average age of 81, as described in a previous publication.(20). The study profiled a total of 315,867 high quality nuclei and identified 12 distinct cell clusters.

Metacells uncover cell type- specific gene expression enrichment between PD and control

At each cell cluster and condition (PD or control), single-cell profiles within each metacell are aggregated to generate metacell gene expression profiles, either by averaging the log-normalized single-cell expression data (average - "avg") or by summing the raw single-cell values followed by log normalization (sum). Additionally, the method allows for the inclusion of metacell size (the number of single cells in each metacell) as a weighting factor (weighted) or omitting it (unweighted). To explore global transcriptomic changes using metacell method, we first examine the number of DEGs in each cell cluster (at least 1.1-fold change (FC) and false discovery rate (adjusted p value less than 0.05) and calculate the percentage of preservation compared to the single-cell method. The average method demonstrates higher preservation compared to the sum method across the majority of the clusters (**Fig. 2A**, **Supplementary Fig. S1A-S1B**). While the number of DEGs is reduced in metacells compared to the single cell method, the number of conserved markers increases, potentially reflecting enhanced cell cluster specificity and reduced noise in metacells (**Supplementary Fig. S1C**). However, this observation has not been formally tested, and further studies are needed to confirm it.

While weighted aggregation can incorporate cell-level weight, it substantially increases the data size and computation time, without consistently improving downstream analyses such as differential expression andpathway enrichment (**Supplementary Fig. S1A-S1C**, **Supplementary Table S1**). Therefore, the unweighted method provides a good balance between efficiency and accuracy. Distinct gene signatures of five major brain cell types (e.g. neurons, microglia, astrocytes, oligodendrocytes and oligodendrocytes progenitor cells (OPCs)) are highlighted using the selected metacell method (average and unweighted) (**Fig. 2B**, **Supplementary Fig. S1E**, **Table 2, Supplementary Table S3**). Next, we compare our DEG signatures with other published snRNA-seq of human SN from three independent cohorts in PD(21, 22, 25). We reprocess the original datasets using our own analytical

pipeline to identify cell clusters (**Supplementary Table S4-10**, **Supplementary Fig. S1F-G, S1I-J, S1L-M**). We then assess the overlap of DEG signatures in each cell type using Fisher's exact test (FET). The results show that, for three datasets, there is a decrease in corrected p-value but an increase in fold enrichment, indicating more reduced statistical preservation strength but greater specificity in the preservation of DEG signatures across major cell cluster between the two datasets in metacells (**Supplementary Table S11-13**, **Supplementary Fig. S1H, Supplementary Fig. S1K, Supplementary Fig. S1N**).

Metacells reveal common and unique cell type- specific biological pathways between PD and control

Functional enrichment analysis is performed on the DEG signatures from both metacell and single cell methods. Using selected conditions (average and unweighted), we identify common and unique biological pathways. We observe that 10-30% and 0-60% shared pathways in major cell types from up-regulated and down-regulated DEGs, respectively (**Supplementary Table S14-16, Supplementary Figure S2A-B**). Similar to previously reported results, upregulated genes across cell types are involved in heat stress, Hsf1 activation, protein folding, and cell migration pathway. In contrast, downregulated genes are associated with nervous system development, synaptic transmission, neurotransmitter transports across cell types, and glial cell development in glial cells (**Figure 2C**). Many pathways unique to the single cell and metacell methods are also detected (**Supplementary Table S17-18**, **Supplementary Figure S2A-B**). Positive correlation of both up-regulated and down-regulated pathways across cell types indicates highly conserved pathways between the two methods. Additionally, several pathways showing significant difference in enrichments in the single cell and metacell based DEG signatures are highly relevant in PD biological pathways. These include protein folding in glial cells, autophagy in astrocytes, mitochondrial process in microglia and astrocytes, cytokine production in microglia, vasculature development, neurotransmitter release, calcium transport in neurons (**Figure 2D-E**).

Additional metacell-specific DEGs showed biologically coherent and cell-type associated functional enrichment. In glial populations, these genes were enriched for pathways related to metal ion response and ion homeostasis, driven in part by metallothionein genes (*MT1M*, *MT1G*, *MT1X*, and *MT1E*) (**Supplementary Table S19 - 20**). In neuronal populations, metacell-specific DEGs were predominantly enriched for pathways related to synaptic transmission. Comparison with DEG results from two independent datasets (Columbia dataset(25) and KAIST dataset(21)) further supported the biological relevance. Recurrently identified genes such as heat-shock and stress-response genes (*BAG3*, *DNAJA4*, *STIP1*, and *FKBP4*) consistent with the enhanced detection of stress-response pathways in the metacell analysis in glial population (**Supplementary Table S21**). Synaptic genes, including *GRIN2B* and *GAD1*, also overlapped with DEGs identified in the independent Columbia dataset(25), supporting the stronger enrichment of synaptic pathways detected using the metacell approach (**Supplementary Table S21**).

A novel framework to address single cell sparsity in co-expression network

To understand the interactions among genes, we construct gene co-expression networks to identify gene modules using MEGENA under the MCMNA framework (**Supplementary Figure S3A**). While metacell improves the gene-gene correlation, as noted in previous studies(27-30), gene-gene correlation still suffers from data sparsity. Therefore, zero counts are removed for each gene pair before calculating Spearman correlation coefficients (SCCs), leading to an increase in overall correlation distribution in metacells compared to single cell (**Supplementary Figure S3B**). To address inherent noise in sc/snRNA-seq data observed by the drift in the mean signed SCCs across cell types, we propose a framework to create a Biologically Meaningful Correlation (BMC) matrix at the optimal threshold of number of cells and genes (**Supplementary Figure S3A, C**). We perform FET analysis between *STMN2*-related genes and *STMN2* perturbation DEG signatures in PD mouse SN(19) across all clusters and three neuronal clusters. Compared to the metacell FDR only and single cell matrix only, BMC approach yields higher -log10(FDR) and fold enrichment in the MC-average comparison, and higher

fold enrichment in the MC-sum comparison (**Figure 3A, Supplementary Table S22**). These suggest that BMC matrix indeed reduces noise the weak positive correlation, resulting in a more consistent gene subset aligned with the STMN2 perturbation. Averaging log-normalized expression values indicate more statistically significant enrichment and is therefore used for downstream analysis (**Figure 3A**, **Supplementary Table S22**). Optimized BMC matrix is then subjected to MEGENA. Additionally, this method enables the construction of a gene co-expression network for the small neuron cluster 9 (c9), which was not possible due to high sparsity and small sample size. No MEGENA modules are found in the fibroblast cluster 10 and the T cells cluster 11.

To assess potential bias from excluding zero-count observations, we correlate each gene's average expression with its MEGENA connectivity (node degree) using Spearman's correlation. Correlations are weak across all clusters ($|\rho| < 0.2$; Supplementary Figure S3D), indicating that network connectivity is largely independent of baseline expression. Only Cluster 2 (astrocyte) and Cluster 7 (neuron) show moderate negative correlations (ρ = -0.2 to -0.3), suggesting that lower-expressed genes tend to have slightly higher connectivity. These results demonstrate that excluding zero-count observations do not preferentially favor highly expressed genes.

Metacell-based co-expressed networks reveal cell-type-specific functional gene modules

In total, we identify 1,057 significant gene modules (FDR adjusted P values less than 0.05) in all the cell types, and 833 modules in 5 major cell types (**Table 3, Supplementary Table S23**). To annotate biological functions associated with each module, functional enrichment analysis is performed. To prioritize gene modules associated with PD, a summary score is calculated based on the following FET enrichment tests: 1) enrichment for the DEG signatures between PD and control (Upregulated (UP) and Downregulated (DN) DEGs), and 2) enrichment for the PD genetic risk variants (PD GWAS catalogue) (**Supplementary Table S24**)(36). Given that the central nervous system consists of various cell types, each with distinct roles in PD pathogenesis and progression, we focus on the major brain cell types: neurons (cluster 6 (c6), cluster 7 (c7), and cluster 9 (c9), astrocytes (cluster 2 (c2)), microglia

(cluster 1 (c1)), oligodendrocyte precursor cells (OPCs) (cluster 5 (c5)), and oligodendrocytes (cluster 0 (c0) and cluster 3 (c3)). The findings of MEGENA modules reveal cell-type-specific functional modules characterized by highly coordinated molecular dysregulations in PD (**Supplementary Table S25-27**). The top 10 PD associated gene modules within each major cell type are shown in **Figure 3B**. Among the top 50 ranked modules, there are 17 neuronal modules, of which 14 originate from the neuron cluster c9. The number of top ranked modules from other glial cells are as follows: oligodendrocytes (c0: 5, c3: 13), microglia (c1: 4), astrocytes (c2: 7), and OPC (c5: 4).

We validate the biological coherence of our network modules in another snRNAseq dataset from the SN of PD and normal subjects (termed Ma dataset) at the cell type specific level(25). As shown in **Supplementary Table S28-29** based on a global approach across all identified modules, module preservation is highest in oligodendrocytes (~30-50%), followed by microglia/astrocytes (~20-30%), and neurons (~5-20%) (**Supplementary Figure S4A**). Additionally, we examine the conservation of the PD-related modules (i.e. modules enriched for up-regulated, down-regulated, and/or PD GWAS genes) in the two datasets. PD-related modules show higher and more significant preservation across all cell clusters (**Supplementary Table S30-31**). Similar to the global approach, module preservation is strongest in oligodendrocytes (35- 90%), followed by astrocytes/microglia (~60-90%), and neurons (~0-30%). Notably, in our dataset, the PD-related modules of neuron cluster C9 show greater preservation in the corresponding neuron cluster C4 from the Ma dataset than the PD-related modules identified in the other neuron cluster C6 of our dataset showed in C4 of the Ma dataset (**Supplementary Figure S4B**).

We additionally performed a network-based module preservation analysis using Weighted Gene Co-expression Network Analysis (WGCNA)(37). This approach evaluates preservation of the modules from our dataset in the Ma dataset. Overall, the module preservation results are consistent with those from the FET analysis. Moderate-to-high module preservation is observed across major cell types, with the highest preservation in oligodendrocytes, followed by microglia, astrocytes while

module preservation is more variable across neuronal clusters. PD-associated modules generally show greater preservation than total global modules (**Supplementary Table S32-33**). These results are broadly consistent with those from the FET analysis and provide complementary evidence for the cross-cohort reproducibility of PD-associated gene modules.

Neuronal gene modules are most dysregulated in PD

Among all the modules, the module M3 in the co-expression network from c9 is ranked as the top for its most significant enrichment of the down-regulated DEGs using Fisher's Exact Test (FET) (multiple testing corrected p or false discovery rate (FDR) = 1.7E-200, 2.3-fold enrichment (FE)) and GWAS genes (FDR = 8.1E-04, 2-FE) (**Figure 3B**, **Supplementary Table S25**). The global network of c9 shows a highly hierarchical structure, suggesting strong modular organization and multiscale biological processes (**Figure 3C**). Seven child modules of the module M3 in c9's co-expression network are highly enriched for down-regulated DEGs such as M27─►M70─►M100 (M27: FDR = 5.8E-18, 2.3 FE), M28─►M74 (M28: FDR = 1.1E-20, 2.4 FE), M29─►M79 (M29: FDR = 9.3E-24, 2.2 FE), M32─►M82─►M81 (M32: FDR = 2.2 E-16, 2.4 FE), M30 (FDR = 9.5 E-11, 2.8 FE), M31 (FDR = 5.8E-18, 2.5 FE), M33 (FDR = 1.2 E-11, 2.7 FE) (**Figure 3E, Supplementary Table S26**). M3 is associated with synapse organization (FDR p= 7.9E-24). All the other child modules of M3 are also enriched for down-regulated DEGs with synapse-related functions such as synapse assembly, regulation of neuron projection development, regulation of trans synaptic signaling, neuronal system, receptor localization to synapse and dendrite development (**Figure 3F, Supplementary Table S25, Supplementary Table S27**). These child modules of M3 are also among the top 50 modules from all the cell clusters and among the top 20 modules from c9 (**Figure 3D**, **Supplementary Table S25**). Interestingly, other branches of c9 separated from M3 module are not enriched for the downregulated DEGs. These modules are associated with pathways such as ion transport, metabolic process, and transcription, suggesting that only a small subset of c9 neurons appears to adopt the disease phenotype.

Apart from the neuron cluster c9, the modules in cluster c6 are less enriched for DEGs. In c6, a few top-ranked modules are associated with upregulated DEGs, such as M5 (FDR = 1.7E-08, 4.3 FE), M13 (FDR = 1.7E-08 , 10 FE), M126 (FDR = 1.7E-08, 45 FE), M8 (FDR = 3.73E-05, 4.6 FE), followed by modules are associated with downregulated DEGs such as M92 (FDR = 1.7E-05, 16.9 FE), M124 (FDR = 1.7, 36.1 FE) (**Supplementary Figure S5A, Supplementary Table S26**). These are also linked to pathways involved in synapse organization and axon development (**Figure S5B, Supplementary Table S25, Supplementary Table S27**). Interestingly, no module in the network from the neuron cluster c7 is enriched for DEGs (**Supplementary Table S25**), though many modules have meaningful biological functions (**Supplementary Figure S5C**). The global network of c7 also shows a more compact structure (**Supplementary Figure S5D**). This implies that the dysregulated genes in c7 are not well coordinated.

Glial gene modules associated with PD are involved in development process, metabolic processes and immune responses

In addition to neuronal modules, we also identify numerous glial modules in oligodendrocytes, microglia, astrocytes, and OPCs. Oligodendrocytes represent the largest population in the dataset, and consequently, the single-cell co-expression networks of cluster 0 (c0) and cluster 3 (c3) are among the largest, containing many genes and gene interactions. Among the top 50 ranked modules overall of c3, M10─►M41─►M164 emerged as one branch of particular interest. M10 is a parent module of M41 and M42, with its members enriched with both up (FDR = 2.9E-05, 4.3 FE), and down-regulated DEGs (FDR = 5.9E-34, 10.3 FE), and involved in cell polarity (FDR = 0.005), mitochondria organization (FDR p = 0.05), vesicle organization (FDR = 0.05) (**Figure 4A-B**, **Supplementary Figure S6A**). M10 has two branches, M41─►M164 and M42─►M166. One is the M41─►M164 branch in which both modules enriched with downregulated DEG (M41: FDR = 1.4E-43, 16 FE; M164: FDR=1E-44, 17 FE), and their members are associated with autophagy of mitochondria (M41: FDR = 0.04; M164: FDR = 0.04) (**Supplementary Figure S6B**). Another is M42─►M166 with both modules enriched with up-

regulated DEGs (M42: FDR = 3.7E-11, 12 FE; M166: FDR = 1.7E-06, 11 FE) and associated with apoptosis pathway (M42: FDR = 0.006; M166: FDR = 0.001) (**Supplementary Fig. S6C**). Some branches in the network of c3 are also associated with protein folding (M8─►M38─►M159), and oligodendrocyte differentiation (M5─►M20─►M111), highlighting the diverse pathways underlying oligodendrocyte functions. The network from the cell cluster c0 has a slightly smaller number of modules ranked at the top 50 modules from all the cell clusters and the most significant module M3 is enriched for both up-regulated (FDR = 2.2E-04, 2.1 FE), down-regulated DEGs (FDR = 1E-15, 2.3 FE), and is associated with actin filament organization (FDR = 1.5E-04). Several child modules of M3 from c0 are also enriched for DEGs, including M6─►M67 (involved in nervous system development), M8 (involved in metabolic process), and M17 (involved in nervous system development) (**Supplementary Figure S6E-F**).

We also identify several significant modules in the astrocyte cluster c2. Among those, the most significant module M3 is enriched for the down-regulated DEGs (FDR = 2.1E-38, 3.6 FE) and the GWAS genes (FDR = 4E-04, 1.9 FE) (**Figure 4C, Supplementary Figure S7A**). Some of M3's child modules are functionally important and enriched for down-regulated genes. The M3─►M59 branch is highly associated with regulation of small GTPase mediate signaling transduction (M3: FDR = 2.1E-10; M59: FDR = 0.004) (**Figure 4D**) while the M3─►M64 branch is associated with synapse assembly (M64: FDR = 5.1E-03) (**Figure 4E**). On the other hand, the modules M6 and M72 are highly enriched for upregulated DEGs (M6: FDR = 5.8E-14, 21.3 FE; M72: FDR =2.35E-08, 18 FE) (**Supplementary Figure S7B**), and linked to inclusion body assembly (M6: FDR = 1.7E-04), and regulation of endothelial cell chemotaxis (FDR = 2.5E-03) (**Supplementary Figure S7C**). These findings demonstrate the role of astrocytes in modulating and supporting neuronal and synaptic functions.

Furthermore, we identify several microglial modules highly associated with PD. The top microglia module M15, which is associated with mononuclear cell differentiation, is enriched for down-regulated genes (FDR = 4.8E-09, 6.6 FE) (**Figure 4F-G**, **Supplementary Figure S7C**). Additionally, we also

observe a few branches associated with GTPase activity. While the branch of M16─►M70─►M156 is associated with down-regulated DEGs (M16: FDR = 3.9E-08, 6.1 FE; M70: FDR = 1.7E-07, 6.3 FE; M156: FDR = 9.7E-08, 6.7 FE) (**Supplementary Figure S7D**), the branch of M12─►M55 is associated with up-regulated DEGs (M12: FDR = 3.6E-04, 8.7 FE; M55: FDR = 5.4-04, 13.8 FE) (**Supplementary Figure S7E**). The presence of co-expressed genes with the roles in innate immune signaling and GTPase activity might suggest the role of certain gene modules in translating or facilitating myonuclear cells to microglia into more reactive states.

Last but not least, although the modules in the network of OPC are less enriched for DEGs compared to other cell types, several modules are enriched for down-regulated DEGs (**Supplementary Figure S7F**) and are associated with glial cell development, nervous system development, and synaptic transmission (**Supplementary Figure S7G**). Interestingly, these down-regulated modules are the daughter modules of M2 (FDR = 0.01, 1.3 FE) with diverse functions, including the branch M6─►M57 (M6: FDR =2.9E-07, 6.4 FE; M57 – cFET = 5.3E-07, 7.3 FE) involved in regulation of trans-synaptic signaling (M6: FDR = 6.1E-03, M57: FDR= 1.4E-03), the branch M8─►M67 (M8: FDR = 5.3E-03, 5.3 FE; M67: FDR = 3.8E-04, 8.1 FE) involved in glial cell differentiation (M8: FDR = 1.8E-04; M67: FDR = 1.4E-06). These findings suggest that certain components of OPCs support both neuronal and glial functions, which have been understudied in the field.

Metacell-based Bayesian Networks predict well-known and novel key drivers

While the gene co-expression network analysis identifies coherent gene modules associated with PD in each major brain cell type, the findings remain inherently associative and don't establish causal relationships among genes. To move beyond correlation and uncover potential regulatory mechanisms, we construct Bayesian probabilistic causal networks (BNs) to identify gene-gene causal relationships. Given the sparsity existing in the datasets, we further remove additional genes and metacells, in a process of pruning to find the optimal cutoff (**Figure 1C**). To evaluate whether expression-based pruning preferentially excluded transcriptional regulators, we examined TFs before and after applying the Tg = 0.4 threshold.

Across all major cell type, around 38-77% of TF were removed, compared with 54-74% of genes overall were removed. Thus, TFs were not preferentially depleted by the filtering procedure (**Supplementary Table S34**).

After that, we construct a global BN for each cell cluster, followed by key driver analysis (KDA) to predict key regulators for each cell cluster(38, 39) (**Figure 5A-B**, **Figure S8A**, **Figure 6A-B**, **Supplementary Figure S9A**, **Supplementary Figure SC, Supplementary Figure S9E**). We identify a total of 151 key drivers (138 unique genes) and interestingly several drivers are detected in multiple cell clusters (**Table 4, Supplementary Table S35**). We also estimate the relevance of each gene to PD as the cooccurrence of a given gene and PD in PubMed. Among 138 unique key drivers, 43 (~30%) have occurrence above or equal to 5 (**Figure 5C**). Then, we classify the key drivers into two categories, 1) well-studied key drivers (cooccurrence ≥ 5), and 2) novel key drivers (cooccurrence <5) (**Table 4, Supplementary Table S36**).

Novel and known key drivers of neuronal clusters converge on synaptic regulation

The key drivers of neuronal clusters c7 and c9 are among the most significant drivers across all the cell cluster. The most significant driver is *CADPS2* in c9 (KDA FDR = 1.8E-39; 1.8 FE), which is the most downregulated in c9 (Fold Change (FC) = 0.48, FDR = 5.2E-35). In neurons, *CADPS2* encodes for a calcium binding protein that regulates synaptic exocytosis and dense core vesicles(40, 41), and has been previously studied in dopamine release(42). Also *CADPS2* is a novel small neuronal cell marker in idiopathic PD(43). *CADPS2* is also a member of the gene modules including M3, M32, and M81, which are associated with synapse organization, and neural system pathways (**Supplementary Table S23, Supplementary Table S23**). The BN in c9 also indicates *CADPS2* as an upstream driver of 4 down-regulated key drivers such as *CDH18*, *RYR2*, *UNC13C*, and *FSTL5*, three of which (*CDH18*, *RYR2*, *UNC13C*) play roles in calcium-depending synaptic cell-cell adhesion(44, 45), release of calcium ions(46), exocytosis(47), respectively (**Figure 5D, Supplementary Table S36**). Interestingly, the 2-layer subnetwork centered around *CAPDS2* (111 genes) is also involved in and modulation of chemical synaptic transmission

(FDR = 2E-04), regulation of trans-synaptic signaling (FDR=2.1E-03), synaptic vesicle docking (FDR=3.2E-03) (**Supplementary Table S37**). These genes form a coordinated subnetwork to modulate calcium-mediated process which is essential for neuronal communication and synaptic function.

RALYL, previously identified as a down-regulated key regulator from the network analysis of bulk transcriptomic data in PD(19), also emerges as a key driver in c9 (KDA: FDR = 2.1E-09; 2.2 FE; Expression: 0.6 FC, FDR = 9.8E-23) (**Supplementary Figure S8B, Supplementary Table S36)**. *RALYL* is a member of M3, M29, and M74 (**Supplementary Table S23**). The subnetwork around *RALYL* is associated with metabolic pathways such as cell surface receptor protein tyrosine kinase activity (FDR p = 3.7E-03), cellular response to histamine (FDR = 1E-02), and hypoxia (FDR = 1E-02) (**Supplementary Table S37**). Thirty of the 36 drivers in c9, which are downregulated in PD, are novel as only six genes have been associated with PD or studied in PD (**Table 4, Supplementary Table S35**).

In contrast to c9, twenty four of the 54 drivers in c7 have well-known functions associated with PD (24/54 genes). For example, a major driver *UCHL1* (Ubiquitin C-Terminal Hydroxylase) is implicated in the early onset of family and sporadic PD(5), influencing α-synuclein degradation and contributing to PD susceptibility(48). In cluster c7, *UCHL1* is significantly downregulated and enriched in cluster 7 (KDA: FDR = 8E-06, FE=2; Expression: FC=0.8, FDR = 5.4E-22). BN results indicate *UCHL1* is the downstream effector of two key drivers, *STMN2* (previously identified as a key driver in bulk transcriptomic data) and *OAT* (**Figure 5E, Supplementary Table S36**). The subnetwork around *UCHL1* is involved in regulation of high voltage-gated calcium channel activity (FDR = 6.3E-02), insulin secretion (FDR = 6.3E-02), and membrane invagination (FDR p = 6.3E-02) (**Supplementary Table S37**).

Besides *UCHL1*, we also identify other GWAS-associated gene *NSF* as a down-regulated key driver in c7 (KDA: FDR = 5.2E-34, FE =1.9; Expression: FC=07, FDR = 1.8E-56). *NSF* encodes N-ethylmaleimide-sensitive fusion protein, which plays an essential role in intracellular vesicle transport, synaptic transmission and membrane fusion(49), interacting with α-synuclein via SNARE complex protein(50), with diminishing expression level in a MPTP mouse model of PD(51) and lower blood and

brain level in PD(52). Additionally, *NSF* also directly regulates other key drivers such as *ATP1B1*, *SV2B*, and *STXBP1*, which play a role in regulating ATPase Na+/K+ pump(53), vesicle trafficking and exocytosis(54), and neurotransmitter release(55), respectively (**Supplementary Figure S8F, Supplementary Table S36**). The subnetwork centered around *NSF* is also involved in protein localization to plasma membrane (FDR = 9.4E-06), neuron projection development (FDR = 1.6E-04) and synaptic vesicle membrane organization (FDR=5.02E-03) (**Table S37**), highlighting the functional context of the PD GWAS gene.

STMN2, previously identified as regulator (19), is also a key driver in c7 (FDR = 8E-09, 1.9 FE) and downregulated (FC=0.8, FDR = 7.5E-15) (**Figure 5F, Supplementary Table S36**). *STMN2 i*s known to regulate microtube dynamic, axon formation and neurite outgrowth during development (56-58). The subnetwork around *STMN2* also indicates the enrichment of pathways such as regulation of microtube depolymerization (FDR = 3.4E-02), regulation of macroautophagy (FDR=3.9E-02), and polymeric cytoskeletal fiber (FDR p = 9.5E-02) (Supplementary **Table S37**). The subnetworks around *STMN2* are enriched for the knock-down signature of *STMN2* in mouse SN(19), validating the accuracy of our predicted metacell-based causal network (**Figure 5G**).

Additionally, we also identify *PREPL* as a key driver (KDA: FDR = 1.7E-18, FE=2.2; Expression: FC=0.8, FDR = 3.0E-23). *PREPL* encodes for propyl endopeptidase-like enzyme and has recently been implicated in neuronal functions, including protein trafficking, endocytosis, synaptic activity(59, 60), and the facilitation of α-synuclein aggregation seeding(61), highlighting its relevance as a candidate for further investigation. The subnetwork around *PREPL* (161 genes) suggests *PREPL* as downstream of *ATP1B1*, and upstream of many other key drivers such as *STXBP1*, *SLC9A6*, *RTN1*, *NPTN*, *TSPYL4*, *SV2A*, *SERINC1* (**Figure 5H, Supplementary Table S36**). This network is involved in positive regulation of synaptic transmission (FDR = 2.6E-03), negative formation of amyloid beta formation (FDR = 2.6E-03), and intracellular monoatomic cation homeostasis (FDR = 2.6E-03) (**Supplementary Table S37**).

In cluster c6, we identify the PD GWAS gene, *MDGA2,* as an up-regulated key driver (KDA: FDR = 2.1E-08, FE=3.3; Expression: FC=1.2, FDR = 2.1E-05). *MDGA2*, which encodes a regulator of axonal growth(62), contains a SNP that has been potentially linked to earlier onset of familial Parkinson's disease(63). The subnetwork around *MDGA2* (68 genes), which is involved in chemical synaptic transmission (FDR = 2.7E-02) and synapse organization (FDR p = 3.5E-02), shows that *MDGA2* directly regulates another key driver *NPAS3* (**Supplementary Figure S6E, Supplementary Table S36-37**).

Emerging key drivers in glial cells suggest novel causal roles in homeostasis, metabolic responses, immunity, and heat shock response

In oligodendrocytes, *MOBP* or myelin-associated oligodendrocyte basic protein, is identified as a key driver. *MOBP* plays a role in myelination and structural constituent of myelin sheath(64, 65). This gene is the top driver with the highest network significance and downregulated in c3 (KDA: FDR p = 9.2E-42, 5.5 FE; 0.8 FC, FDR = 3E-59) and c0 (KDA: FDR = 2.7E-15, 6.05 FE; Expression: 0.9 FC, FDR p = 6.3E-100). The subnetworks around *MOBP* in c3 and c0 indicate *MOBP* as an upstream of key drivers such as *NEAT1*, *COL4A5*, *CARNS1*, *LINC01505* in c3 (**Figure 6B**), and *CARNS1* in c0 (**Figure S9B**). In c3, the subnetwork around *MOBP* (307 genes) are associated with regulation of dendritic spine development (p = 2.7E-04), myelination (p = 9.9E-04), neuronal cell-cell adhesion (p = 1.1E-03), and protein localization to synapse (p = 2.1E-03) while in c0, the subnetwork (61 genes) is associated with transition metal ion transport (p = 1.1E-04), substantia nigra development (p = 3.3E-04), and regulation of neuron apoptotic process (p=6.5E-04) in c0 (Supplementary **Table S36**). Although *MOBP*'s role is not novel, the specific pathway co-enrichment identified in our study highlights its dual function in both structural maintenance and cellular response. In c3, the subnetwork (307 genes in c3, 61 genes in c0) are associated with regulation of dendritic spine development (p = 2.7E-04), myelination (p = 9.9E-04), neuronal cell-cell adhesion (p = 1.1E-03), and protein localization to synapse (p = 2.1E-03) in c3 and associated with transition metal ion transport (p = 1.1E-04), substantia nigra development (p = 3.3E-04), and regulation of neuron apoptotic process (p=6.5E-04) in c0 (**Supplementary Table S37**).

We also identify a few key drivers in astrocytes including *WDR49*, and heat shock protein family genes such as *HSPA1B*, *HSPB1*, *HSP90AA1, HSPH1*. *WDR49* is downregulated in PD and highly ranked as a key driver in cluster 2 astrocytes (KDA: FDR = 7.6E-12, 5.53 FE; Expression: 0.9 FC, FDR p = 3.4E-05). Interestingly, *WDR49* encodes a protein with unknown function, however, recent study suggests *WDR49* as the disease-associated subpopulation in Frontotemporal dementia carrying GRN mutation and Alzheimer's Disease(66). The subnetwork around *WDR49* (100 genes) is involved in phosphatidic acid process (FDR p = 9.0E-03) (**Figure S8D, Supplementary Table S36-36**). Interestingly, a recent study shows that α-Synuclein (α-Syn), a protein causally linked to PD, binds to vesicles containing phosphatidic acid (PA), a plasma membrane phospholipid(67). The identification of *WDR49* as a key driver opens a new avenue for research into its potential role in PD pathogenesis.

We also identify key drivers in microglial clusters. One driver is *APBB1IP* which is prominently associated with activation and modulation of innate immune response. *APBB1IP* is a regulator of leukocyte recruitment and pathogen clearance through complement-mediated phagocytosis(68, 69). In our analysis, *APBB1IP* is moderately downregulated (KDA: FDR = 8.5E-10, FE=5.45; Expression: FC=0.85, FDR = 1.1E-09), and the subnetwork around *APBB1IP* is involved in synapse pruning (FDR p = 6.7E-04), microglial cell migration (FDR p = 3.2E-02), and macrophage migration (FDR p = 4.7E-02) (**Figure 6E**, **Supplementary Table S36-37**). The observed association between *APBB1IP* and pathways related to microglial activation underscores its importance in disease-related glial responses. Interestingly, the direct effectors of *APBB1IP* such as *C3* and *CX3CR1* are also downregulated and involved in immune responses and inflammation (**Figure 6E**). Moreover, *ST6GAL1*, an upstream gene of *APBB1IP*, encodes a member of glycosyltransferase family, ST6Gal-I sialyltransferase, is known to regulate several immunological process such as immunoglobulin production in B cells(70) or inflammatory pathways(71). Therefore, our findings further underscore the importance of *APBB1IP* and its surrounding effectors in driving immune responses in microglia.

Last but not least, *HSP90AA1* is identified as key driver and is consistently upregulated across multiple glial cells including OPC (KDA: FDR = 3.6E-11, 10.4 FE; Expression: 1.3 FC, FDR =9.9E-13), oligodendrocytes in c3 (KDA: FDR = 1.2E-07, 3.69 FE; Expression: 1.1 FC, FDR =2.7E-08), oligodendrocytes in c0 (KDA: FDR = 1.7E-05, 3.52 FE; Expression: 1.2 FC, FDR = 0.005), microglia (KDA: FDR = 3.2E-06, 4.02 FE; Expression: 1.2 FC, FDR p = 9.7E-11), and astrocytes (KDA: FDR = 0.02, 4.3 FE; Expression: 1.1 FC, FDR = 1.2E-068) (**Figure 6E, Supplementary Figure S9F-I**). In the OPC cluster c5, the subnetwork around *HSP90AA1* is involved in responses to unfolded protein (FDR = 1.9E-10), protein ubiquitination (FDR = 7.8E-06), and apoptosis (FDR = 6.4E-05) (**Figure 6E, Supplementary Table S36**). The subnetworks around *HSP90AA1* in other glial cells are enriched for similar pathways (**Supplementary Table S37-38**). The consistent regulatory pattern of *HSP90AA1* in glial cells suggests its potential involvement in maintaining glial homeostasis and contribution to the underlying neurodegenerative mechanisms.

DISCUSSION

In this study, we develop a metacell-based multiscale network analysis (MCMNA) framework for analyzing snRNA-seq data and apply it to a PD snRNA-seq data to identify cell type specific gene modules and key drivers for PD. Use of metacells rather than single cells addresses the challenge in dealing with the intrinsic sparsity in snRNA-seq data. The application of MCMNA to PD systematically identifies gene modules associated with PD and putative key network drivers for PD in the major brain cell types. Notably, 30% of the predicted drivers in neurons have been studied in PD, including *UCHL1*, *NSF*, *SNAP25*, *STMN2*, and *PREPL* while the rest 70% are novel or understudied, suggesting that our method can capture previously unrecognized regulators with potential significance in the disease.

Disease associated networks often exhibit sparse connectivity, reflecting the complexity and heterogeneity of underlying biological processes. Especially in snRNA-seq, some signals can be hidden by sparsity in gene expression profiles. One key advantage of the metacell approach in snRNA-seq is the ability to reduce data sparsity, thereby significantly enhancing downstream biological signals(27-29). Aggregation of similar cells into metacells allows us to improve the detection of conserved markers specific to each cell type and reduce the false positive differentially expressed genes. Metacell-specific DEGs captured biologically relevant signals consistent with some previous PD studies. In glial populations, the enrichment of metal ion homeostasis pathways was driven by metallothionein genes whose dysregulation has also been reported in PD(72). Additionally, we observed several canonical dopaminergic neurons (*SLC6A3*, *TH*, *SLC18A2*) were downregulated in microglia and astrocytes in PD. Although unexpected, dopaminergic-associated transcriptional signals in glial have reported previously dataset(22), suggesting that such signals are not unique to our dataset Although dopaminergic-associated transcripts detected in glial populations may reflect biologically relevant expression, we cannot fully exclude a contribution from ambient RNA. Future studies incorporating ambient RNA correction algorithm such as SoupX (73) or CellBender (74), will help further distinguish cell-intrinsic transcriptional signals from potential background contamination. Similarly, heat-shock and proteostasis-related genes were recurrently identified in independent datasets(21,

25), consistent with the involvement of cellular stress responses in PD. Together, these findings support the ability of metacell analysis to detect subtle but biologically coherent disease-associated changes.

Importantly, it strengthens gene-gene correlation analysis, which serves as a foundation for constructing more robust networks. We examine different metacell construction method and parameters choices (e.g. graining level – the ratio between the number of cells and the number of metacells, and expression representation – average vs. summing up, unweighted vs weighted) on our dataset to accelerate and enhance substantial downstream analyses. Additionally, the denser and more accurate correlation structure stemmed from biologically meaningful matrix (BMC) results in more meaningful gene co-expression networks. Additionally, the process of pruning enables more accurate construction of causal Bayesian Networks.

Our metacell-based MEGENA results show that different modules capture distinct biological pathways and reveal cell-type-specific regulatory patterns. For example, the top-ranked modules in neurons are involved in synaptic transmission and nervous system development while the top-ranked modules in glial cells are enriched with diverse functions such as development process, metabolic signaling, and immune responses. We identify three distinct neuronal clusters, each displaying unique network architecture that reflects the cellular heterogeneity in PD. The typical neuronal cluster c7 with Tyrosine hydroxylase (TH) marker exhibits a more compact and highly connected network structure, while the neuronal cluster c9 with the novel *RIT2* marker has a sparser structure. Cluster c7 has no hierarchical modules that are enriched with PD signatures, indicating that many dysregulated genes in this neuron are rather highly coordinated. Interestingly, in c9 neurons prone to be most degenerated in PD, only a small subset of cells demonstrates the most pronounced network changes (c9_M3), highlighting the selective vulnerability within this cluster. Previous studies using midbrain datasets and mouse models have implicated SOX6+/AGTR1+ dopaminergic neuronal populations as particularly vulnerable in PD(75-77). In our human substantia nigra dataset, neither SOX6 nor AGTR1 was identified as a defining feature of c9 or within its dysregulated M3 module (Supplementary **Table S23**). Given that the substantia nigra is the

primary site of dopaminergic neuronal degeneration in PD, our analysis provides a complementary, anatomically and disease-relevant context for investigating selective neuronal vulnerability. The RIT2-enriched c9 signature therefore represents an additional axis of vulnerability in the human substantia nigra that is not fully captured by the previously described *SOX6*+/*AGTR1*+ signature. However, differences in species, anatomical sampling, and analytical resolution may also contribute to this apparent distinction, and further investigation is required to determine their relationship.

In addition to neurons, our metacell-based MEGENA allows us to uncover key features within glial cells, highlighting their distinct and potentially underappreciated roles in disease pathologies. Beyond neurons, oligodendrocytes cluster c0 and c3 have gene modules highly associated with PD, revealing broader functional roles than previously described. Several modules in c0 and c3 are enriched for mitochondrial autophagy, apoptosis, oligodendrocyte differentiation, and protein folding, revealing a more dynamic and multifaceted contribution to PD. Additionally, we observe an increased abundance of astrocytes (**Figure S1D**), yet their functional role in PD remains poorly understood. Our MEGENA pinpoints additional astrocytes involvement, highlighting modules enriched for signaling transduction, synapse assembly, suggesting possible contribution to maintaining or altering synaptic environment during disease progression. Microglia, in contrast, has fewer high-ranked modules, which are strongly associated with the immune response pathway. This agrees with well-established knowledge of their roles in neuroinflammation. Additionally, our study also indicates that several OPC modules associated with neural functions and glial differentiation are dysregulated, further highlighting the critical roles of OPC in PD.

Our module preservation analyses show that module preservation varies substantially across cell types, with highest score in oligodendrocytes, followed by astrocyte, microglia and neurons (**Supplementary Figure S4A-B**). This indicates that gene co-expression networks are more stable and conserved in glial populations, while neuronal networks are comparatively more dynamic, and reflect greater heterogeneity. PD-related modules also have higher preservation overall, suggesting that disease-associated transcriptional programs are more robust and reproducible.

Our analysis identifies both known and novel gene targets that are strongly related to PD-related pathways, allowing us to propose a dynamic mechanistic model of neurodegeneration and susceptibility. Several key drivers in neuronal clusters such as *CADPS2*, *NSF*, *PREPL* are associated with calcium signaling, synaptic transmission, protein trafficking and have been studied in PD in some extent(43, 51, 52, 61, 78, 79), highlighting the central role in neuronal functions. We also observe distinct neuronal subpopulations with unique key driver profiles and transcriptomic alterations, suggesting heterogeneity in the degree of regulatory pattern in degeneration and vulnerability. This observation aligns with previous studies reporting selective neuronal vulnerability in neurodegenerative diseases including PD(31, 80). Our findings suggest that multiple pathogenic processes may operate in parallel or sequentially. For example, in novel cluster 9 that shows degeneration in PD*, CADPS2* enriched for calcium signaling can point to novel mechanism of calcium dysregulation, contributing to stress or glial-neuronal signaling imbalance. In contrast, cluster c7 is dominated by key drivers in synaptic transmission, exocytosis and protein trafficking such as *NSF* or *PREPL*.

Oligodendrocytes in the central nervous system (CNS) are essential for forming myelin sheaths—multi-layered lipid membranes that insulate axons(81, 82). Among the top key drivers we identify in oligodendrocytes, *MOBP* stands out as the highest-ranked gene across both oligodendrocyte clusters c0 and c3. It is the third most abundant protein in CNS myelin and is known to contribute to myelin integrity. Moreover, recent study suggests a possible association between *MOBP* and Parkinson's disease(83). Our findings highlight *MOBP* as a potential therapeutic target, given its role in maintaining structural stability and mediating cellular responses. Additionally, key drivers identified in microglia cells point toward compelling hypotheses involving innate immune activation. Consistent with the important role of *APBB1IP* suggested by our network, *APBB1IP* has been identified as modulator of immune gene co-expression in specific microglial subtype in a recent aged mouse hippocampus study(84). It is also recognized as a core component of microglial expression signatures(85, 86). Furthermore, human GWAS has linked *APBB1IP*

to both AD and schizophrenia(87, 88). Our findings contribute to this growing body of evidence by highlighting the relevance of *APBB1IP* in PD.

Heat shock proteins (HSPs) are molecular chaperons that manage protein folding and prevent misfolding by regulating protein aggregation and disaggregation in both intracellular and extracellular environments, playing a critical role in maintaining proteostasis(89). In our study, we observe consistent upregulation of HSPs across multiple glial cell types (**Figure 2B**). Pathways analysis further highlights the activation of processes such as protein folding and Hsf1 activation (**Figure 2C**). In particular, in PD, HSP60, HSP70, and HSP90 interact with α-synuclein, becoming incorporated into α-synuclein aggregates as they attempt to prevent or repair protein misfolding and accumulation(90-93). Among these, we nominate *HSP90AA1* as a key candidate as it is significantly upregulated in 5 glial cell types and ranks highly in our network analysis. Notably, *HSP90AA1* has been identified as a marker of microglial activation in recent PD omics-studies(43, 94). Altogether, these findings support emerging evidence that non-neuronal cells significantly contribute to disease progression through immune signaling, stress response, or other metabolic and cellular response.

Our result also reveals strong concordance between bulk and snRNA-seq analyses, providing mutual validation of key molecular drivers implicated in PD. Several key drivers previously identified in bulk transcriptomic studies such as *STMN2* and *RALYL* are also drivers in our snRNA-seq network. This overlap underscores the robustness of our network-based approach and demonstrates that strong, biologically relevant signals identified in bulk are not only preserved but also refined at the single cell level. The replication of these findings confirms the reliability of bulk-based insights while offering higher resolution interpretation of cell-type-specific dynamics. By disentangling the contributions of distinct cell populations, we uncover cell-specific regulatory responses, enabling increased specificity in identifying therapeutic targets.

We apply our pipeline to a previously published snRNA-seq dataset to identify key drivers(25). In this dataset, we identify 121 candidate key driver genes (**Supplementary Table S38**); however, there is

minimal overlap with our results. Only a small number of genes are shared between the two analyses, including *HSP90AA1* in glial cells, and *CADPS2*, *CPLX1*, *SERINC1* in neurons (**Supplementary Table S39**). The limited concordance likely reflects the smaller sample size of the external dataset, which reduces the robustness of network construction, and consequently, the reliability of key driver identification.

We also highlight the functional contexts of PD GWAS genes using cell type specific Bayesian causal networks. Our causal networks in 5 major cell types across 8 cell clusters harbor a total of 509 PD GWAS genes, but only two of them (*NSF* in c7 and *MDGA2* in c6) are identified as key drivers (Supplementary **Table S39**. Additionally, we predict *UCHL1*, a known PD causal gene, as a putative driver. Moreover, our network reveals a substantial number of novel key driver genes. These understudied genes may represent critical, yet unrecognized components involved in disease progression, particularly within specific cell types, enabling a broader and more comprehensive understanding of PD mechanisms.

We also compare our DEGs signatures with previously published bulk proteomics datasets of human SN(95). We retrieve the identified differentially expressed proteins (DEPs) from 2 published cohorts (**Supplementary Table 41-43**). and assess the overlap with cell-type-specific DEGs using FET. The results show that the shared DEPs are predominantly enriched in neuronal clusters, with consistent directionality (**Supplementary Figure S10A-B, Supplementary Table S44-45**). While bulk proteomics capture an overall signal of the molecular dysregulation in PD, these findings indicate that the strongest and most robust changes are driven by neuronal population. This underscores the importance of cell-type resolved analyses for accurately interpreting disease-associated signatures.

In summary, our study identifies a set of molecular subnetworks and putative key driver genes that illuminate the molecular architecture of PD at the single cell resolution. By applying MCMNA to address sparsity in snRNA-seq, we uncover well known gene and novel regulatory candidates that may have important roles in PD. These findings offer a better understanding of the cellular heterogeneity and regulatory dynamics underlying neuronal and glial cell populations.

Limitations of the study

Even though the cohort is the largest dataset of SN to date, the sample size is still relatively small. Furthermore, the current study lacks experimental validation to confirm computational validation. Even though we observe that previously perturbation signatures overlapped with our predicted *STMN2* network in c7 neurons, more validation is needed for the novel targets. Future work will focus on expanding the analysis to larger and more diverse datasets, and implement different confounding factors such as sex, Braak stages in the study to get more insights into the dynamic regulatory machinery. Functional perturbation of prioritized regulators in relevant *in vitro* and *in vivo* models to determine whether these predicted drivers directly contribute to the disease pathogenesis and represent tractable therapeutic targets. Our approach can also be improved with comprehensive longitudinal studies, extended studies beyond sporadic cases and specific brain regions, and incorporation of other omics-based networks such as proteomics and epigenomics. More importantly, this integrative approach establishes a foundation to guide future efforts toward generating increasingly high-quality and informative co-expression and causal inference, paving the way for deeper mechanistic insights and the discovery of novel therapeutic targets.

Data Availability

This study did not generate new unique reagents. The raw data of the our snRNA-seq(20) are available from the Gene Expression Omnibus (GEO) under the accession code GSE184950. The raw data from Lee et al.(21) are available from GEO under the accession code GSE148434. The raw data from Martirosyan et al. dataset(22) are available from GEO under the accession code GSE243639. The raw data from Ma et al.(25) are available from GEO under the accession code GSE253462.

The scripts and data used in this study have been deposited on GitHub (105) and Zenodo (106). Any additional information required to reanalyze the data reported in this study is available upon request from the lead contact.

ACKNOWLEDGEMENTS

We thank the members from the B.Z. laboratory and Z.Y. laboratory for the comments and feedback during the development of the manuscript. The schematics were created using BioRender. This work was supported in part by grants from Parkinson's Foundation Center Grant PF-RC-936279 (to B.Z & Z.Y.), National Institutes of Health R01AG074010 (to B.Z.) and R01AG085182 (to B.Z.). This work was also supported in part through the computational and data resources and staff expertise provided by Scientific Computing and Data at the Icahn School of Medicine at Mount Sinai and supported by the Clinical and Translational Science Awards (CTSA) grant UL1TR004419 from the National Center for Advancing Translational Sciences. Research reported in this publication was also supported by the Office of Research Infrastructure of the NIH under award number S10OD026880 and S10OD030463. The content is solely the responsibility of the authors and does not necessarily represent the official views of the NIH. These funding sources had no role in the design and conduct of the study or collection, management, and analysis of the data.

AUTHORS' CONTRIBUTION

Conceptualization, B.Z.; Investigation, L.C., G.Y., X.Z., M.W., Z.Y., and B.Z.; Resources, B.Z.; Writing – Original Draft, L.C. and B.Z.; Writing – Review & Editing, All authors; Supervision, B.Z., Funding Acquisition, B.Z., and Z.Y.

DECLARATION OF INTERESTS

The authors declare that they have no competing interests

Table information

Table 1. Distribution of Metacell Sizes Across Cell Clusters in the MSSM dataset

Table 2. Number of Differentially Expressed Genes (DEGs) Identified by the Metacell (MC) Method per Cell Cluster

Table 3. Number of MEGENA modules, modules associated with PD DEGs/ pathway annotation

Table 4. Summary of Key Driver Genes

Supplementary Information

Table S1. Seurat data sizes for the SC and MC method across MSSM, KAIST, VIB and Columbia datasets.

Table S2. Comparison of major pathways between the SC and MC methods in the MSSM dataset.

Table S3. Differentially Expressed Genes (DEGs) Identified by the Metacell (MC) Method per Cell Cluster.

Table S4. Conserved Marker Genes in the MSSM Dataset Identified by Metacell.

Table S5. Distribution of Metacell Sizes Across Cell Clusters in the VIB dataset.

Table S6. Differentially Expressed Genes (DEGs) Identified by the Metacell (MC) Method per Cell Cluster in the VIB dataset.

Table S7. Distribution of Metacell Sizes Across Cell Clusters in the KAIST dataset.

Table S8. Differentially Expressed Genes (DEGs) Identified by the Metacell (MC) Method per Cell Cluster in the KAIST Dataset.

Table S9. Distribution of Metacell Sizes Across Cell Clusters in the Columbia dataset.

Table S10. Differentially Expressed Genes (DEGs) Identified by the Metacell (MC) Method per Cell Cluster in the Columbia Dataset.

Table S11. Fisher's Exact Test (FET) Pathway Overlap Analysis Between the VIB and MSSM Datasets.

Table S12. Fisher's Exact Test (FET) Pathway Overlap Analysis Between the KAIST and MSSM Datasets.

Table S13. Fisher's Exact Test (FET) DEGs Overlap Analysis Between the Columbia and MSSM Datasets.

Table S14. Shared Enriched Pathways across All Cell Types Between the Metacell (MC) and Single-Cell (SC) Methods.

Table S15. Summary Statistics of Shared and Unique Upregulated Pathways Identified by the SC and MC Methods.

Table S16. Summary Statistics of Shared and Unique Downregulated Pathways Identified by the SC and

MC Methods.

Table S17. Unique Enriched Pathways per Cell Cluster Identified by the Metacell (MC) Method.

Table S18 . Unique Enriched Pathways per Cell Cluster Identified by the Single-Cell (SC) Method.

Table S19. Unique DEGs per Cell Cluster Identified by Metacell (MC) Method.

Table S20. Enriched Pathways per Cell Cluster Driven by unique DEGs per Cell Cluster Identified by Metacell (MC) Method using EnrichR database.

Table S21. Intersection Analysis of Unique DEGs Identified by the Metacell (MC) Method and DEGs in Columbia and KAIST Dataset.

Table S22. Fisher's Exact Test (FET) Results for STMN2 Pathway Preservation.

Table S23. Gene Membership in Significant Co-Expression Modules per Cell Cluster.

Table S24. GWAS Genes used in the study.

Table S25. Module Ranking and Functional Pathway Annotation per Cell Cluster.

Table S26. Comprehensive Fisher's Exact Test (FET) Results across All MEGENA Modules.

Table S27 . Complete Pathway Enrichment Information for All MEGENA Modules.

Table S28. Summary of Module Preservation of All Modules at Cell Type Specific Levels.

Table S29. FET Results of Module Preservation of All Modules at Cell Type Specific Levels.

Table S30. Module Preservation of PD-related Modules at Cell Type Specific Levels.

Table S31. FET Results of Module Preservation of PD-related Modules at Cell Type Specific Levels.

Table S32. Module preservation analysis of All the Modules by WGCNA at Cell Type Specific Levels.

Table S33. Summary of Module Preservation Analysis by WGCNA at Cell Type Specific Levels.

Table S34. Number of TF and genes removed during pruning process.

Table S35. Detailed Key Driver Genes Identified in Major Cell Types.

Table S36. Two-Layer Bayesian Network of selected key driver.

Table S37. Pathway Enrichment Results for the Two-Layer Network of a Key Driver Gene using the EnrichR database.

Table S38. Detailed Key Drivers Gene identified in Major Cell Types in the Columbia dataset.

Table S39. Shared Key drivers in two datasets.

Table S40. GWAS Genes Mapped to Cell Type-Specific Gene Co-Expression Networks.

Table S413. Number of gene pairs before and after BMC filtering.

Table S42. Differentially Expressed Proteins (DEPs) identified in the published cohort of 15 PD and 15 Control samples.

Table S43. Differentially Expressed Proteins (DEPs) identified in the published cohort of 9 PD and 10 Control samples.

Table S44. Fisher's Exact Test (FET) Results of shared DEGs and DEPs from the cohort of 15 PD and 15 Control samples.

Table S45. Fisher's Exact Test (FET) Results of shared DEGs and DEPs from the cohort of 9 PD and 10 Control samples.

Supplementary figure legends

Figure S1. Generation and characterization of metacells.

(A-B) Number of up-regulated (A) and down-regulated (B) differentially expressed genes (DEGs) in single cells and metacells across different conditions in 12 cell clusters.

(C) Number of conserved markers expressed genes (DEGs) in single and metacells across different conditions in 12 cell clusters.

(D) Nuclei fraction distribution between PD and control across 12 clusters. P-value was calculated using Wilcoxon rank sum test. * p-value < 0.05, ** p-value < 0.01.

(E) Volcano plot showing DEGs in selected major cell clusters c0 Oligodendrocyte, c5 Oligodendrocyte progenitor cell, and c6 Neuron. Top 5 upregulated and 5 downregulated DEGs (by -log10(FDR)) of each cell cluster are labeled in red or blue, respectively.

(F, I, L) UMAP plot showing cell clusters in VIB dataset(22) (F) or KAIST dataset(21)(I) or Columbia dataset(25) (L)

(G, J, M) Expression pattern of known brain cell- type marker genes in VIB dataset(22) (G) or KAIST dataset(21) (J) or Columbia dataset(25)(M)

(H, K, N) Comparation of Fishe Exact Test (FET) enrichment of DEGs of MSSM dataset and VIB dataset(22) (H) or MSSM dataset and KAIST dataset(21) (K) or MSSM dataset and Columbia dataset(25) (N) at cell cluster information. The size of dot is proportional to the Fold Enrichment and color intensity indicates the value of -log10(FET FDR). Ast, astrocytes; Neu, neurons; Mic, microglia; Oli, oligodendrocytes; OPC, oligodendrocyte progenitor cells; End, endothelia; Fib, fibroblast-like cells; Per, pericytes, T, T cells.

Figure S2. Comparison of the enrichments of all the pathways in the single cell (SC) and Metacell (MC) DEG signatures.

The -log10(FDR) values of all pathways from (A) upregulated or (B) downregulated differentially expressed genes (DEGs) were plotted. Black dot indicates the unique pathways from each method. Spearman correlation was calculated from all pathways in each cluster.

Figure S3. Generation of Biologically Meaningful Correlation (BMC) Matrix.

(A) Workflow of the method.

(B) Correlation distribution of SC and MC across 9 cell clusters. SC and MC at the mincell cutoff = 20%.

(C) Plot of Mean Square Error values against different min cell cut off across 12 cell cluster.

(D) Plot of gene expression versus gene network connectivity (the number of nodes that directly connect with a given node in a gene network).

Figure S4. Summary of the module preservation analysis of the MSSM and Columbia datasets. (A) Module preservation summary for all the co-expressed gene modules. (B) Module preservation summary for the PD-associated co-expressed gene modules within the same cell type.

Figure S5. Metacell-based gene coepxression of neuronal cell clusters.

(A) Enrichment of the DEGs of PD in MEGENA modules in cluster 6 neurons. Color intensity is proportional to -log10(corrected FET p-value).

(B-C) Enrichment of the major pathways in MEGENA modules in cluster 6 neurons (B) and cluster 7 neurons (C).

(D) The global MEGENA based gene coexpression network in Cluster 7 (neurons). The pie chart of each node indicates whether it is a DEG or GWAS gene. The size of a node is proportional to the node connectivity in the MEGENA network. (UP: Upregulated; DN: Downregulated).

Figure S6. Metacell-based gene coepxression of the oligodendrocyte clusters.

(A, D) Enrichment of the major pathways in MEGENA modules in cluster 3 (A) and cluster 0 (D).

(B, C, F) The top-ranked module M41 of cluster 3 (B), M42 of cluster 3 (C), M3 of cluster 0 (F). The pie chart of each node indicates whether it is a DEG or GWAS gene. The size of a node is proportional to the node connectivity in the MEGENA network. (UP: Upregulated; DN: Downregulated).

(E) Enrichment of the DEGs of PD in MEGENA modules in cluster 0. Color intensity is proportional to -log10(corrected FET p-value).

Figure S7. Metacell-based gene coepxression of other glial cell clusters.

(A, C, G) Enrichment of the major pathways in MEGENA modules in cluster 2 (astrocytes) (A) cluster 1 (microglia) (C), and oligodendrocyte progenitor cells (OPC) (G).

(B, D, E, H, I) The top-ranked module M6 of cluster 2 (astrocytes) (B), M16 of cluster 1 (microglia) (D), M12 of cluster 1 (microglia) (E), M6 of cluster 5 (OPC) (H), and M8 of cluster 5 (OPC) (I). The pie chart of each node indicates whether it is a DEG or GWAS gene. The size of a node is proportional to the node connectivity in the MEGENA network. (UP: Upregulated; DN: Downregulated).

(F) Enrichment of the DEGs of PD in MEGENA modules in cluster 5 (OPC). Color intensity is proportional to -log10(corrected FET p-value).

Figure S8. Bayesian Probabilistic Causal Network (BN) of neuronal cells.

(A) BN in cluster 6 (neurons).

(B-D) The 2-layer network neighborhoods of *RALYL* in cluster 9 neurons (B), *NSF* in cluster 7 neurons (C), *MDGA2* in cluster 6 neurons (D), respectively. The size of a node is proportional to the node connectivity in the BN. The directionality of the arrow indicates the upstream-downstream effect. Nodes with burgundy labels are key drivers in that network.

Figure S9. Bayesian Probabilistic Causal Network (BN) of glial cells.

(A, C, E) BN in cluster 0 (oligodendrocytes), C2 (astrocytes), and C5 (Oligodendrocyte progenitor cells (OPC)).

(B, D, F-I) The 2-layer network neighborhoods of *MOBP* in cluster 0 (oligodendrocyte) (B), *WDR49* in cluster 2 (astrocytes) (D), *HSP90AA1* in cluster 0 (oligodendrocyte) (F), cluster 1 (microglia) (G), cluster 2 (astrocytes) (H), and cluster 3 (oligodendrocyte) (I), respectively. The size of a node is proportional to the

node connectivity in the BN. The directionality of the arrow indicates the upstream-downstream effect. Nodes with burgundy labels are key drivers in that network.

Figure S10. Comparation of FET enrichment of DEGs of MSSM dataset and DEPs of the bulk proteomics study (A) using cohort of 15 PD and 15 Control or (B) using the cohort of 10 PD and 9 Control. The size of dot is proportional to the Fold Enrichment and color intensity indicates the value of -log10(FET FDR). Ast, astrocytes; Neu, neurons; Mic, microglia; Oli, oligodendrocytes; OPC, oligodendrocyte progenitor cells; End, endothelia; Fib, fibroblast-like cells; Per, pericytes; T, T cells.

Figure legends

Figure 1. Integrative network biology framework for decoding molecular mechanisms of Parkinson's Disease at single cell level.

(A) Construction and characterization of metacells. Metacells were generated using SuperCell by constructing a k-NN network. Cells were aggregated within each cell cluster and condition, followed by differential expression and pathway enrichment analyses.

(B) Metacell-based Multiscale Embedding Gene Co-expression Network Analysis. Gene correlations were used to generate a biologically meaningful correlation (BMC) matrix, followed by MEGENA to identify multiscale co-expression modules and functional enrichment analysis.

(C) Metacell-based Bayesian Network and Key Driver analysis. Bayesian networks were constructed using RIMBANet with TF–target relationships from TFLink as regulatory priors. Key driver analysis was then performed to prioritize candidate regulators in the reconstructed causal networks.

μ, mean gene expression within a metacell; G, gene; N, network node; TF, transcription factor; k-NN, k-nearest neighbors; BMC, biologically meaningful correlation; MEGENA, Multiscale Embedded Gene Co-expression Network Analysis; BN, Bayesian network; KDA, key driver analysis

Figure 2. Generation and characterization of metacells.

(A) Number of differentially expressed genes (DEGs) in single and metacells across different conditions in 12 cell clusters.

(B) Volcano plot showing DEGs in selected major cell clusters c1 Microglia, c2 Astrocyte, c3 Oligodendrocyte, c7 Neuron and c9 Neuron. Top 5 upregulated and 5 downregulated DEGs (by -log10(FDR)) of each cell cluster are labeled in red or blue, respectively.

(C) Heatmap of top pathways enriched for up- and down-regulated genes in major cell type cluster.

(D-E) Barplot of gain of enriched pathways from up-regulated genes. (D) or downregulated DEGs (E) in major cell clusters. Color indicates the -log10(FDR) value of the corresponding pathway from single cell (SC) and metacell (MC) method. The number on the bar indicates the difference of -log10(FDR) value between MC and SC. Ast, astrocytes; Neu: neurons; Mic, microglia; Oli, oligodendrocytes; OPC, oligodendrocyte progenitor cells.

Figure 3. Metacells-based gene co-expression network of Parkinson's Disease.

(A) Comparison of FET enrichment of STMN2-related genes and STMN2 perturbation DEGs. Top value -log10(FDR), bottom value FE (SC: Single cell, MC: Metacell, FDR: False Discovery Rate, C6: Cluster 6, C7: Cluster 7, C9: Cluster 9, all: all cell clusters).

(B). Top 10 modules enriched for the PD-related signatures within each major cell cluster (UP: Upregulated; DN: Downregulated).

(C). A global MEGENA network in cluster 9 neurons. The module membership is represented by different colors. Seven Top ranked modules (M27, M28, M29, M30, M31, M32, M33) from parental module M3 and other modules enriched with other pathways (M5, M6, M12, M24) are highlighted.

(D) The top 30 MEGENA modules most enriched for the PD-related gene signatures include the PD DEGs with each cell cluster, and PD GWAS genes.

(E) Enrichment of the DEGs of PD in MEGENA modules in cluster 9 neurons. Color intensity is proportional to -log10(corrected FET p-value). (F) Enrichment of the major pathways in MEGENA modules in cluster 9 neurons.

Figure 4. Metacell-based gene coepxression of several glial cell clusters.

(A,C,F) Enrichment of the DEGs of PD in MEGENA modules in cluster 3 oligodendrocytes (A), cluster 2 astrocytes (C), or cluster 1 microglia (F). Color intensity is proportional to -log10(corrected FET p-value).

(B, D, E, G). The top-ranked module M10 of cluster 3 oligodendrocyte (B), M59 of cluster 2 astrocytes (D), M64 of cluster 2 astrocytes (E), or M15 of cluster 1 microglia (G). The pie chart of each node indicates whether it is a DEG or GWAS gene. The size of a node is proportional to the node connectivity in the MEGENA network. (UP: Upregulated; DN: Downregulated).

Figure 5. Bayesian Probabilistic Causal Netwwrk (BN) predicts novel key drivers in neuronal cells.

(A) BN in cluster 9 neurons.

(B) BN in cluster 7 neurons.

(C) Key driver identification in major cell types.

(D-F, H) The 2-layer network neighborhoods of *CADPS2* in cluster 9 neurons, *UCHL1* in cluster 7 neurons, *STMN2* in cluster 7 neurons, and *PREPL* in cluster 7 neurons, respectively. The size of a node is proportional to the node connectivity in the BN. The directionality of the arrow indicates the upstream-downstream effect. Nodes with burgundy labels are key drivers in that network.

(G). FET Enrichment for the overlap in vivo perturbation signature of STMN2 knockdown in mice and each n-layer neighborhood regulated by the STMN2 driver gene in cluster 7 neuron BN.

Figure 6. Bayesian Probabilistic Causal Network (BN) predicts novel key drivers in glial cells.

(A, C) BN in cluster 3 neurons (A), cluster 1 microglia (C).

(B, D, E) The 2-layer network neighborhoods of *MOBP* in cluster 3 oligodendrocytes, *APBB1IP* in cluster 1 microglia, *HSP90AA1* in cluster 5 oligodendrocyte progenitor cells. The size of a node is proportional to the node connectivity in the BN. The directionality of the arrow indicates the upstream-downstream effect. Nodes with burgundy labels are key drivers in that network.

METHODS

Postmortem substantia nigra pars compacta samples used

The framework we develop in this paper, is created using a discovery cohort from our previous study of human 23 PD cases and 9 controls that contain 30,038 genes across 315,867 nuclei (20). To validate some of our findings, we used three publicly available snRNA-seq datasets of human Substantia Nigra, Lee et al. dataset(21) of 6 PD and 6 Control cases. Martirosyan et al. dataset(22) of 15 PD and 14 Control, and Ma et al. (25)dataset of 11 PD and 13 Control.

snRNA-seq data preprocessing and clustering analysis

Three publicly available SnRNA-seq datasets were downloaded and subjected to the same pipeline we already established in the previous study(20). Briefly, from Cell Ranger–derived unique molecular identifier (UMI) count matrices from all sequencing libraries, we performed QC by removing low- quality nuclei with too few genes (<200) or an excessive number (>2500) of genes detected. Then we removed insufficiently detected genes and discarded mitochondrial genes. We then performed a pre-clustering analysis using scRNA-seq data integration workflow based on R packages Harmony(96) (v.1.2.3) and Seurat (v.4.3.0.1)(97, 98). A total of 2000 most variable gene features were identified, scaled, and centered after regression out covariates sex, age, and PMI. Next, dimensional reduction was performed using principal components analysis (PCA) based on the 2000 most variable genes. Preclustering analysis, cluster stability analysis and double prediction analysis were performed as previously described(20). As a result,

we obtained 28,339 genes across 44,450 high quality nuclei in KAIST dataset, 33,537 genes across 69,147 high quality nuclei in VIB dataset, and 31,280 genes across 194,905 high quality nuclei in Columbia dataset.

Cluster cell type annotation

For each major cluster of 3 external datasets, we interrogated the expression patterns of known gene markers to annotate clusters into major cell types: neurons (*SLC17A6, RBFOX3*, *GAD1*), astrocytes (*AQP4*), oligodendrocytes (*MOG*), microglia (*C3*, *CSF1R*), oligodendrocyte progenitor cells (*VCAN*), endothelial cells (*FLT1*), pericytes (*PDGFRB*), and T cell (*SKAP1*). Additionally, we used sets of marker genes we identified in our study such as typical dopaminergic markers in c7 (*SLC6A3*, *TH*, *SLC18A2*), enriched marker in c6 (*DCC* and *GALNTL6*), and novel enriched marker in c9 (*RIT2*).

Construction of Metacells

Metacells were built based on a log-normalized gene expression matrix using an R package SuperCell (v.1.0.1) (29). The set of features (genes) used for the construction of metacells was defined by the set of the most 2000 variable genes. The top principal components (top 10 by default) are used to build a single-cell network with a k-nearest neighbors algorithm built in the R package (k-NN, k =5 by default). To preserve the cell type annotation and phenotype information from snRNA-seq data, metacells were constructed by merging single cell (nodes) at a graining level $\gamma = 15$ at the cell cluster and condition specific manner. A gene expression profile of metacells was computed by averaging gene expression within metacells, or summing up the single cell raw values, followed by log normalization.

Identification of differentially expressed genes

DEGs between PD and control and DEGs between PD and control in each cluster were identified using the R package MAST (v.1.16.0) implemented in Seurat (97, 98), with correction for PMI, age, and sex. DEGs were identified at the cutoff of Bonferroni corrected P value ≤0.05 and fold change ≥10%

Identification of conserved markers

A conserved marker is defined as a cell type specific gene that distinguishes a given cell type from others and shows consistent expression across 2 groups (Control and PD). Conserved markers in each cluster were performed using the *FindConservedMarkers* function in the R Seurat package(97, 98). Genes that reached the average expression levels across the cluster with logFC >0 were identified as conserved markers.

Overlap and functional enrichment analysis

To understand biological processes underlying the DEGs and co-expressed modules, we tested these signatures for enrichment of gene ontology (GO) and canonical functional pathway gene sets. We used the functional gene sets collection from the Molecular Signature Database MSigDb that was downloaded from the R package called *msigdb (v.25.1.1)*. Functional enrichment of DEGs with MSigDB gene annotation collections was examined using the R package *clusterProfiler (v.4.14.6)*(99). To test the pathways of 2 layer-network neighborhood of candidate genes, we loaded the gene members onto *EnrichR* server and identified the top pathways associated with these genes(100-102). Functional enrichment analysis between two gene sets were determined by Fisher's exact test (FET). Fold Enrichment (FE) was calculated as the ratio between observed overlap and expected overlap size. We employed the Benjamini-Hochberg (BH) approach to correct for FDR.

Generation of Biologically meaningful correlation (BMC) matrix

We implemented a novel framework to generate biologically meaningful correlation (BMC) matrix. First, we filtered out unexpressed genes using a range of minimum cell cutoff (i.e. requiring that each gene be expressed in at least x% of cells expressed), selected genes that were conserved markers, and removed noisy cells (those expressing fewer than 500 genes). For each gene pair, we excluded zero counts before calculating all possible signed Spearman correlation coefficients (SCCs) (FDR = 1) using the *calculate.rho.signed* function in MEGENA (v.1.6.0)(32). To capture all signed correlation, we set direction as "positive" or "negative", then combined results from both directions.

Next, we evaluated whether the distribution of correlation coefficient (ρ) in each cell cluster followed a truncated normal distribution. For each cell cluster at each minimum cell cutoff, we first calculated the mean and standard deviation of the observed ρ values, and used to define a truncated normal distribution bounded by minimum and maximum observed ρ. The truncated normal density was generated at 100 equally spaced points across this range using the *truncnorm* function R package (v 1.0-9). We applied linear interpolation using *approx* function in R to estimate the histogram densities at 100 equally spaced points across the observed range of correlations. We then calculated Mean Square Error (MSE) using this equation: $MSE = \frac{1}{n}\sum_{i=1}^{n}\left(Y_i - \hat{Y}_i\right)$, where n is the number of evaluation points (100 in this case) in the correlation distribution, Y is the observed counts of the SCC, and $\hat{Y}$ is the predicted counts of SCCs from the stimulated truncated normalization distribution.

We then plotted MSE values against the minimum cell cutoff to identify the optimal cutoff. The mean (μ) of all gene pair correlation was calculated. Finally, at the optimal minimum cell cutoff, all significant gene pairs correlation (FDR < 0.05) were identified. However, even after applying FDR < 0.05, substantial fraction of gene pairs fell within the (0, μ) interval across clusters ranging from 0% in C9 to 47% in C5 **(Table S24)**. Therefore, at the optimal mincell cutoff, we further subtracted gene pairs in the (0, μ) interval. This new set of significant correlations, named BMC matrix, potentially removed weak and suspicious associations prior to network construction.

MEGENA co-expression network analysis

For each cell cluster, optimized BMC matrix was then subjected to MEGENA pipeline(32) (v.1.6.0). Briefly, significant SCCs were ranked and iteratively tested for planarity to grow a Planar Filtered Network (PFN) by using the PMFG algorithm. Multiscale Clustering Analysis (MCA) was conducted with the resulting PFN to identify co-expression modules at different network scale topology. We identified a total of 1057 modules in all cell clusters (**Table 3)**. To annotate the biological functions associated within the modules, we performed MSigDB gene set enrichment analysis using FET as

described above. Most of these modules (42.2% to 73.3%) were enriched for MSigDB GO Pathway gene sets (adjusted P value < 0.05 (**Table 3**), indicating that MEGENA can capture biologically functional signals. For simplicity, modules were annotated by top enriched pathway and then grouped into functional category. The MEGENA modules are formed in a hierarchy with parent-child relationship and illustrated by a sunburst plot using R package *ggsunburst*.

To prioritize the gene modules with respect to the PD phenotypes, we applied a ranking metric across multiple features such as enrichment with 1) the DEGs signatures identified and 2) PD GWAS signatures. PD GWAS hits were downloaded from GWAS catalogue and filtered to remove redundance. The ranking of the top modules was based on the average of -log10(Padj value) of all the features using this equation

$$Module\ Score_m = \frac{-log_{10}\left(P_{adj,m}^{Upregulated\ DEG}\right)+-log_{10}\left(P_{adj,m}^{Downregulated\ DEG}\right)+-log_{10}(P_{adj,m}^{GWAS})}{3},$$

where $P_{adj,m}^{Upregulated\ DEG}$, $P_{adj,m}^{Downregulated\ DEG}$, and $P_{adj,m}^{GWAS}$ are the adjusted P-values from the FET enrichment test for the DEGs and GWAS signatures, respectively, for module m. Higher scores indicate modules with stronger combined enrichment for disease associated transcriptional and genetic signals.

The circular track plot to visualize the gene co-expression network modules and their enrichment was generated using R package *NetWeaver (v.0.0.6)(103)*. The global network of c9 Neuron was visualized using Gephi (version 0.10.1). The global network of other cell clusters and subnetwork of selected gene modules were visualized using Cytoscape (version 3.10.3).

MEGENA co-expression module preservation analysis

We assessed module preservation in the MEGENA co-expression network between our MSSM SnRNAseq data and Columbia's snRNA-seq data using the Fisher's Exact Test, with genes from the corresponding global network in our dataset as the background. We performed pairwise comparisons of modules within the same cell type (e.g. microglia modules versus microglia modules).

Additionally, we used module preservation analysis in weighted gene correlation network analysis (WGCNA) (v.1.73)(37). For each cluster, MEGENA modules identified in the MSSM SnRNAseq dataset were used as reference modules, and module preservation was evaluated in the Columbia dataset. Expression matrices were restricted to genes available in both datasets. Because MEGENA allows a single gene to belong to multiple modules, each gene-module membership was treated as a separate feature. Prior to the preservation analysis, the expression datasets were evaluated using the goodSamplesGenes function, and the genes or the samples with zero variance or excessive missing values were removed. Module preservation was then evaluated using the modulePreservation function which took the MSSM gene coexpression networks as the reference networks and the Columbia dataset as the testing dataset. The composite module preservation statistics, Zsummary.pres, was calculated for each module. Modules with Zsummary.pres $\geqslant$ 10 were classified as highly preserved, modules with 2 < Zsummary.pres < 10 were considered moderately preserved, and modules with Zsummary.pres $\leqslant$ 2 were considered not preserved.

Bayesian probabilistic causal network inference

We constructed the global Bayesian probabilistic causal network (BN) based on a Monte Carlo Markov Chain simulation-based procedures using RIMBANet software(33, 34). We used established transcription factor (TF)-target relationships for *Homo sapiens* acquired from TFLink database(104) as prior to infer regulatory relationships among genes. In the causal network construction, the TFs are allowed to be parent nodes of their target genes, but targets are inhibited to be parent nodes of their TFs.

First, we began by removing genes expressed in fewer than 20% of metacells. To resolve the issue of dropouts in snRNA-seq, we applied an iterative pruning procedure to remove genes and nuclei in a data matrix. For this, we defined two thresholds: Tg, representing the maximum allowable gene missing rate, and Tm, representing the maximum allowable metacell missing rate. For each iteration, genes exceeding the Tg threshold and metacells exceeding the Tm threshold were removed. This

pruning process was repeated iteratively until the number of remaining genes and metacells stabilized, with no changes observed between iterations. After two iterations, the numbers converged. We then selected the cutoff Tg = 0.4 and Tm = 0.4 for all cell clusters.

We employed a network averaging approach where 1000 networks will be generated starting with a different random structure. Links that appear in more than 30% of the networks were used as a final consensus network. An iterative de-loop procedure was executed to ensure that the consensus network was a directed acyclic graph, removing the most-weakly supported link of all links involved in any loop. The global network of cell clusters was visualized using Cytoscape (version 3.10.3).

Key driver analysis (KDA)

For each major cell type cluster, we performed key driver analysis (KDA)(35) on the consensus Bayesian network to identify key hub genes that are DEGs and regulate a large number of downstream nodes. Here we loaded all the BN nodes and DEGs from the corresponding cluster as input for KDA, making the resulting key drivers' representative of the global network. The algorithm defined a background subnetwork by identifying nodes within an L-layer neighborhood of each DEG in the network. For each node in this subnetwork, it assessed enrichment within its L-layer downstream neighborhood for DEGs. In our analysis, we tested combination of 1-layer (1L), 2-layer (2L), and 3-layer (3L) neighborhoods, considering both directed and undirected edges to account for potential missing directionality in the causal BN. Then key drivers were compiled based on those identified from the 3L directed analysis and those shared by both 2L and 1L undirected analyses. To ensure that key drivers had sufficient downstream targets, we also excluded drivers with a degree lower than the average degree of all nodes in the network. KDA was performed by the R package *KDA* (version 0.02).

PubMed literature mining

The function EUtilsSummary (type = "esearch", db="pubmed", datetype='pdat') from the R package RISmed (version 2.3.0) was used to query and download content from Pubmed. The query words were built

as “KDG and Parkinson's disease” where the word KDG was replaced by the gene symbol of a specific key driver gene. For example, the query of “CADPS2 and Parkinson's disease” would pull out all the papers that have the occurrence of CADPS2 and Parkinson's disease.

Statistical Analysis

Fisher’s exact test (FET) was used to examine the significance of the overlap between two gene sets. An overlap with BH corrected $p < 0.05$ was considered to be statistically significant unless stated otherwise.

REFERENCES

1. De Lau LM, Breteler MM. Epidemiology of Parkinson's disease. The Lancet Neurology. 2006;5(6):525-35.10.1016/S1474-4422(06)70471-9
2. Parkinson J. An essay on the shaking palsy. The Journal of neuropsychiatry and clinical neurosciences. 2002;14(2):223-36.10.1176/jnp.14.2.223
3. Chu Y, Kordower JH. Age-associated increases of α-synuclein in monkeys and humans are associated with nigrostriatal dopamine depletion: is this the target for Parkinson's disease? Neurobiology of disease. 2007;25(1):134-49.10.1016/j.nbd.2006.08.021
4. Pakkenberg B, Møller A, Gundersen HJ, Dam AM, Pakkenberg H. The absolute number of nerve cells in substantia nigra in normal subjects and in patients with Parkinson's disease estimated with an unbiased stereological method. Journal of Neurology, Neurosurgery & Psychiatry. 1991;54(1):30-3.10.1136/jnnp.54.1.30
5. Blauwendraat C, Nalls MA, Singleton AB. The genetic architecture of Parkinson's disease. The Lancet Neurology. 2020;19(2):170-8.10.1016/S1474-4422(19)30287-X
6. Nalls MA, Blauwendraat C, Vallerga CL, Heilbron K, Bandres-Ciga S, Chang D, et al. Identification of novel risk loci, causal insights, and heritable risk for Parkinson's disease: a meta-analysis of genome-wide association studies. The Lancet Neurology. 2019;18(12):1091-102.10.1016/S1474-4422(19)30320-5
7. McGeer PL, Itagaki S, Boyes BE, McGeer E. Reactive microglia are positive for HLA-DR in the substantia nigra of Parkinson's and Alzheimer's disease brains. Neurology. 1988;38(8):1285-.10.1212/WNL.38.8.1285
8. Le W-d, Rowe D, Xie W, Ortiz I, He Y, Appel SH. Microglial activation and dopaminergic cell injury: an in vitro model relevant to Parkinson's disease. Journal of Neuroscience. 2001;21(21):8447-55.10.1523/JNEUROSCI.21-21-08447.2001
9. Gao HM, Jiang J, Wilson B, Zhang W, Hong JS, Liu B. Microglial activation-mediated delayed and progressive degeneration of rat nigral dopaminergic neurons: relevance to Parkinson's disease. Journal of neurochemistry. 2002;81(6):1285-97.10.1046/j.1471-4159.2002.00928.x
10. Zhang W, Wang T, Pei Z, Miller DS, Wu X, Block ML, et al. Aggregated α-synuclein activates microglia: a process leading to disease progression in Parkinson's disease. The FASEB Journal. 2005;19(6):533-42.10.1096/fj.04-2751com

11. Zhang W, Phillips K, Wielgus AR, Liu J, Albertini A, Zucca FA, et al. Neuromelanin activates microglia and induces degeneration of dopaminergic neurons: implications for progression of Parkinson’s disease. Neurotoxicity research. 2011;19:63-72.10.1007/s12640-009-9140-z

12. Waak J, Weber SS, Waldenmaier A, Görner K, Alunni-Fabbroni M, Schell H, et al. Regulation of astrocyte inflammatory responses by the Parkinson's disease-associated gene DJ–1. The FASEB Journal. 2009;23(8):2478-89.10.1096/fj.08-125153

13. Gray MT, Woulfe JM. Striatal blood–brain barrier permeability in Parkinson's disease. Journal of Cerebral Blood Flow & Metabolism. 2015;35(5):747-50.10.1038/jcbfm.2015.32

14. Kam T-I, Hinkle JT, Dawson TM, Dawson VL. Microglia and astrocyte dysfunction in parkinson's disease. Neurobiology of disease. 2020;144:105028.10.1016/j.nbd.2020.105028

15. Hirsch EC, Hunot S. Neuroinflammation in Parkinson's disease: a target for neuroprotection? The Lancet Neurology. 2009;8(4):382-97.10.1016/S1474-4422(09)70062-6

16. Bae E-J, Pérez-Acuña D, Rhee KH, Lee S-J. Changes in oligodendroglial subpopulations in Parkinson’s disease. Molecular brain. 2023;16(1):65.10.1186/s13041-023-01055-5

17. Azevedo C, Teku G, Pomeshchik Y, Reyes JF, Chumarina M, Russ K, et al. Parkinson’s disease and multiple system atrophy patient iPSC-derived oligodendrocytes exhibit alpha-synuclein–induced changes in maturation and immune reactive properties. Proceedings of the National Academy of Sciences. 2022;119(12):e2111405119.10.1073/pnas.2111405119

18. Djelloul M, Holmqvist S, Boza-Serrano A, Azevedo C, Yeung MS, Goldwurm S, et al. Alpha-synuclein expression in the oligodendrocyte lineage: an in vitro and in vivo study using rodent and human models. Stem cell reports. 2015;5(2):174-84.10.1016/S1474-4422(06)70471-9

19. Wang Q, Zhang Y, Wang M, Song W-M, Shen Q, McKenzie A, et al. The landscape of multiscale transcriptomic networks and key regulators in Parkinson’s disease. Nature communications. 2019;10(1):5234. 10.1038/s41467-019-13144-y

20. Wang Q, Wang M, Choi I, Sarrafha L, Liang M, Ho L, et al. Molecular profiling of human substantia nigra identifies diverse neuron types associated with vulnerability in Parkinson’s disease. Science advances. 2024;10(2):eadi8287.10.1126/sciadv.adi8287

21. Lee AJ, Kim C, Park S, Joo J, Choi B, Yang D, et al. Characterization of altered molecular mechanisms in Parkinson’s disease through cell type–resolved multiomics analyses. Science advances. 2023;9(15):eabo2467.10.1126/sciadv.abo2467

22. Martirosyan A, Ansari R, Pestana F, Hebestreit K, Gasparyan H, Aleksanyan R, et al. Unravelling cell type-specific responses to Parkinson’s Disease at single cell resolution. Molecular neurodegeneration. 2024;19(1):1-24.10.1186/s13024-023-00699-0

23. Adams L, Song MK, Yuen S, Tanaka Y, Kim Y-S. A single-nuclei paired multiomic analysis of the human midbrain reveals age-and Parkinson’s disease–associated glial changes. Nature aging. 2024;4(3):364-78.10.1038/s43587-024-00583-6

24. Chatila Z, Yadav A, Mares J, Flowers X, Yun T, Rashid M, et al. RNA-and ATAC-sequencing reveals a unique CD83+ microglial population focally depleted in Parkinson’s disease. BioRxiv. 2023:2023.05. 17.540842.10.1101/2023.05.17.540842

25. Ma M, Paryani F, Jakubiak K, Xia S, Antoku S, Kannan A, et al. The spatial landscape of glial pathology and T cell response in Parkinson’s disease substantia nigra. Nature Communications. 2025;16(1):7146.10.1038/s41467-025-62478-3

26. Cheng Y, Ma X, Yuan L, Sun Z, Wang P. Evaluating imputation methods for single-cell RNA-seq data. BMC bioinformatics. 2023;24(1):302.10.1186/s12859-023-05417-7

27. Baran Y, Bercovich A, Sebe-Pedros A, Lubling Y, Giladi A, Chomsky E, et al. MetaCell: analysis of single-cell RNA-seq data using K-nn graph partitions. Genome biology. 2019;20:1-19.10.1186/s13059-019-1812-2

28. Ben-Kiki O, Bercovich A, Lifshitz A, Tanay A. Metacell-2: a divide-and-conquer metacell algorithm for scalable scRNA-seq analysis. Genome biology. 2022;23(1):100.10.1186/s13059-022-02667-1

29. Bilous M, Tran L, Cianciaruso C, Gabriel A, Michel H, Carmona SJ, et al. Metacells untangle large and complex single-cell transcriptome networks. BMC bioinformatics. 2022;23(1):336. 10.1186/s12859-022-04861-1

30. Persad S, Choo Z-N, Dien C, Sohail N, Masilionis I, Chaligné R, et al. SEACells infers transcriptional and epigenomic cellular states from single-cell genomics data. Nature Biotechnology. 2023;41(12):1746-57.10.1038/s41587-023-01716-9

31. Liang M, Chu L, Yue Z. New Multiomic Studies Shed Light on Cellular Diversity and Neuronal Susceptibility in Parkinson's Disease. Movement Disorders. 2025;40(3):431-7.10.1002/mds.30097

32. Song W-M, Zhang B. Multiscale embedded gene co-expression network analysis. PLoS computational biology. 2015;11(11):e1004574.10.1371/journal.pcbi.1004574

33. Zhu J, Zhang B, Smith EN, Drees B, Brem RB, Kruglyak L, et al. Integrating large-scale functional genomic data to dissect the complexity of yeast regulatory networks. Nature genetics. 2008;40(7):854-61.10.1038/ng.167

34. Zhu J, Wiener MC, Zhang C, Fridman A, Minch E, Lum PY, et al. Increasing the power to detect causal associations by combining genotypic and expression data in segregating populations. PLoS computational biology. 2007;3(4):e69.10.1371/journal.pcbi.0030069

35. Zhang B, Zhu J, editors. Identification of key causal regulators in gene networks. Proceedings of the World Congress on Engineering; 2013.

36. Sollis E, Mosaku A, Abid A, Buniello A, Cerezo M, Gil L, et al. The NHGRI-EBI GWAS Catalog: knowledgebase and deposition resource. Nucleic acids research. 2023;51(D1):D977-D85.10.1093/nar/gkac1010

37. Langfelder P, Horvath S. WGCNA: an R package for weighted correlation network analysis. BMC bioinformatics. 2008;9(1):559.10.1186/1471-2105-9-559

38. Wang M, Li A, Sekiya M, Beckmann ND, Quan X, Schrode N, et al. Transformative Network Modeling of Multi-Omics Data Reveals Detailed Circuits, Key Regulators, and Potential Therapeutics for Alzheimer's Disease. Neuron. 2020; S0896-6273(20):8.10.1016/j.neuron.2020.11.002

39. Wang Q, Zhang Y, Wang M, Song WM, Shen Q, McKenzie A, et al. The landscape of multiscale transcriptomic networks and key regulators in Parkinson's disease. Nat Commun. 2019;10(1):5234.10.1038/s41467-019-13144-y
40. Grishanin RN, Kowalchyk JA, Klenchin VA, Ann K, Earles CA, Chapman ER, et al. CAPS acts at a prefusion step in dense-core vesicle exocytosis as a PIP2 binding protein. Neuron. 2004;43(4):551-62.10.1016/j.neuron.2004.07.028
41. Zhang L, Wei Z, Dai Y, He F, Sun T. The role of CAPS in Ca2+-regulated exocytosis: promotion of vesicle tethering, priming, and fusion. Neuropharmacology. 2024:110247.10.1016/j.neuropharm.2024.110247
42. Iguchi H, Katsuzawa T, Saruta C, Sadakata T, Kobayashi S, Sato Y, et al. Calcium-dependent activator protein for secretion 2 is involved in dopamine release in mouse midbrain neurons. Frontiers in Molecular Neuroscience. 2024;17:1444629.10.3389/fnmol.2024.1444629
43. Smajić S, Prada-Medina CA, Landoulsi Z, Ghelfi J, Delcambre S, Dietrich C, et al. Single-cell sequencing of human midbrain reveals glial activation and a Parkinson-specific neuronal state. Brain. 2022;145(3):964-78.10.1093/brain/awab446
44. Mukherjee S, Goswami S, Dash S, Samanta D. Structural basis of molecular recognition among classical cadherins mediating cell adhesion. Biochemical Society Transactions. 2023;51(6):2103-15.10.1042/BST20230356
45. Moreland T, Poulain FE. To stick or not to stick: The multiple roles of cell adhesion molecules in neural circuit assembly. Frontiers in Neuroscience. 2022;16:889155.10.3389/fnins.2022.889155
46. Steinberg C, Roston TM, van der Werf C, Sanatani S, Chen SW, Wilde AA, et al. RYR2-ryanodinopathies: from calcium overload to calcium deficiency. Europace. 2023;25(6):euad156.10.1093/europace/euad156
47. Ansari U, Chen V, Sedighi R, Syed B, Muttalib Z, Ansari K, et al. Role of the UNC13 family in human diseases: A literature review. AIMS neuroscience. 2023;10(4):388.10.3934/Neuroscience.2023029
48. Liu Y, Fallon L, Lashuel HA, Liu Z, Lansbury PT. The UCH-L1 gene encodes two opposing enzymatic activities that affect α-synuclein degradation and Parkinson's disease susceptibility. Cell. 2002;111(2):209-18.10.1016/S0092-8674(02)01012-7
49. Yang J, Kong L, Zou L, Liu Y. The role of synaptic protein NSF in the development and progression of neurological diseases. Frontiers in Neuroscience. 2024;18:1395294.10.3389/fnins.2024.1395294
50. Burré J, Sharma M, Tsetsenis T, Buchman V, Etherton MR, Südhof TC. α-Synuclein promotes SNARE-complex assembly in vivo and in vitro. Science. 2010;329(5999):1663-7.10.1126/science.1195227
51. Miller RM, Callahan LM, Casaceli C, Chen L, Kiser GL, Chui B, et al. Dysregulation of gene expression in the 1-methyl-4-phenyl-1, 2, 3, 6-tetrahydropyridine-lesioned mouse substantia nigra. Journal of Neuroscience. 2004;24(34):7445-54.10.1523/JNEUROSCI.4204-03.2004
52. Gao X, Huang Z, Feng C, Guan C, Li R, Xie H, et al. Multimodal analysis of gene expression from postmortem brains and blood identifies synaptic vesicle trafficking genes to be associated with Parkinson’s disease. Briefings in bioinformatics. 2021;22(4):bbaa244.10.1093/bib/bbaa244

53. Li Z, Langhans SA. Transcriptional regulators of Na, K-ATPase subunits. Frontiers in cell and developmental biology. 2015;3:66.10.3389/fcell.2015.00066

54. Paulussen I, Beckert H, Musial TF, Gschossmann LJ, Wolf J, Schmitt M, et al. SV2B defines a subpopulation of synaptic vesicles. Journal of Molecular Cell Biology. 2023;15(9):mjad054.10.1093/jmcb/mjad054

55. Janz R, Goda Y, Geppert M, Missler M, Südhof TC. SV2A and SV2B function as redundant Ca2+ regulators in neurotransmitter release. Neuron. 1999;24(4):1003-16.10.1016/S0896-6273(00)81046-6

56. Thornburg-Suresh EJ, Summers DW. Microtubules, Membranes, and Movement: New Roles for Stathmin-2 in Axon Integrity. Journal of neuroscience research. 2024;102(9):e25382.10.1002/jnr.25382

57. Gagliardi D, Pagliari E, Meneri M, Melzi V, Rizzo F, Comi GP, et al. Stathmins and motor neuron diseases: pathophysiology and therapeutic targets. Biomedicines. 2022;10(3):711.10.3390/biomedicines10030711

58. San Juan IG, Nash LA, Smith KS, Leyton-Jaimes MF, Qian M, Klim JR, et al. Loss of mouse Stmn2 function causes motor neuropathy. Neuron. 2022;110(10):1671-88. e6.10.1016/j.neuron.2022.02.011

59. Duarte ML, Wang M, Gomes I, Liu C, Sharma A, Fakira AK, et al. Multiomics Analyses Identify Proline Endopeptidase–Like Protein As a Key Regulator of Protein Trafficking, a Pathway Underlying Alzheimer’s Disease Pathogenesis. Molecular Pharmacology. 2023;104(1):1-16.10.1124/molpharm.122.000641

60. Morawski M, Nuytens K, Juhasz T, Zeitschel U, Seeger G, Waelkens E, et al. Cellular and ultra structural evidence for cytoskeletal localization of prolyl endopeptidase-like protein in neurons. Neuroscience. 2013;242:128-39.10.1016/j.neuroscience.2013.02.038

61. Santos GS, Oyadomari WY, Carvalho EA, Torquato RS, Oliveira V. Prolyl endopeptidase-like facilitates the α-synuclein aggregation seeding, and this effect is reverted by serine peptidase inhibitor PMSF. Biomolecules. 2020;10(6):962.10.3390/biom10060962

62. Joset P, Wacker A, Babey R, Ingold EA, Andermatt I, Stoeckli ET, et al. Rostral growth of commissural axons requires the cell adhesion molecule MDGA2. Neural development. 2011;6:1-16.10.1186/1749-8104-6-22

63. Latourelle JC, Pankratz N, Dumitriu A, Wilk JB, Goldwurm S, Pezzoli G, et al. Genomewide association study for onset age in Parkinson disease. BMC medical genetics. 2009;10:1-14.10.1186/1471-2350-10-98

64. Montague P, McCallion AS, Davies RW, Griffiths IR. Myelin-associated oligodendrocytic basic protein: a family of abundant CNS myelin proteins in search of a function. Developmental neuroscience. 2006;28(6):479-87.10.1159/000095110

65. Yamamoto Y, Yoshikawa H, Nagano S, Kondoh G, Sadahiro S, Gotow T, et al. Myelin-associated oligodendrocytic basic protein is essential for normal arrangement of the radial component in central nervous system myelin. European Journal of Neuroscience. 1999;11(3):847-55.10.1046/j.1460-9568.1999.00490.x

66. Rajicic A, Giannini LA, Gerrits E, van Buuren R, Melhem S, Slotman JA, et al. WDR49-Positive Astrocytes Mark Severity of Neurodegeneration in Frontotemporal Lobar Degeneration and Alzheimer's Disease. Glia. 2025;73(5):948-68.10.1002/glia.24663

67. Mizuno S, Sasai H, Kume A, Takahashi D, Satoh M, Kado S, et al. Dioleoyl-phosphatidic acid selectively binds to α-synuclein and strongly induces its aggregation. FEBS letters. 2017;591(5):784-91.10.1002/1873-3468.12592
68. Medraño-Fernandez I, Reyes R, Olazabal I, Rodriguez E, Sanchez-Madrid F, Boussiotis VA, et al. RIAM (Rap1-interacting adaptor molecule) regulates complement-dependent phagocytosis. Cellular and Molecular Life Sciences. 2013;70:2395-410.10.1007/s00018-013-1268-
69. Klapproth S, Sperandio M, Pinheiro EM, Prünster M, Soehnlein O, Gertler FB, et al. Loss of the Rap1 effector RIAM results in leukocyte adhesion deficiency due to impaired β2 integrin function in mice. Blood, The Journal of the American Society of Hematology. 2015;126(25):2704-12.10.1182/blood-2015-05-647453
70. Irons EE, Punch PR, Lau JT. Blood-borne ST6GAL1 regulates immunoglobulin production in B cells. Frontiers in Immunology. 2020;11:617.10.3389/fimmu.2020.00617
71. Holdbrooks AT, Ankenbauer KE, Hwang J, Bellis SL. Regulation of inflammatory signaling by the ST6Gal-I sialyltransferase. PLoS One. 2020;15(11):e0241850.10.1371/journal.pone.0241850
72. Bhattacharya A, Fon EA, Dagher A, Iturria-Medina Y, Stratton JA, Savignac C, et al. Cell type transcriptomic modules reveal shared molecular mechanisms in Alzheimer’s and Parkinson’s disease. GigaScience. 2026;15:giag059.10.1093/gigascience/giag059
73. Young MD, Behjati S. SoupX removes ambient RNA contamination from droplet-based single-cell RNA sequencing data. Gigascience. 2020;9(12):giaa151.10.1093/gigascience/giaa151
74. Fleming SJ, Chaffin MD, Arduini A, Akkad A-D, Banks E, Marioni JC, et al. Unsupervised removal of systematic background noise from droplet-based single-cell experiments using CellBender. Nature methods. 2023;20(9):1323-35.10.1038/s41592-023-01943-7
75. Poulin J-F, Zou J, Drouin-Ouellet J, Kim K-YA, Cicchetti F, Awatramani RB. Defining midbrain dopaminergic neuron diversity by single-cell gene expression profiling. Cell reports. 2014;9(3):930-43.10.1016/j.celrep.2014.10.008
76. La Manno G, Gyllborg D, Codeluppi S, Nishimura K, Salto C, Zeisel A, et al. Molecular diversity of midbrain development in mouse, human, and stem cells. Cell. 2016;167(2):566-80. e19.10.1016/j.cell.2016.09.027
77. Kamath T, Abdulraouf A, Burris SJ, Langlieb J, Gazestani V, Nadaf NM, et al. Single-cell genomic profiling of human dopamine neurons identifies a population that selectively degenerates in Parkinson’s disease. Nature neuroscience. 2022;25(5):588-95.10.1038/s41593-022-01061-1
78. Obergasteiger J, Überbacher C, Pramstaller PP, Hicks AA, Corti C, Volta M. CADPS2 gene expression is oppositely regulated by LRRK2 and alpha-synuclein. Biochemical and biophysical research communications. 2017;490(3):876-81.10.1016/j.bbrc.2017.06.134
79. Tang L, Xu N, Huang M, Yi W, Sang X, Shao M, et al. A primate nigrostriatal atlas of neuronal vulnerability and resilience in a model of Parkinson’s disease. Nature Communications. 2023;14(1):7497.10.1038/s41467-023-43213-2

80. Surmeier DJ, Obeso JA, Halliday GM. Selective neuronal vulnerability in Parkinson disease. Nature Reviews Neuroscience. 2017;18(2):101-13.10.1038/nrn.2016.178
81. Stadelmann C, Timmler S, Barrantes-Freer A, Simons M. Myelin in the central nervous system: structure, function, and pathology. Physiological reviews. 2019;99(3):1381-431.10.1152/physrev.00031.2018
82. Baumann N, Pham-Dinh D. Biology of oligodendrocyte and myelin in the mammalian central nervous system. Physiological reviews. 2001;81(2):871-927.10.1152/physrev.2001.81.2.871
83. Siokas V, Aloizou AM, Liampas I, Bakirtzis C, Tsouris Z, Sgantzos M, et al. Myelin-associated oligodendrocyte basic protein rs616147 polymorphism as a risk factor for Parkinson's disease. Acta Neurologica Scandinavica. 2022;145(2):223-8.10.1111/ane.13538
84. Tomkins JE, Methi A, Bouland G, Doludda B, Murphy K, Miller JA, et al. Genetic modulation of immune gene co-expression in the aged mouse hippocampus by the Apbb1ip locus. bioRxiv. 2025:2025.06. 04.657838.10.1101/2025.06.04.657838
85. Geirsdottir L, David E, Keren-Shaul H, Weiner A, Bohlen SC, Neuber J, et al. Cross-species single-cell analysis reveals divergence of the primate microglia program. Cell. 2019;179(7):1609-22. e16.10.1016/j.cell.2019.11.010
86. Boche D, Gordon MN. Diversity of transcriptomic microglial phenotypes in aging and Alzheimer's disease. Alzheimer's & Dementia. 2022;18(2):360-76.10.1002/alz.12389
87. Morgan A, Turic D, Jehu L, Hamilton G, Hollingworth P, Moskvina V, et al. Association studies of 23 positional/functional candidate genes on chromosome 10 in late-onset Alzheimer's disease. American Journal of Medical Genetics Part B: Neuropsychiatric Genetics. 2007;144(6):762-70.10.1002/ajmg.b.30509
88. Ashbrook DG, Cahill S, Hager R. A cross-species systems genetics analysis links APBB1IP as a candidate for schizophrenia and prepulse inhibition. Frontiers in behavioral neuroscience. 2019;13:266.10.3389/fnbeh.2019.00266
89. Hunt AP, Minett GM, Gibson OR, Kerr GK, Stewart IB. Could heat therapy be an effective treatment for Alzheimer's and Parkinson's diseases? A narrative review. Frontiers in physiology. 2020;10:1556.10.3389/fphys.2019.01556
90. Saxena SK, Sharma D, Kumar S, Maurya VK, Ansari S, Malhotra HS, et al. Decoding the role of large heat shock proteins in the progression of neuroinflammation-mediated neurodegenerative disorders. Neuroprotection. 2025.10.1002/nep3.68
91. Bohush A, Bieganowski P, Filipek A. Hsp90 and its co-chaperones in neurodegenerative diseases. International journal of molecular sciences. 2019;20(20):4976.10.3390/ijms20204976
92. Li H, Yang J, Wang Y, Liu Q, Cheng J, Wang F. Neuroprotective effects of increasing levels of HSP70 against neuroinflammation in Parkinson's disease model by inhibition of NF-κB and STAT3. Life Sciences. 2019;234:116747.10.1016/j.lfs.2019.116747
93. Klucken J, Shin Y, Masliah E, Hyman BT, McLean PJ. Hsp70 reduces α-synuclein aggregation and toxicity. Journal of Biological Chemistry. 2004;279(24):25497-502.10.1074/jbc.M400255200

94. Zhu B, Park J-M, Coffey SR, Russo A, Hsu I-U, Wang J, et al. Single-cell transcriptomic and proteomic analysis of Parkinson’s disease Brains. Science Translational Medicine. 2024;16(771):eabo1997.10.1126/scitranslmed.abo1997
95. Jang Y, Pletnikova O, Troncoso JC, Pantelyat AY, Dawson TM, Rosenthal LS, et al. Mass spectrometry–based proteomics analysis of human substantia nigra from Parkinson's disease patients identifies multiple pathways potentially involved in the disease. Molecular & Cellular Proteomics. 2023;22(1).10.1016/j.mcpro.2022.100452
96. Korsunsky I, Millard N, Fan J, Slowikowski K, Zhang F, Wei K, et al. Fast, sensitive and accurate integration of single-cell data with Harmony. Nature methods. 2019;16(12):1289-96.10.1038/s41592-019-0619-0
97. Butler A, Hoffman P, Smibert P, Papalexi E, Satija R. Integrating single-cell transcriptomic data across different conditions, technologies, and species. Nature biotechnology. 2018;36(5):411-20.10.1038/nbt.4096
98. Satija R, Farrell JA, Gennert D, Schier AF, Regev A. Spatial reconstruction of single-cell gene expression data. Nature biotechnology. 2015;33(5):495-502.10.1038/nbt.3192
99. Yu G, Wang L-G, Han Y, He Q-Y. clusterProfiler: an R package for comparing biological themes among gene clusters. Omics: a journal of integrative biology. 2012;16(5):284-7.10.1089/omi.2011.0118
100. Chen EY, Tan CM, Kou Y, Duan Q, Wang Z, Meirelles GV, et al. Enrichr: interactive and collaborative HTML5 gene list enrichment analysis tool. BMC bioinformatics. 2013;14:1-14.10.1186/1471-2105-14-128
101. Kuleshov MV, Jones MR, Rouillard AD, Fernandez NF, Duan Q, Wang Z, et al. Enrichr: a comprehensive gene set enrichment analysis web server 2016 update. Nucleic acids research. 2016;44(W1):W90-W7.10.1093/nar/gkw377
102. Xie Z, Bailey A, Kuleshov MV, Clarke DJ, Evangelista JE, Jenkins SL, et al. Gene set knowledge discovery with Enrichr. Current protocols. 2021;1(3):e90.10.1002/cpz1.90
103. Wang M, Roussos P, McKenzie A, Zhou X, Kajiwara Y, Brennand KJ, et al. Integrative network analysis of nineteen brain regions identifies molecular signatures and networks underlying selective regional vulnerability to Alzheimer’s disease. Genome medicine. 2016;8(1):104.10.1186/s13073-016-0355-3
104. Liska O, Bohár B, Hidas A, Korcsmáros T, Papp B, Fazekas D, et al. TFLink: an integrated gateway to access transcription factor–target gene interactions for multiple species. Database. 2022;2022:baac083.10.1093/database/baac083
105. Chu L, ZHANG B. Multiscale Transcriptomic Network Models of Parkinson's Disease at the Single-Cell Level. GitHub repository. https://github.com/linhchu-3/PD_metacell_network.
106. Chu L, ZHANG B. MetaCell-based Multiscale Network Analysis (MCMNA). Zenodo; 2026. doi:10.5281/zenodo.20834300

Click here to access/download;Table;Table 1. Metacell-size.xlsx

Table 1. Number of metacell in major cell types

Cluster	Metacell number		
	Control	PD	Total
C0 Oli	1743	7191	8934
C1 Mic	438	1545	1983
C2 Ast	306	1457	1763
C3 Oli	441	1433	1874
C4 End	290	1182	1472
C5 OPC	204	1157	1361
C6 Neu	288	923	1211
C7 Neu	419	848	1267
C8 Per	130	520	650
C9 Neu	183	99	282

Table 2

Click here to access/download;Table;Table 2. DEG-size_by-Metacell.xlsx

Cluster	Number of DEGs identified by Metacell (average, unweighted)		
	Upregulated DEGs	Downregulated DEGs	Total
**C0 Oli**	83	98	181
**C1 Mic**	77	105	182
**C2 Ast**	58	142	200
**C3 Oli**	124	137	261
**C4 End**	136	238	374
**C5 OPC**	49	39	88
**C6 Neu**	149	68	217
**C7 Neu**	194	514	708
**C8 Per**	67	114	181
**C9 Neu**	149	899	1048
**C10 Fib**	0	1	1
**C11 T**	0	0	0

Table 3

Click here to access/download;Table;Table 3. Summary of MEGENA modules.xlsx

Cluster	Cell type	Number of modules	Numer of signficant modules	Note	Number of module with pathway annotation
C0	Oligodendrocyte	104	70	Major cell type	33
C1	Microglia	129	93	Major cell type	63
C2	Astrocyte	113	91	Major cell type	42
C3	Oligodendrocyte	265	204	Major cell type	97
C4	Endothelia	109	75		55
C5	Oligodendrocyte progenitor cell	145	102	Major cell type	43
C6	Neuron	275	165	Major cell type	71
C7	Neuron	63	53	Major cell type	27
C8	Pericyte	238	149		94
C9	Neuron	72	55	Major cell type	26
	Total in all cell types		1057		
	Total modules in major cell types		833		

Percentage of module with pathway enrichment	Numer of modules associated with upregulated DEGs	Numer of modules associated with downregulated DEGs	Numer of modules associated with GWAS genes
47.1%	5	11	0
67.7%	8	12	0
46.2%	5	16	1
47.5%	18	14	0
73.3%	11	23	2
42.2%	0	8	0
43.0%	11	8	1
50.9%	0	1	0
63.1%	11	15	1
47.3%	0	17	1

Click here to access/download;Table;Table 4. Summary of Key Driver Genes.xlsx

Gene	FDR network	Clusterid	Cell_type	DEGs
MOBP	9.21226E-42	Cluster 3	Oli	DOWN
CADPS2	1.89876E-39	Cluster 9	Neu	DOWN
SNAP25	4.58012E-38	Cluster 7	Neu	DOWN
NSF	5.18355E-34	Cluster 7	Neu	DOWN
TUBA1B	1.09898E-26	Cluster 7	Neu	DOWN
CTNNA3	9.14221E-23	Cluster 6	Neu	UP
CDH18	2.90425E-21	Cluster 9	Neu	DOWN
QKI	8.26895E-21	Cluster 6	Neu	UP
ATP1B1	1.89952E-20	Cluster 7	Neu	DOWN
LMCD1-AS1	2.2634E-20	Cluster 0	Oli	DOWN
YWHAG	1.05962E-19	Cluster 7	Neu	DOWN
AC016831.7	5.93343E-19	Cluster 3	Oli	UP
STXBP1	8.40587E-19	Cluster 7	Neu	DOWN
ATP6V1B2	1.45309E-18	Cluster 7	Neu	DOWN
PREPL	1.68954E-18	Cluster 7	Neu	DOWN
ST18	3.43037E-18	Cluster 6	Neu	UP
PPP2R2B	3.08793E-17	Cluster 0	Oli	DOWN
RIMS1	1.05017E-16	Cluster 9	Neu	DOWN
CIRBP	2.83015E-16	Cluster 3	Oli	DOWN
AGPAT4	3.16844E-16	Cluster 0	Oli	DOWN
ZNF385D	5.02798E-16	Cluster 9	Neu	DOWN
PPP2R1A	2.02198E-15	Cluster 7	Neu	DOWN
TAGLN3	2.43878E-15	Cluster 7	Neu	DOWN
MOBP	2.66953E-15	Cluster 0	Oli	DOWN
NCKAP5	9.53044E-15	Cluster 6	Neu	UP
INA	1.04713E-14	Cluster 7	Neu	DOWN
GRIK2	1.3459E-14	Cluster 9	Neu	DOWN
LINC01505	2.38875E-14	Cluster 3	Oli	DOWN
NAALADL2	4.05882E-14	Cluster 0	Oli	DOWN
RNF220	4.18166E-14	Cluster 6	Neu	UP
NDRG4	5.66485E-14	Cluster 7	Neu	DOWN
NEAT1	9.28666E-14	Cluster 3	Oli	UP
HSP90AB1	1.6997E-13	Cluster 7	Neu	DOWN
RTN3	2.31924E-13	Cluster 7	Neu	DOWN
SV2B	2.37708E-13	Cluster 7	Neu	DOWN
SYT1	2.65429E-13	Cluster 7	Neu	DOWN
SLC9A6	6.72434E-13	Cluster 7	Neu	DOWN
PRDX3	7.84196E-13	Cluster 7	Neu	DOWN
NEFL	1.45787E-12	Cluster 7	Neu	DOWN
TRPM3	1.61898E-12	Cluster 9	Neu	DOWN
SV2A	2.0525E-12	Cluster 7	Neu	DOWN
DYNLT3	2.3045E-12	Cluster 7	Neu	DOWN
CHORDC1	2.41788E-12	Cluster 3	Oli	UP
ZFPM2	4.59149E-12	Cluster 9	Neu	DOWN
WDR49	7.65653E-12	Cluster 2	Ast	DOWN
SLC35F3	1.1931E-11	Cluster 3	Oli	UP
IRS2	1.61137E-11	Cluster 3	Oli	UP

NAPB	1.69433E-11	Cluster 7	Neu	DOWN
FSTL5	2.02846E-11	Cluster 9	Neu	DOWN
NRXN3	2.9778E-11	Cluster 0	Oli	DOWN
HSP90AA1	3.60675E-11	Cluster 5	OPC	UP
TSPYL4	4.48731E-11	Cluster 7	Neu	DOWN
HSPH1	5.04294E-11	Cluster 3	Oli	UP
RELN	9.0848E-11	Cluster 9	Neu	DOWN
CARNS1	1.06311E-10	Cluster 0	Oli	DOWN
PSD3	1.50424E-10	Cluster 0	Oli	DOWN
HMGB1	1.65668E-10	Cluster 3	Oli	UP
CHN1	2.51511E-10	Cluster 7	Neu	DOWN
PLEKHH1	2.58888E-10	Cluster 3	Oli	DOWN
CDIP1	2.93404E-10	Cluster 7	Neu	DOWN
OAT	2.93404E-10	Cluster 7	Neu	DOWN
GLDN	3.42564E-10	Cluster 0	Oli	DOWN
FGF14	4.50302E-10	Cluster 9	Neu	DOWN
NSG1	4.64979E-10	Cluster 7	Neu	DOWN
APBB1IP	8.54351E-10	Cluster 1	Mic	DOWN
KIF3A	1.88889E-09	Cluster 7	Neu	DOWN
ZFYVE16	1.98436E-09	Cluster 0	Oli	DOWN
HSPA1B	2.12676E-09	Cluster 2	Ast	UP
RALYL	2.13818E-09	Cluster 9	Neu	DOWN
CARNS1	2.87771E-09	Cluster 3	Oli	DOWN
TIAM1	5.14323E-09	Cluster 9	Neu	DOWN
SNX10	5.88232E-09	Cluster 7	Neu	DOWN
PPP3CA	6.22547E-09	Cluster 9	Neu	DOWN
STMN2	8.12876E-09	Cluster 7	Neu	DOWN
NPAS3	1.02625E-08	Cluster 6	Neu	UP
PLEKHH1	1.068E-08	Cluster 0	Oli	DOWN
TSPYL1	1.26511E-08	Cluster 7	Neu	DOWN
KLHL3	1.49504E-08	Cluster 9	Neu	DOWN
IGSF21	1.49504E-08	Cluster 9	Neu	DOWN
SRRM4	1.49504E-08	Cluster 9	Neu	DOWN
EPB41	1.49504E-08	Cluster 9	Neu	DOWN
CTNNA3	1.51411E-08	Cluster 0	Oli	DOWN
CACNA1A	1.77074E-08	Cluster 9	Neu	DOWN
AL359091.1	1.89004E-08	Cluster 3	Oli	DOWN
RAB6B	1.99932E-08	Cluster 7	Neu	DOWN
MDGA2	2.06594E-08	Cluster 6	Neu	UP
KIF21A	2.49608E-08	Cluster 7	Neu	DOWN
KCND3	2.83236E-08	Cluster 9	Neu	DOWN
HSPB1	3.04917E-08	Cluster 2	Ast	UP
SLC26A3	3.76762E-08	Cluster 3	Oli	DOWN
CAMK4	3.77013E-08	Cluster 9	Neu	DOWN
PSAP	3.79245E-08	Cluster 7	Neu	DOWN
SLC17A6	5.42435E-08	Cluster 7	Neu	DOWN
ENO2	9.78856E-08	Cluster 7	Neu	DOWN
NPTN	9.93365E-08	Cluster 7	Neu	DOWN
HSP90AA1	1.18516E-07	Cluster 3	Oli	UP

EFR3A	1.76577E-07	Cluster 7	Neu	DOWN
RABGAP1L	2.56737E-07	Cluster 9	Neu	DOWN
MSRA	3.27897E-07	Cluster 9	Neu	DOWN
DNER	4.71134E-07	Cluster 7	Neu	DOWN
HSPA1A	8.96263E-07	Cluster 3	Oli	UP
HSPH1	1.12105E-06	Cluster 1	Mic	UP
LRCH1	1.23183E-06	Cluster 9	Neu	DOWN
LINGO1	1.37409E-06	Cluster 3	Oli	DOWN
MAP7D2	1.74094E-06	Cluster 7	Neu	DOWN
UNC13C	2.1177E-06	Cluster 9	Neu	DOWN
PDE1A	2.12292E-06	Cluster 9	Neu	DOWN
ZNF521	2.48268E-06	Cluster 9	Neu	DOWN
COL4A5	2.87927E-06	Cluster 3	Oli	DOWN
HSP90AA1	3.22132E-06	Cluster 1	Mic	UP
GALNT13	4.18235E-06	Cluster 9	Neu	DOWN
PIP4K2A	4.20398E-06	Cluster 6	Neu	UP
SLC14A1	4.29232E-06	Cluster 2	Ast	DOWN
CLTC	4.33542E-06	Cluster 7	Neu	DOWN
TERF2IP	4.46601E-06	Cluster 7	Neu	DOWN
NTRK3	5.24225E-06	Cluster 9	Neu	DOWN
RYR2	5.93555E-06	Cluster 9	Neu	DOWN
RGS7	6.96662E-06	Cluster 9	Neu	DOWN
MAP1B	7.90668E-06	Cluster 7	Neu	DOWN
UCHL1	8.01583E-06	Cluster 7	Neu	DOWN
SERINC1	9.53234E-06	Cluster 7	Neu	DOWN
MOG	1.03566E-05	Cluster 0	Oli	DOWN
NCDN	1.51592E-05	Cluster 7	Neu	DOWN
HSP90AA1	1.73943E-05	Cluster 2	Ast	UP
GABRB1	1.99361E-05	Cluster 2	Ast	DOWN
MCTP1	2.68032E-05	Cluster 9	Neu	DOWN
YWHAZ	3.29825E-05	Cluster 7	Neu	DOWN
P4HA1	4.2445E-05	Cluster 1	Mic	UP
PATJ	8.40131E-05	Cluster 9	Neu	DOWN
LMCD1-AS1	0.00011215	Cluster 3	Oli	DOWN
LMCD1-AS1	0.00011608	Cluster 2	Ast	DOWN
GRIN1	0.000120242	Cluster 7	Neu	DOWN
ST6GAL1	0.000138384	Cluster 1	Mic	DOWN
MICU1	0.00021693	Cluster 9	Neu	DOWN
PRUNE2	0.000236518	Cluster 3	Oli	UP
QDPR	0.000269033	Cluster 2	Ast	UP
PLP1	0.000274736	Cluster 7	Neu	UP
CPLX1	0.000316944	Cluster 7	Neu	DOWN
NEFM	0.000363602	Cluster 7	Neu	DOWN
RTN1	0.000882048	Cluster 7	Neu	DOWN
ETV1	0.000926521	Cluster 9	Neu	DOWN
CALN1	0.001081454	Cluster 9	Neu	DOWN
PPP3CA	0.001346304	Cluster 7	Neu	DOWN
HSPH1	0.001455366	Cluster 2	Ast	UP
SDCBP	0.003187749	Cluster 7	Neu	DOWN

LINC00609	0.003807398	Cluster 0	Oli	DOWN
KIAA1217	0.009682925	Cluster 6	Neu	UP
CNTN4	0.013895365	Cluster 9	Neu	DOWN
SRCIN1	0.022011465	Cluster 3	Oli	DOWN
HSP90AA1	0.025656444	Cluster 0	Oli	UP
IGF1R	0.033855889	Cluster 3	Oli	UP

Figure 1

Click here to access/download;Figure;Figure 1.pdf

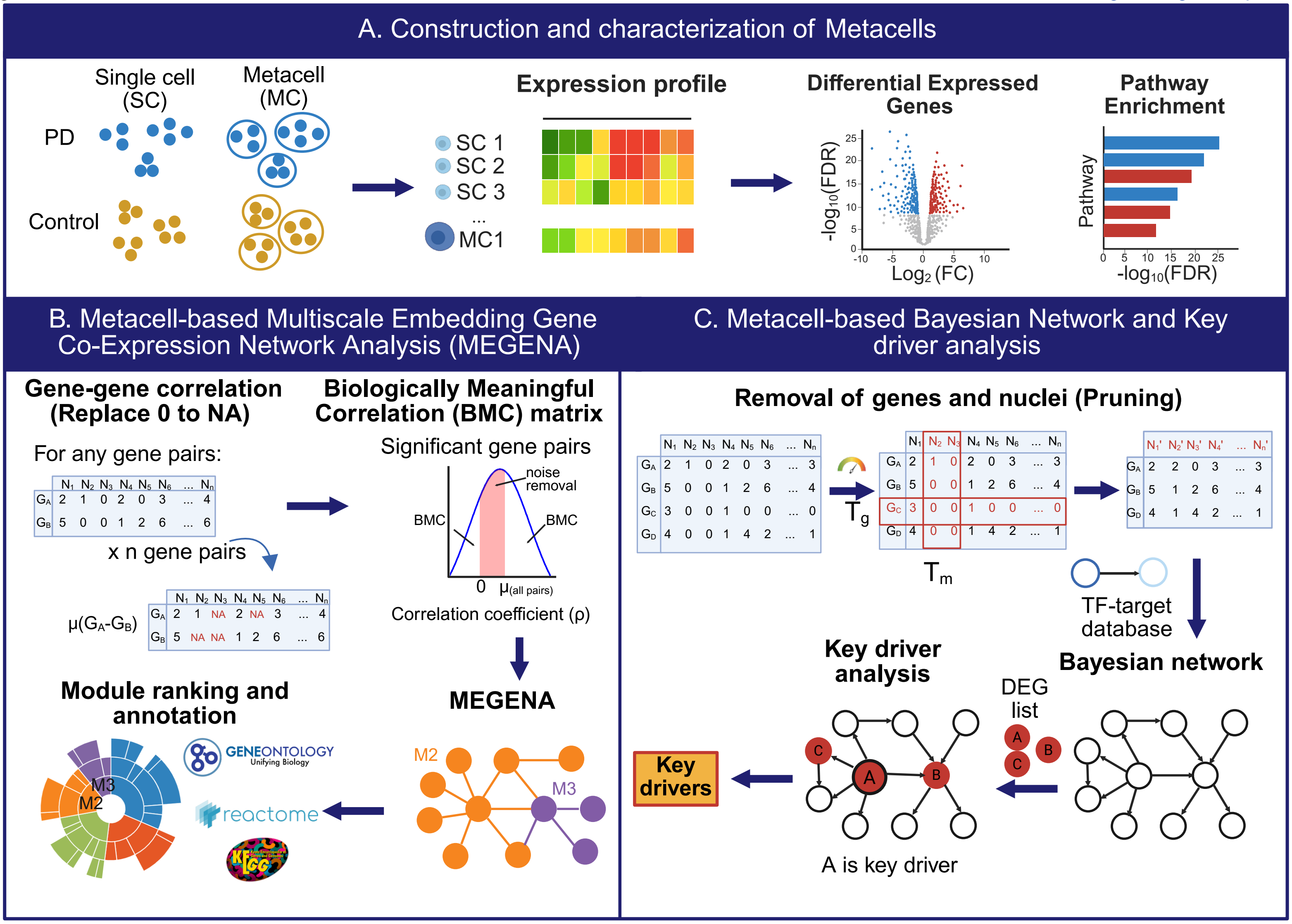

Figure 2

Click here to access/download;Figure;Figure 2.pdf

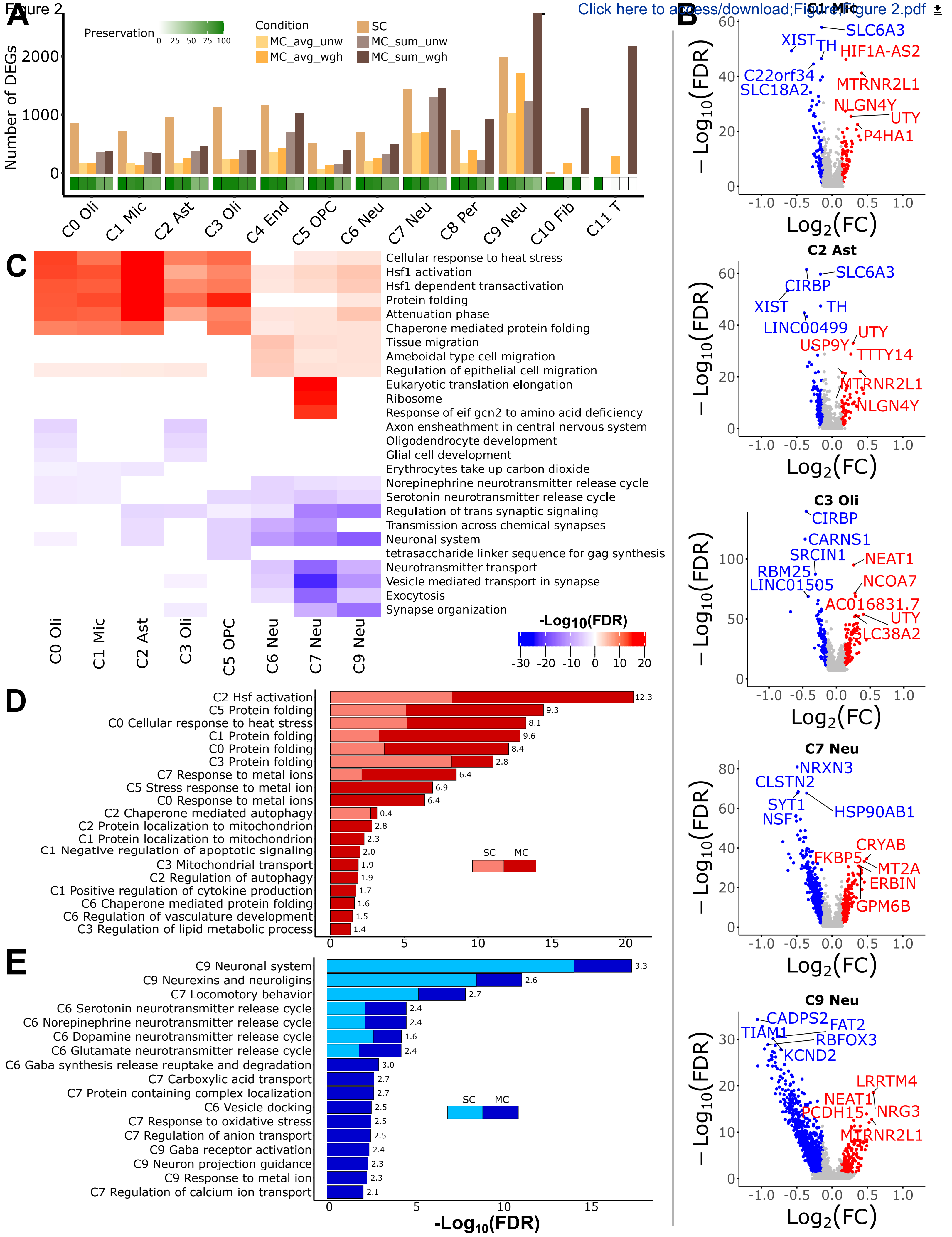

Figure 3

Click here to access/download;Figure;Figure 3.pdf

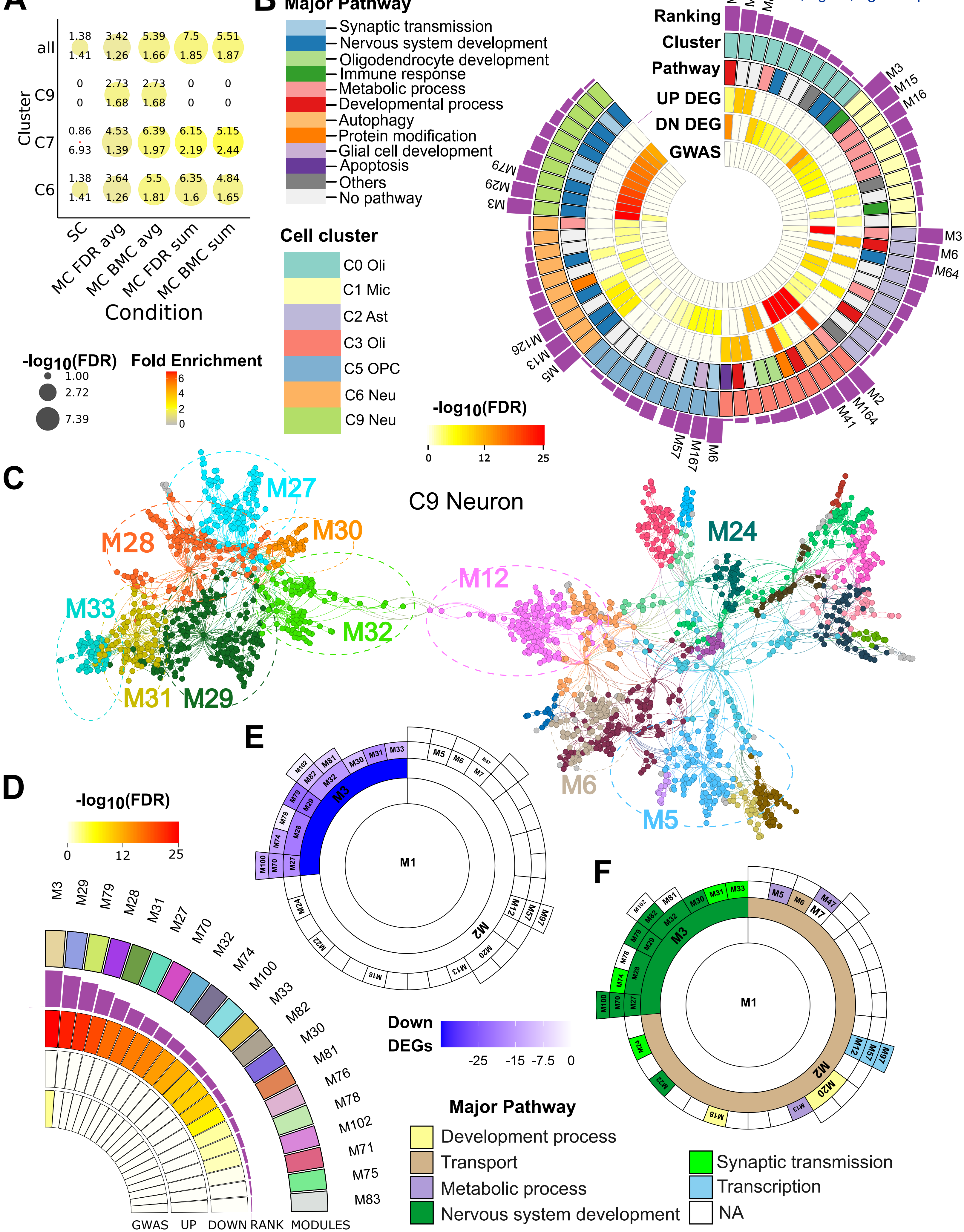

Figure 4

Click here to access/download;Figure;Figure 4.pdf

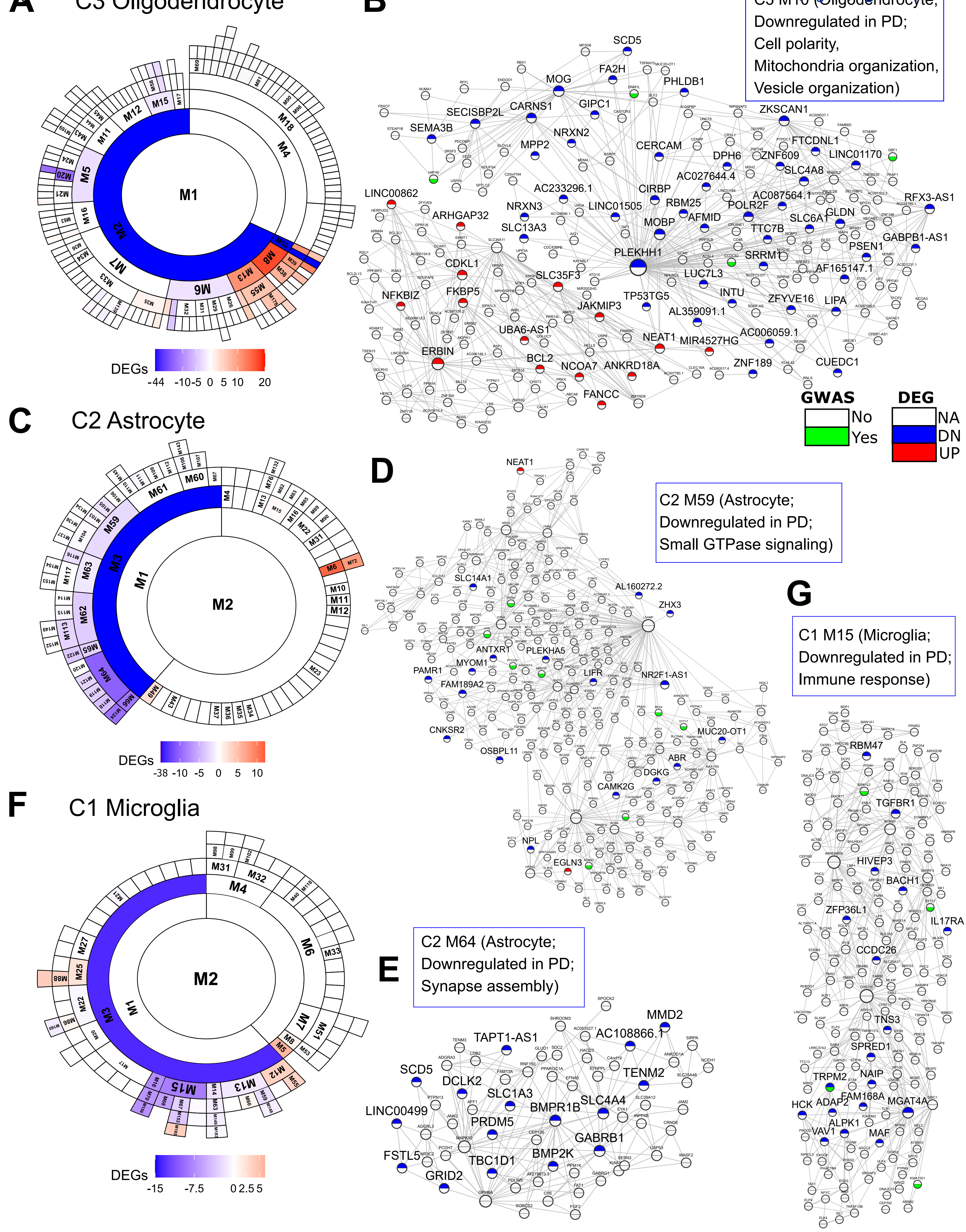

Click here to access/download;Figure;Figure 5.pdf

Figure 5

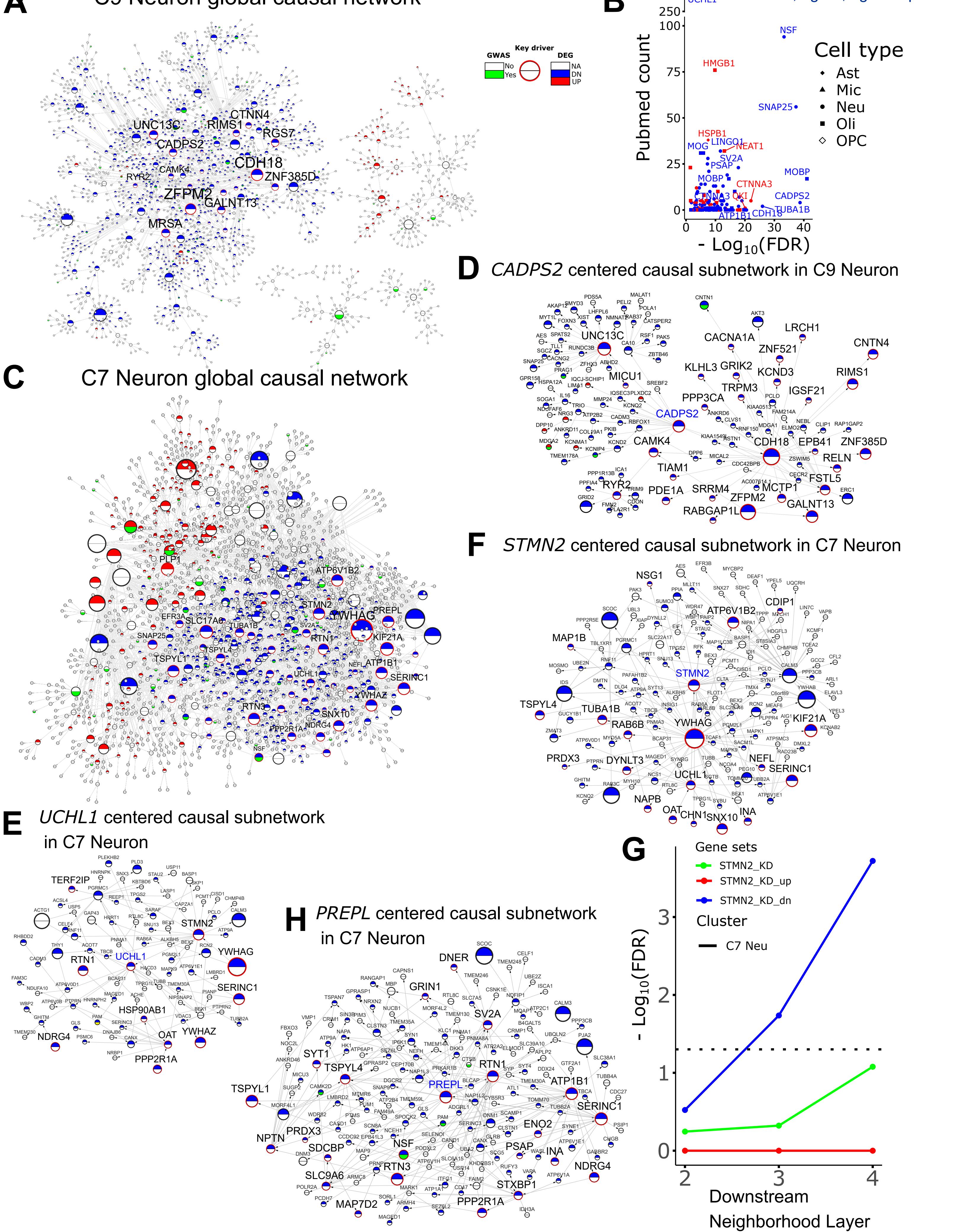

Figure 6 Click here to access/download;Figure;Figure 6.pdf

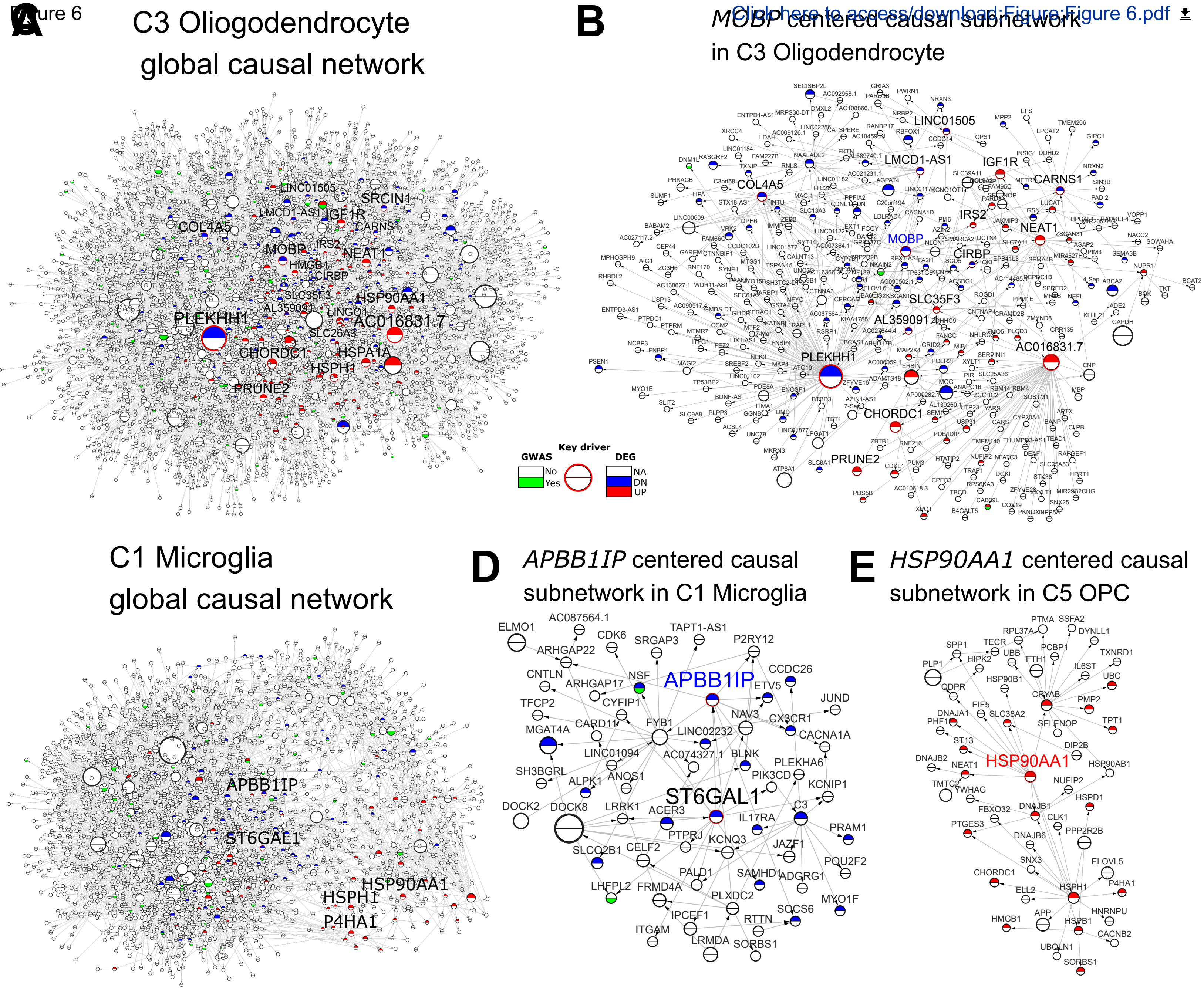

Supplementary Figures S1-S10

Click here to access/download
Supplementary Material
Figure Supplement_final_v5.pdf

Click here to access/download
Supplementary Material
Table S1. Data-Size_SC-and-MC_4-datasets.xlsx

Supplementary Table S2

Click here to access/download

Supplementary Material

Table S2. ComparePathways_SC-and-MC_MSSM-data.xlsx

Supplementary Table S3

Click here to access/download
Supplementary Material
Table S3. DEGs_Metacell_per-Cell-Cluster.xlsx

Click here to access/download
Supplementary Material
Table S4.
ConservedMarkerGenes_Metacell_MSSM.xlsx

Click here to access/download
Supplementary Material
Table S5. Metacell-Sizes_VIB-data.xlsx

Supplementary Table S6

Click here to access/download

Supplementary Material

Table S6. DEG_MC_VIB-data.xlsx

Click here to access/download
Supplementary Material
Table S7. Metacell-Size_KAIST-data.xlsx

Supplementary Table S8

Click here to access/download
Supplementary Material
Table S8. DEG_MC_KAIST-data.xlsx

Supplementary Table S9

Click here to access/download
Supplementary Material
Table S9. Metacell-sizes_Columbia-data.xlsx

Supplementary Table S10

Click here to access/download
Supplementary Material
Table S10. DEG_MC_Columbia-data.xlsx

Supplementary Table S11

Click here to access/download
Supplementary Material
Table S11. Overlap-FET_DEGs_VIB-and-MSSM.csv

Supplementary Table S12

Click here to access/download
Supplementary Material
Table S12. Overlap-FET_DEGs_KAIST-and-MSSM.csv

Click here to access/download
Supplementary Material
Table S13. Overlap-FET_DEGs_Columbia-and-MSSM.csv

Supplementary Table S14

Click here to access/download
Supplementary Material
Table S14. Overlapped-Pathways_MC-SC.xlsx

Click here to access/download
Supplementary Material
Table S15. SummaryStatistics_Up-Pathways_SC-and-MC.csv

Click here to access/download
Supplementary Material
Table S16. SummaryStatistics_DownPathways_SC-and-MC.csv

Supplementary Table S17

Click here to access/download
Supplementary Material
Table S17. Unique-Pathways_MC.xlsx

Supplementary Table S18

Click here to access/download
Supplementary Material
Table S18. Unique-Pathways_SC.xlsx

Supplementary Table S19

Click here to access/download
Supplementary Material
Table S19. Unique-DEGs_MC.xlsx

Click here to access/download
Supplementary Material
Table S20. Pathways_Unique-DEGs_MC.xlsx

Click here to access/download
Supplementary Material
Table S21. Overlap_Unique-DEGs_Columbia-and-KAIST.xlsx

Click here to access/download
Supplementary Material
Table S22. FET_STMN2_Pathway-Preservation.csv

Supplementary Table S23

Click here to access/download
Supplementary Material
Table S23. Gene-Module_Membership.xlsx

Supplementary Table S24

Click here to access/download
Supplementary Material
Table S24. GWAS-Genes.csv

Click here to access/download
Supplementary Material
Table S25. Module-ranking_pathway-annotation.xlsx

Supplementary Table S26

Click here to access/download
Supplementary Material
Table S26. FET_MEGENA Modules.xlsx

Click here to access/download
Supplementary Material
Table S27. Pathways-enriehed_MEGENA-Modules.xlsx

Click here to access/download
Supplementary Material
Table S28. Module-Preservation-Summary.xlsx

Supplementary Table S29

Click here to access/download

Supplementary Material

Table S29. Module-Preservation_FET.xlsx

Click here to access/download
Supplementary Material
Table S30. Module-Preservation_PD-Modules.xlsx

Supplementary Table S31

Click here to access/download
Supplementary Material
Table S31. PD-related-Module-Preservation_FET.xlsx

Supplementary Table S32

Click here to access/download
Supplementary Material
Table S32. ModulePreservation_WGCNA.xlsx

Click here to access/download
Supplementary Material
Table S33. Summary_Module-Preservation_WGCNA.xlsx

Supplementary Table S34

Click here to access/download
Supplementary Material
Table S34. TF-and-genes-removed_by-pruning.xlsx

Click here to access/download
Supplementary Material
Table S35. Key-Driver.xlsx

Supplementary Table S36

Click here to access/download

Supplementary Material

Table S36. Key-drivers_by-2layers.xlsx

Click here to access/download
Supplementary Material
Table S37. Pathways_Key-Driver-NetworkNeighbors.xlsx

Supplementary Table S38

Click here to access/download

Supplementary Material

Table S38. Key-Drivers_Columbia.xlsx

Supplementary Table S39

Click here to access/download

Supplementary Material

Table S39. Shared-Key-Drivers.xlsx

Supplementary Table S40

Click here to access/download
Supplementary Material
Table S40. GWAS-Genes_in_Co-ExpressionNetworks.xlsx

Supplementary Table S41

Click here to access/download

Supplementary Material

Table S41. Gene-Pairs_with-without-BMC.xlsx

Click here to access/download
Supplementary Material
Table S42. DEPs_15PD-15Ctrl.csv

Supplementary Table S43

Click here to access/download

Supplementary Material

Table S43. DEPs_9PD-10Ctrl.csv

Supplementary Table S44

Click here to access/download

Supplementary Material

Table S44 Overlap_DEGs_DEPs15PD-15Ctrl.csv

Click here to access/download
Supplementary Material
Table S45. Overlap_DEGs_DEPs9PD-10Ctrl.csv

Personal Cover & responses to reviewers' comments
Click here to access/download;Personal Cover;Cover-and_Responses.pdf

Icahn School of Medicine at Mount Sinai

Bin Zhang, PhD
Willard T.C. Johnson Research Professor of Neurogenetics
Professor, Departments of Genetics & Genomic Sciences, Pharmacological Sciences, and Artificial Intelligence and Human Health
Director, Mount Sinai Center for Transformative Disease Modeling
Icahn Institute of Genomics, Icahn School of Medicine at Mount Sinai

1425 Madison Avenue (Icahn 3-43)
Box 1498
New York, NY 10029
Phone: 212-659-1726
Fax: 212-659-5507
Email: bin.zhang@mssm.edu

September 5th, 2026

Dear Editor,

We thank you and the reviewers for the careful evaluation of our manuscript, "Multiscale Transcriptomic Network Models of Parkinson's Disease at the Single Cell Level" (GIGA-D-26-00192). We appreciate the constructive comments and suggestions, which have greatly helped us improve the clarity, rigor, and reproducibility of our work.

In the revised manuscript, we have:

- performed a sensitivity analysis and showed excluding zero-count observations did not artificially bias the inferred co-expression network structure or preferentially enrich universally highly expressed genes.
- performed a module preservation analysis at the cell type specific level to provide an orthogonal assessment of the robustness and reproducibility of the identified network modules across datasets.
- performed in-depth analyses of the DEGs specifically identified by the metacell analysis and showed they are biologically meaningful.

We have provided a detailed point-by-point response addressing all the major and minor comments. We look forward to hearing from you soon.

Sincerely,

Bin Zhang, PhD
Willard T.C. Johnson Research Professor of Neurogenetics
Professor, Genetics & Genomic Sciences, Pharmacological Sciences, AI & Human Health
Director, Mount Sinai Center for Transformative Disease Modeling
Icahn School of Medicine at Mount Sinai

Responses to Reviewers' Comments

We sincerely appreciate the very thoughtful and constructive comments from the two reviewers, which provided us with valuable insights and opportunities to greatly improve the quality of the manuscript. We have revised the manuscript according to the reviewers' comments and provided a point-by-point response to each suggestion below.

Responses to the reviewers' comments are provided below with our responses given in blue.

Editor

GIGA-D-26-00192
Multiscale Transcriptomic Network Models of Parkinson's Disease at the Single Cell Level
Chu Linh; Gefei Yu; Xianxiao Zhou; Minghui Wang; Zhenyu Yue; Bin Zhang
GigaScience

Dear Prof. Zhang,

Your manuscript "Multiscale Transcriptomic Network Models of Parkinson's Disease at the Single Cell Level" (GIGA-D-26-00192) has been assessed by our reviewers. Although it is of interest, we are unable to consider it for publication in its current form. The reviewers have raised a number of points which we believe would improve the manuscript and may allow a revised version to be published in GigaScience.

Their reports, together with any other comments, are below. Please also take a moment to check our website at https://www.editorialmanager.com/giga/ for any additional comments that were saved as attachments.

In addition, please register any new software application in the bio.tools and SciCrunch.org databases to receive RRID (Research Resource Identification Initiative ID) and biotoolsID identifiers, and include these in your manuscript. Computational workflows should be registered in workflowhub.eu and the DOIs cited in the relevant places in the manuscript. These will facilitate tracking, reproducibility and re-use of your tool.

If you are able to fully address these points, we would encourage you to submit a revised manuscript to GigaScience. Once you have made the necessary corrections, please submit online at:

https://www.editorialmanager.com/giga/

If you have forgotten your username or password please use the "Send Login Details" link to get your login information. For security reasons, your password will be reset.

Please include a point-by-point within the 'Response to Reviewers' box in the submission system. Please ensure you describe additional experiments that were carried out and include a detailed rebuttal of any criticisms or requested revisions that you disagreed with. Please also ensure that your revised manuscript conforms to the journal style, which can be found in the Instructions for Authors on the journal homepage. If the data and code has been modified in the revision process please be sure to update the public versions of this too.

The due date for submitting the revised version of your article is 18 Oct 2026.

I look forward to receiving your revised manuscript soon.

Best wishes,

Weixue Mu
GigaScience
www.gigasciencejournal.com

Response #1: Thank you for this recommendation. The software/code developed for this study has already been archived in Zenodo and assigned a persistent DOI (10.5281/zenodo.20834299]), which is cited in the manuscript to ensure long-term accessibility and reproducibility.

Reviewer #1:

Chu et al.'s manuscript offers a detailed Multiscale Transcriptomic Network Analysis (MCMNA) pipeline, which seeks to decipher the molecular mechanisms of Parkinson's Disease (PD) through the use of single-nucleus RNA-sequencing (snRNA-seq) data.
By applying a metacell aggregation approach to cluster log-normalised gene expression matrices, the authors effectively address the inherent sparsity and noise of snRNA-seq datasets. The integration of Multiscale Embedded Gene Co-expression Network Analysis (MEGENA) with Bayesian probabilistic causal networks successfully identifies both known targets (such as UCHL1 and NSF) and novel putative driver genes (such as CADPS2 and WDR49) across major neuronal and glial cell types.

In summary, the study employs a robust methodology and offers a significant computational framework. However, certain analytical parameters, integration steps, and correlation assumptions require further clarification to ensure full reproducibility and robustness.

Response #2: We sincerely thank the reviewer for the highly positive evaluation of our study and for the insightful and constructive comments and suggestions.

Major Comments

1. The authors outline a method to tackle data sparsity by eliminating zero-values for every gene pair before computing Spearman correlation coefficients for the BMC matrix. While this strategy effectively enhances the overall correlation distribution in comparison to single-cell data, excluding all zero counts may artificially inflate correlation structures, potentially overlooking biologically important transcripts with low expression levels. The authors need to include an additional sensitivity analysis as supplementary material, showing that the complete exclusion of a particular set does not unintentionally favor genes that are universally highly expressed.

Response #3: We appreciated the important suggestion. To evaluate whether excluding zero-count unintentionally favor genes that highly expressed, we performed a sensitivity analysis examining the relationship between each gene's average expression level and its network activity (defined as the node degree in the MEGENA network). For each cluster, we calculated the average gene expression and correlated it with its node degree using Spearman's rank correlation. Across the cell clusters, the observed correlations were generally weak ($|\rho| < 0.2$), indicating that network connectivity was not strongly associated with baseline expression level. Only cluster 2 Astrocyte ($\rho = -0.239$) and cluster 7 Neuron ($\rho = -0.323$) showed modest negative correlation, suggesting that genes with lower average expression tended to have slightly higher network connectivity rather than the opposite. These findings indicate that excluding zero-count observations did not artificially bias the inferred co-expression network structure or preferentially enrich universally highly expressed genes. We have added these results in Figure Supplement 2D, and described them in the manuscript (Lines 198-204, Page 9).

2. The research employs the Harmony R package for merging data and the MAST package for identifying differentially expressed genes, while accounting for covariates including sex, age, and post-mortem interval (PMI). Considering the study independently reprocesses several external snRNA-seq datasets (the Lee, Martirosyan, and Ma datasets), the authors must clearly address how Harmony successfully reduced batch effects within these technologically and biologically diverse groups. Including a UMAP visualisation of the data before and after integration would greatly strengthen confidence in the alignment.

Response #4: This is a misunderstanding as the external snRNA-seq datasets were reprocessed independently using the same analysis pipeline and no cross-dataset batch correction or dataset merging was performed. Here, the consistency across studies was evaluated by comparing module and pathway-level results.

3. The reliability of network module validation through external datasets hinges on Fisher's Exact Test. Given that the FET necessitates stringent threshold standards for identifying gene set overlaps, the authors might overlook more subtle, rank-based preservation patterns across the datasets. Integrating a threshold-free metric like Gene Set Enrichment Analysis (GSEA) with FET might offer a more robust and comprehensive evaluation of module consistency across various neuronal and glial clusters.

Response #5: We thank the reviewer for this helpful suggestion. We agree that Fisher's Exact Test (FET), which relies on discrete gene-set overlap, may not capture more subtle patterns of network preservation across independent datasets. To provide a more comprehensive assessment and complement to our original analysis, we have now performed the WGCNA module preservation analysis at the cell type specific level in addition to FET. This approach evaluates whether the connectivity (if genes maintain similar relative connectivity patterns) and density structure of each module is preserved (if the genes in the module remain strongly interconnected in the test dataset) in an independent dataset, without requiring a predefined threshold for gene-set overlap.

We define a module as highly preserved if Zsummary.pres ≥ 10, moderately preserved if **2 <** Zsummary.pres < 10, and not preserved otherwise. Similar to the previous FET results, moderate-to-high module preservation was highest in oligodendrocytes (43–53%), followed by microglia (50%), astrocytes (40%), and neurons (~8–44%). Overall, PD-associated modules showed greater moderate-to-high preservation. Consistent with the global findings, PD-associated modules showed the highest preservation in oligodendrocytes (87–97%), followed by microglia (95%), astrocytes (81%), OPCs (71%), and neurons (0–29%). Importantly, among neuronal clusters, PD-associated modules showed greater preservation in Cluster 9 and Cluster 6, but not in Cluster 7 (Supplemental Table S30-31).

These analyses provide an orthogonal assessment of the robustness and reproducibility of the identified network modules across datasets and align with the findings using our FET analysis. These analyses were added into the manuscript (Lines 991-1002, Pages 42-43; Lines 539-544, Page 23).

4. Bayesian causal networks were developed by incorporating transcription factor (TF)-target associations from the TFLink repository as priors. The authors should indicate if these priors were

specifically curated for brain- or neural-derived functional genomic information, as transcription factor-target regulatory interactions can show substantial tissue variability.

Response #6: We agree that TF-target regulatory interactions exhibit tissue-specific activity. The TF-target interactions used as priors in our Bayesian causal network analysis were obtained from the TFLink database, which is not restricted to brain- or neural-specific regulatory interactions. Importantly, to our knowledge, there is currently no comprehensive, experimentally validated TF-target regulatory interaction database that is specific to the human substantia nigra or its constituent neuronal and glial cell types. Rather, TFLink is a comprehensive and continuously maintained repository that integrates experimentally validated and manually curated TF-target interactions from multiple public resources and the literature across diverse biological contexts. We selected TFLink because of its broad coverage, high-quality curation, and regular updates, providing a comprehensive set of prior regulatory relationships for Bayesian network inference. These priors were used to constrain the network structure rather than to define brain-specific regulatory programs. We clarified this in the revised manuscript (Lines 1005-1008, Page 43).

Minor Comments

1. Software Provenance: In the GigaScience submission checklist, the authors checked "No" in the Resources section, stating there are no experimental data. However, computational reproducibility requires exact software provenance. The authors should make sure to clearly state the exact version numbers of all R packages used (such as Seurat, SuperCell, MAST, Harmony) in the Methods section.

Response #7: We have updated our method section in the manuscript.

2. The authors observed that weighted aggregation did not consistently enhance downstream analyses while increasing data size, prompting them to choose the unweighted averaging method. Adding the statistical metric used would improve the transparency of the method.
Response #8: We have added supplementary tables (now Supplementary Table S1, S2) to improve the transparency of the method.

Reviewer #2:

Review of "Multiscale Transcriptomic Network Models of Parkinson's Disease at the Single Cell Level"
In this manuscript, the authors present an integrative, data-driven computational framework (MCMNA) to delineate cell-type-specific gene regulatory networks in the substantia nigra of

Parkinson's disease (PD). By combining metacell aggregation with co-expression network analysis (MEGENA) and Bayesian causal network inference, the authors seek to overcome the technical sparsity inherent to single-nucleus RNA sequencing (snRNA-seq) datasets and identify biologically meaningful regulatory modules and putative key drivers across neuronal and glial populations.

This manuscript presents a technically sophisticated computational framework that integrates metacell aggregation, co-expression network analysis and Bayesian causal inference to investigate cell-type-specific molecular networks in Parkinson's disease. The study represents a valuable resource for the field and identifies numerous hypotheses worthy of future investigation. However, several methodological issues—including potential ambient RNA contamination, incomplete validation of the metacell framework, statistical implications of averaging log-normalized expression, dependence on clustering resolution, and overinterpretation of causal and therapeutic implications—should be addressed before the biological conclusions can be fully supported. Addressing these concerns would substantially strengthen the manuscript and improve confidence in the proposed disease mechanisms and candidate regulators.

Response #9: We appreciate the positive evaluation of the study. Please see our responses to the major concerns below.

Major strengths

1. Rigorous analytical framework
The proposed pipeline directly addresses one of the major limitations of single-cell network inference—technical sparsity and dropout. The generation of a Biologically Meaningful Correlation (BMC) matrix to remove weak background correlations prior to co-expression analysis represents a thoughtful strategy to improve network robustness and reduce false-positive interactions.

2. Multiscale characterization of disease-associated networks
The study provides a detailed map of transcriptomic network organization across multiple neuronal and glial populations. In particular, distinguishing the network architecture of the vulnerable RIT2+ (c9) neuronal population from the more compact TH+ (c7) cluster provides potentially important insights into selective neuronal vulnerability in PD.

3. Integration of independent sources of evidence
The prioritization of co-expression modules using both differential expression and PD GWAS enrichment substantially strengthens the biological relevance of the identified networks. This integrative strategy increases confidence that the highlighted modules reflect disease-associated biology rather than statistical artifacts.

4. Identification of putative causal regulators

The Bayesian causal network analysis identifies 138 putative key drivers of PD. Recovery of previously implicated regulators, including STMN2, UCHL1 and NSF, provides confidence in the computational framework, while the identification of numerous novel, cell-type-specific candidates (for example CADPS2 in neurons and MOBP or APBB1IP in glial populations) offers an important resource for future experimental studies.

Major concerns

1. Potential ambient RNA contamination in glial populations

One of the most concerning observations is the presence of canonical dopaminergic neuronal markers (SLC6A3, TH and SLC18A2) among the most significantly downregulated genes in both microglia (c1) and astrocytes (c2) (Figure 2B). These genes are not expected to be expressed in glial populations under normal conditions. This raises the possibility of ambient RNA contamination, doublets, or cell-type misclassification.

The authors should clarify the origin of these signals and describe whether ambient RNA correction methods were applied. Because the downstream differential expression, pathway enrichment and network analyses rely on these gene lists, the robustness of the glial analyses depends on adequately addressing this issue.

Response #10: We thank the reviewer for raising this important concern regarding the detection of dopaminergic markers as differentially expressed genes in glial populations. Several observations suggested that these findings are unlikely to be primarily driven by cell-type misclassification. Cell type annotation was performed at the single-cell level, and the microglia and astrocyte populations retained robust expression of established lineage-specific glial markers without broad neuronal transcriptional signatures. In addition, doublets were carefully identified and removed during quality control, further reducing the likelihood that neuronal-glial doublets account for these signals in our previous publication[1].

We acknowledge that that ambient RNA correction was not explicitly performed. However, TH, SLC6A3, and SLC18A2 were detected at relatively low levels in glial cells, and their identification as differentially expressed genes should not be interpreted as indicating high or canonical expression of these genes in glia. Notably, these genes were not significantly differentially expressed at the single-cell level but emerged in the metacell analysis. This is consistent with the increased statistical power of metacell approach, and therefore can reveal subtle disease-associated expression differences.

Importantly, the presence of dopaminergic-associated transcripts in glial populations is not without precedent. Martirosyan et al. [2] reported TH-enriched microglial and astrocytic populations and observed their depletion in PD, providing independent evidence that TH-

associated transcriptional signals can be detected in glial populations in the human substantial nigra. We have clarified these points in the revised manuscript (Lines 490-493 , Page 21).

2. Validation of the metacell strategy remains incomplete
The primary motivation for metacell aggregation is to improve signal-to-noise ratio and recover biological signals that are difficult to detect in conventional single-cell analyses. However, the current validation primarily demonstrates preservation of DEGs identified using standard single-cell approaches. While this establishes consistency, it does not demonstrate that the additional DEGs uniquely identified by the metacell approach represent genuine biological discoveries. The authors should provide additional validation demonstrating that these unique findings reflect improved biological sensitivity rather than aggregation-induced artifacts. Replication in independent datasets, enrichment for established biological pathways, or orthogonal experimental validation would substantially strengthen this conclusion.

Response #11: We thank the reviewer for this important suggestion. To determine whether the additional DEGs detected by the metacell analysis represent biologically meaningful signals rather than aggregation-induced artifacts, we performed pathway-enrichment analysis specifically on the metacell-specific DEGs, which are genes identified as significant by the metacell approach but not by the conventional single-cell analysis (Supplementary Table S19)

The metacell-specific DEGs showed biologically coherent, cell-type-associated enrichment. Across the glial populations, these genes were enriched in pathways related to responses to cadmium and zinc ions and the regulation of ion homeostasis (driven by *MT1M, MT1G*, *MT1X*, *MT1E*) (Supplementary Table S20) Various studies have shown that changes in metal ion homeostasis may lead to damage to the CNS, in neurodegeneration, and in PD[3]. In neuronal populations, the metacell-specific genes were enriched in pathways associated with synaptic transmission, consistent with neuronal function.

We further compared these metacell-specific DEGs with DEG results from two independent datasets. This comparison identified recurrent genes (e.g. *BAG3*, *DNAJA4*, *STIP1*, *FKBP4*) involved the heat-shock response, providing independent support for the increased enrichment of stress-response pathways observed in meta cells. In addition, synaptic genes (e.g. *GRIN2B*, *GAD1*) identified in the Columbia's independent dataset overlapped with the metacell-specific gene set, consistent with the stronger enrichment of synaptic pathways detected by the metacell approach (Supplementary Table S21).

Together, these results indicate that the additional DEGs recovered by metacell analysis are not a random collection of genes. Instead, they exhibit cell-type-relevant functional coherence and partial reproducibility across independent datasets. Although these analyses cannot exclude all

possible effects of aggregation, they support the conclusion that the metacell approach improves sensitivity for detecting biologically relevant signals that are less readily identified by conventional single-cell analysis.

We have added these analyses and the corresponding results to the revised manuscript (Lines 165-176, Pages 7-8; Lines 487-490, Lines 493 - 496, Page 21).

3. Averaging log-normalized expression may introduce statistical bias
The authors construct metacells by averaging log-normalized expression values ("avg") rather than summing raw counts followed by normalization ("sum"). Because logarithmic transformation is nonlinear, averaging expression values in log space may artificially reduce within-metacell variance compared with aggregating raw counts before normalization.
Reduced variance could potentially inflate downstream statistical significance and contribute to the larger number of DEGs identified using the "avg" method. The authors should justify this methodological choice and demonstrate that their principal findings are robust to alternative metacell aggregation strategies.

Response #12: Contrary to the concern that averaging normalized expression values might artificially reduce variance and inflate differential expression, we observed the opposite trend in our data: the "sum" strategy consistently identified a larger number of DEGs than the "avg" strategy (Fig. 2A, Figure S1A-B; Lines 120-132, Page 6). Therefore, our empirical results do not support the hypothesis that the "avg" approach inflates statistical significance through variance reduction.

We selected the "avg" approach because it produced results that were more consistent with the original single-cell analyses. Specifically, the differential expression patterns and downstream biological interpretations derived from metacells generated using the averaging strategy showed greater concordance with those obtained from the original single-cell data, suggesting that this approach better preserves the underlying biological signals while reducing technical sparsity (Supplementary Fig. 2A-C, Supplementary Table S2).

Our choice is also consistent with the recommendations of the developers of the SuperCell framework, who suggest that averaging normalized expression values is appropriate when metacells are used for downstream analyses such as visualization, cell annotation, co-expression analysis, gene regulatory network inference, and trajectory analysis, as this strategy avoids repeating upstream normalization and scaling procedures while preserving biologically meaningful relationships.

4. Limited cell-type resolution

The analyses rely on relatively broad cell-type clusters, many of which have been shown by recent single-cell studies to contain multiple transcriptionally distinct subpopulations. Since metacell construction depends on the initial clustering, the resulting co-expression and Bayesian networks may be sensitive to clustering resolution.
For example, would the conclusions differ if multiple microglial subpopulations were analyzed independently? Similarly, because PD is associated with selective degeneration of dopaminergic neurons and reactive expansion of certain glial populations, the authors should discuss how changes in cell abundance across disease states might influence metacell construction and downstream network inference.

Response #13: Our study intentionally performed metacell construction within the major annotated cell types rather than finer subpopulations for two reasons.

First, reliable co-expression and Bayesian network inference require a relatively large number of cells to generate robust metacells and stable correlation estimates. Further subdividing already relatively small cell populations would substantially reduce the number of cells available for metacell construction, thereby decreasing statistical power and increasing the risk of unstable network inference. However, the proposed metacell-based network analysis can be performed on any cell subpopulation with a sufficient number of cells in larger single-cell or single-nucleus RNA-seq datasets that will be generated for PD in the future.

Second, our primary objective was to identify cell-type-specific rather than cell-state-specific regulatory networks that are reproducible across multiple independent PD cohorts. Using major cell types provided a consistent annotation framework across datasets despite differences in sequencing depth, sample composition, and clustering resolution.

In our framework, metacells were constructed within each annotated cell type, thereby minimizing the direct influence of changes in global cell-type composition on metacell construction. Consequently, differences in the abundance of one cell type (e.g., dopaminergic neurons) do not directly affect the construction of metacells for another cell type (e.g., microglia or astrocytes).

5. Interpretation of causal mechanisms and therapeutic targets
A conceptual limitation of the study is that differential gene expression largely reflects disease-associated transcriptional changes, many of which are likely secondary consequences rather than primary drivers of neurodegeneration. Although the Bayesian framework attempts to infer causal relationships, the current study remains computational.
Accordingly, the concluding statement in the abstract that "The multiscale network models developed here lay down a foundation for the discovery of novel mechanisms and therapeutic

targets for PD" appears stronger than is currently supported by the presented data. While the framework successfully prioritizes candidate regulators, no experimental evidence is provided demonstrating that these pathways contribute causally to dopaminergic neurodegeneration or represent tractable therapeutic targets. The authors should moderate these conclusions accordingly.

Response #14: We thank the reviewer for this important comment. We agree that disease-associated transcriptional changes may reflect primary drivers, downstream consequences, or compensatory responses, and that our Bayesian network provides computational evidence rather than experimental proof of causality. We have therefore moderated the language in the Abstract and revised the Discussion to emphasize the need for future perturbation studies in relevant in vitro and in vivo models to experimentally validate the predicted key drivers in PD and assess their therapeutic potential (Lines 624-626, Pages 26-27).

Specific comments

1. Figure 1: Please define all variables and abbreviations used in the schematic (e.g., μ, G, N and TF). The figure legend should more explicitly describe each analytical step.

Response #15: We thank the reviewer for this comment. We have updated the figure legend.

2. Please write out the full term for FET in the Figure S1 legend and provide references for the VIB, KAIST and Columbia datasets.

Response #16: We thank the reviewer for this comment. We have updated the figure legend.

3. In Figure S3B, μ(SC) appears to be missing.

Response #17: Due to high sparsity and small sample size, a co-expression network of the neuron Cluster 9 was unable to be constructed. Therefore, there is no correlation distribution of network in Cluster 9 in Figure S3B.

4. The manuscript uses PD GWAS loci to prioritize network modules. Because most GWAS variants reside in non-coding regions, the authors should clarify how variants were assigned to genes. Were loci mapped using nearest-gene assignment, eQTL colocalization, chromatin interaction data (Hi-C), TWAS, or another functional annotation strategy?

Response #18: In this study, we did not perform de novo mapping of GWAS variants to genes. Instead, we used a published, manually curated PD GWAS gene resource, in which GWAS loci had already been assigned to genes based on the methodology described by the original study.

Therefore, gene assignment was determined by the curation strategy of the published database rather than by nearest-gene mapping or an in-house functional annotation pipeline.

5. The mathematical definition of the summary score is insufficiently described. Please provide the explicit equation used to combine DEG and GWAS enrichment statistics, including the weighting of each component.

Response #19: We have included the equation in the method section to describe our methodology (Lines 976-980, Page 42).

$$Module\ Score_m = \frac{-log_{10}\left(P_{adj,m}^{Upregulated\ DEG}\right)+-log_{10}\left(P_{adj,m}^{Downregulated\ DEG}\right)+-log_{10}(P_{adj,m}^{GWAS})}{3},$$

where $P_{adj,m}^{Upregulated\ DEG}$, $P_{adj,m}^{Downregulated\ DEG}$, and $P_{adj,m}^{GWAS}$ are the adjusted P-values from the FET enrichment test for the DEGs and GWAS signatures, respectively, for module m. Higher scores indicate modules with stronger combined enrichment for disease associated transcriptional and genetic signals.

6. The manuscript identifies cluster c9 as a highly vulnerable neuronal population characterized by RIT2 expression. Recent human substantia nigra snRNA-seq studies have consistently identified SOX6+/AGTR1+ dopaminergic neurons as the population exhibiting the greatest vulnerability in PD. Does cluster c9 (or its dysregulated M3 module) significantly overlap with these established vulnerable neuronal signatures, or does RIT2 define an independent axis of selective vulnerability? Could the authors comment on this in the discussion?

Response #20: We thank the reviewer for raising this important point. Previous studies using midbrain datasets and mouse models have identified SOX6+/AGTR1+ dopaminergic neuronal populations as particularly vulnerable in PD. We examined these markers in our results, however, neither SOX6 nor AGTR1 was identified as a defining feature of cluster c9 or as a gene member of the dysregulated M3 module (Supplementary Table S23). Therefore, based on the current dataset, we do not find evidence that the RIT2-associated c9 population directly corresponds to the previously described SOX6+/AGTR1+ vulnerable dopaminergic population.

Importantly, our study directly profiles the human substantia nigra, a primary site of dopaminergic neuronal degeneration and a region central to PD pathology, providing a complementary disease-relevant context to findings derived from broader midbrain datasets and mouse models. The absence of the SOX6+/AGTR1+ signature in c9 therefore raises the possibility that RIT2 and the

M3 network capture an additional axis of selective neuronal vulnerability that is particularly relevant to the human substantia nigra. Nevertheless, differences in species, anatomical sampling, cellular composition, and analytical resolution may contribute to the observed differences, and the lack of overlap alone does not establish that these vulnerability mechanisms are independent. Further investigation, including direct integration with larger substantia nigra single-nucleus datasets and experimental characterization of RIT2-expressing neuronal populations, will be needed to determine the relationship between these vulnerability signatures. We have added this point to the Discussion (Lines 516-525, Page 22-23).

7. Construction of the Bayesian causal networks involved removing genes expressed in fewer than 40% of metacells (Tg = 0.4). While this filtering step may improve computational stability, it likely biases the resulting networks against lowly expressed transcription factors and other regulatory genes. The authors should report how many known PD-associated transcription factors were removed during this pruning step and discuss the resulting bias in their key driver analysis.

Response #21: We thank the reviewer for raising this important point. To assess whether the Tg = 0.4 pruning threshold disproportionately excluded transcriptional regulators, we quantified the removal of known transcription factors (TFs) and compared it with the removal of genes overall. Across all of the cell types, the percentages of removed TF, were similar to, and in some cases, slightly lower than, the overall percentage of genes removed during the pruning process (Supplementary Table S34), suggesting that the Tg = 0.4 filtering step did not preferentially deplete known PD-associated TFs relative to the background gene set. Nevertheless, we agree that an expression-based threshold inherently reduces representation of genes with sparse or low expression, including individual lowly expressed regulatory genes. Consequently, such regulators cannot be evaluated as potential key drivers if they are excluded before Bayesian network construction. We have therefore added the number and proportion of TFs removed by the filtering step and clarified this limitation in the revised manuscript (Lines 337-341, Page 14).

8. Approximately 70% of the predicted key drivers are described as novel. Validation based primarily on recovery of a single experimentally supported regulator (STMN2) provides only limited confidence in the broader set of newly identified candidates. Additional benchmarking using independent perturbation datasets, CRISPR screens, published regulatory networks or external validation datasets would substantially strengthen confidence in these predictions.

Response #22: We thank the reviewer for this important comment. We agree that validation based primarily on *STMN2* provides limited support for the broader set of novel key drivers. Currently, large-scale CRISPR screening and perturbation datasets in PD-relevant neuronal

models, particularly dopaminergic neurons, remain limited, restricting systematic independent validation of these predictions. We have acknowledged this limitation in the Discussion and emphasized that future studies using CRISPR screens and other functional perturbation approaches in relevant cellular and in vivo models will be important for validating the prioritized key drivers and establishing their roles in PD pathogenesis (Lines 619-626, Pages 27).

Editor Comments:
GigaScience has also published a number of relevant papers that may help contextualize your work. We have listed several recent examples below for your reference. Citing some of these articles, where appropriate in the Background or Discussion, would help frame your contribution within ongoing community efforts and strengthen the presentation of related methods and findings.
1. Bhattacharya, et al. Celltype transcriptomic modules reveal shared molecular mechanisms in Alzheimer's and Parkinson's d. GigaScience. 2026.
https://doi.org/10.1093/gigascience/giag059/8689923.
2. Liu, et al. Multi-omics and high-spatial-resolution omics: deciphering complexity in neurological disorders. GigaScience. 2025.
https://doi.org/10.1093/gigascience/giaf137/8371776.

Response #23: We thank the editor for bringing these relevant studies to our attention. We have incorporated the findings from Bhattacharya et al. into our discussion.

1. Wang Q, Wang M, Choi I, et al. Molecular profiling of human substantia nigra identifies diverse neuron types associated with vulnerability in Parkinson's disease. *Science advances*. 2024;10(2):eadi8287.
2. Martirosyan A, Ansari R, Pestana F, et al. Unravelling cell type-specific responses to Parkinson's Disease at single cell resolution. *Molecular neurodegeneration*. 2024;19(1):1-24.
3. Bhattacharya A, Fon EA, Dagher A, et al. Cell type transcriptomic modules reveal shared molecular mechanisms in Alzheimer's and Parkinson's disease. *GigaScience*. 2026;15:giag059.
