# Supplementary material for: Multiscale transcriptomic network models of Parkinson’s disease at the single-cell level
Source: Gigascience. 2026 Oct 6;15:giag096. doi: 10.1093/gigascience/giag096 (PMC13723071; doi:10.1093/gigascience/giag096)
Supplement: Supplementary file 4 [file giag096_reviewer_1_report_original_submission.pdf]

Reviewer Report

Title: Multiscale Transcriptomic Network Models of Parkinson's Disease at the Single Cell Level

Version: Original Submission **Date:** 7/12/2026

Reviewer name: Reviewer 1

Reviewer Comments to Author:

Chu et al.'s manuscript offers a detailed Multiscale Transcriptomic Network Analysis (MCMNA) pipeline, which seeks to decipher the molecular mechanisms of Parkinson's Disease (PD) through the use of single-nucleus RNA-sequencing (snRNA-seq) data.

By applying a metacell aggregation approach to cluster log-normalised gene expression matrices, the authors effectively address the inherent sparsity and noise of snRNA-seq datasets. The integration of Multiscale Embedded Gene Co-expression Network Analysis (MEGENA) with Bayesian probabilistic causal networks successfully identifies both known targets (such as UCHL1 and NSF) and novel putative driver genes (such as CADPS2 and WDR49) across major neuronal and glial cell types.

In summary, the study employs a robust methodology and offers a significant computational framework. However, certain analytical parameters, integration steps, and correlation assumptions require further clarification to ensure full reproducibility and robustness.

Major Comments

1. The authors outline a method to tackle data sparsity by eliminating zero-values for every gene pair before computing Spearman correlation coefficients for the BMC matrix. While this strategy effectively enhances the overall correlation distribution in comparison to single-cell data, excluding all zero counts may artificially inflate correlation structures, potentially overlooking biologically important transcripts with low expression levels. The authors need to include an additional sensitivity analysis as supplementary material, showing that the complete exclusion of a particular set does not unintentionally favor genes that are universally highly expressed.
2. The research employs the Harmony R package for merging data and the MAST package for identifying differentially expressed genes, while accounting for covariates including sex, age, and post-mortem interval (PMI). Considering the study independently reprocesses several external snRNA-seq datasets (the Lee, Martirosyan, and Ma datasets), the authors must clearly address how Harmony successfully reduced batch effects within these technologically and biologically diverse groups. Including a UMAP visualisation of the data before and after integration would greatly strengthen confidence in the alignment.
3. The reliability of network module validation through external datasets hinges on Fisher's Exact Test. Given that the FET necessitates stringent threshold standards for identifying gene set overlaps, the authors might overlook more subtle, rank-based preservation patterns across the datasets. Integrating a threshold-free metric like Gene Set Enrichment Analysis (GSEA) with FET might offer a more robust and comprehensive evaluation of module consistency across various neuronal and glial clusters.
4. Bayesian causal networks were developed by incorporating transcription factor (TF)-target associations from the TFLink repository as priors. The authors should indicate if these priors were

specifically curated for brain- or neural-derived functional genomic information, as transcription factor-target regulatory interactions can show substantial tissue variability.

Minor Comments

1. Software Provenance: In the GigaScience submission checklist, the authors checked "No" in the Resources section, stating there are no experimental data. However, computational reproducibility requires exact software provenance. The authors should make sure to clearly state the exact version numbers of all R packages used (such as Seurat, SuperCell, MAST, Harmony) in the Methods section.
2. The authors observed that weighted aggregation did not consistently enhance downstream analyses while increasing data size, prompting them to choose the unweighted averaging method. Adding the statistical metric used would improve the transparency of the method.

Methods

Are the methods appropriate to the aims of the study, are they well described, and are necessary controls included? Choose an item.

Conclusions

Are the conclusions adequately supported by the data shown? Choose an item.

Reporting Standards

Does the manuscript adhere to the journal's guidelines on minimum standards of reporting? Choose an item.

Choose an item.

Statistics

Are you able to assess all statistics in the manuscript, including the appropriateness of statistical tests used? Choose an item.

Quality of Written English

Please indicate the quality of language in the manuscript: Choose an item.

Declaration of Competing Interests

Please complete a declaration of competing interests, considering the following questions:

- Have you in the past five years received reimbursements, fees, funding, or salary from an organisation that may in any way gain or lose financially from the publication of this manuscript, either now or in the future?
- Do you hold any stocks or shares in an organisation that may in any way gain or lose financially from the publication of this manuscript, either now or in the future?

- Do you hold or are you currently applying for any patents relating to the content of the manuscript?
- Have you received reimbursements, fees, funding, or salary from an organization that holds or has applied for patents relating to the content of the manuscript?
- Do you have any other financial competing interests?
- Do you have any non-financial competing interests in relation to this paper?

If you can answer no to all of the above, write 'I declare that I have no competing interests' below. If your reply is yes to any, please give details below.

I declare that I have no competing interests

I agree to the open peer review policy of the journal. I understand that my name will be included on my report to the authors and, if the manuscript is accepted for publication, my named report including any attachments I upload will be posted on the website along with the authors' responses. I agree for my report to be made available under an Open Access Creative Commons CC-BY license (http://creativecommons.org/licenses/by/4.0/). I understand that any comments which I do not wish to be included in my named report can be included as confidential comments to the editors, which will not be published.

I agree to the open peer review policy of the journal

To further support our reviewers, we have joined with Publons, where you can gain additional credit to further highlight your hard work (see: https://publons.com/journal/530/gigascience). On publication of this paper, your review will be automatically added to Publons, you can then choose whether or not to claim your Publons credit. I understand this statement.

Yes Choose an item.
