# Supplementary material for: Multiscale transcriptomic network models of Parkinson’s disease at the single-cell level
Source: Gigascience. 2026 Oct 6;15:giag096. doi: 10.1093/gigascience/giag096 (PMC13723071; doi:10.1093/gigascience/giag096)
Supplement: Supplementary file 6 [file giag096_reviewer_2_report_original_submission.pdf]

Reviewer Report

Title: Multiscale Transcriptomic Network Models of Parkinson's Disease at the Single Cell Level

Version: Original Submission **Date:** 7/16/2026

Reviewer name: Reviewer 2

Reviewer Comments to Author:

Review of "Multiscale Transcriptomic Network Models of Parkinson's Disease at the Single Cell Level"
In this manuscript, the authors present an integrative, data-driven computational framework (MCMNA) to delineate cell-type-specific gene regulatory networks in the substantia nigra of Parkinson's disease (PD). By combining metacell aggregation with co-expression network analysis (MEGENA) and Bayesian causal network inference, the authors seek to overcome the technical sparsity inherent to single-nucleus RNA sequencing (snRNA-seq) datasets and identify biologically meaningful regulatory modules and putative key drivers across neuronal and glial populations.

This manuscript presents a technically sophisticated computational framework that integrates metacell aggregation, co-expression network analysis and Bayesian causal inference to investigate cell-type-specific molecular networks in Parkinson's disease. The study represents a valuable resource for the field and identifies numerous hypotheses worthy of future investigation. However, several methodological issuesâ€”including potential ambient RNA contamination, incomplete validation of the metacell framework, statistical implications of averaging log-normalized expression, dependence on clustering resolution, and overinterpretation of causal and therapeutic implicationsâ€—should be addressed before the biological conclusions can be fully supported. Addressing these concerns would substantially strengthen the manuscript and improve confidence in the proposed disease mechanisms and candidate regulators.

Major strengths

1. Rigorous analytical framework

The proposed pipeline directly addresses one of the major limitations of single-cell network inferenceâ€—technical sparsity and dropout. The generation of a Biologically Meaningful Correlation (BMC) matrix to remove weak background correlations prior to co-expression analysis represents a thoughtful strategy to improve network robustness and reduce false-positive interactions.

2. Multiscale characterization of disease-associated networks

The study provides a detailed map of transcriptomic network organization across multiple neuronal and glial populations. In particular, distinguishing the network architecture of the vulnerable RIT2+ (c9) neuronal population from the more compact TH+ (c7) cluster provides potentially important insights into selective neuronal vulnerability in PD.

3. Integration of independent sources of evidence

The prioritization of co-expression modules using both differential expression and PD GWAS enrichment substantially strengthens the biological relevance of the identified networks. This integrative strategy increases confidence that the highlighted modules reflect disease-associated biology rather than statistical artifacts.

4. Identification of putative causal regulators
The Bayesian causal network analysis identifies 138 putative key drivers of PD. Recovery of previously implicated regulators, including STMN2, UCHL1 and NSF, provides confidence in the computational framework, while the identification of numerous novel, cell-type-specific candidates (for example CADPS2 in neurons and MOBP or APBB1IP in glial populations) offers an important resource for future experimental studies.
Major concerns
1. Potential ambient RNA contamination in glial populations
One of the most concerning observations is the presence of canonical dopaminergic neuronal markers (SLC6A3, TH and SLC18A2) among the most significantly downregulated genes in both microglia (c1) and astrocytes (c2) (Figure 2B). These genes are not expected to be expressed in glial populations under normal conditions. This raises the possibility of ambient RNA contamination, doublets, or cell-type misclassification.
The authors should clarify the origin of these signals and describe whether ambient RNA correction methods were applied. Because the downstream differential expression, pathway enrichment and network analyses rely on these gene lists, the robustness of the glial analyses depends on adequately addressing this issue.
2. Validation of the metacell strategy remains incomplete
The primary motivation for metacell aggregation is to improve signal-to-noise ratio and recover biological signals that are difficult to detect in conventional single-cell analyses. However, the current validation primarily demonstrates preservation of DEGs identified using standard single-cell approaches. While this establishes consistency, it does not demonstrate that the additional DEGs uniquely identified by the metacell approach represent genuine biological discoveries.
The authors should provide additional validation demonstrating that these unique findings reflect improved biological sensitivity rather than aggregation-induced artifacts. Replication in independent datasets, enrichment for established biological pathways, or orthogonal experimental validation would substantially strengthen this conclusion.
3. Averaging log-normalized expression may introduce statistical bias
The authors construct metacells by averaging log-normalized expression values ("avg") rather than summing raw counts followed by normalization ("sum"). Because logarithmic transformation is nonlinear, averaging expression values in log space may artificially reduce within-metacell variance compared with aggregating raw counts before normalization.
Reduced variance could potentially inflate downstream statistical significance and contribute to the larger number of DEGs identified using the "avg" method. The authors should justify this methodological choice and demonstrate that their principal findings are robust to alternative metacell aggregation strategies.
4. Limited cell-type resolution
The analyses rely on relatively broad cell-type clusters, many of which have been shown by recent single-cell studies to contain multiple transcriptionally distinct subpopulations. Since metacell construction depends on the initial clustering, the resulting co-expression and Bayesian networks may be sensitive to clustering resolution.
For example, would the conclusions differ if multiple microglial subpopulations were analyzed

independently? Similarly, because PD is associated with selective degeneration of dopaminergic neurons and reactive expansion of certain glial populations, the authors should discuss how changes in cell abundance across disease states might influence metacell construction and downstream network inference.

5. Interpretation of causal mechanisms and therapeutic targets

A conceptual limitation of the study is that differential gene expression largely reflects disease-associated transcriptional changes, many of which are likely secondary consequences rather than primary drivers of neurodegeneration. Although the Bayesian framework attempts to infer causal relationships, the current study remains computational.

Accordingly, the concluding statement in the abstract that "The multiscale network models developed here lay down a foundation for the discovery of novel mechanisms and therapeutic targets for PD" appears stronger than is currently supported by the presented data. While the framework successfully prioritizes candidate regulators, no experimental evidence is provided demonstrating that these pathways contribute causally to dopaminergic neurodegeneration or represent tractable therapeutic targets. The authors should moderate these conclusions accordingly.

Specific comments

1. Figure 1: Please define all variables and abbreviations used in the schematic (e.g., Î¼, G, N and TF). The figure legend should more explicitly describe each analytical step.
2. Please write out the full term for FET in the Figure S1 legend and provide references for the VIB, KAIST and Columbia datasets.
3. In Figure S3B, Î¼(SC) appears to be missing.
4. The manuscript uses PD GWAS loci to prioritize network modules. Because most GWAS variants reside in non-coding regions, the authors should clarify how variants were assigned to genes. Were loci mapped using nearest-gene assignment, eQTL colocalization, chromatin interaction data (Hi-C), TWAS, or another functional annotation strategy?
5. The mathematical definition of the summary score is insufficiently described. Please provide the explicit equation used to combine DEG and GWAS enrichment statistics, including the weighting of each component.
6. The manuscript identifies cluster c9 as a highly vulnerable neuronal population characterized by RIT2 expression. Recent human substantia nigra snRNA-seq studies have consistently identified SOX6+/AGTR1+ dopaminergic neurons as the population exhibiting the greatest vulnerability in PD. Does cluster c9 (or its dysregulated M3 module) significantly overlap with these established vulnerable neuronal signatures, or does RIT2 define an independent axis of selective vulnerability? Could the authors comment on this in the discussion?
7. Construction of the Bayesian causal networks involved removing genes expressed in fewer than 40% of metacells (Tg = 0.4). While this filtering step may improve computational stability, it likely biases the resulting networks against lowly expressed transcription factors and other regulatory genes. The authors should report how many known PD-associated transcription factors were removed during this pruning step and discuss the resulting bias in their key driver analysis.
8. Approximately 70% of the predicted key drivers are described as novel. Validation based primarily on recovery of a single experimentally supported regulator (STMN2) provides only limited confidence in the broader set of newly identified candidates. Additional benchmarking using independent perturbation

datasets, CRISPR screens, published regulatory networks or external validation datasets would substantially strengthen confidence in these predictions.

Methods

Are the methods appropriate to the aims of the study, are they well described, and are necessary controls included? Choose an item.

Conclusions

Are the conclusions adequately supported by the data shown? Choose an item.

Reporting Standards

Does the manuscript adhere to the journal's guidelines on [minimum standards of reporting?]() Choose an item.

Choose an item.

Statistics

Are you able to assess all statistics in the manuscript, including the appropriateness of statistical tests used? Choose an item.

Quality of Written English

Please indicate the quality of language in the manuscript: Choose an item.

Declaration of Competing Interests

Please complete a declaration of competing interests, considering the following questions:

- Have you in the past five years received reimbursements, fees, funding, or salary from an organisation that may in any way gain or lose financially from the publication of this manuscript, either now or in the future?
- Do you hold any stocks or shares in an organisation that may in any way gain or lose financially from the publication of this manuscript, either now or in the future?
- Do you hold or are you currently applying for any patents relating to the content of the manuscript?
- Have you received reimbursements, fees, funding, or salary from an organization that holds or has applied for patents relating to the content of the manuscript?
- Do you have any other financial competing interests?
- Do you have any non-financial competing interests in relation to this paper?

If you can answer no to all of the above, write 'I declare that I have no competing interests' below. If your reply is yes to any, please give details below.

I declare that I have no competing interests

I agree to the open peer review policy of the journal. I understand that my name will be included on my report to the authors and, if the manuscript is accepted for publication, my named report including any attachments I upload will be posted on the website along with the authors' responses. I agree for my report to be made available under an Open Access Creative Commons CC-BY license (http://creativecommons.org/licenses/by/4.0/). I understand that any comments which I do not wish to be included in my named report can be included as confidential comments to the editors, which will not be published.

I agree to the open peer review policy of the journal

To further support our reviewers, we have joined with Publons, where you can gain additional credit to further highlight your hard work (see: https://publons.com/journal/530/gigascience). On publication of this paper, your review will be automatically added to Publons, you can then choose whether or not to claim your Publons credit. I understand this statement.

Yes Choose an item.
